# Fungal symbiont of an ambrosia beetle possesses high nutrient content and suppresses competing fungi with antimicrobial compounds

**DOI:** 10.1093/ismejo/wraf258

**Published:** 2025-11-20

**Authors:** Maximilian Lehenberger, Yu Pan, Stefanie Ungerer, Michael Reichelt, Daniela Pemp, Christian Paetz, Josef Lehenberger, Niklas Gentsch, Felix Feistel, Peter Gros, Leane Lehmann, Jonathan Gershenzon

**Affiliations:** Max Planck Institute for Chemical Ecology, Department of Biochemistry, Hans-Knöll Straße 8, Jena, Thuringia 07745, Germany; Max Planck Institute for Chemical Ecology, Department of Biochemistry, Hans-Knöll Straße 8, Jena, Thuringia 07745, Germany; CAS Center for Excellence in Molecular Plant Sciences, 319 Yueyang Road, Shanghai 200031, China; Max Planck Institute for Chemical Ecology, Department of Biochemistry, Hans-Knöll Straße 8, Jena, Thuringia 07745, Germany; Max Planck Institute for Chemical Ecology, Department of Biochemistry, Hans-Knöll Straße 8, Jena, Thuringia 07745, Germany; Julius-Maximilians-Universität Würzburg, Lehrstuhl für Lebensmittelchemie, Chair of Food Chemistry, Am Hubland, 97074 Würzburg, Germany; Max Planck Institute for Chemical Ecology, Department of Biochemistry, Hans-Knöll Straße 8, Jena, Thuringia 07745, Germany; Max Planck Institute for Chemical Ecology, Department of Biochemistry, Hans-Knöll Straße 8, Jena, Thuringia 07745, Germany; Max Planck Institute for Chemical Ecology, Department of Biochemistry, Hans-Knöll Straße 8, Jena, Thuringia 07745, Germany; Max Planck Institute for Chemical Ecology, Department of Biochemistry, Hans-Knöll Straße 8, Jena, Thuringia 07745, Germany; Thüringer Landesamt für Landwirtschaft und Ländlichen Raum, Naumburger Straße 9807743 Jena, Germany; Julius-Maximilians-Universität Würzburg, Lehrstuhl für Lebensmittelchemie, Chair of Food Chemistry, Am Hubland, 97074 Würzburg, Germany; Max Planck Institute for Chemical Ecology, Department of Biochemistry, Hans-Knöll Straße 8, Jena, Thuringia 07745, Germany

**Keywords:** ambrosia beetle, nutrition, antimicrobial, acetic acid, biosorption, fungi, terpenes, phenolic compounds

## Abstract

Wood-colonizing beetles are associated with a diversity of microbes many of which are thought to act as mutualists with their beetle hosts, but the evidence is usually anecdotal. The ship-timber beetle *Elateroides dermestoides*, one of the few fungus-farming but nonsocial ambrosia beetles, is described to have a mutualistic relationship with the yeast-like fungus *Alloascoidea hylecoeti*. Here, we tested the hypothesis that *A. hylecoeti* has a high nutrient content thus allowing it to function as a valuable food source for the solitary larvae of *E. dermestoides*, which bore into the wood of dead trees, an extremely nutrient-poor substrate. Our analyses revealed that *A. hylecoeti* is rich in soluble sugars, free amino acids, ergosterol, phosphorus, and potassium compared to the other fungi measured, and also accumulates high amounts of fatty acids, B vitamins and nitrogen. We also tested whether *A. hylecoeti* possesses chemical mechanisms to suppress antagonistic microbes. Extracts from *A. hylecoeti* and chemical compounds produced or accumulated by this fungus were found to significantly inhibit the growth of potentially competing fungi. The active substances include fungal-produced monoterpenes and acetic acid, as well as phenolic compounds accumulated from host tree tissues. Moreover, sufficient acetic acid was released by *A. hylecoeti* to drop the medium pH to as low as 3.6, which inhibited all tested competitors, whereas the growth of *A. hylecoeti* was promoted. Taken together, the nutritional properties and competitive ability of *A. hylecoeti* may make a major contribution to the success of its insect partner, the ship-timber beetle under natural conditions.

## Introduction

The colonization of woody tissue by insects is often promoted by beneficial microbes, and in many cases, such symbiotic associates are a key to insect survival by fulfilling fundamental physiological requirements [1–3]. Among wood-dwelling insects, fungus-farming ambrosia beetles (Curculionidae: Scolytinae and Platypodinae) are well known for their obligate mutualisms with species-specific fungi (jointly termed ambrosia fungi), which they actively cultivate on the walls of their tunnel systems, or galleries ([3, 4] and references therein). In Europe, ambrosia beetle galleries are mostly excavated in hardwoods such as beech or oak, whereas a few species colonize conifers [4, 5]. In these galleries, ambrosia fungi colonize the surrounding woody tissue [5, 6] and line the tunnel walls with asexual fruiting structures (conidiophores) that nurture the beetles and their larvae and are often the sole source of beetle nutrition [3, 5].

Typically, ambrosia beetles possess a highly specialized organ allowing for vertical transmission of mutualistic fungi, the mycetangium or mycangium [7, 8]. Such spore-transmitting structures are known for all ambrosia beetles, but also evolved in some phloem-colonizing bark beetles, which are associated with a broad range of fungi as well [8]. However, ambrosia beetle galleries not only contain mutualistic ambrosia fungi, but also a huge diversity of other microbes such as yeasts, bacteria, nematodes, and mites with mostly unknown biological effects on the ambrosia beetle-fungus system [9]. Additionally, fungi such as *Aspergillus* spp., *Chaetomium globosum*, and the entomopathogen *Beauveria bassiana* can act as aggressive pathogens within many ambrosia beetle systems, having the potential to overgrow ambrosia cultivars and kill beetles and offspring [4, 10, 11]. Ambrosia beetles may counteract such pathogens through their advanced sociality [12] as most species are known to exhibit parental care, division of labor, and protection of fungal crops by elimination of fungal weeds [4, 12].

Among ambrosia beetles, those of the Lymexylidae show some differences from other groups, but also engage in symbiosis with species-specific fungi [13]. However, knowledge about the ecological roles of these fungi is very limited. The Lymexylidae encompass less than 100 described species [14] (but see also ref. [15] for Lymexyloidea), of which the most common European representative is the ship-timber beetle *Elateroides dermestoides* (former *Hylecoetus dermestoides*). Although the colonization of woody tissue and the dependence on mutualistic fungi match the criteria of a typical ambrosia beetle, the characteristics of *E. dermestoides* differ in many ways. Most importantly, larvae are nonsocial and solitary, drilling large tunnels into the sapwood of hardwoods and softwoods [5, 13, 16]. The larvae inoculate several microbes on their tunnel walls, including the yeast-like fungus *Alloascoidea hylecoeti* (formerly *Ascoidea hylecoeti,* [17]) as well as *Cyberlindnera* sp. in the Saccharomycetales, which are believed to be of crucial importance for this symbiosis [5, 16–18]. To date, it remains unknown how deeply fungi like *A. hylecoeti* penetrate the woody tissue surrounding the gallery walls. However, due to its formation of a vegetative mycelium in contrast to typical single-celled yeasts [17–19], this fungus may grow at least few millimeters inside woody tissue as indicated for other ambrosia beetle fungi [6, 20, 21]. The fungal community of *E. dermestoides* [5, 16, 18] is vectored by adult females through mycetangial pouches at the apex of the ovipositor [5] and is transmitted to offspring during egg laying. This insect is the largest European ambrosia beetle (up to 18 mm) with a long larval stage (>2y), indicating that efficient strategies for competing with other organisms likely evolved to ensure survival in its substrate [22–24].

The sapwood of trees is typically low in nutrients necessary for animal and microbial life, with cellulose, hemicellulose, and lignin as the primary carbon sources and very low amounts of soluble sugars [25]. These carbohydrate and phenolic polymers require specialized enzymes for breakdown [26]. Here, wood-boring insects benefit from symbionts that are able to depolymerize such woody carbon sources [25, 27]. Moreover, elements such as nitrogen, phosphorus, and potassium, which are required for the successful development of all living organisms, are highly limited in sapwood [28–30]. Another dietary requirement of insects is their general dependence on sterols, compounds that are available at only low levels in woody tissue [27, 31]. However, an alternate source of sterols for wood-colonizing insects might be the fungal sterol ergosterol [31, 32]. Typically, fungal tissue is also rich in specific fatty acids as well as certain amino acids and B vitamins essential for an insect’s diet [33–37]. Hence, wood-boring beetles may greatly benefit from mutualistic association with a filamentous fungus able to provide sterols, fatty acids, amino acids, and B vitamins as well as to depolymerize and transform major sapwood components to usable carbon sources [4, 6, 29, 38]. Ambrosia fungi are believed to serve as particularly suitable sources of these nutrients [4, 27, 39–42]. However, few detailed studies on the nutritional quality of these mutualistic fungi have been carried out, and those that have lack much comparison with other mutualistic and non-mutualistic fungi [4, 27, 39–41].

We hypothesized that *A. hylecoeti*, the symbiotic fungus of the ship-timber beetle *E. dermestoides*, is especially rich in various classes of nutrients compared to other fungi as ship-timber beetle larvae must survive in a habitat with low nutrient levels. Thus, we carried out chemical analyses to compare the overall nutritional quality of *A. hylecoeti* with that of other *Alloascoidea* species, related single-cell yeasts, and other beetle and non-beetle-associated filamentous fungi.

Although the wood of freshly dead trees may not be a very nutritious substrate, it is nevertheless a very competitive habitat colonized by an enormous diversity of microbes like wood-degrading basidiomycetes (e.g. *Pleurotus* spp., *Laetiporus* spp., *Grifola* spp., and *Flammulina* spp.) and mycoparasites like *Trichoderma* spp. or *Chaetomium* spp. [43–46]. Typically, such fungi are wind-dispersed but are also passively vectored by wood-feeding insects and can dominate this niche due to their specialization for growth on dead wood [20, 44, 47, 48]. However, for large wood-tunneling insects with long generation times such as *E. dermestoides*, these fungi might be serious threats as they could outcompete the growth of symbionts such as *A. hylecoeti* reducing the insect’s survival chances.

Competition in such microbial systems is often mediated by chemical compounds that are released from fungi or bacteria and enrich the region immediately surrounding them [32, 46, 49]. For bark and ambrosia beetle systems, a few reports have pointed to the mechanisms of competition between mutualist fungi and other microbes [33, 50–54]. For example, ethanol accumulating in weakened or dying trees was found to select for beneficial microbial associates of the ambrosia beetle *Xylosandrus germanus* [55]. Ethanol might function as a beneficial suppressor of microbes in other ambrosia beetle systems as well [56]. However, there is little knowledge about chemical substances mediating microbial competition in most bark and ambrosia beetle systems.

We hypothesized that *A. hylecoeti*, as the symbiotic fungus of *E. dermestoides*, an ambrosia beetle living in the competitive environment of fresh wood, possess chemical mechanisms to ensure its persistence against other fungi. Thus, we tested the activity of *A. hylecoeti* extracts towards several potentially competing fungi and explored the importance of chemical compounds, including *A. hylecoeti*-produced monoterpenes and organic acids, and phenolics acquired from the host tree in suppressing the growth of different types of fungal competitors. As potential competitors, we studied fungi that we either previously observed in logs colonized by *E. dermestoides* in the field (such as *Pleurotus* spp.) or which could potentially colonize the same substrate.

## Materials and methods

### Fungal species and culture media

For the nutritional profiling, we compared *A. hylecoeti* as well as the other two commercially available *Alloascoidea* species (*A. africana*, *Alloascoidea* sp. P395) with other single-celled yeasts (Saccharomycetes), including two *Cyberlindnera* sp. strains [16], which were previously isolated from the *E. dermestoides* system. Additionally, we included five further yeasts (*Kuraishia capsulata*, *Yamadazyma scolyti*, *Wickerhamomyces bisporus*, *Nakazawaea holstii*, and *Danielozyma ontarioensis*) associated with the bark beetle *Ips typographus* [33, 57] as well as with an ambrosia beetle in case of *D. ontarioensis* [58]. We also analyzed other beetle and non-beetle associated filamentous fungi including some fungi the insect host will likely face in the field. Of these, seven are well-known symbionts of bark and ambrosia beetles from five ascomycete orders (2 × Ophiostomatales, 2 × Microascales, 1 × Saccaromycetales, 1 × Phaffomycetales, 1 × Serinales). The free-living nematophagous fungus *Esteya vermicola* (Ophiostomatales) was included as a bark beetle-associated, but nonmutualistic species [59, 60]. Further, we included three well-known potential pathogens of bark and ambrosia beetles from three ascomycete orders (1 × Eurotiales, 1 × Sordariales, 1 × Hypocreales) as well as seven wood-degrading basidiomycetes [potential competitors of *A. hylecoeti*] from two orders (5 × Agaricales, 2 × Polyporales). For a more detailed overview of all strains and additional information see Suppl. Table S1.

We maintained all fungal species at −80°C in glycerol / water (40 / 60) stocks at the Max Planck Institute for Chemical Ecology (Jena, Germany). Prior to individual experiments, fungi were revived on 4% potato dextrose agar (PDA; Sigma Aldrich, Germany) and maintained at 4°C until they were subcultured on fresh PDA at room temperature (RT) for 4–6 days before experimental use. All nutritional analyses as well as the identification of phenolic compounds and the ^13^C labeled glucose experiment were performed on 5% beech sawdust (Mycogenetics, Germany) with 2% agar (Roth, Germany) and water. Additionally, media were supplemented with 50 μg/ml of penicillin (Thermo Scientific, Germany) and streptomycin (Duchefa Biochemie, Haarlem) to prevent bacterial contamination.

Measurement of phenolic accumulation and metabolism as well as all bioassays were done on PDA with 2% agar whereas all experiments utilizing liquid media were performed in 2% potato dextrose broth (PDB, Sigma Aldrich, Germany), which was in some cases supplemented with 2% beech sawdust. Generally, we placed a circular autoclaved slice of cellophane (PureNature, Germany) on petri dishes allowing us to separate fungal biomass from colonized medium. See SI Methods for further details.

### Nutritional analyses

We inoculated a plug of pure mycelium (Ø 6 mm) from each filamentous fungus on a nutrient-poor wood-based medium. For single-celled yeasts, we inoculated approx. 15 mg of pure yeast biomass (washed twice with water; fresh weight) on each petri dish. Beech sawdust was selected as the wood source as the majority of fungi in our study naturally occur on hardwoods such as beech. The two mutualist fungi studied, *E. polonica* and *G. penicillata*, as well as the four yeasts *K. capsulata*, *Y. scolyti*, *N. holstii*, and *W. bisporus* are typically found with *Ips typographus* and thus on spruce (*Picea abies*), but were still inoculated on beech wood medium to allow comparison with the remaining fungi. In general, fungal growth on our minimal medium was very slow and yielded low amounts of biomass for filamentous fungi as well as for single-celled yeasts. Therefore, we utilized large petri dishes (145 × 20 mm, Greiner, Germany) from which we prepared 6–10 replicates per fungus for analyses of amino acids, sugars, and B vitamins. For all further nutritional analyses, we focused on filamentous fungi including *A. hylecoeti*. For the ergosterol measurements, we prepared another six replicates per fungus, whereas the identification of fatty acids and the elemental analysis required higher amounts of fungal biomass. For fatty acid analyses, harvested biomass was pooled to ensure at least 6–7 individual replicates per fungus. For the elemental characterization, seven replicates were required for the quantification of nitrogen and carbon whereas the remaining seven replicates were required for the analysis of 12 further elements. All petri dishes were incubated on cellophane at 25°C and 65% humidity for up to 28 days until petri dishes for each fungus were completely covered. For single-celled yeasts, the plates were incubated under the same conditions as described above for 12 days until sufficient amount of biomass was visible. After the collection of biomass, all samples were freeze dried and immediately processed for further analyses. Data were generally normalized based on the dry weight determined for each sample. Further, we analyzed free amino acids, sugars, and B vitamins from field nests of *E. dermestoides* (*N =* 7) and *X. saxesenii* (*N =* 6) and compared our findings with uninfested wood samples (controls, 2 × *N =* 6) from identical logs. See SI Methods for further details on the analysis of free amino acids, soluble sugars, B vitamins, fatty acids, ergosterol, and the elements as well as statistical methods.

### Accumulation of phenolic compounds in fungal biomass and culture medium

For the identification and quantification of major phenolic compounds in fungal biomass, five representative fungi (*A. hylecoeti* and *R. sulphurea* = Ambrosia beetle fungi; *E. polonica* = Bark beetle fungus; *P. ulmarius* and *C. globosum* = Competing fungi; see also Suppl. Table S1) were chosen. Fungi were inoculated on beech sawdust medium with cellophane on large petri dishes (*N =* 6) as described above for the nutritional analyses and biomass samples were examined. Additionally, we investigated the abundance of the major phenolics in fungal culture medium over different time points to determine if there are any changes depending on the incubation time. Here, we used the same medium and fungi as described above, but inoculated fungi in standard petri dishes (90 × 14 mm, Roth, Germany) on cellophane. After seven different incubation times (7, 10, 12, 14, 17, 21, and 28 days; *N =* 6), cellophane with biomass was removed and medium samples were taken. Methanol extracts were prepared from freeze-dried samples as described above. See SI Methods for further details.

### ^13^C_6_-labeled glucose experiment to clarify the origin of phenolics

To examine if the major phenolics in *A. hylecoeti* culture medium are of fungal or nonfungal origin, we supplemented beech sawdust medium with ^13^C_6_-labeled glucose. See SI Methods for details.

### Catabolism of phenolics from artificially enriched media

The ability of five representative fungi [see above; instead of *P. ulmarius*, we used *P. ostreatus*] to metabolize the major phenolics was examined by inoculating fungi on PDA with cellophane (*N =* 6). Here, PDA medium was supplemented with a mixture of eight phenolics (see Suppl. Table S4 for individual compounds and concentrations [Treatment: high conc.]), which were first dissolved individually in dimethylsulfoxide (DMSO, Sigma Aldrich, Germany). See SI Methods for details.

### Fungal assays using *A. hylecoeti* culture supernatant

To determine if *A. hylecoeti* has any activity towards competing fungi, solid PDA medium was supplemented with 2% of sterilized supernatant from an *A. hylecoeti* PDB culture. Here, a plug (Ø 6 mm) of pure mycelium of *A. hylecoeti* was inoculated in 300 ml of liquid PDB + 2% beech sawdust in a 500 ml glass flask and incubated on a shaker at 140 rcf and 25°C for 20 d. To exclude any contaminations, we transferred biomass as well as medium from this liquid culture to PDA and confirmed the sole presence of *A. hylecoeti* in the liquid culture based on its characteristic growth and citrus smell. The liquid culture was filtered with a coffee filter to separate biomass from medium. Subsequently, the harvested liquid medium was filtered again using a filter with a pore size of 0.45 μm to remove any remaining fungal particles. Afterwards, we sterile-filtered (filter pore size: 0.22 μm) the supernatant and added it to PDA medium at 2% after autoclaving. Five potential competing fungi were chosen (*T. harzianum*, *C. globosum*, *P. ulmarius*, *P. ostreatus*, *A. aegerita*; see also Suppl. Table S1) to test for possible activity. Additionally, we tested the autotoxicity of the supernatant by including *A. hylecoeti* in this experiment. See SI Methods for details.

### Fungal bioassays with chemical compounds associated with *A. hylecoeti*

We examined the effect of 11 compounds associated with *A. hylecoeti* on the growth of the competing fungi used above as well as on *A. hylecoeti* itself. The choice of monoterpenes was based on previous work [24], whereas choice of phenolic and related compounds was based on earlier results in this study. PDA medium was supplemented (after autoclaving) with the individual compounds, which were first dissolved in DMSO. The applied concentrations were based on our previous findings in medium colonized by *A. hylecoeti*, except for the monoterpenes, which we generally added at a concentration of 50 μg/ml. See Suppl. Table S4 for individual concentrations and SI Methods for further details.

### Fungal bioassays using a phenolic blend

To investigate if the increased phenolic content left over in previously cultured medium might influence subsequent fungi, we prepared an artificial blend of eight individual phenolics and added it to PDA medium. Here, we used concentrations of the major phenolics similar to those we determined in medium colonized by *A. hylecoeti* (see Suppl. Table S4 for compounds and applied concentrations). The phenolic blend was prepared from DMSO stocks of the individual compounds and was added to PDA after autoclaving. As a control, we used PDA supplemented with 0.25% DMSO, which reflects the same amount used in the blend. We tested the effect of increased phenolic content in the medium on seven fungi (*R. sulphurea* and *A. hylecoeti* = Ambrosia beetle fungi; *E. polonica* = Bark beetle fungus; *P. ostreatus* and *P. ulmarius* = Competing and wood-degrading fungi; *C. globosum* and *T. harzianum* = Pathogenic fungi; Suppl. Table S1). See SI Methods for more details.

### pH measurements of fungi in liquid cultures

We examined the ability of several fungi to change the pH of culture medium to clarify if pH and the respective pH-lowering compounds might play a role in the *E. dermestoides*–*A. hylecoeti* system. We inoculated six fungi (*R. sulphurea*, *A. grosmanniae* and *A. hylecoeti* = Ambrosia beetle fungi; *P. ostreatus* and *A. aegerita* = Competing, wood-degrading fungi; *C. globosum* = Antagonistic fungus; see also Suppl. Table S1) in liquid PDB medium containing 2% beech sawdust (*N =* 6). Here, one plug (Ø 6 mm) of pure fungal mycelium was transferred to 150 ml of 0.5% PDB medium +2% beech sawdust within a 250 ml glass flask and incubated on a shaker for 9 days at 25°C and 170 rcf. Six further flasks containing blank medium were incubated under the same conditions as described above, serving as the control. See SI Methods for details.

### The influence of pH on fungal growth

The direct effect of the pH on fungal growth was investigated by inoculating six fungi (*A. hylecoeti* and *R. sulphurea* = Ambrosia beetle fungi; *C. globosum, A. aegerita*, *P. ostreatus* and *P. nameko* = Competing and wood-degrading fungi; see also Suppl. Table S1) in liquid PDB media with either a pH of 3.5 or 5.0 (*N =* 6 per fungus/pH). See SI Methods for details.

### Identification of major organic acids

For the identification and quantification of major organic acids in fungal supernatant, six representative filamentous fungi (*A. hylecoeti, R. sulphurea* and *A. grosmanniae* = Ambrosia beetle fungi; *P. ostreatus, F. velutipes* and *C. globosum* = Competing and antagonistic fungi; see also Suppl. Table S1) were chosen. Here, fungi were inoculated in liquid 0.5% PDB medium containing 2% beech sawdust (*N =* 6). See SI Methods for details.

### Quantification of acetic acid

We quantified the presence of acetic acid in fungal liquid cultures of *A. hylecoeti* (*N =* 6) as well as in cultures of two yeasts (*W. bisporus, Y. scolyti*; *N =* 5). The three microbes were inoculated in liquid 3% PDB medium on a shaker for 9 days at 25°C and 170 rcf. Here, one plug (Ø 6 mm) of pure fungal mycelium of *A. hylecoeti* was transferred to 150 ml of medium in a 250 ml glass flask, whereas 100 μl of a liquid yeast preculture in 3% PDB with an OD of 0.1 was used as the inoculum for each yeast replicate. After 9 d, the liquid cultures of *A. hylecoeti* were individually filtered using a coffee filter whereas we separated biomass and medium of yeast cultures via centrifugation in 50 ml Falcon tubes. Biomass was collected and freeze-dried for 4 days to record dry weight. Supernatant was stored at −20°C for 5d until further processed. Additionally, we examined the presence of acetic acid in field nests of *E. dermestoides* (*N =* 6; see above). We used nests that had been stored at −20°C without freeze-drying. Nest material was carefully cut into smaller pieces and subsequently transferred to a 50 ml Falcon tube, and all material was extracted with 10 ml of methanol. Tubes were incubated at RT and gently shaken for 5 h. Afterwards, the extract was filtered using a clean metal mesh to remove nest material and was further slowly evaporated using N_2_ gas on ice until ~1 ml of methanol extract was left. The concentrated extract was centrifuged again at 9400 rcf for 1 min to remove any remaining nest material and was stored at −20°C until further processed. See SI Methods for details.

### Bioassays using acetic acid

Bioactivity was tested by inoculating seven fungi on PDA medium supplemented with three different concentrations (0.0025, 0.05, and 0.125%) of acetic acid (Roth, Germany), which were added to PDA after autoclaving (with a medium temperature of ~56°C). As control, we used PDA without any addition of acetic acid. From each of seven fungi (*R. sulphurea* and *A. hylecoeti* = Ambrosia beetle fungi; *T. harzianum* and *C. globosum* = Pathogenic fungi; *P. ulmarius, P. ostreatus* and *A. aegerita* = Competing, wood-degrading fungi; see also Suppl. Table S1), one plug of pure fungal mycelium (Ø 6 mm) was inoculated on cellophane on freshly prepared medium. All plates (*N =* 7 per fungus / treatment) were incubated at 25°C and 65% humidity and checked daily until the plates for one of the treatments for each fungus were completely covered. Afterwards, fungal biomass was removed and freeze-dried for 4 days to record dry weight. See SI Methods for details.

## Results

### *A. hylecoeti* is rich in amino acids, sugars, B vitamins, ergosterol, fatty acids, and various nutrients

We characterized the nutritional content of *A. hylecoeti*, the yeast-like fungal symbiont of *E. dermestoides* in comparison with two other *Alloascoidea* species, seven single-celled yeasts, and 12 filamentous fungi (Suppl. Table S1 for the individual species). All of the samples were taken from fungi cultivated on a minimal beech sawdust-based medium. (Fig. 1A–C; Suppl. Figs S1–10).

**Figure 1:**
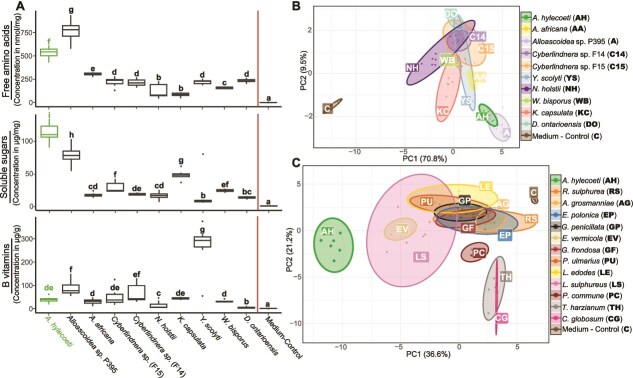
The ship-timber beetle-associated fungus *A. hylecoeti* has a higher nutritional content compared to most other yeasts and filamentous fungi. The nutritional content of fungal biomass was determined with cultures inoculated on 5% beech sawdust medium in large petri dishes until the petri dishes were completely covered or until a sufficient amount of biomass was available in case of the single-celled yeasts. **A** = boxplots comparing *A. hylecoeti* with seven other insect-associated yeasts (including two *Cyberlindnera* strains isolated from *E. dermestoides*) and with two further *Alloascoidea* species. We analyzed total free amino acid content in nmol/mg of dry biomass (upper panel; based on the sum of 18 analyzed amino acids), total soluble sugars in μg/mg dry biomass (middle panel; based on the sum of four analyzed sugars and one sugar alcohol), and total B vitamin content in μg/g dry biomass (lower panel; based on the sum of four analyzed B vitamins). Bold letters above each boxplot indicate significant differences between all six fungi (*N =* 7–10) and the medium control (see Suppl. Table S6 for individual *P* values; GLM with adjusted pairwise contrasts). *A. hylecoeti* is highlighted in green. **B** = PCA of individual concentrations of free amino acids, soluble sugars, and B vitamins of the seven yeasts and two additional *Alloascoidea* species in comparison to those of *A. hylecoeti* (in green) and a medium control. **C** = PCA of individual free amino acids, soluble sugars, B vitamins, ergosterol, and fatty acids of 12 filamentous fungi in comparison to those of *A. hylecoeti* (in green) and a medium control (see Suppl. Table S6 for results of the pairwise PERMANOVAs as well as Suppl. Figs S1–S7).

*A. hylecoeti* accumulated significantly higher amounts of free amino acids in comparison to all of the seven single-celled yeast as well as to *A. africana* (*P* ≤ .0001, GLM with adjusted pairwise contrasts, see Suppl. Table S6 for individual *P* values, Fig. 1A). Indeed, only *Alloascoidea* sp. P395 was found to be richer in its free amino acid content than *A. hylecoeti* (*P* ≤ .002). Most of the essential amino acids, including histidine, methionine, threonine, tryptophan and the branched-chain amino acids isoleucine, leucine, and valine tended to be more abundant in *A. hylecoeti* and *Alloascoidea* sp. P395 than in all of the yeasts (Suppl. Fig. S1A). For soluble sugars, the highest content was found in *A. hylecoeti* compared to all other yeasts, including the remaining two *Alloascoidea* species (*P* ≤ .008; Fig. 1A), due especially to high concentrations of fructose, glucose, and trehalose (Suppl. Fig. S1B). Concerning B vitamins (Fig. 1A), *A. hylecoeti* did not differ in content from most of the analyzed yeasts and *Alloascoidea* species. Most fungi accumulated high concentrations of pantothenic acid (Suppl. Fig. S1C), with *Y. scolyti* containing the highest overall amounts of B vitamins compared to all other analyzed species (*P* ≤ .0001). In general, all fungi showed a significantly higher content of nutrients (free amino acids, soluble sugars, and B vitamins) compared to the medium control without any fungal growth based on the concentrations of the individual nutrients. A PCA analysis revealed a significant difference among the taxa analyzed (PERMANOVA: *F* = 397.9, *R*^2^ = 0.98, *P* = .001; Fig. 1B), and pairwise comparisons indicated that *A. hylecoeti*, which formed a separate clade together with *Alloascoidea* sp. P395, differed significantly from all other analyzed fungi (*P* < .0002, pairwise PERMANOVAs; see Suppl. Table S6 for further details).

Comparing the nutrient content of the yeast-like fungus *A. hylecoeti* with the filamentous fungi, *A. hylecoeti* accumulated significantly higher amounts of free amino acids in comparison to 10 of the 12 tested fungi (*P* ≤ .0011; GLM with adjusted pairwise contrasts, see Suppl. Table S6 for individual *P* values), but did not differ from *E. vermicola* and *Laetiporus sulphureus* (Suppl. Fig. S2A). For the majority of the essential amino acids, *A. hylecoeti* had higher amounts than any of the filamentous fungi (Suppl. Fig. S4). The same trend was true for soluble sugars where *A. hylecoeti* had a far higher total amount compared to the filamentous fungi (Suppl. Fig. S2B, *P* < .0001), due especially to high concentrations of glucose, fructose, and trehalose (Suppl. Fig. S5). Considering other nutrients, *A. hylecoeti* was among the fungi with the highest content of B vitamins, ergosterol, fatty acids, and the elements nitrogen, phosphorus, and potassium (see SI results). A PCA analysis based on the concentration of individual nutrients revealed a statistical difference between the taxa analyzed (PERMANOVA: *F* = 111.5, *R*^2^ = 0.95, *P* = .001; Fig. 1C), where *A. hylecoeti* differed significantly from all other tested fungi (*P* < .0034, pairwise PERMANOVAs; see Suppl. Table S6 for further details).

Taken together, our results demonstrated that the yeast-like fungus *A. hylecoeti* is rich in nutrients compared to both yeasts and filamentous fungi. Analysis of field nests of *E. dermestoides* and another ambrosia beetle, *X. saxesenii*, in beech logs confirmed these trends, showing that nest areas had generally higher concentrations of soluble sugars, B vitamins, and free amino acids than the surrounding uncolonized wood (SI Results).

### *A. hylecoeti* accumulates phenolic compounds in its mycelium and adjacent culture medium

**Figure 2:**
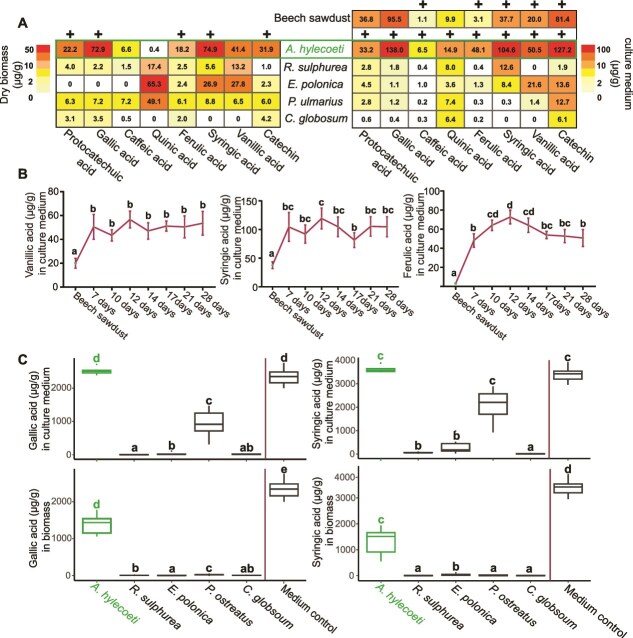
*A. hylecoeti* accumulates host tree-derived phenolics and related compounds in its biomass and culture medium without catabolizing them. **A** = Heatmaps showing the average quantities of eight individual compounds detected by targeted LC–MS analyses in μg/g dry biomass (left; see Suppl. Fig. S9 for an overview of identified phenolic compounds in biomass) and dried culture medium after fungal inoculation (right) with five different fungal species. For chemical analysis, fungi were inoculated on 5% beech sawdust medium in large petri dishes until the petri dishes were completely covered (*N =* 6). The compounds present in culture medium (right heatmap) were determined from 7-day old fungal cultures inoculated in standard petri dishes (*N =* 6, see data of **b** and Suppl. Fig. S11). Samples were only taken from areas on the petri dish that were colonized by the fungus. The **+** symbol above the individual heatmap column indicates a significant increase in the concentration of a specific compound for *A. hylecoeti* in comparison to the remaining four fungi as well as to the beech sawdust medium control (right heatmap, first row) without any fungal growth (for individual *P* values, see Suppl. Table S6; GLM with adjusted pairwise contrasts). *A. hylecoeti* is highlighted in green. **B** = Line plots showing the concentrations of the most abundant phenolics in μg/g over seven different time points (7–28 d, *N =* 6) in 5% beech sawdust medium in standard petri dishes colonized by *A. hylecoeti* in comparison to uninoculated beech sawdust medium (control). After each individual time point, all fungal biomass growing on cellophane was removed and medium samples were analyzed. The control was sampled at the same time as the 28 days treatment. Bold letters above each time point indicate a significant difference between the individual treatments (GLM with adjusted pairwise contrasts, error bars = SD). Here, the three phenolic compounds vanillic acid, syringic acid, and ferulic acid are shown (see Suppl. Fig. S10 and S11 for remaining compounds and fungi). **C** = Boxplots showing the concentration of the most abundant phenolic compounds in μg/g identified in dried culture medium (above) after fungal colonization as well as in dry fungal biomass (below) from five different fungal species. Fungi were inoculated on cellophane using PDA as culture medium (*N =* 6), which was supplemented with a blend of eight compounds (see Suppl. Table S4 for added concentrations: Treatment high conc.). In general, fungi were inoculated until petri dishes were completely covered or up to a max. of 15 days (for fungi that were inhibited by the chemical blend). Biomass and medium (covered from fungus) were analyzed separately for remaining compounds. Bold letters above each boxplot indicate significant differences between fungi and control (PDA with blend but without fungus) (see Suppl. Table S6 for *P* values; GLM with adjusted pairwise contrasts). Here, the two abundant phenolic compounds gallic acid (left side) and syringic acid (right side) are shown (see Suppl. Fig. S12 for remaining six compounds). *A. hylecoeti* is highlighted in green.

To determine if phenolics and phenolic-related compounds might play a role in the interactions between *E. dermestoides* and *A. hylecoeti*, we analyzed the fungal biomass and culture medium of five different fungi inoculated on a minimal beech sawdust-based medium with a focus on eight individual phenolic and related compounds known to be abundant in beech wood (Fig. 2A and Suppl. Fig. S11). Protocatechuic acid, gallic acid, quinic acid, ferulic acid, syringic acid, vanillic acid, and catechin were identified as abundant in the biomass of the five investigated fungi (Fig. 2A, left heatmap), whereas caffeic acid, coumaric acid, and sinapic acid (Suppl. Fig. S11) were less abundant. *A. hylecoeti* accumulated significantly higher concentrations of protocatechuic acid, gallic acid, ferulic acid, syringic acid, and catechin in mycelium compared to the remaining four fungi (*P* < .0031, *P* < .0001, *P* < .0001, *P* < .0032, *P* < .0009, respectively; GLM with adjusted pairwise contrasts, Suppl. Table S6). Next, we analyzed the medium previously colonized by the fungus (separated from fungal biomass via cellophane) and found that *A. hylecoeti* growth had caused the accumulation of higher amounts of protocatechuic acid, gallic acid, caffeic acid, quinic acid, ferulic acid, syringic acid, vanillic acid, and catechin compared to the previously colonized medium of the remaining four fungi (*P* < .00014, *P* < .007, *P* < .0001, *P* < .007, *P* < .0001, *P* < .0001, *P* < .0001, *P* < .0001, respectively). In control medium (beech sawdust), we generally detected high concentrations of the investigated compounds (see Fig. 2A, right heatmap), but found caffeic acid, ferulic acid, vanillic acid, syringic acid, and catechin to be at higher levels still in the medium previously colonized by *A. hylecoeti* (*P* < .0001, *P* < .0001, *P* < .0001, *P* < .0001, *P* < .0007, respectively).

Our results demonstrate that *A. hylecoeti* accumulates specific phenolic compounds in its biomass and culture medium, whereas the remaining four fungi showed very low accumulation of these compounds.

### Phenolic accumulation in *A. hylecoeti* is not due to biosynthesis, but to uptake from host tree tissues without catabolism

To test if the enriched concentrations of phenolics in the culture medium of *A. hylecoeti* are derived from *A. hylecoeti* itself, we supplemented the medium with ^13^C-labeled glucose. Then, we extracted the phenolics and analyzed them by LC–MS to look for evidence of ^13^C-incorporation. However, we could not detect any increased ^13^C-labeling of these phenolic compounds compared to a control fed unlabeled glucose (see Suppl. Table S3). This indicates that these compounds are likely derived from the beech sawdust directly and are not formed by the biosynthetic processes of *A. hylecoeti*.

We tested whether the accumulation of phenolics by *A. hylecoeti* when growing on beech sawdust medium, in contrast to the four other fungi studied, was due to a lack of catabolism. This assumption was supported by the findings of a longer time course experiment over 28 days that showed no decline in concentration (Fig. 2B–C, SI Results and Suppl. Figs S12 and S13). To confirm the inability of *A. hylecoeti* to break down phenolics, we performed bioassays where we supplemented medium with an artificial blend consisting of eight phenolic compounds, on which we inoculated fungi. There was no difference in phenolic concentration between *A. hylecoeti* culture medium and the nonfungal control medium for gallic acid, syringic acid (Fig. 2C, upper row), caffeic acid, protocatechuic acid, quinic acid, catechin, and vanillic acid (*P* > .9, *P* > .9, *P* > .5, *P* > .9, *P* > .9, *P* > .9, *P* > .9, respectively; GLM with adjusted pairwise contrasts, Suppl. Table S6, Suppl. Fig. S14A). Only the level of ferulic acid was strongly reduced by *A. hylecoeti* in culture medium (*P* < .0001). For the remaining fungi, however, the phenolic levels in the culture medium were mostly much lower compared to the nonfungal control medium (gallic acid, caffeic acid, protocatechuic acid, catechin, vanillic acid, syringic acid: *P* < .038, *P* < .0001, *P* < .007, *P* < .0001, *P* < .0001, *P* < .0001 (except *P. ostreatus*: *P* > .7)). Additionally, we found high concentrations of gallic acid, syringic acid, protocatechuic acid, catechin, and vanillic acid in *A. hylecoeti* biomass (Fig. 2C lower row, Suppl. Fig. S14B), that were higher than the phenolic levels in the biomass of the remaining fungi (*P* < .0001). In the biomass of the other fungi tested, phenolics were generally only present in trace amounts or absent entirely (Fig. 2C lower row, Suppl. Fig. S14B) except for quinic acid in biomass of *P. ostreatus*, which showed a similar concentration compared to the medium control.

The mutualist *A. hylecoeti* behaves completely unlike the other fungi tested (two mutualists, one wood degrader and one potential pathogen) by accumulating several beech phenolic compounds in its biomass and not decreasing the levels of most phenolics in its culture medium.

### *A. hylecoeti*-associated monoterpenes and phenolics inhibit the growth of competing fungi

**Figure 3:**
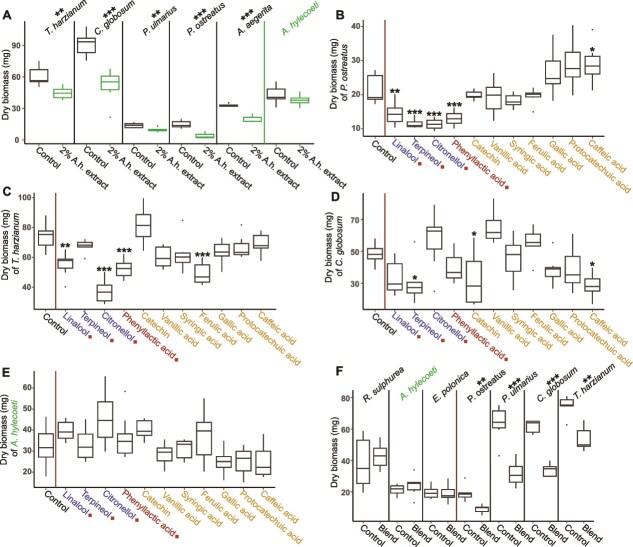
*A. hylecoeti*-associated chemical compounds inhibit the growth of competing fungi. The activity of *A. hylecoeti* extracts and pure compounds was tested against five competing fungi and itself by performing bioassays with sterile-filtered supernatant (**a**; *N =* 6–7), individual compounds (**B–E**, *N =* 6–7), and a blend consisting of eight beech host tree phenolics (**F**, tested with seven fungi, *N =* 5–6). All individual compounds as well as phenolics for the blend (**B–F**) were dissolved in DMSO before adding them to PDA medium. The control medium was supplemented with DMSO at the same concentration. The four compounds marked with an orange symbol (**B–E**) are produced by *A. hylecoeti*, whereas the remaining compounds were found to be accumulated in beech sawdust medium when *A. hylecoeti* is present. Here (**B–E**), monoterpenes are highlighted in blue, phenyllactic acid in red, and phenolic compounds in orange (see Fig. 2). All tested concentrations are listed in Suppl. Table S4. Welch’s two-sample t-test (**a, f**) as well as fitted GLM models and pairwise contrasts including Dunnett’s corrections (**B–E**) were used to identify significance. Asterisks above fungal names or boxplots indicate a significant difference between the treatment and a control (ns - *P* > .05, ^*^ - *P* < .05, ^**^ - *P* < .01, ^***^ - *P* < .001; see Suppl. Table S6 for individual *P* values). **A** = Boxplots showing the effect of 2% *A. hylecoeti* extract (*A. hylecoeti* extract; sterile-filtrated from a liquid 20d old PDB + 2% beech sawdust culture) added to solid PDA medium on growth (mg dry biomass) for five competing fungi as well as on *A. hylecoeti* itself in comparison to control medium (PDA with 2% sterile-filtrated PDB + 2% beech sawdust). *A. hylecoeti* extract treatments are highlighted in green. **B** = Boxplots visualizing the effect of individual compounds on *P. ostreatus*. **C** = Boxplots visualizing the effect of individual compounds on *T. harzianum*. **D** = Boxplots visualizing the effect of individual compounds on *C. globosum*. **E** = Boxplots visualizing the effect of individual compounds on *A. hylecoeti*. See Suppl. Fig. S13 for remaining two fungi. **f** = Boxplots showing the growth of fungi on medium enriched with the phenolic blend in comparison to control medium without phenolic compounds. The red line separates the noninhibited mutualistic fungi *R. sulphurea*, *A. hylecoeti* (highlighted in green), and *E. polonica* from the inhibited, competitive fungi *P. ostreatus*, *P. ulmarius*, *C. globosum*, and *T. harzianum*.

To elucidate the potential of *A. hylecoeti* to deploy chemical compounds to outcompete other fungi, we performed multiple bioassays testing the effect of *A. hylecoeti* culture medium and 11 individual chemicals associated with this fungus on five competing fungi. All competing fungi inoculated on PDA medium supplemented with 2% of sterile-filtrated *A. hylecoeti* culture medium grew significantly less than on a PDA control medium (Fig. 3A). Here, the strongest inhibition was observed for the wood-degrading fungi *A. aegerita* and *P. ostreatus* as well as for the antagonistic fungus *C. globosum* (*P* < .0001, *P* < .0001, *P* < .0003, respectively; t-test), followed by the wood-degrader *P. ulmarius* and the antagonist *T. harzianum* (*P* < .009, *P* < .003, respectively). The filtered supernatant had no effect on the growth of *A. hylecoeti* itself (*P* > .16; t-test). As these results confirmed the general ability of *A. hylecoeti*-associated chemicals to inhibit competing fungi, we then examined individual compounds, including three previously reported monoterpene alcohols (24) and the eight phenolics shown to be accumulated above (Fig. 3B–E, Suppl. Fig. S15). Overall, the growth of each fungus was negatively affected by several of the tested chemicals, whereas the others had neutral or in few cases positive effects at the applied concentration. The monoterpene citronellol completely changed the morphology of *C. globosum* from reddish to whitish, but did not affect its growth, a result that was not observed for any of the other applied compounds. Besides individual compounds, we also tested the effect of an artificial blend (Fig. 3F) consisting of the eight individual phenolic compounds at concentrations found in the *A. hylecoeti* beech culture medium (see Suppl. Table S4). This blend had no effect on the mutualistic fungi *R. sulphurea*, *A. hylecoeti*, and *E. polonica*, but inhibited both wood-degrading fungi *P. ostreatus* and *P. ulmarius* (*P* < .0032, *P* < .0005, respectively; t-test) as well as the two potential myco-pathogens *C. globosum* and *T. harzianum* (*P* < .0001, *P* < .003, respectively; t-test).

In summary, our results demonstrate that several *A. hylecoeti*-associated chemicals efficiently inhibit various competing fungi, whereas *A. hylecoeti* showed no negative effects for any of the tested compounds. Neutral or positive effects observed for competing fungi on single phenolics disappeared when cultivated on the phenolic blend, where all tested fungi except the mutualistic ones were inhibited.

### Acetic acid and low pH favor *A. hylecoeti*, but suppress competing fungi

**Figure 4:**
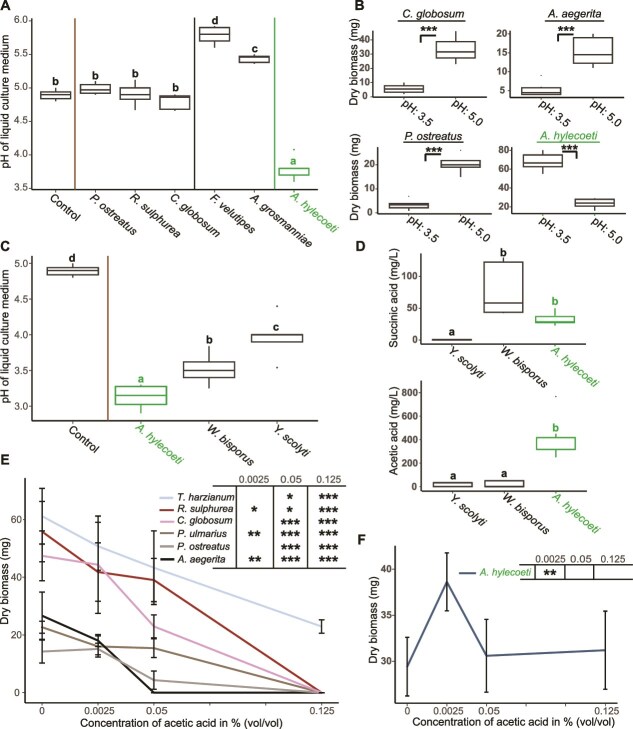
*A. hylecoeti* outcompetes other fungi by releasing acetic acid and lowering medium pH. Bold letters and asterisks indicate a significant difference between fungi or individual treatments (ns - *P* > .05, ^*^ - *P* < .05, ^**^ - *P* < .01, ^***^ - *P* < .001; see Suppl. Table S6 for individual *P* values). *A. hylecoeti* is highlighted in green **A** = Boxplots showing the pH of six representative fungi inoculated in liquid 0.5% PDB + 2% Beech sawdust medium for 9 days (*N =* 6; GLM with adjusted pairwise contrasts) in comparison to medium without fungus (Control). **B** = The effect of pH on the growth of six fungi in mg of dry biomass was investigated by inoculating fungi in liquid PDB cultures (*N =* 6, incubation time = 6 d) with a low pH of 3.5 (simulating pH identified for *A. hylecoeti*) and a moderate pH of 5.0. Significant differences in growth were determined using Welch’s two-sample t-test for each fungus. See Suppl. Fig. S14 g for data of the remaining two fungi. **C** = Boxplots showing the pH of *A. hylecoeti* and two yeasts isolated from the bark beetle *Ips typographus*, inoculated in liquid 3% PDB for 9 days (*N =* 6; GLM with adjusted pairwise contrasts) in comparison to control medium. **D** = major organic acids in mg/L identified from culture supernatant (3% PDB; incubation time = 9 d, *N =* 6) of *A. hylecoeti* and two yeasts (see Suppl. Fig. S16A–F for further identified organic acids from yeast and filamentous fungi supernatants). A GLM with adjusted pairwise contrasts was applied to identify significant differences. **E, F** = Line plots visualizing the inhibitory effect of acetic acid on growth in mg of dry biomass for five antagonistic and competing fungi to *A. hylecoeti* as well as one ambrosia beetle mutualist fungus (*R. sulphurea*) (**E**, Asterisks indicate significant inhibition) as well as the beneficial effect on *A. hylecoeti* (**F**, Asterisks indicate significantly enhanced growth). PDA medium was supplemented with three acetic acid treatments (0.0025, 0.05, and 0.125%, *N =* 7). Pure PDA medium was used as the control (*N =* 7). Error bars are showing the SD. Statistical differences for each fungus were revealed using fitted GLM models and pairwise contrasts including Dunnett’s corrections and are indicated within the tables above **E** and **F**.

As preliminary experiments indicated that *A. hylecoeti* has the capability to decrease the pH of its liquid culture medium, we compared this ability with that of other filamentous fungi that could be competitors to *A. hylecoeti*. Then, we identified the major organic acids involved in pH reduction and performed bioassays to elucidate the impact of these acids on fungal growth (Fig. 4A–F, Suppl. Figs S16–S19).

When we grew *A. hylecoeti* and five potentially competing filamentous fungi in liquid 0.5% PDB medium with 2% beech sawdust *P. ostreatus*, *R. sulphurea*, and *C. globosum* did not influence the pH of their respective media (Fig. 4A), whereas *F. velutipes* and *A. grosmanniae* caused a slight increase (*P* < .0001; GLM with adjusted pairwise contrasts). However, *A. hylecoeti* significantly reduced the pH of its culture medium from an initial value of ~4.9 (control) to ~3.8 (*P* < .0001). We next performed bioassays with filamentous fungi testing the effect of a low (3.5) and a moderate (5.0) pH on fungal growth (Fig. 4B and Suppl. Fig. S15G). Here, all potential competing fungi tested, such as *C. globosum, A. aegerita*, *P. ostreatus* as well as *P. nameko*, were highly inhibited by low pH, compared to growth on a moderate pH (*P* < .0003, *P* < .0007, *P* > .0001, *P* < .023, respectively; t-test). On the other hand, the mutualist *R. sulphurea* exhibited a similar growth rate at both pH conditions (*P* > .19; t-test), whereas *A. hylecoeti* was highly favored by the low pH (*P* < .0001; t-test). As *A. hylecoeti* is a yeast-like fungus (but shows filamentous growth, see also ref. (18)), we additionally included two yeasts in our experiments to clarify if a decrease of pH is typical for yeasts. Two yeasts associated with the bark beetle *Ips typographus*, *W. bisporus* and *Y. scolyti*, together with *A. hylecoeti* were inoculated in 3% PDB medium for 9 days and pH was compared to an uninoculated control (Fig. 4C). All three species significantly decreased the pH (*P* < .0001; GLM with adjusted pairwise contrasts), although *A. hylecoeti* cultures still showed the lowest pH compared to *W. bisporus* (*P* < .022) and *Y. scolyti* (*P* < .0001).

To determine the compound(s) released into the medium that was capable of decreasing culture pH so drastically, we inoculated both *I. typographus* yeasts as well as the six filamentous fungi mentioned above in PDB medium and screened the culture supernatant for major organic acids via LC–MS. A number of compounds were identified (see SI Results for details), but these were not present in sufficient concentration to cause the pH decrease observed. However, acetic acid, reported previously from *A. hylecoeti* (23) was indeed present in sufficient amounts. We quantified the presence of this acid in *A. hylecoeti*, *W. bisporus*, and *Y. scolyti* culture supernatant using NMR. Acetic acid was found to be the major acid in *A. hylecoeti* cultures (Fig. 4D, lower boxplot), but was detected in much lower amounts in both yeast cultures (*P* < .0001; GLM with adjusted pairwise contrasts; see also Suppl. Table S5 and Suppl. Fig. S18 for NMR results), even though the pH of all these cultures was below 4 (Fig. 4C). We confirmed the ability of acetic acid to decrease culture pH by adding increasing amounts of this acid to culture media while constantly measuring the pH (Suppl. Fig. S19). We then conducted a bioassay testing three different concentrations of acetic acid on seven fungi (Fig. 4E–F) and compared it to a control (0%). All six fungi were inhibited at 0.05% acetic acid (*P* < .014; GLM with adjusted pairwise contrasts), whereas the 0.125% treatment completely suppressed fungal growth for five of the species. Strikingly, the growth of *A. hylecoeti* was even promoted by acetic acid at 0.0025% (*P* < .0015) and did not show any reduction at the remaining concentrations tested. Finally, we confirmed that field nests of *E. dermestoides* also contained quantities of acetic acid sufficient to suppress the growth of competing fungi in our laboratory bioassays (Suppl. Table S5).

Taken together, our results show that *A. hylecoeti* strongly decreased the pH of its culture medium by releasing acetic acid. The low pH as well as acetic acid itself promoted the growth of *A. hylecoeti* and significantly inhibiting the growth of all potential competing fungi tested.

## Discussion

Wood-colonizing bark and ambrosia beetles commonly benefit from an association with microbes such as filamentous fungi [4] to meet some of the challenges of growth in woody tissue, such as its low content of nutrients and an abundance of competitors [29, 38]. Despite its poor nutritional quality, the wood of weakened or recently dead trees is still a highly sought, limited resource, mostly colonized by insects and wood-degrading fungi [44]. The slow-growing larvae of the ship-timber beetle *E. dermestoides* successfully drill their tunnels in such an environment, and *A. hylecoeti*, an associated yeast-like fungus forming a vegetative mycelium may help support their growth along with other fungal associates, including single-celled yeasts such as *Cyberlindnera* sp. [16, 18]. Here, we investigated the nutrient content of *A. hylecoeti* in comparison with two other *Alloascoidea* species, several yeasts (including *Cyberlindnera* strains), and various other beetle and non-beetle-associated fungi. Within a broad range of tested fungi, *A. hylecoeti* and the other *Alloascoidea* species measured were found to be among the richest in nutrients. We also studied whether *A. hylecoeti* has chemical mechanisms that could enable it to outcompete other fungi and thus promote the success of *E. dermestoides*. The accumulation of host tree phenolics and related compounds by *A. hylecoeti* in colonized woody medium had a negative effect towards competing fungi in laboratory bioassays. This fungus also produces metabolites, such as monoterpenes and acetic acid, that suppressed the growth of fungal competitors but enhanced the viability of *A. hylecoeti*. Sufficient acetic acid was released to lower the pH of the surrounding environment to pH 3.6.

Analyses of various classes of nutrients showed that the *E. dermestoides* symbiont, *A. hylecoeti*, was particularly rich in free amino acids, soluble sugars, ergosterol, as well as phosphorus and potassium compared to the other fungi tested, and also accumulated substantial amounts of fatty acids, B vitamins, and nitrogen. These substances may all be needed for insect growth, especially on plant-based diets, which are often notoriously low in these compounds [28]. Among the beetle-associated mutualistic fungi in our study, *A. hylecoeti* turned out to be the richest source of these nutrients, although the non-filamentous *Cyberlindnera* sp., also associated with *E. dermestoides*, was found to have a very high content of free amino acids, soluble sugars, and B vitamins as well. Previous research on ambrosia beetles has indicated that their fungal symbionts likely provide nutrients for insect development [4, 39, 41], but rarely have nutrients besides amino acids been analyzed or other fungi included for comparison and fungi are typically grown on rich medium rather than on a substrate relevant to the host tree. The high nutritional status of *A. hylecoeti* as well as of *Cyberlindnera* sp. could make an essential contribution to the development of *E. dermestoides*, where solitary larvae excavate large tunnels in a nutrient-poor substrate (sapwood) for more than two years [22]. Most other ambrosia beetle systems have shorter generation times with smaller and nonsolitary larvae that are cared for by adults, which actively farm mutualist fungi [4, 11]. In such situations, ambrosia beetle larvae might be able to consume more fungal tissue than when solitary because the adults are constantly renewing the growth substrate, removing pathogens, and even fertilizing their fungal gardens [3, 11].

Solitary larvae, such as those of *E. dermestoides*, consume less fungal tissue and so may benefit from having a concentrated source of nutrients provided by a fungal partner. Nevertheless, it is not possible to prove that the nutritional quality of *A. hylecoeti* provides a genuine benefit for *E. dermestoides* without direct bioassays on this insect. Such work is difficult at present due to the long duration of the larval stage and the fact that no artificial diet has been developed, but is still critical to understand the relationship between *E. dermestoides* and its symbiotic fungi. Besides its potential ecological role, phylogenetic considerations may also contribute to the high nutrient content of *A. hylecoeti* as the other *Alloascoidea* species analyzed also had high concentrations of most of the nutrients measured.

The ability of beetle-associated symbionts to detoxify host tree defense compounds is another major potential benefit of such mutualisms. For instance, in the Eurasian bark beetle *Ips typographus*, one of the major symbiotic fungi has been described to metabolize spruce phenolic defenses [61, 62], which may facilitate the colonization success of its beetle host. However, in this study, the symbiotic fungus *A. hylecoeti* does not detoxify the major phenolic compounds of the beech host, but rather accumulates many of them in large quantities compared to the other fungi studied without any apparent negative effects on its growth. On the other hand, the wood-degraders and myco-pathogenic fungi studied did not accumulate these beech wood phenolics studied and their growth was in fact inhibited by them. Moreover, the symbiont *A. hylecoeti* not only accumulated phenolics in the mycelium, but also in the medium immediately adjacent to mycelia, which might represent a mechanism to inhibit competing fungi. To our knowledge, such a defense strategy has not been described yet for any mutualist fungus whose insect host lives in dead wood.

The ability of *A. hylecoeti* to enrich the phenolic content of its mycelium increases with the availability of phenolics in the medium. These observations might be explained by the phenomenon of fungal biosorption, where some species are capable of absorbing phenolics and other organic compounds from their growth medium [63]. Given the high degree of accumulation of phenolic compounds in *A. hylecoeti* when cultivated on medium enriched with several phenolics, its cells might have practical value in wastewater treatment, an application being studied for multiple *Aspergillus* species [64, 65]. Nevertheless, the accumulation of phenolics by its symbiotic fungus imply that larvae of the ship-timber beetle also need to tolerate a diet rich in phenolic compounds, which might require special adaptations. Perhaps *E. dermestoides* larvae or its gut microbes metabolize these compounds [66] with beneficial effects [67, 68].

High competitive pressure within a limited resource such as the wood of freshly killed trees may select for fungi that outcompete other microbes by rapid growth or the release of antagonistic chemical compounds [32]. This behavior would also benefit any insect associates that gain advantages from these fungi. In our study with the ship-timber beetle symbiont *A. hylecoeti*, we found that multiple chemical compounds could be involved in inhibiting the growth of competitive fungi. These include phenolic compounds accumulated from the beech substrate and three de novo biosynthesized monoterpenes, linalool, terpineol, and citronellol. Although monoterpene-producing fungi are rare, the antimicrobial activity of these three terpene alcohols towards several fungi and bacteria has been well described in past studies [69–71]. Moreover, *A. hylecoeti* excreted substantial amounts of phenyllactic acid (see Suppl. Table S16), a well-known broad-spectrum antimicrobial compound [72], typically produced by lactic acid bacteria. Phenyllactic acid hindered the growth of all tested competitive fungi in this study except the pathogen *C. globosum* (see Suppl. Fig. S16). We identified high concentrations of phenyllactic acid in the culture supernatant of both *Ips typographus* yeasts as well, indicating that this metabolite may mediate competition within this bark beetle system as well.

We also demonstrated that acetic acid, previously reported from *A. hylecoeti* [23], is released in large amounts by this species, suppressing the growth of competing fungi. The growth of *A. hylecoeti* itself, in contrast, was enhanced by acetic acid and the resulting low pH conditions. The suppression of competitors using structurally simple compounds, such as acetic acid, has so far been reported only for a few ambrosia-associated fungi [55, 56, 73]. For example, ethanol was found to be a major mediator of competition against potential myco-pathogens, with only mutualist microbes remaining, similar to what we observed for acetic acid. As we also identified acetic acid as being present in larval tunnels in field nests, we assume that the fungus and insect are well adapted to its presence. In fact, *A. hylecoeti* not only was not inhibited, but was the only fungus tested to show enhanced growth at low pH. Fungi that decrease the pH of their substrate are well known [32, 74], particularly to facilitate the degradation of carbohydrate polymers in woody tissue [75–77], which might also be occurring within and around *E. dermestoides* nests.

We have compiled evidence largely from laboratory experiments that *A. hylecoeti*, a fungal associate of the wood-boring ambrosia beetle *E. dermestoides*, deploys a broad assortment of chemical compounds, including monoterpenes, phenyllactic acid, acetic acid and phenolics accumulated from the host tree tissues as inhibitors of competing microbes. However, additional studies need to be performed under natural conditions to determine if such chemically-mediated competition occurs on native substrates. In addition, the consequences of these compounds for the insect partner, *E. dermestoides*, still need to be explored and might be negative. These chemical compounds may also have other, non-symbiont-related functions in *A. hylecoeti*. For example, acetic acid released into the surroundings might help to break down cellulose and other carbohydrate polymers by decreasing the pH of the colonized substrate [78, 79]. Monoterpenes, like other volatile terpenes, might be involved in deterring the predation of arthropods besides *E. dermestoides* [80]. Nevertheless, the combination in *A. hylecoeti* of a high content of nutrients along with different chemical mediators of competition could make this species a good mutualist for ambrosia beetles like *E. dermestoides* that live in a nutrient-poor but highly competitive environment.

## Supplementary Material

suppl_wraf258

## Data Availability

All data generated or analyzed during this study are included in this published article (and its supplementary information files).
