# Supplementary material for: Fungal symbiont of an ambrosia beetle possesses high nutrient content and suppresses competing fungi with antimicrobial compounds
Source: ISME J. 2025 Nov 20;19(1):wraf258. doi: 10.1093/ismejo/wraf258 (PMC12684719; doi:10.1093/ismejo/wraf258)
Supplement: suppl_wraf258 [file suppl_wraf258.zip › SI Methods_Lehenberger et al.pdf]

Supplementary methods Lehenberger et al.

Fungal species, culture media, and collection of field nests

Field nests of the ambrosia beetles *Xyleborinus saxesenii* [N = 6] and *Elateroides dermestoides* [N = 7] were collected in beech forests around Tharandt, Germany during June-July 2022 and 2023. Here, several recently dead *Fagus sylvatica* logs with clear signs of beetle colonization were opened using a chainsaw and chisel. Samples of active field nests of *X. saxesenii* [~1 × 1 cm] as well as of larval tunnels of *E. dermestoides* [~1 × 0.5 cm] were removed [lumen of the tunnels and approx. 2 mm of surrounding woody tissue] and stored at -20°C until further processed for the nutritional analyses. For *E. dermestoides*, we collected six further nest samples for the quantification of acetic acid. Here, we sampled almost entire larval tunnels [lumen of tunnels and approx. 2 mm of surrounding woody tissue] yielding high fresh weights [see also Suppl. Table S5]. Control samples [uninfested wood] were taken from identical logs [separately for *E. dermestoides* and *X. saxesenii*] but at least 20 cm away from any signs of beetle infestation [2 × N = 6]. Samples were stored at -20°C.

Nutritional profiling

Differences in nutritional value were determined by comparing the sums of all measured amino acids, B vitamins, sugars, and fatty acids [classified as “total”] between individual fungi. Further, we compared the nutritionally important elements phosphorus, potassium, and nitrogen as well as the ergosterol content between fungi. To evaluate statistically significant differences among experimental groups, we fitted a series of generalized linear models [GLM] testing five model variants of three families to account for distributional and variance heterogeneity: Gaussian [identity link]; log-transformed Gaussian; sqrt-transformed Gaussian; Gamma [log link];

Gamma [inverse link]. Gamma models were generally avoided in case zero values were present in the individual datasets. Model selection was primarily based on the corrected Akaike Information Criterion [AICc], which accounts for small sample size, and was complemented by the Bayesian Information Criterion [BIC] to provide a robust balance between model fit and complexity. Simulation-based residual diagnostics (including a visual inspection of residual plots) were performed using the “DHARMa” package [1] including tests for quantile residual uniformity [Kolmogorov-Smirnov-test], overdispersion, and the presence of outliers. In addition, dispersion and heteroscedasticity were further evaluated using the “performance” package [2]. Furthermore, Cook´s distance was calculated to identify influential cases using the “stats” package [3]. All diagnostic criteria were jointly considered for model evaluation. The model with the lowest AICc value among those meeting the most acceptable residual diagnostics was selected for downstream inference. We then performed a type II likelihood ratio test [ANOVA] to test for overall group effects. Estimated marginal means [EMMs] and pairwise contrasts were computed using the “emmeans” package [4] with adjusted contrasts to account for multiple comparisons. Significance groups were assigned using compact letter displays via the “multcompView” and “multcomp” packages [5]. All individual amino acids, B vitamins, sugars, fatty acids, and elements per fungus [and field nest] were additionally visualized by heatmaps [and a barplot for relative abundance of fungal fatty acids] using the software R and the packages “ggplot2” [6] and “pheatmap” [7] without further statistical analyses. To compare the overall nutritional profiles of the examined fungi, we conducted principal component analyses [PCA] and permutational multivariate analyses of variance [PERMANOVA]. Here, data were normalized using a log1p transformation and further z-scaled to account for

differences in variance and measurement scales. The package “ggplot2” [6] was used for the final visualization of the PCA plot. For group comparisons, we performed pairwise PERMANOVAs based on Bray-Curtis dissimilarities using log1p-transformed but non-scaled data applying the “pairwiseAdonis” [8] and the “vegan” [9] package. To account for multiple testing, we applied the Benjamini-Hochberg [BH] correction. In general, we separated our analyses into the individual amino acids, B-vitamins, and sugars for the five tested yeasts and *A. hylecoeti* as well as into all filamentous fungi [including *A. hylecoeti*], where we compared all individual amino acids, B vitamins, sugars, fatty acids, and ergosterol. All plots were modified using the software Adobe Illustrator [version CS5]

Free amino acids

Methanol extracts were prepared from dried fungal biomass by homogenizing it with 1 mL of methanol [Honeywell] and three metal beads [Ø 3 mm, Askubal] on a paint shaker for 5min. Samples were centrifuged afterwards at 9400 rcf for 2 min and supernatant was collected for further analyses. Additionally, stored field nests [-20°C] were freeze-dried for 5 d and carefully cut into smaller pieces. Here, samples were homogenized for 10 min with metal beads in 1 mL of methanol using a paint shaker and centrifuged as described above.

Amino acids from fungal tissue and field nests were quantified with a targeted LC-MS/MS protocol using a C18-column [XDB-C18, 50 x 4.6 mm, 1.8 µm; Agilent Technologies, Santa Clara, CA, USA] after diluting the methanol extracts 1:10 [v:v] with water containing 10 µg/ml of a mixture of U-$^{15}N/^{13}C$ labeled amino acids [algal amino acid mix, Isotec, Miamisburg, OH, USA] and 5 uM of D5-tryptophan [Cambridge Isotope Laboratories, Inc.; Andover, MA]. For details on the chromatography and mass spectrometry [Agilent 1260 LC system [Agilent

Technologies, Santa Clara, CA, USA] coupled with a QTRAP 6500 tandem mass spectrometer [SCIEX, Darmstadt, Germany], see [10]. The mass spectrometer was operated in the positive ionization mode with multiple reaction monitoring [see Suppl. Table S2d for MRM table]. Water containing 0.05% formic acid and acetonitrile was employed as mobile phases A and B, respectively. All amino acids were quantified relative to the peak area of the corresponding labeled compound, except for asparagine [using U-$^{15}N/^{13}C$-aspartate and a response factor of 1.0].

B vitamins

From the methanol extract acquired during the analysis of amino acids, 50 μL was mixed with 50 μL of methanol containing 200 ng/mL D3-thiamine [B1], 200 ng/mL $^{13}C,^{15}N_2$-riboflavin [B2], 200 ng/mL D4-nicotinic acid [B3], 200 ng/mL D5-pyridoxine [B6], and 200 ng/mL $^{13}C_3,^{15}N$-pantothenic acid [B5] [all Toronto Research Chemicals, Toronto, Canada] as internal standards. B vitamin analysis was performed by LC-MS/MS on an Agilent 1260 series HPLC system [Agilent Technologies] coupled to a tandem mass spectrometer QTRAP 6500 [SCIEX, Darmstadt, Germany]. Chromatographic separation was achieved on a Zorbax Eclipse XDB-C18 column [50 x 4.6mm, 1.8μm, Agilent Technologies]. Water containing 0.05% formic acid and acetonitrile were employed as mobile phases A and B, respectively. The elution profile was: 0–3.0 min, 0% B; 3.0–6.0 min, 0–80% B; 6.0–6.01 min, 80–100% B; 6.01–7.0 min, 100% B; and 7.1–10.0 min, 0% B. Flow rate was kept at 1.1 mL/min, and the column temperature was maintained at 25°C. The mass spectrometer was equipped with a Turbo spray ion source operated in the positive ionization mode. The ion spray voltage was maintained at 5,500 eV. The turbo gas temperature was set at 620°C. Nebulizing gas was set at 60 psi, curtain gas at 40 psi, heating gas at

60 psi, and collision gas was set to “medium.” The mass spectrometer was operated in multiple reaction monitoring [MRM] mode [see Suppl. Table S2e for MRM table].

Soluble sugars

Soluble sugars were analyzed from the same methanolic extract [see amino acid analysis above] at 1:10 dilution in water containing 5 µg/mL $^{13}C_6$-glucose [Sigma-Aldrich], and 5 µg/mL $^{13}C_6$-fructose [Toronto Research Chemicals, Toronto, Canada], by LC-MS/MS as described in ref. [11]. Targeted analysis used an Agilent 1200 HPLC system coupled to an API 3200 tandem mass spectrometer [AB Sciex, Darmstadt, Germany]. The HPLC was equipped with a hydrophilic interaction liquid chromatography [HILIC] column [apHera-NH2 Polymer; Supelco, Bellefonte, PA, USA], and chromatographic separation was performed using water and acetonitrile as mobile phases A and B, respectively, with a flow rate of 1.0 ml min-1. The column temperature was maintained at 20°C. The mass spectrometer equipped with a turbo spray ion source was operated in the negative ionization mode. The ion spray voltage was maintained at −4,200 eV and the turbo gas temperature was set at 500°C. Nebulizing gas was set at 60 psi, curtain gas at 30 psi, heating gas at 60 psi, and collision gas at 4 psi. Multiple reaction monitoring was used to monitor analyte precursor ion to product ion [see Suppl. Table S2c for MRM table]. Data were acquired using the software Analyst 1.5.1 and quantification was performed using the software MultiQuant 3.0.3 [Sciex, Massachusetts, USA]. The concentrations of glucose and fructose were determined relative to the internal standards of $^{13}C_6$-glucose and $^{13}C_6$-fructose, respectively. The contents of sucrose, trehalose [both from Sigma-Aldrich] and mannitol [Fluka] were calculated based on external standard curves.

Fatty acids

Here, 1-40 mg dried fungal biomass were weighed and 350 mg Silibeads [Typ ZY 1.2 – 1.4 mm; Sigmund Lindner GmbH, Germany] were added for homogenization in a cell disruptor [Thermo Savant FastPrep FP120, Qbiogene Inc., USA] at 6.5 m/sec for 10 sec at 4°C. Extraction of fatty acids was performed as previously described [12] with slight modifications regarding ratios of solvents and concentration of sodium chloride. After addition of 1000 µl of chloroform:methanol 7:3 [v/v] and 40 µl of a mixture of chloroform:methanol [7:3, v:v] containing 271,3 µg tridecanoic acid [CAS 638-53-9; Sigma-Aldrich, Germany] as internal standard to powdered samples, samples were vortexed and extracted at room temperature for 40 min. 200 µL methanol and 200 µL 0.9 % [m/V] sodium chloride were added, vortexed and centrifuged at 2900 rcf for one minute. The aqueous phase was discarded and the washing process was repeated twice with 100 µL methanol and 100 µL 0.9 % [m/V] sodium chloride. After the chloroform phase was evaporated to dryness, it was reconstituted in 80 µL of trimethylsulfonium hydroxide:methyl tert-butyl ether 5:8 [v:v]. All solvents and additives for analysis of fatty acid methyl esters were obtained from Sigma-Aldrich [Germany], VWR [Belgium], Bernd Kraft [Germany] and Riedel-de-Haen [Germany].

Chromatographic separation of fatty acid methyl esters was achieved with a Trace 1300 GC system [Thermo Fisher Scientific, USA] equipped with a Supelco SP-2560 100m x 0.25 mm fused silica capillary column with 0.20 µm film thickness with poly-biscyanopropylsiloxane as stationary phase [Sigma Aldrich, Germany]. Injection volume was 1 µl splitless. The injector temperature was 250°C, and the initial column temperature was programmed at 120°C, then ramped at 4°C/min to 240°C and held for 15 min. Helium [5.3; Linde, Germany] was used as carrier gas at constant pressure of 500 kPa. After chromatographic separation, simultaneous

detection by a flame ionization detector module [Thermo Fisher Scientific, USA] and an ISQ series single quadrupole mass spectrometer [Thermo Fisher Scientific, USA] was enabled using a Dual Detector Microfluidics kit [Thermo Fisher Scientific, USA]. The temperature of the flame ionization detector was set at 250°C and hydrogen and air flow rates were set at 35 and 250 mL/min, respectively. The mass spectrometer was operated in the full scan mode between m/z 50 and 450 using a scan time of 0.2 min. Electron impact ionization occurred at 70 eV with an interface temperature of 250°C and source temperature of 250°C. Data were evaluated using XCalibur software [Version 4.2.47, Thermo Fisher Scientific, USA].

Retention times of fatty acid methyl esters were determined by analyzing a reference mixture of methyl palmitelaidate [CAS 10030-74-7; Larodan, Sweden], methyl vaccinate [CAS 6198-58-9; Larodan, Sweden], methyl cis-11-octadecenoate [CAS 1937-63-9; Larodan, Sweden], and TraceCERT® 37 component fatty acid methyl ester mix [Sigma-Aldrich, Germany]. Additionally, molecular ion peaks, base ion peaks, 2nd highest ion peak and further characteristic ion peaks resulting from typical fragmentation pattern of fatty acid methyl esters were determined in the mass spectrum of each fatty acid methyl ester in the reference mixture via full scan and their presence in the mass spectrum of each peak assigned to a fatty acid in the sample measurements was verified [see Suppl. Table S2f for details of analysis].

Calculation of relative and total concentration of fatty acids was performed using peak areas caused by fatty acid methyl esters resulting from flame ionization detection [13]. As correction factors for fatty acids detected in samples determined with the specific mass spectrometric system ranged between 0.94 and 1.02, no correction factors were included in the calculation.

Ergosterol

For the extraction of sterols, dried fungal biomass was transferred to 1.5 mL glass vials and gently crushed using pestles for microcentrifuge tubes [Reichelt, Germany]. The homogenized samples were supplemented with 1 mL hexane [Sigma Aldrich] and transferred to an ultrasonic bath for 5 minutes. Afterwards, samples were gently shaken overnight to allow all sterols to extract followed by centrifugation at 5600 rcf for 2 minutes. 100 μl of supernatant was then transferred to new glass vial with an insert, and 10 μl *N*-methyl-*N*-[trimethylsilyl] trifluoracetamide [MSTFA derivatization reagent, Macherey-Nagel, Germany] was added to each sample and incubated for 10 min at room temperature. Derivatized hexane extracts were stored at -20°C until further analysis.

A 6890 Series gas chromatograph [GC, Agilent Technologies] coupled to an Agilent 5973 quadrupole mass selective detector [interface temperature 270°C, quadrupole temperature 150°C, source temperature 230°C, electron energy 70 eV] was used to quantify sterols. An OPTIMA-5ms capillary column [30 m × 250 μm × 0.25 μm, Macherey-Nagel]] was used for sample separation, and He as carrier gas. 2 μl sample was injected in splitless mode at an initial oven temperature of 150°C. The temperature was held for 3 min, then increased to 250°C with a gradient of 40℃/min, and further increased to 300°C with a gradient of 5°C /min and then held for 5 min. Ergosterol was identified by comparing the retention time and mass spectrum to those of an authentic standard [Thermo Scientific, Germany], but it was quantified [based on extracted ion [EIC]] with external standard curves using a dilution series of ergosterol and normalized by sample dry weight.

Elemental composition

Element analyzer

To analyze nitrogen [N] and carbon [C] from fungal tissue, dried biomass was weighed into tin caps together with tungsten-[VI]-oxide [Elementar Analysensysteme GmbH, Germany] and pressed to form a pellet, which was analyzed using an elemental analyzer [Vario EL III, Elementar]. For every analysis run, a blank [containing 10 mg tungsten-[VI]-oxide], a daily calibration sample, and a control sample were additionally analyzed. The daily calibration sample, [4 mg acetanilide with 5 mg tungsten-[VI]-oxide] was later used as a factor to correct the measured values of the samples. Each pellet was individually combusted with an oxygen flow at 950 °C using helium as the carrier gas leading analytes into a column containing copper [see manual Elementar Analysensysteme GmbH [Elementar 2006] for detailed information].

ICP-OES analyses

For the analysis of aluminum [Al], calcium [Ca], copper [Cu], Iron [Fe], potassium [K], magnesium [Mg], manganese [Mn], sodium [Na], phosphorus [P], sulfur [S], and tin [Zn] inductively coupled plasma optical emission spectrometry [ICP-OES; iCAP pro x duo, Thermo Scientific] was applied following the methods described in ref.[14]. Here, dried fungal biomass was homogenized by nitric acid pressure digestion according to the methods described in ref. [15] with slight modifications due to the low amounts of fungal biomass by weighing biomass into pressure digestion containers with 3 mL of 65% nitric acid and 1 mL of 30% hydrogen peroxide and incubating them for 7 h at 170°C. An element stock solution was diluted 1:10 with 20% nitric acid [HNO_3] to reach the estimated concentrations of the samples. This diluted stock solution was used to prepare calibration standards with the dilutions 1:100, 1:50, 1:10, 1:5, 1:2 and a blank, which contained only 20% HNO_3. The daily control sample was prepared using 5 ml of the 1:10 diluted element stock solution

and 45 ml of 20 % HNO_3. This sample was made by mixing 5 mL of the undiluted element stock solution with 45 ml of 20 % HNO_3.

The origin and accumulation of phenolic compounds in *A. hylecoeti*

Accumulation in fungal biomass and culture medium

Chromatography was performed on an Agilent 1200 HPLC system [Agilent Technologies, Boeblingen, Germany]. Separation was achieved on an Agilent Zorbax Eclipse XDB-C18 column [50 x 4.6 mm, 1.8 µm; Agilent Technologies, Santa Clara, CA, USA]. Formic acid [0.05%] in water and acetonitrile was employed as mobile phases A and B, respectively. The elution profile was: 0.0-0.5 min, 5% B; 0.5-6.0 min, 5-37.4% B; 6.0-6.02 min, 37.4-80% B; 6.02-7.5 min, 80-100% B; 7.5-9.5 min, 100% B and 9.5-12 min 5% B. The mobile phase flow rate was 1.1 mL/min. The column temperature was maintained at 25°C.

The HPLC was coupled to an API 3200 tandem mass spectrometer [Applied Biosystems, Darmstadt, Germany] equipped with a Turbospray ion source operated in the negative ionization mode. The instrument parameters were optimized by infusion experiments with pure standards. The ion spray voltage was maintained at -4500 eV. The turbo gas temperature was set at 500°C. Nebulizing gas was set at 60 psi, curtain gas at 30 psi, heating gas at 60 psi and collision gas at 4 psi. The mass spectrometer was operated in multiple reaction monitoring [MRM] mode, and details of the instrument parameters can be found in Suppl. Table S2. Both Q1 and Q3 quadrupoles were maintained at unit resolution. Analyst 1.5 software [Applied Biosystems, Darmstadt, Germany] was used for data acquisition and processing. Compounds were quantified by external standard curves using dilution series of commercial standards [see Suppl. Table S2 for suppliers] and normalized by the individual sample dry weight.

We compared individual phenolics in fungal biomass from *A. hylecoeti* with the other fungal species to determine the possible accumulation of such compounds. Data of the most abundant phenolics was first visualized by heatmaps and then further processed for statistical analyses as described for the nutritional profiling using fitted GLMs. An overview of all identified phenolics in fungal biomass was visualized by a heatmap as well without further statistical analyses. To examine the abundance over time for the most dominant compounds identified in culture medium of *A. hylecoeti*, data was visualized by line plots using the R package “ggplot” [6] and further analyzed using fitted GLMs. Additionally, we plotted the abundance in culture medium over time for the most dominant compounds for all five fungi and visualized the findings by line plots. No statistical analyses were performed for incubation times 10-28 d as most compounds were only present in very low concentrations for the remaining four fungi [in contrast to *A. hylecoeti*]. However, we used parts of the dataset [Incubation time: 7 d] to compare the presence of phenolics in the previously colonized culture medium of the five tested fungi, prepared a heatmap [see above], and performed fitted GLMs for individual compounds as described previously. All plots were modified using the software Adobe Illustrator [version CS5].

$^{13}C_6$-labeled Glucose experiment to clarify the origin of phenolics

To determine if the major phenolics in *A. hylecoeti* culture medium are produced by fungi, we supplemented beech sawdust medium with 0.8% of $^{13}C_6$-labeled glucose [Deutero, Germany] and with 0.8% of unlabeled glucose [Roth, Germany] serving as the control. Afterwards, we inoculated a fresh culture of *A. hylecoeti* [N = 6] on cellophane using standard petri dishes at 25°C and 65% humidity. Biomass and medium samples were harvested after 16 d and subsequently freeze-dried. Methanol extracts were prepared as described above. Incorporation of ^{13}C into phenolics was

analyzed by ultra-high-performance liquid chromatography–electrospray ionization–high resolution mass spectrometry [UHPLC–ESI–HRMS] with a Dionex Ultimate 3000 series UHPLC [Thermo Scientific] and a Bruker timsToF mass spectrometer [Bruker Daltonik, Bremen, Germany] as described in ref. [16]. UHPLC was used applying a reversed-phase Zorbax Eclipse XDB-C18 column [100 mm × 2.1 mm, 1.8 µm, Agilent Technologies, Waldbronn, Germany] with a solvent system of 0.1% formic acid [A] and acetonitrile [B] at a flow rate of 0.3 ml/min. The elution profile was the following: 0 to 0.5 min, 5% B; 0.5 to 11.0 min, 5% to 60% B in A; 11.0 to 11.1 min, 60% to 100% B, 11.1 to 12.0 min, 100% B and 12.1 to 15.0 min, 5% B. Electrospray ionization [ESI] in negative ionization mode was used for the coupling of LC to MS. The mass spectrometer parameters were set as follows: capillary voltage -3.5 KV, end plate offset 500V, nebulizer pressure 2.8 bar, nitrogen at 280°C at a flow rate of 8 L/min as drying gas. Acquisition was achieved at 12 Hz with a mass range from m/z 50 to 1500. At the beginning of each chromatographic analysis, 10 µL of a sodium formate-isopropanol solution [10 mM solution of NaOH in 50/50 [v/v] isopropanol water containing 0.2% formic acid] was injected into the dead volume of the sample injection for re-calibration of the mass spectrometer using the expected cluster ion m/z values.

Integration of peak area of selected ion traces was carried out using Quant analysis software [Bruker Daltonik, Bremen, Germany]. Ion traces for the molecular ion [M-H]- for the non-labelled analyte and the traces with one or two ^{13}C-atoms incorporated were isolated with a width of m/z 0.005. The m/z listed in Suppl. Table S2b were used. The sum of the peak areas [total] of non-labelled and one- and two-times-labelled ions was calculated and each isotopologue expressed as a percentage of the total.

Catabolism of phenolics from artificially enriched media

The phenolic mixture was prepared from individual stock solutions and finally added to PDA after autoclaving. Fungi were inoculated until petri dishes were completely covered or up to a max. of 15 d for fungi that were inhibited by the mixture. Controls [PDA with phenolic mixture, but without fungus] were incubated for 15 d. Then, biomass and medium samples were taken and subsequently freeze-dried. Methanol extraction as well as analyses for phenolic compounds were performed as described above. Here, we compared the amount of the respective phenolic compound from control plates with plates covered with the individual fungi. If a compound was present in much lower proportions in a plate previously covered from fungus, this indicated possible catabolism. Data were first visualized by boxplots and then analyzed for statistical differences using fitted GLMs as described above for the nutrient analyses. All plots were modified using the software Adobe Illustrator [version CS5].

Insights into the competitiveness of *A. hylecoeti* using various bioassays

Supernatant bioassay

A plug of pure fungal mycelium [Ø 6 mm] from each fungus was inoculated on cellophane on the prepared medium as well as on a control [PDA with 2% sterile-filtered PDB+ 2% beech sawdust without fungus]. All plates [N = 6-7 per fungus] were incubated at 25°C and 65% humidity and checked daily until control dishes were completely covered. Afterwards, all fungal biomass was removed and freeze-dried for 4 d to record dry weight.

The dry weights of controls and the 2% *A. hylecoeti* extract treatment were compared separately for each fungus using Welch´s two-sample t-test [see also Suppl. Table S6] using the software R [version 4.2.1.]. Boxplots were made using the

R package “ggplot2” [6] and were modified using the software Adobe Illustrator [version CS5].

Bioassays with multiple compounds associated with *A. hylecoeti*

Each compound was tested separately and compared to a control [PDA with 0.25% DMSO]. However, all plates were prepared within two days and inoculated at the same day with fungus to allow a comparison between each treatment and control. A plug of pure fungal mycelium [Ø 6 mm] from each fungus was inoculated on cellophane on the prepared media and all plates [N = 6-7 per fungus] were incubated at 25°C and 65% humidity and checked daily. Once the dishes from one treatment were completely covered, the experiment was stopped on the same day for all remaining treatments for the same fungus species. Fungal biomass was removed and freeze-dried for 4 d to record dry weights.

For statistical analyses, we applied fitted GLMs as described for the nutrient data. Here, we specifically focused on pairwise contrasts comparing fungal growth [mg] on the control with each of the eleven tested compounds. P-values were adjusted for multiple testing using Dunnett´s correction. Boxplots were made using the R package “ggplot2” [6] and were modified using the software Adobe Illustrator [version CS5].

Bioassays using a phenolic blend

A plug of pure fungal mycelium [Ø 6 mm] from each fungus was inoculated on cellophane on the prepared media and all plates [N = 5-6 per fungus] were incubated at 25°C and 65% humidity and checked daily until the dishes for the control of each individual fungus were completely covered. Fungal biomass was removed and freeze-dried for 4 d to record dry weight.

We used Welch´s two-sample t-test [see also Suppl. Table S6] and the software R [version 4.2.1.] to identify significant differences between fungal growth on the control and the phenolic blend treatment separately for each fungus. Boxplots were made using the R package “ggplot2” [6] and were modified using the software Adobe Illustrator [version CS5].

The impact of *A. hylecoeti* on pH

pH measurements of fungi in liquid cultures

To exclude any contaminations, we transferred biomass as well as medium from each individual liquid culture to PDA and morphologically confirmed the sole presence of the respective fungus. After 12 d, each liquid culture was filtered using a coffee filter and supernatant was collected. Subsequently, the pH was measured using a pH meter [WTW pH526]. Additionally, we repeated this experiment with two yeasts [*Wickerhamomyces bisporus*, *Yamadazyma scolyti*, N = 6] in 3% PDB media without beech sawdust to test if changes in pH are generally associated with species of the Saccharomycotina. Both yeasts were included for pH measurements as well as for the identification of excreted organic acids in culture supernatant [see below]. To allow direct comparison, we further included *A. hylecoeti* as described above. Here, 100 µl of a prepared liquid yeast pre-culture in 3% PDB with an OD of 0.1 was used as the inoculum for each yeast replicate. Otherwise, the procedure and conditions were the same as described above.

We used fitted GLMs as previously described for the nutrient analyses to identify significant differences between the pH of fungi and control medium and plotted data using the software R [version 4.2.1.]. Boxplots were made using the R package “ggplot2” [6] and were modified using the software Adobe Illustrator [version CS5].

The influence of pH on fungal growth

One plug [Ø 6 mm] of pure fungal mycelium was transferred to 50 mL of 3% PDB medium in a 100 mL glass flask and incubated on a shaker for 6 d at 25°C and 170 rcf. Before, the pH was adjusted using either 0.3M HCL or 0.3M NaOH. After 6 d, each liquid culture was filtered using a coffee filter and biomass was collected and freeze-dried for 4 d to record dry weights.

We applied a Welch two-sample T-Test [see also Suppl. Table S6] using the software R [version 4.2.1.] to identify a significant impact of pH on fungal growth. Boxplots were made using the R package “ggplot2” [6] and were modified using the software Adobe Illustrator [version CS5].

Identification of major organic acids

Experiments were initiated by transferring one plug [Ø 6 mm] of pure fungal mycelium to 150 mL of medium in a 250 mL glass flask followed by incubation for 9 d on a shaker at 25°C and 170 rcf. An aliquot [1 mL] of colonized liquid medium was taken and centrifuged at 9400 rcf to separate biomass and medium. 500 µl of supernatant were removed and mixed with 500 µl of methanol by vortexing for 20 s followed by inoculation at RT for 10 min. Samples were centrifuged again as described above and collected extracts were stored at -20°C until further processed. Additionally, we examined the major organic acids in liquid cultures inoculated with two yeasts [*Wickerhamomyces bisporus*, *Yamadazyma scolyti*; N = 6 per yeast]. To allow direct comparison, we further included *A. hylecoeti* as described above. Here, 100 µl of a prepared liquid yeast pre-culture in 3% PDB with an OD of 0.1 was used as the inoculum for each yeast replicate. We used a 3% PDB medium without the addition of beech sawdust. Otherwise, the procedure and conditions were the same as described above.

Identification and quantification of organic acids was performed using the same chromatography and LC-MS/MS conditions as described above for phenolic compounds. Details of the instrument parameters can be found in Suppl. Table S2. Compounds were quantified by external standard curves using dilution series of commercial standards [see Suppl. Table S2a for suppliers] and normalized by the individual sample dry weight.

Here, we compared individual organic acids in liquid culture medium of six filamentous fungi as well as of two yeasts and the yeast-like fungus *A. hylecoeti*. Data were first visualized by boxplots and further analyzed using fitted GLMs. All plots were modified using the software Adobe Illustrator [version CS5].

Bioassay using succinic acid

A possible inhibitory effect of succinic acid was analyzed by supplementing PDA with succinic acid [80 µg/mL; see also Suppl. Table S4], which was dissolved first in DMSO. Here, we tested five fungi [*A. hylecoeti* = Ambrosia beetle fungus; *P. ostreatus*, *P. nameko, A. aegerita* and *C. globosum* = Wood-degrading, competing fungi; see also Suppl. Table S1]. A plug of pure fungal mycelium [Ø 6mm] from each fungus was inoculated on cellophane on the prepared media and all plates [N = 6 per fungus] were incubated at 25°C and 65% humidity and checked daily until the dishes for either the control [PDA + 0.25% DMSO] or the succinic acid treatment of each individual fungus were completely covered. Fungal biomass was removed and freeze-dried for 4 d to record dry weights.

Here, we used Welch´s two-sample t-test and the software R [version 4.2.1.] to compare fungal growth in mg of dry biomass between control and succinic acid treatment. Boxplots were made using the R package “ggplot2” [6] and were modified using the software Adobe Illustrator [version CS5].

Identification and quantification of acetic acid by NMR

NMR samples with water extracts were measured in 5 mm OD tubes into which a 2 mm OD flame-sealed capillary containing benzene-d_6 as an external standard was inserted. NMR spectra were referenced either to the residual solvent peaks of benzene-d_6 at δ_H7.16 and δ_C128.39, respectively, or, for the quantitative determination of acetic acid in methanolic nest extracts, to the water signal at δ_H4.90. A Bruker Avance III HD spectrometer equipped with a 5 mm TCI cryoprobe and cryoplatform was used. Data acquisition and processing was performed using Bruker TopSpin ver. 3.6.1. The ERETIC II quantification tool implemented in TopSpin was used to process the quantitative NMR data. The spectral parameters for the NMR experiments were as follows: pulse program *zg30*, spectral width 4 ppm, transmitter frequency offset 1 ppm, fid size 64K, relaxation delay 5 sec, total acquisition time 16.4 s, scans accumulated for each sample 32. For processing, spectra were calculated using 32K data points with a line broadening of 0.3 Hz. Signal integration was performed after baseline correction. In addition to the characteristic odor, the identity of acetic acid was confirmed by HMBC [see Suppl. Figure S16]. The methyl function of acetic acid dissolved in methanol showed resonances at δ_H1.91 and δ_C20.9, respectively, and the carboxy function showed a resonance at δ_C175.0.

Significant differences in acetic acid concentration from fungal liquid cultures were determined using fitted GLMs as described for the nutrient analyses. The plot was modified using the software Adobe Illustrator [version CS5]. We did not perform any statistical analyses of the field nest data but included these samples to determine if acetic acid is also present in biologically relevant concentrations under natural conditions.

Bioassay using acetic acid

The effect of acetic acid on fungal growth was analyzed using fitted GLMs as described for the nutrient data. Here, we specifically focused on pairwise contrasts comparing fungal growth [mg] on the control with each of the three acetic acid concentrations. P-values were adjusted for multiple testing using Dunnett´s correction [see also Suppl. Table S6]. Data were visualized by line plots and the R package “ggplot” [6] and plots were modified using the software Adobe Illustrator [version CS5].

The effect of acetic acid on pH of culture medium

To clarify, how the addition of acetic acid to culture medium influences the pH, we transferred increasing amounts of the pure compound to 400 mL of autoclaved liquid 3% PDB medium [N = 5]. Here, we first measured the pH of the PDB medium without any addition. Then, we added increasing amounts of acetic acid [200, 300, 500, 700, 1000, 1900, and 3500 µl] to the individual five replicates / bottles. We slowly added acetic acid to the medium under constant stirring while the pH was measured continually using a pH meter [WTW pH526].

Data were visualized with a boxplot using the software R [version 4.2.1.] and the R package “ggplot” [6] and finally modified using the software Adobe Illustrator [version CS5]. We did not perform any statistical analyses for this experiment.

References

1. Hartig F. DHARMa: residual diagnostics for hierarchical [multi-level/mixed] regression models. CRAN: Contributed Packages. 2016.
2. Lüdecke D, Ben-Shachar MS, Patil I, Waggoner P, and Makowski D. performance: An R package for assessment, comparison and testing of statistical models. *J op sou soft*. 2021; **6**(60). 3139.
3. Team RC. R: A language and environment for statistical computing. R Foundation for Statistical Computing, Vienna, Austria. http://www. R-project. org/. 2016.
4. Lenth R. emmeans: Estimated Marginal Means, aka Least-Squares Means. R package version 1.8. 5. 2023.

5. Hothorn T, Bretz F, and Westfall P. Simultaneous inference in general parametric models. *Biometrical Journal: J Math Meth Biosc*. 2008; **50(**3): 346-363.
6. Wickham H, ggplot2: Elegant Graphics for Data Analysis. 2016. Springer-Verlag New York.
7. Kolde R, pheatmap: Pretty Heatmaps. R package version 1.0. 12. 2019.
8. Martinez Arbizu P. pairwiseAdonis: Pairwise multilevel comparison using adonis. R package. Version 0.4. Available on URL: https://github. com/pmartinezarbizu/pairwiseAdonis. 2020.
9. Oksanen J, Kindt R, and Simpson GL. vegan3d: static and dynamic 3D plots for the 'vegan'package. R package version. 2017. **1**.
10. Crocoll C, Mirza N, Reichelt M, Gershenzon J, and Halkier BA. Optimization of engineered production of the glucoraphanin precursor dihomomethionine in *Nicotiana benthamiana*. *Front Bioeng Biotech*. 2016. **4**: 14.
11. Madsen SR, Kunert G, Reichelt M, Gershenzon J, and Halkier BA. Feeding on leaves of the glucosinolate transporter mutant gtr1gtr2 reduces fitness of Myzus persicae. *J Chem Ecol*. 2015. **41**: p. 975-984.
12. Folch J, Lees M, and Stanley GHS. A simple method for the isolation and purification of total lipides from animal tissues. *J biol chemi*. 1957. **226**(1): 497-509.
13. 12966-4:: ISO. Animal and vegetable fats and oils - Gas chromatography of fatty acid methyl esters - Part 4: Determination by capillary gas chromatography.
14. Feeding stuffs A. DIN EN 15621:2017-10. Methods of sampling and analysis - Determination of calcium, sodium, phosphorus, magnesium, potassium, sulphur, iron, zinc, copper, manganese and cobalt after pressure digestion by ICP-OES. dx.doi.org/10.31030/2666147.
15. Method handbook, VDLUFA MB VII. , Mikrowellenbeheitzter Druckaufschluss, Cap. 2.1.3, 2021, VDLUFA. 2021.
16. Müller AT, Reichelt M, Cosio EG, Salinas N, Nina A, Wang D, Moossen H, Geilmann H, Gershenzon J, Köllner TG, and Mithöfer A. Combined –omics framework reveals how ant symbionts benefit the Neotropical ant-plant *Tococa quadrialata* at different levels. *iScience*. 2022; **25**(10).105261.

Supplementary Figures

Suppl. Fig. S1: Heatmaps showing the average quantity of individual free amino acids, soluble sugars, and B vitamins (N = 7-10) for the seven investigated yeasts and the three *Alloascoidea* species in comparison to the control medium. **a** = Free amino acids in nmol/mg of dry biomass, **b** = Soluble sugars in µg/mg of dry biomass, and **c** = B vitamins in µg/g of dry biomass. Fungi were inoculated on 5% beech sawdust medium in large petri dishes until the petri dish was completely covered (in

case of the *Alloascoidea* species) or until a sufficient amount of biomass was available for the single-celled yeasts. *A. hylecoeti* is highlighted in green.

Suppl. Fig. S2: The nutritional content of fungal biomass was analyzed from cultures inoculated on 5% beech sawdust medium in large petri dishes until the petri dishes were completely covered. The 13 individual fungi were classified into four different groups (nematophagous fungus, mutualists, wood degraders, and potential myco-pathogens) depending on their ecological niche, whereas the fifth group is a pure beech sawdust control medium without any fungus. Bold letters above each boxplot indicate significant differences between all 13 fungi (see Suppl. Table S6 for individual *P* values; GLM with adjusted pairwise contrasts). *A. hylecoeti* is highlighted in green. **a** = Boxplots showing the total free amino acid content in nmol/mg of dry biomass (N = 6) based on the sum of 19 analyzed amino acids (see Suppl. Fig. S2). **b** = Boxplots showing the total soluble sugar content in µg/mg of dry biomass (N = 6) based on the sum of four analyzed sugars plus one sugar alcohol (see Suppl. Fig. S3). **c** = Boxplots showing the total free B vitamin content in µg/g of dry biomass (N = 6) based on the sum of five analyzed B vitamins (see Suppl. Fig. S4). **d** = Boxplots showing the total potassium content in mg/g of dry biomass (N = 6 -12). **e** = Boxplots showing the total phosphorus content in mg/g of dry biomass (N = 6 - 12). **f** = Boxplots showing the total nitrogen content in percentage (%) of dry biomass (N = 6 - 7).

Suppl. Fig. S3: The fatty acid and ergosterol content of fungal biomass was analyzed from cultures inoculated on 5% beech sawdust medium in large petri dishes until the petri dish was completely covered. The individual 13 fungi were classified into four different groups (nematophagous fungus, mutualists, wood degraders, potential myco-pathogens) depending on their ecological niche, whereas

the fifth group indicates a pure beech sawdust medium control without any fungus. Bold letters above each boxplot indicate significant differences between all 13 fungi (see Suppl. Table S6 for individual *P*values; GLM with adjusted pairwise contrasts). *A. hylecoeti* is highlighted in green **a** = Boxplots showing the total free fatty acid content in µg/mg of dry biomass (N = 6-7) based on the SUM of 19 analyzed fatty acids (see Suppl. Fig. S6). **b** = Boxplots showing the free ergosterol content in mg/g of dry biomass (N = 6).

Suppl. Fig. S4: Heatmap showing the average quantity for each of the 19 analyzed free amino acids per fungus (N = 6) in nmol/mg of dry biomass plus the total amount (Total). Fungi were classified into four different groups (nematophagous fungus, mutualists, wood degraders, potential myco-pathogens) depending on their ecological niche, whereas the fifth group indicates a pure beech sawdust medium control without any fungus. Fungi were inoculated on 5% beech sawdust medium in large petri dishes until the petri dish was completely covered. *A. hylecoeti* is highlighted in green.

Suppl. Fig. S5: Heatmap showing the average quantity for each of the four analyzed soluble sugars as well as the sugar alcohol mannitol per fungus (N = 6) in µg/mg of dry biomass plus the total amount (Total). Fungi were classified into four different groups (nematophagous fungus, mutualists, wood degraders, potential myco-pathogens) depending on their ecological niche, whereas the fifth group indicates a pure beech sawdust medium control without any fungus. Fungi were inoculated on 5% beech sawdust medium in large petri dishes until the petri dish was completely covered. *A. hylecoeti* is highlighted in green.

Suppl. Fig. S6: Heatmap showing the average quantity for each of the five analyzed free B vitamins per fungus (N = 6) in µg/g of dry biomass plus the total amount (Total). Fungi were classified into four different groups (nematophagous fungus, mutualists, wood degraders, potential myco-pathogens) depending on their ecological niche, whereas the fifth group indicates a pure beech sawdust medium control without any fungus. Fungi were inoculated on 5% beech sawdust medium in large petri dishes until the petri dish was completely covered. *A. hylecoeti* is highlighted in green.

Suppl. Fig. S7: Heatmap showing the average quantity for each of the 19 identified free fatty acids per fungus (N = 6-7) in µg/mg of dry biomass plus the total amount (Total). Fungi were classified into four different groups (nematophagous fungus, mutualists, wood degraders, potential myco-pathogens) depending on their ecological niche, whereas the fifth group indicates a pure beech sawdust medium control without any fungus. Fungi were inoculated on 5% beech sawdust medium in large petri dishes until the petri dish was completely covered. *A. hylecoeti* is highlighted in green.

Suppl. Fig. S8: Barplots showing the relative abundances in percentage (%) per fungus and beech sawdust control for the 19 identified free fatty acids (N = 6-7). Fungi were inoculated on 5% beech sawdust medium in large petri dishes until the petri dish was completely covered. *A. hylecoeti* is highlighted in green.

Suppl. Fig. S9: Heatmaps showing the average value of free sugars in µg/mg (**a**), free B vitamins in µg/g (**b**), and free amino acids in nmol/mg (**c**) identified from field nests of the ambrosia beetles *Xyleborinus saxesenii* and *Elateroides dermestoides* in comparison to un-colonized beech wood (Control). Field nests were opened using a

chainsaw and an axe, and nest samples were removed and dried for five days (freeze dryer) until further processed.

Suppl. Fig. S10: Heatmap showing the average quantity of 12 analyzed elements in µg/g of dried biomass for 13 fungi, which were classified into four different groups (nematophagous fungus, mutualists, wood degraders, potential myco-pathogens) depending on their ecological niche, whereas the fifth group indicates a pure beech sawdust medium control without any fungus. Fungi were inoculated on 5% beech sawdust medium in large petri dishes until the petri dish was completely covered (N = 6-12). *A. hylecoeti* is highlighted in green.

Suppl. Fig. S11: Heatmap showing the average quantity of ten individual phenolic compounds and related compounds in µg/g identified from dry biomass from 13 individual fungi, which were classified into four different groups (nematophagous fungus, mutualists, wood degraders, potential myco-pathogens) depending on their ecological niche, whereas the fifth group indicates a pure beech sawdust medium control without any fungus. Fungi were inoculated on 5% beech sawdust medium in large petri dishes until the petri dish was completely covered (N = 6). *A. hylecoeti* is highlighted in green.

Suppl. Fig. S12: Line plots showing the abundance of individual phenolic compounds in µg/g over seven different time points (7 – 28 d, N = 6) in 5% beech sawdust medium in standard petri dishes colonized by *A. hylecoeti* in comparison to blank beech sawdust medium (control). After each individual time point, all fungal biomass growing on cellophane was removed and medium samples were analyzed. The control was sampled together with the 28 d treatment. Bold letters above each

time point indicate a significant difference between the individual treatments (GLM with adjusted pairwise contrasts, Error bars = SD).

Suppl. Fig.S13: Line plots comparing the abundance of phenolic compounds in culture medium in µg/g over seven different time points (7 – 28 d, N = 6) between *A. hylecoeti* (red line) and four representative fungi (*R. sulphurea*, *E. polonica* as ambrosia and bark beetle fungi; *P. ulmarius*, *C. globosum* as competing fungi). Fungi were inoculated on 5% beech sawdust medium in standard petri dishes, whereas the control was blank beech sawdust medium. The day 7 dataset was additionally visualized through a heatmap (see Fig. 2a, right heatmap). Error bars are showing the SD. Here, seven phenolic compounds are shown: Vanillic acid, Protocatechuic acid, Gallic acid, Ferulic acid, Catechin, Caffeic acid, and Syringic acid.

Suppl. Fig. S14: Boxplots showing the concentration of individual phenolic compounds and related compounds identified in µg/g in dry culture medium (**a**) after fungal colonization and in dry fungal biomass (**b**) from five different fungal species. Fungi were inoculated on cellophane using PDA as culture medium (N = 6), which was supplemented with a blend of eight compounds (see Suppl. Table S4 for added concentrations: Treatment high conc.). In general, fungi were inoculated until petri dishes were completely covered or up to a max. of 15 d (for fungi, which were inhibited from blend). Biomass and medium (covered from fungus) were analyzed separately for remaining compounds applied. Bold letters above each boxplot indicate significant differences between fungi and control (PDA with blend but without fungus) (see Suppl. Table S6 for *P* values; GLM with adjusted pairwise contrasts). Here, six phenolic compounds and related compounds are shown: Caffeic acid, Ferulic acid, Protocatechuic acid, Quinic acid, Catechin, and Vanillic acid. *A. hylecoeti* is highlighted in green.

Suppl. Fig. S15: The potential bioactivity of *A. hylecoeti* was tested against five competing fungi and itself by performing bioassays testing individual compounds (N = 6-7). All individual compounds were dissolved in DMSO before adding them to PDA medium. Control medium was supplemented with DMSO at the same concentration as used for the bioassays. Here (**a-b**), monoterpenes are highlighted in blue, phenyllactic acid in red, and phenolic compounds in orange. The four compounds marked with an orange symbol are produced by *A. hylecoeti*, whereas the remaining compounds have been found to be accumulated in medium when *A. hylecoeti* is present (see Fig. 2). All tested concentrations were provided in Suppl. Table S4. A fitted GLM model and pairwise contrasts including Dunnett´s correction was used to identify significances. Asterisks above boxplots indicate a significant difference between the tested compound and a control (ns - $P > 0.05$, * - $P < 0.05$, ** - $P < 0.01$, *** - $P < 0.001$; see Suppl. Table S6 for individual *P* values). **a** = Boxplots visualizing the effect of individually tested compounds on *A. aegerita*. **b** = Boxplots visualizing the effect of individually tested compounds on *P. ulmarius*.

Suppl. Fig. S16: Identification of major organic acids from culture supernatants of filamentous fungi (**a-c**) and two *Ips typographus* yeasts (**d-f**) (N = 6). A GLM with adjusted pairwise contrasts (**a-f**) was applied to identify significant differences. Bold letters indicate a significant difference between fungi (see Suppl. Table S6 for individual P values). **a** = Boxplots showing the amount of isopropylmalic acid in µg/L in culture supernatant (0.5% PDB + 2% beech sawdust) after an incubation time of 9 d. **b** = Boxplots showing the amount of phenyllactic acid in µg/L in culture supernatant (0.5% PDB + 2% beech sawdust) after an incubation time of 9 d. **c** = Boxplots showing the amount of succinic acid in mg/L in culture supernatant (0.5% PDB + 2% beech sawdust) after an incubation time of 9 d. **d** = Boxplots showing the

amount of phenyllactic acid in mg/L in culture supernatant (3% PDB) after an incubation time of 9 d for the two *Ips typographus* yeasts *Y. scolyti* and *W. bisporus* in comparison to *A. hylecoeti*. **e** = Boxplots showing the amount of kynurenic acid in µg/L in culture supernatant (3% PDB) after an incubation time of 9 d for the two *Ips typographus* yeasts *Y. scolyti* and *W. bisporus* in comparison to *A. hylecoeti*. **f** = Boxplots showing the amount of isopropylmalic acid in µg/L in culture supernatant (3% PDB) after an incubation time of 9 d for the two *Ips typographus* yeasts *Y. scolyti* and *W. bisporus* in comparison to *A. hylecoeti*. **g** = The effect of pH on the growth of two fungi in mg of dry biomass was investigated by inoculating fungi in liquid 3% PDB cultures (N = 6, Incubation time = 6 d) with a low pH of 3.5 (simulating pH identified for *A. hylecoeti*) and a moderate pH of 5.0. Differences in growth was revealed using Welch´s Two-Sample t-test for each fungus. Asterisks indicate significant differences in growth between treatments (ns - $P > 0.05$, * - $P < 0.05$, ** - $P < 0.01$, *** - $P < 0.001$; see Suppl. Table S6 for individual *P* values). *A. hylecoeti* is highlighted in green.

Suppl. Fig. S17: Boxplots comparing the growth of five fungi in mg of dry biomass when inoculated on PDA medium (N = 6) enriched with succinic acid (Conc.: 80 µg/mL). *A. hylecoeti* is highlighted in green.

Suppl. Fig. S18: a) HMBC-spectra of acetic acid from *E. dermestoides* nest extracts in comparison to an uncolonized wood control (red 1 – 6 = Individual nest samples, blue = Wood control). **b**) ^{1}H-^{13}C HMBC of nest extract no.5 (see a).

Suppl. Fig. S19: Boxplots visualizing the decrease of pH of 400 mL PDB with increased amounts (in µl) of added acetic acid (N = 5).

Supplementary Tables

Suppl. Table S1: Overview of the 25 fungal isolates used in this study and additional information on their phylogenetic order, classification, and source.

Suppl. Table S2: a) Details of the analysis of phenolic compounds and organic acids by targeted LC-MS/MS (HPLC 1200 (Agilent Technologies)-API3200 (Applied Biosystems)) in negative ionization mode. Listed are the settings for multiple reaction monitoring of each compound, the LC retention time and the supplier of the commercial standards. **b**) Details for the quantification of 13C incorporation into phenolic compounds by LC-ESI-Q-ToF-MS (timsTOF mass spectrometer (Bruker Daltonik)) in negative ionization mode. **c**) Details of the analysis of sugars by LC-MS/MS (HPLC 1200 (Agilent Technologies)-API3200 (Applied Biosystems)) in negative ionization mode. **d**) Details of the analysis of amino acids by LC-MS/MS (HPLC 1260 (Agilent Technologies)-QTRAP6500 (Sciex)) in positive ionisation mode. **e**) Details of analysis of B vitamins by LC-MS/MS (HPLC 1260 (Agilent Technologies)-QTRAP6500 (SCIEX)) in positive ionization mode. **f**) Details of analysis of fatty acids by a 1300 Trace GC system with a simultaneous detection by FID and MS.

Suppl. Table S3: $^{13}C_6$ labeled glucose feeding experiment indicating that the compounds catechin (sheet 1), gallic acid (sheet 2), quinic acid (sheet 3), protocatechuic acid (sheet 4), and ferulic acid (sheet 5) are not produced by fungi. The sum of the peak areas (total) of the non-labelled molecular ion and molecular ions with one or two ^{13}C atoms was calculated and each isotopologue expressed as a percentage of the total (in yellow). The isotopologues are compared between *A. hylecoeti* (N = 7) inoculated on either 0.8% unlabeled glucose (Control) or ^{13}C-labeled glucose in water-agar supplemented with 2% beech sawdust.

Suppl. Table S4: Individual compounds and tested concentrations in µg/mL used for bioassays as well as for the bioassays of blends (low and high concentration). The table also provides information on the chemical classification of the compound and if it is a fungal compound. Bold compounds indicate substances used for the bioassays of blends.

Suppl. Table S5: Overview of the NMR data for the quantification of acetic acid from *A. hylecoeti* (N = 5) and two *Ips typopgraphus* yeasts (N = 6) from liquid PDB culture supernatant (Slide 1) as well of *E. dermestoides* field nests (Slide 2; N = 6).

Suppl. Table S6: Overview of statistical analyses including all *P* values sorted by figures.
