# Supplementary material for: Fungal symbiont of an ambrosia beetle possesses high nutrient content and suppresses competing fungi with antimicrobial compounds
Source: ISME J. 2025 Nov 20;19(1):wraf258. doi: 10.1093/ismejo/wraf258 (PMC12684719; doi:10.1093/ismejo/wraf258)
Supplement: suppl_wraf258 [file suppl_wraf258.zip › SI Results_Lehenberger et al.pdf]

Supplementary results Lehenberger et al.

Nutritional profiling of filamentous fungi

B vitamin analysis (Suppl. Fig. S2c) showed that *A. hylecoeti* had a higher content of these vitamins than the remaining four mutualistic fungi ($P < 0.018$; GLM with adjusted pairwise contrasts, see Suppl. Table S6 for individual p-values) as well as the three pathogenic fungi ($P < 0.0001$). There was no significant difference, however, between *A. hylecoeti* and *E. vermicola*, as well as with the wood-degrading fungi *L. edodes, L. sulphureus, and G. frondosa*, except *P. ulmarius* ($P < 0.0001$). In *A. hylecoeti*, we found thiamine, nicotinic acid, and pantothenic acid as the most abundant B vitamins (Suppl. Fig. S6).

Among the elements measured, potassium, phosphorus, and nitrogen are often limiting for many living organisms. Our measurements revealed significantly higher amounts of potassium (Suppl. Fig. S2d) in *A. hylecoeti* compared to 11 of the examined fungi ($P \leq 0.001$; GLM with adjusted pairwise contrasts) except *E. vermicola* and *L. sulphureus*. Similar to potassium, *A. hylecoeti* also contained the highest amounts of phosphorus (Suppl. Fig. S2e) compared to all remaining fungi ($P < 0.0001$) with *L. sulphureus* as the only exception. Analysis of the total nitrogen content (Suppl. Fig. S2f) showed that *A. hylecoeti* accumulated significantly more nitrogen compared to seven of the remaining fungi ($P < 0.03$), while there was no difference compared with *R. sulphurea*, *E. polonica*, *E. vermicola*, and *T. harzianum* and higher nitrogen levels in the biomass of *L. sulphureus* ($P < 0.0001$). In general, the most abundant elements among the fungi were sodium (especially for *A. hylecoeti*), phosphorus, sulfur, potassium, magnesium, and calcium (Suppl. Fig. S10).

For fatty acids (Suppl. Fig. S3a), *A. hylecoeti* contained the highest total fatty acid content among the mutualistic and wood-degrading fungi measured (P < 0.0001; GLM with adjusted pairwise contrasts), except for *L. sulphureus*, where we could not detect a significant difference. Here, also the nematophagous fungus *E. vermicola* and the pathogen *P. commune* did not differ from *A. hylecoeti*, while the potential myco-pathogens *C. globosum* and *T. harzianum* contained higher fatty acid levels (P < 0.021, P < 0.0001, respectively). Among fungi, the most dominant fatty acids were linolelaidic acid, cis-9-oleic acid, and palmitic acid (Suppl. Fig. S7 and S8).

Measurement of free ergosterol content (Suppl. Fig. S3b) in fungal biomass showed that *A. hylecoeti* was again among the fungi with the highest content, higher than ten of the remaining fungi (P < 0.00028; GLM with adjusted pairwise contrasts), while there was no difference with *T. harzianum* and a lower content compared to *L. sulphureus* (P < 0.025).

Nutrient content of field nests of ambrosia beetles

We examined field nests of the ambrosia beetles *X. saxesenii* and *E. dermestoides* focusing on free sugars, B vitamins, and amino acids (see Suppl. Fig. S9a-c) and compared our findings with the chemical contents of uncolonized beech wood. Here, we could not perform proper statistical analyses as the amount of uncolonized woody tissue surrounding each *E. dermestoides* field nest was high due to the sampling technique. However, our findings still indicate that nest areas of both beetle species are generally richer in soluble sugars, B vitamins, and free amino acids compared to uncolonized beech wood.

Lack of catabolism of phenolic compounds by *A. hylecoeti* in contrast to other fungi

First, we inoculated all five fungi on minimal beech sawdust-based medium and incubated them for a longer time course to clarify, if *A. hylecoeti* simply requires more time to metabolize the beech phenolics. After 28 days of incubation, the culture medium of *A. hylecoeti* was still more enriched with phenolics, such as syringic acid, caffeic acid, vanillic acid, and ferulic acid, than the control medium without fungi ($P < 0.0001$; GLM with adjusted pairwise contrasts, see Suppl. Table S6 for individual p-values as well as Fig. 2b and Suppl. Fig. S12). Protocatechuic acid was available in similar quantities compared to control while catechin and gallic acid were identified in slightly lower amounts after 28 d ($P < 0.0006$, $P < 0.003$, respectively). In contrast, the same phenolics were absent or only barely present in the culture medium of the remaining fungi (see Suppl. Fig. S13).

Identification of compounds released by fungi that may lower the pH of their surroundings

To determine the compound(s) released into the fungal medium that was capable of decreasing culture pH so drastically, we inoculated two *I. typographus* yeasts as well as *A. hylecoeti* and five other filamentous fungi used in this study in PDB medium and screened the culture supernatant for major organic acids via LC-MS. Succinic acid was identified as one of the most abundant acids in *A. hylecoeti* cultures as well as in *W. bisporus* (Fig. 4d and Suppl. Fig. 16c), followed by isopropylmalic acid and phenyllactic acid, which were, in contrast to succinic acid, also present in cultures of some of the remaining filamentous fungi (Suppl. Fig. S16a-b) and the two yeasts (Suppl. Fig. S16 d-f). Notably, the phenyllactic acid concentration in the culture supernatant of the yeasts (Suppl. Fig. 16d) was much higher compared to *A. hylecoeti* ($P < 0.0001$; GLM with adjusted pairwise contrasts, see Suppl. Table S6 for individual P values). Additionally, we identified kynurenic

acid in *W. bisporus* and *Y. scolyti* cultures (Suppl. Fig. S16e). As succinic acid was found in large amounts in *A. hylecoeti* cultures, we performed a bioassay with our panel of filamentous fungi (see Suppl. Fig. S17). At the concentration present in *A. hylecoeti* cultures (see Suppl. Table S4), succinic acid had no effect on the growth of *P. ostreatus*, *P. nameko*, *A. aegerita* as well as *A. hylecoeti* ($P > 0.2$, $P > 0.3$, $P > 0.1$, $P > 0.9$, respectively; t-test, see Suppl. Table S6), but showed an inhibitory trend for the antagonistic fungus *C. globosum* ($P < 0.051$; t-test, see Suppl. Table S6). Overall, none of these identified acids was present in a sufficient concentration to cause the pH decrease observed.
