# Supplementary material for: Fungal symbiont of an ambrosia beetle possesses high nutrient content and suppresses competing fungi with antimicrobial compounds
Source: ISME J. 2025 Nov 20;19(1):wraf258. doi: 10.1093/ismejo/wraf258 (PMC12684719; doi:10.1093/ismejo/wraf258)
Supplement: suppl_wraf258 [file suppl_wraf258.zip › Suppl. Fig. 11.pdf]

Group	Fungus	Protocatechuic acid	Gallic acid	Caffeic acid	Quinic acid	Ferulic acid	Syringic acid	Vanillic acid	Coumaric acid	Sinapic acid	Catechin
Control	**Sawdust**	41.9	109.1	1.1	9.2	2.7	41.8	18.6	1.6	0.6	86.5
Mutualists	***G. penicillata***	0	0	0.3	0	0.8	0	1.6	1.9	0	0
	E. polonica	0	0	0	65.3	2.4	26.9	27.8	2.4	1.2	2.3
	A. hylecoeti	22.2	72.9	6.6	0.4	18.2	74.9	41.4	7.7	3.8	31.9
	A. grosmanniae	5.3	0.9	1.3	59.9	8.7	19.8	148.2	4.0	0.9	1.0
	R. sulphurea	4.0	2.2	1.5	17.4	2.5	5.6	13.2	2.8	1.5	1.0
Nematophagous, non-mutualist	**E. vermicola**	0	0	0	31.5	1.7	0	23.2	1.9	0	0
Wood degraders	***G. frondosa***	0	0	0.3	0	1.0	0	1.5	2.0	0.3	0.4
	P. ulmarius	6.3	7.2	7.2	49.1	6.1	8.8	6.5	8.1	4.0	6.0
	L. edodes	1.9	0	0	27.1	0.6	0	0	2.0	0	0
	L. sulphureus	0	19.9	3.1	0	23.4	100.6	0	19.1	2.2	20.2
Potential pathogens	***C. globosum***	3.1	3.5	0.5	0	2.0	0	0	1.3	1.0	4.2
	P. commune	0	1.5	0	0	47.5	49.0	32.7	1.0	12.8	5.6
	Trichoderma sp.	2.1	5.5	0.5	0	64.3	16.5	4.9	0.9	1.8	10.1

(μg/g)

300
80
20
5
1
