# Supplementary material for: Fungal symbiont of an ambrosia beetle possesses high nutrient content and suppresses competing fungi with antimicrobial compounds
Source: ISME J. 2025 Nov 20;19(1):wraf258. doi: 10.1093/ismejo/wraf258 (PMC12684719; doi:10.1093/ismejo/wraf258)
Supplement: suppl_wraf258 [file suppl_wraf258.zip › Suppl. Table S6.docx]

**Overview of statistics and p-values**

| **Species_ID (see $Fungus)** | **Fungal Species** |
| --- | --- |
| P159 | *Raffaelea sulphurea* |
| P167 | *Esteya vermicola* |
| P188 | *Grosmannia penicillata* |
| P331 | *Endoconidiophora polonica* |
| P2, P339 | *Ambrosiella grosmanniae* |
| P10 | *Alloascoidea hylecoeti* |
| P21 | *Penicillium commune* |
| P7 | *Chaetomium globosum* |
| P338 | *Trichoderma harzianum* |
| P211 | *Lentinula edodes* |
| P209 | *Pleurotus ulmarius* |
| P148 | *Pleurotus ostreatus* |
| P213 | *Laetiporus sulphureus* |
| P207 | *Grifola frondosa* |
| Control_Sawdust | Beech medium |
| IT143 | *Wickerhamomyces bisporus* |
| IT130 | *Yamadazyma scolyti* |
| IT72 | *Kuraishia capsulata* |
| F60014 | *Cyberlindnera sp.* |
| F60015 | *Cyberlindnera sp.* |
| P394 | *Alloascoidea africana* |
| P395 | *Alloascoidea* sp. P395 |
| IT141 | *Nakazawaea holstii* |
| IT139 | *Danielozyma ontarioensis* |

**ns - P > 0.05, * - P < 0.05, ** - P < 0.01, *** - P < 0.001**

Statistical analyses are sorted by the respective figure in this supplementary document.

Nutritional analyses (**Figure 1a**)

**B Vitamins**

| Info | Value |
| --- | --- |
| Best model family | gaussian |
| Model name | Normal_log |

| **Resid. Df** | **Resid. Dev** | **Df** | **Deviance** | **Pr(>Chi)** |
| --- | --- | --- | --- | --- |
| 96 | 244.7836 |  |  |  |
| 86 | 24.41376 | 10 | 220.3698 | 2.6E-160 |

| **contrast** | **estimate** | **SE** | **df** | **t.ratio** | **p.value** | **Signif** |
| --- | --- | --- | --- | --- | --- | --- |
| 1_P10 - 2_P395 | -0.80752 | 0.238278 | 86 | -3.38898 | 0.001577 | ** |
| 1_P10 - 3_P394 | 0.21374 | 0.238278 | 86 | 0.89702 | 0.409434 |  |
| 1_P10 - 4_F60015 | -0.10552 | 0.262569 | 86 | -0.40187 | 0.714767 |  |
| 1_P10 - 5_F60014 | -0.44089 | 0.238278 | 86 | -1.85031 | 0.088659 |  |
| 1_P10 - 6_IT141 | 1.371501 | 0.238278 | 86 | 5.755895 | 2.55E-07 | *** |
| 1_P10 - 7_IT72 | -0.18315 | 0.262569 | 86 | -0.69753 | 0.525577 |  |
| 1_P10 - 8_IT130 | -1.85879 | 0.262569 | 86 | -7.07923 | 9.24E-10 | *** |
| 1_P10 - 9_IT143 | 0.15069 | 0.262569 | 86 | 0.573908 | 0.60027 |  |
| 1_P10 - 91_IT139 | 2.328678 | 0.238278 | 86 | 9.772965 | 5.17E-15 | *** |
| 1_P10 - 92_Control | 3.906461 | 0.244807 | 86 | 15.95732 | 2.23E-26 | *** |
| 2_P395 - 3_P394 | 1.021257 | 0.238278 | 86 | 4.285999 | 7.88E-05 | *** |
| 2_P395 - 4_F60015 | 0.701999 | 0.262569 | 86 | 2.673577 | 0.012663 | * |
| 2_P395 - 5_F60014 | 0.366631 | 0.238278 | 86 | 1.538673 | 0.163152 |  |
| 2_P395 - 6_IT141 | 2.179018 | 0.238278 | 86 | 9.144874 | 8.07E-14 | *** |
| 2_P395 - 7_IT72 | 0.624368 | 0.262569 | 86 | 2.377919 | 0.026987 | * |
| 2_P395 - 8_IT130 | -1.05127 | 0.262569 | 86 | -4.00378 | 0.000207 | *** |
| 2_P395 - 9_IT143 | 0.958208 | 0.262569 | 86 | 3.649356 | 0.000688 | *** |
| 2_P395 - 91_IT139 | 3.136196 | 0.238278 | 86 | 13.16194 | 1.42E-21 | *** |
| 2_P395 - 92_Control | 4.713979 | 0.244807 | 86 | 19.25591 | 1.71E-31 | *** |
| 3_P394 - 4_F60015 | -0.31926 | 0.262569 | 86 | -1.2159 | 0.27183 |  |
| 3_P394 - 5_F60014 | -0.65463 | 0.238278 | 86 | -2.74733 | 0.010592 | * |
| 3_P394 - 6_IT141 | 1.157761 | 0.238278 | 86 | 4.858875 | 9.32E-06 | *** |
| 3_P394 - 7_IT72 | -0.39689 | 0.262569 | 86 | -1.51156 | 0.167889 |  |
| 3_P394 - 8_IT130 | -2.07253 | 0.262569 | 86 | -7.89326 | 2.39E-11 | *** |
| 3_P394 - 9_IT143 | -0.06305 | 0.262569 | 86 | -0.24012 | 0.810805 |  |
| 3_P394 - 91_IT139 | 2.114938 | 0.238278 | 86 | 8.875945 | 2.69E-13 | *** |
| 3_P394 - 92_Control | 3.692721 | 0.244807 | 86 | 15.08422 | 5.49E-25 | *** |
| 4_F60015 - 5_F60014 | -0.33537 | 0.262569 | 86 | -1.27725 | 0.250495 |  |
| 4_F60015 - 6_IT141 | 1.47702 | 0.262569 | 86 | 5.625261 | 4.3E-07 | *** |
| 4_F60015 - 7_IT72 | -0.07763 | 0.284796 | 86 | -0.27258 | 0.800379 |  |
| 4_F60015 - 8_IT130 | -1.75327 | 0.284796 | 86 | -6.15622 | 5.06E-08 | *** |
| 4_F60015 - 9_IT143 | 0.256209 | 0.284796 | 86 | 0.899624 | 0.409434 |  |
| 4_F60015 - 91_IT139 | 2.434197 | 0.262569 | 86 | 9.270693 | 4.75E-14 | *** |
| 4_F60015 - 92_Control | 4.01198 | 0.268508 | 86 | 14.94173 | 8.7E-25 | *** |
| 5_F60014 - 6_IT141 | 1.812387 | 0.238278 | 86 | 7.606201 | 8.61E-11 | *** |
| 5_F60014 - 7_IT72 | 0.257737 | 0.262569 | 86 | 0.981595 | 0.377039 |  |
| 5_F60014 - 8_IT130 | -1.4179 | 0.262569 | 86 | -5.4001 | 1.07E-06 | *** |
| 5_F60014 - 9_IT143 | 0.591577 | 0.262569 | 86 | 2.253033 | 0.035954 | * |
| 5_F60014 - 91_IT139 | 2.769565 | 0.238278 | 86 | 11.62327 | 1.16E-18 | *** |
| 5_F60014 - 92_Control | 4.347348 | 0.244807 | 86 | 17.75827 | 3.02E-29 | *** |
| 6_IT141 - 7_IT72 | -1.55465 | 0.262569 | 86 | -5.92092 | 1.35E-07 | *** |
| 6_IT141 - 8_IT130 | -3.23029 | 0.262569 | 86 | -12.3026 | 5.87E-20 | *** |
| 6_IT141 - 9_IT143 | -1.22081 | 0.262569 | 86 | -4.64948 | 2.05E-05 | *** |
| 6_IT141 - 91_IT139 | 0.957178 | 0.238278 | 86 | 4.01707 | 0.000203 | *** |
| 6_IT141 - 92_Control | 2.534961 | 0.244807 | 86 | 10.35494 | 3.69E-16 | *** |
| 7_IT72 - 8_IT130 | -1.67564 | 0.284796 | 86 | -5.88364 | 1.53E-07 | *** |
| 7_IT72 - 9_IT143 | 0.33384 | 0.284796 | 86 | 1.172207 | 0.285942 |  |
| 7_IT72 - 91_IT139 | 2.511828 | 0.262569 | 86 | 9.566351 | 1.27E-14 | *** |
| 7_IT72 - 92_Control | 4.089611 | 0.268508 | 86 | 15.23085 | 3.49E-25 | *** |
| 8_IT130 - 9_IT143 | 2.009477 | 0.284796 | 86 | 7.055843 | 9.83E-10 | *** |
| 8_IT130 - 91_IT139 | 4.187465 | 0.262569 | 86 | 15.94805 | 2.23E-26 | *** |
| 8_IT130 - 92_Control | 5.765248 | 0.268508 | 86 | 21.47139 | 1.44E-34 | *** |
| 9_IT143 - 91_IT139 | 2.177988 | 0.262569 | 86 | 8.294913 | 3.87E-12 | *** |
| 9_IT143 - 92_Control | 3.755771 | 0.268508 | 86 | 13.98754 | 4.39E-23 | *** |
| 91_IT139 - 92_Control | 1.577783 | 0.244807 | 86 | 6.44501 | 1.47E-08 | *** |

| Sample | mean | SE | lower.CL | upper.CL | group |
| --- | --- | --- | --- | --- | --- |
| 92_Control | 0.737341 | 0.177602 | 0.440061 | 1.235444 | a |
| 91_IT139 | 3.571828 | 0.168488 | 2.188964 | 5.828309 | b |
| 6_IT141 | 9.30224 | 0.168488 | 5.700797 | 15.17887 | c |
| 3_P394 | 29.60716 | 0.168488 | 18.14449 | 48.3113 | d |
| 9_IT143 | 31.53397 | 0.201381 | 17.56349 | 56.61695 | d |
| 1_P10 | 36.66255 | 0.168488 | 22.46833 | 59.8239 | de |
| 4_F60015 | 40.74263 | 0.201381 | 22.69244 | 73.15041 | de |
| 7_IT72 | 44.03152 | 0.201381 | 24.52426 | 79.05537 | de |
| 5_F60014 | 56.97669 | 0.168488 | 34.91767 | 92.97137 | ef |
| 2_P395 | 82.20972 | 0.168488 | 50.38151 | 134.1452 | f |
| 8_IT130 | 235.225 | 0.201381 | 131.0134 | 422.3292 | g |

**Soluble sugars**

| Info | Value |
| --- | --- |
| Best model family | gaussian |
| Model name | Normal_log |

| **Resid. Df** | **Resid. Dev** | **Df** | **Deviance** | **Pr(>Chi)** |
| --- | --- | --- | --- | --- |
| 96 | 141.0095 |  |  |  |
| 86 | 7.599824 | 10 | 133.4097 | 0 |

| **contrast** | **estimate** | **SE** | **df** | **t.ratio** | **p.value** | **Signif** |
| --- | --- | --- | --- | --- | --- | --- |
| 1_P10 - 2_P395 | 0.371668 | 0.132944 | 86 | 2.795683 | 0.007984 | ** |
| 1_P10 - 3_P394 | 1.834242 | 0.132944 | 86 | 13.79714 | 6.4E-23 | *** |
| 1_P10 - 4_F60015 | 1.339157 | 0.146497 | 86 | 9.141203 | 6.35E-14 | *** |
| 1_P10 - 5_F60014 | 1.763296 | 0.132944 | 86 | 13.26349 | 6.08E-22 | *** |
| 1_P10 - 6_IT141 | 1.939923 | 0.132944 | 86 | 14.59207 | 2.32E-24 | *** |
| 1_P10 - 7_IT72 | 0.838666 | 0.146497 | 86 | 5.724808 | 2.63E-07 | *** |
| 1_P10 - 8_IT130 | 2.287243 | 0.146497 | 86 | 15.61293 | 3.95E-26 | *** |
| 1_P10 - 9_IT143 | 1.506388 | 0.146497 | 86 | 10.28274 | 3.19E-16 | *** |
| 1_P10 - 91_IT139 | 2.085664 | 0.132944 | 86 | 15.68833 | 3.2E-26 | *** |
| 1_P10 - 92_Control | 4.602708 | 0.136587 | 86 | 33.6981 | 1.37E-49 | *** |
| 2_P395 - 3_P394 | 1.462574 | 0.132944 | 86 | 11.00146 | 1.26E-17 | *** |
| 2_P395 - 4_F60015 | 0.967488 | 0.146497 | 86 | 6.604162 | 6.21E-09 | *** |
| 2_P395 - 5_F60014 | 1.391628 | 0.132944 | 86 | 10.4678 | 1.42E-16 | *** |
| 2_P395 - 6_IT141 | 1.568254 | 0.132944 | 86 | 11.79639 | 3.52E-19 | *** |
| 2_P395 - 7_IT72 | 0.466997 | 0.146497 | 86 | 3.187767 | 0.00282 | ** |
| 2_P395 - 8_IT130 | 1.915574 | 0.146497 | 86 | 13.07588 | 1.3E-21 | *** |
| 2_P395 - 9_IT143 | 1.13472 | 0.146497 | 86 | 7.745701 | 3.79E-11 | *** |
| 2_P395 - 91_IT139 | 1.713996 | 0.132944 | 86 | 12.89265 | 2.74E-21 | *** |
| 2_P395 - 92_Control | 4.23104 | 0.136587 | 86 | 30.97698 | 5.52E-47 | *** |
| 3_P394 - 4_F60015 | -0.49509 | 0.146497 | 86 | -3.3795 | 0.001583 | ** |
| 3_P394 - 5_F60014 | -0.07095 | 0.132944 | 86 | -0.53366 | 0.594956 |  |
| 3_P394 - 6_IT141 | 0.10568 | 0.132944 | 86 | 0.794925 | 0.436787 |  |
| 3_P394 - 7_IT72 | -0.99558 | 0.146497 | 86 | -6.7959 | 2.72E-09 | *** |
| 3_P394 - 8_IT130 | 0.453 | 0.146497 | 86 | 3.092221 | 0.003593 | ** |
| 3_P394 - 9_IT143 | -0.32785 | 0.146497 | 86 | -2.23796 | 0.032541 | * |
| 3_P394 - 91_IT139 | 0.251421 | 0.132944 | 86 | 1.891188 | 0.071002 |  |
| 3_P394 - 92_Control | 2.768466 | 0.136587 | 86 | 20.26894 | 1.3E-33 | *** |
| 4_F60015 - 5_F60014 | 0.42414 | 0.146497 | 86 | 2.895216 | 0.006142 | ** |
| 4_F60015 - 6_IT141 | 0.600766 | 0.146497 | 86 | 4.100884 | 0.000146 | *** |
| 4_F60015 - 7_IT72 | -0.50049 | 0.158898 | 86 | -3.14976 | 0.003091 | ** |
| 4_F60015 - 8_IT130 | 0.948086 | 0.158898 | 86 | 5.966633 | 9.62E-08 | *** |
| 4_F60015 - 9_IT143 | 0.167232 | 0.158898 | 86 | 1.052447 | 0.306695 |  |
| 4_F60015 - 91_IT139 | 0.746507 | 0.146497 | 86 | 5.095726 | 3.5E-06 | *** |
| 4_F60015 - 92_Control | 3.263552 | 0.14981 | 86 | 21.78453 | 1.26E-35 | *** |
| 5_F60014 - 6_IT141 | 0.176626 | 0.132944 | 86 | 1.328581 | 0.202207 |  |
| 5_F60014 - 7_IT72 | -0.92463 | 0.146497 | 86 | -6.31161 | 2.2E-08 | *** |
| 5_F60014 - 8_IT130 | 0.523946 | 0.146497 | 86 | 3.576506 | 0.000854 | *** |
| 5_F60014 - 9_IT143 | -0.25691 | 0.146497 | 86 | -1.75368 | 0.093218 |  |
| 5_F60014 - 91_IT139 | 0.322368 | 0.132944 | 86 | 2.424844 | 0.021276 | * |
| 5_F60014 - 92_Control | 2.839412 | 0.136587 | 86 | 20.78837 | 2.98E-34 | *** |
| 6_IT141 - 7_IT72 | -1.10126 | 0.146497 | 86 | -7.51728 | 1.05E-10 | *** |
| 6_IT141 - 8_IT130 | 0.34732 | 0.146497 | 86 | 2.370838 | 0.023892 | * |
| 6_IT141 - 9_IT143 | -0.43353 | 0.146497 | 86 | -2.95934 | 0.005215 | ** |
| 6_IT141 - 91_IT139 | 0.145741 | 0.132944 | 86 | 1.096263 | 0.291948 |  |
| 6_IT141 - 92_Control | 2.662786 | 0.136587 | 86 | 19.49522 | 1.8E-32 | *** |
| 7_IT72 - 8_IT130 | 1.448577 | 0.158898 | 86 | 9.116393 | 6.82E-14 | *** |
| 7_IT72 - 9_IT143 | 0.667723 | 0.158898 | 86 | 4.202208 | 0.000104 | *** |
| 7_IT72 - 91_IT139 | 1.246998 | 0.146497 | 86 | 8.512121 | 1.11E-12 | *** |
| 7_IT72 - 92_Control | 3.764043 | 0.14981 | 86 | 25.12536 | 4.1E-40 | *** |
| 8_IT130 - 9_IT143 | -0.78085 | 0.158898 | 86 | -4.91419 | 7.03E-06 | *** |
| 8_IT130 - 91_IT139 | -0.20158 | 0.146497 | 86 | -1.376 | 0.189635 |  |
| 8_IT130 - 92_Control | 2.315466 | 0.14981 | 86 | 15.45596 | 6.9E-26 | *** |
| 9_IT143 - 91_IT139 | 0.579276 | 0.146497 | 86 | 3.954187 | 0.00024 | *** |
| 9_IT143 - 92_Control | 3.09632 | 0.14981 | 86 | 20.66825 | 3.77E-34 | *** |
| 91_IT139 - 92_Control | 2.517045 | 0.136587 | 86 | 18.4282 | 8.01E-31 | *** |

| Sample | mean | SE | lower.CL | upper.CL | group |
| --- | --- | --- | --- | --- | --- |
| 92_Control | 1.115755 | 0.09909 | 0.836575 | 1.488103 | a |
| 8_IT130 | 11.3022 | 0.112358 | 8.153678 | 15.66651 | b |
| 91_IT139 | 13.82635 | 0.094005 | 10.5211 | 18.16995 | bc |
| 6_IT141 | 15.99566 | 0.094005 | 12.17183 | 21.02075 | cd |
| 3_P394 | 17.77863 | 0.094005 | 13.52858 | 23.36386 | cd |
| 5_F60014 | 19.08578 | 0.094005 | 14.52325 | 25.08165 | de |
| 9_IT143 | 24.67651 | 0.112358 | 17.80223 | 34.20527 | ef |
| 4_F60015 | 29.16833 | 0.112358 | 21.04273 | 40.43159 | f |
| 7_IT72 | 48.11404 | 0.112358 | 34.71063 | 66.69315 | g |
| 2_P395 | 76.7514 | 0.094005 | 58.40366 | 100.8631 | h |
| 1_P10 | 111.3012 | 0.094005 | 84.69418 | 146.2669 | i |

**Free amino acids**

| Info | Value |
| --- | --- |
| Best model family | gaussian |
| Model name | Normal_log |

| **Resid. Df** | **Resid. Dev** | **Df** | **Deviance** | **Pr(>Chi)** |
| --- | --- | --- | --- | --- |
| 96 | 381.0086 |  |  |  |
| 86 | 5.55064 | 10 | 375.4579 | 0 |

| **contrast** | **estimate** | **SE** | **df** | **t.ratio** | **p.value** | **Signif** |
| --- | --- | --- | --- | --- | --- | --- |
| 1_P10 - 2_P395 | -0.37353 | 0.113615 | 86 | -3.28763 | 0.001964 | ** |
| 1_P10 - 3_P394 | 0.555901 | 0.113615 | 86 | 4.892834 | 6.68E-06 | *** |
| 1_P10 - 4_F60015 | 0.899504 | 0.125198 | 86 | 7.184645 | 4.33E-10 | *** |
| 1_P10 - 5_F60014 | 0.932029 | 0.113615 | 86 | 8.20337 | 4.7E-12 | *** |
| 1_P10 - 6_IT141 | 1.629895 | 0.113615 | 86 | 14.34572 | 6.11E-24 | *** |
| 1_P10 - 7_IT72 | 1.873417 | 0.125198 | 86 | 14.96362 | 4.88E-25 | *** |
| 1_P10 - 8_IT130 | 0.882847 | 0.125198 | 86 | 7.051597 | 7.2E-10 | *** |
| 1_P10 - 9_IT143 | 1.209142 | 0.125198 | 86 | 9.657833 | 5.91E-15 | *** |
| 1_P10 - 91_IT139 | 0.818631 | 0.113615 | 86 | 7.205284 | 4.08E-10 | *** |
| 1_P10 - 92_Control | 7.2285 | 0.116729 | 86 | 61.92562 | 1.11E-71 | *** |
| 2_P395 - 3_P394 | 0.929426 | 0.113615 | 86 | 8.18046 | 5.02E-12 | *** |
| 2_P395 - 4_F60015 | 1.273029 | 0.125198 | 86 | 10.16812 | 6.02E-16 | *** |
| 2_P395 - 5_F60014 | 1.305554 | 0.113615 | 86 | 11.491 | 1.59E-18 | *** |
| 2_P395 - 6_IT141 | 2.00342 | 0.113615 | 86 | 17.63334 | 1.22E-29 | *** |
| 2_P395 - 7_IT72 | 2.246942 | 0.125198 | 86 | 17.94709 | 4.01E-30 | *** |
| 2_P395 - 8_IT130 | 1.256372 | 0.125198 | 86 | 10.03507 | 1.06E-15 | *** |
| 2_P395 - 9_IT143 | 1.582667 | 0.125198 | 86 | 12.64131 | 9.48E-21 | *** |
| 2_P395 - 91_IT139 | 1.192156 | 0.113615 | 86 | 10.49291 | 1.4E-16 | *** |
| 2_P395 - 92_Control | 7.602025 | 0.116729 | 86 | 65.12556 | 3.19E-73 | *** |
| 3_P394 - 4_F60015 | 0.343602 | 0.125198 | 86 | 2.74447 | 0.009436 | ** |
| 3_P394 - 5_F60014 | 0.376128 | 0.113615 | 86 | 3.310536 | 0.001873 | ** |
| 3_P394 - 6_IT141 | 1.073993 | 0.113615 | 86 | 9.452882 | 1.47E-14 | *** |
| 3_P394 - 7_IT72 | 1.317515 | 0.125198 | 86 | 10.52344 | 1.29E-16 | *** |
| 3_P394 - 8_IT130 | 0.326945 | 0.125198 | 86 | 2.611423 | 0.013297 | * |
| 3_P394 - 9_IT143 | 0.653241 | 0.125198 | 86 | 5.217658 | 1.89E-06 | *** |
| 3_P394 - 91_IT139 | 0.26273 | 0.113615 | 86 | 2.31245 | 0.027669 | * |
| 3_P394 - 92_Control | 6.672599 | 0.116729 | 86 | 57.16329 | 6.14E-69 | *** |
| 4_F60015 - 5_F60014 | 0.032526 | 0.125198 | 86 | 0.259792 | 0.810379 |  |
| 4_F60015 - 6_IT141 | 0.730391 | 0.125198 | 86 | 5.833881 | 1.46E-07 | *** |
| 4_F60015 - 7_IT72 | 0.973913 | 0.135796 | 86 | 7.171859 | 4.44E-10 | *** |
| 4_F60015 - 8_IT130 | -0.01666 | 0.135796 | 86 | -0.12266 | 0.90266 |  |
| 4_F60015 - 9_IT143 | 0.309638 | 0.135796 | 86 | 2.280167 | 0.029339 | * |
| 4_F60015 - 91_IT139 | -0.08087 | 0.125198 | 86 | -0.64596 | 0.560814 |  |
| 4_F60015 - 92_Control | 6.328996 | 0.12803 | 86 | 49.43367 | 4.87E-64 | *** |
| 5_F60014 - 6_IT141 | 0.697865 | 0.113615 | 86 | 6.142346 | 4.07E-08 | *** |
| 5_F60014 - 7_IT72 | 0.941387 | 0.125198 | 86 | 7.519181 | 1.04E-10 | *** |
| 5_F60014 - 8_IT130 | -0.04918 | 0.125198 | 86 | -0.39284 | 0.721652 |  |
| 5_F60014 - 9_IT143 | 0.277113 | 0.125198 | 86 | 2.213396 | 0.033822 | * |
| 5_F60014 - 91_IT139 | -0.1134 | 0.113615 | 86 | -0.99809 | 0.353142 |  |
| 5_F60014 - 92_Control | 6.296471 | 0.116729 | 86 | 53.94105 | 4.74E-67 | *** |
| 6_IT141 - 7_IT72 | 0.243522 | 0.125198 | 86 | 1.945093 | 0.06177 |  |
| 6_IT141 - 8_IT130 | -0.74705 | 0.125198 | 86 | -5.96693 | 8.48E-08 | *** |
| 6_IT141 - 9_IT143 | -0.42075 | 0.125198 | 86 | -3.36069 | 0.001638 | ** |
| 6_IT141 - 91_IT139 | -0.81126 | 0.113615 | 86 | -7.14043 | 4.96E-10 | *** |
| 6_IT141 - 92_Control | 5.598606 | 0.116729 | 86 | 47.96253 | 5.24E-63 | *** |
| 7_IT72 - 8_IT130 | -0.99057 | 0.135796 | 86 | -7.29452 | 2.81E-10 | *** |
| 7_IT72 - 9_IT143 | -0.66427 | 0.135796 | 86 | -4.89169 | 6.68E-06 | *** |
| 7_IT72 - 91_IT139 | -1.05479 | 0.125198 | 86 | -8.42493 | 1.74E-12 | *** |
| 7_IT72 - 92_Control | 5.355084 | 0.12803 | 86 | 41.82676 | 3.36E-58 | *** |
| 8_IT130 - 9_IT143 | 0.326296 | 0.135796 | 86 | 2.40283 | 0.022512 | * |
| 8_IT130 - 91_IT139 | -0.06422 | 0.125198 | 86 | -0.51291 | 0.644484 |  |
| 8_IT130 - 92_Control | 6.345654 | 0.12803 | 86 | 49.56377 | 4.57E-64 | *** |
| 9_IT143 - 91_IT139 | -0.39051 | 0.125198 | 86 | -3.11914 | 0.003232 | ** |
| 9_IT143 - 92_Control | 6.019358 | 0.12803 | 86 | 47.01519 | 2.43E-62 | *** |
| 91_IT139 - 92_Control | 6.409869 | 0.116729 | 86 | 54.91251 | 1.33E-67 | *** |

| **Sample** | **mean** | **SE** | **lower.CL** | **upper.CL** | **group** |
| --- | --- | --- | --- | --- | --- |
| 92_Control | 0.382747 | 0.084684 | 0.299247 | 0.489546 | a |
| 7_IT72 | 81.02052 | 0.096023 | 61.29187 | 107.0994 | b |
| 6_IT141 | 103.3607 | 0.080338 | 81.83868 | 130.5425 | b |
| 9_IT143 | 157.4293 | 0.096023 | 119.095 | 208.1028 | c |
| 5_F60014 | 207.699 | 0.080338 | 164.4514 | 262.3197 | d |
| 4_F60015 | 214.5655 | 0.096023 | 162.3184 | 283.63 | d |
| 8_IT130 | 218.1695 | 0.096023 | 165.0448 | 288.394 | d |
| 91_IT139 | 232.6389 | 0.080338 | 184.1984 | 293.8184 | d |
| 3_P394 | 302.5413 | 0.080338 | 239.5455 | 382.1037 | e |
| 1_P10 | 527.4843 | 0.080338 | 417.6504 | 666.2024 | f |
| 2_P395 | 766.3539 | 0.080338 | 606.7821 | 967.8901 | g |

**PCA analysis (Fig. 1b)**

**Yeasts and *A. hylecoeti***

Permutation test for adonis under reduced model

Permutation: free

Number of permutations: 9999

adonis2(formula = dist_mat ~ Sample, data = data, permutations = 9999)

Df SumOfSqs R2 F Pr(>F)

Model 10 2.78653 0.97885 397.99 1e-04 ***

Residual 86 0.06021 0.02115

Total 96 2.84674 1.00000

---

Signif. codes: 0 ‘***’ 0.001 ‘**’ 0.01 ‘*’ 0.05 ‘.’ 0.1 ‘ ’ 1

>

**PCA analysis (Fig. 1b)**

**Yeasts and *Alloascoidea***

Pairwise PERMANOVA with BH correction

| **Group1** | **Group2** | **PERMANOVA_F** | **PERMANOVA_p** | **PERMANOVA_p_adj** | **PERMANOVA_Sig** | **Global_PERMANOVA_F** | **Global_PERMANOVA_p** |
| --- | --- | --- | --- | --- | --- | --- | --- |
| Control | IT141 | 516.168 | 0.0001 | 0.000167 | *** | 397.987 | 0.0001 |
| Control | F60014 | 1133.044 | 0.0001 | 0.000167 | *** | 397.987 | 0.0001 |
| Control | IT139 | 1130.767 | 0.0001 | 0.000167 | *** | 397.987 | 0.0001 |
| Control | P10 | 1439.334 | 0.0001 | 0.000167 | *** | 397.987 | 0.0001 |
| Control | P394 | 1301.757 | 0.0001 | 0.000167 | *** | 397.987 | 0.0001 |
| Control | P395 | 1444.15 | 0.0001 | 0.000167 | *** | 397.987 | 0.0001 |
| Control | IT72 | 635.571 | 0.0005 | 0.000585 | *** | 397.987 | 0.0001 |
| Control | IT130 | 818.424 | 0.0002 | 0.000262 | *** | 397.987 | 0.0001 |
| Control | F60015 | 703.237 | 0.0002 | 0.000262 | *** | 397.987 | 0.0001 |
| Control | IT143 | 817.275 | 0.0001 | 0.000167 | *** | 397.987 | 0.0001 |
| IT141 | F60014 | 15.239 | 0.0001 | 0.000167 | *** | 397.987 | 0.0001 |
| IT141 | IT139 | 15.559 | 0.0001 | 0.000167 | *** | 397.987 | 0.0001 |
| IT141 | P10 | 65.129 | 0.0001 | 0.000167 | *** | 397.987 | 0.0001 |
| IT141 | P394 | 39.537 | 0.0001 | 0.000167 | *** | 397.987 | 0.0001 |
| IT141 | P395 | 85.531 | 0.0001 | 0.000167 | *** | 397.987 | 0.0001 |
| IT141 | IT72 | 11.792 | 0.0002 | 0.000262 | *** | 397.987 | 0.0001 |
| IT141 | IT130 | 18.324 | 0.0003 | 0.000367 | *** | 397.987 | 0.0001 |
| IT141 | F60015 | 12.45 | 0.0011 | 0.00112 | ** | 397.987 | 0.0001 |
| IT141 | IT143 | 12.761 | 0.001 | 0.001038 | ** | 397.987 | 0.0001 |
| F60014 | IT139 | 41.808 | 0.0001 | 0.000167 | *** | 397.987 | 0.0001 |
| F60014 | P10 | 173.151 | 0.0001 | 0.000167 | *** | 397.987 | 0.0001 |
| F60014 | P394 | 89.904 | 0.0001 | 0.000167 | *** | 397.987 | 0.0001 |
| F60014 | P395 | 210.98 | 0.0001 | 0.000167 | *** | 397.987 | 0.0001 |
| F60014 | IT72 | 63.369 | 0.0001 | 0.000167 | *** | 397.987 | 0.0001 |
| F60014 | IT130 | 60.882 | 0.0001 | 0.000167 | *** | 397.987 | 0.0001 |
| F60014 | F60015 | 3.805 | 0.0245 | 0.0245 | * | 397.987 | 0.0001 |
| F60014 | IT143 | 79.886 | 0.0001 | 0.000167 | *** | 397.987 | 0.0001 |
| IT139 | P10 | 282.129 | 0.0001 | 0.000167 | *** | 397.987 | 0.0001 |
| IT139 | P394 | 171.67 | 0.0001 | 0.000167 | *** | 397.987 | 0.0001 |
| IT139 | P395 | 335.868 | 0.0002 | 0.000262 | *** | 397.987 | 0.0001 |
| IT139 | IT72 | 80.545 | 0.0001 | 0.000167 | *** | 397.987 | 0.0001 |
| IT139 | IT130 | 54.829 | 0.0001 | 0.000167 | *** | 397.987 | 0.0001 |
| IT139 | F60015 | 21.009 | 0.0002 | 0.000262 | *** | 397.987 | 0.0001 |
| IT139 | IT143 | 103.663 | 0.0002 | 0.000262 | *** | 397.987 | 0.0001 |
| P10 | P394 | 143.719 | 0.0001 | 0.000167 | *** | 397.987 | 0.0001 |
| P10 | P395 | 57.034 | 0.0001 | 0.000167 | *** | 397.987 | 0.0001 |
| P10 | IT72 | 181.653 | 0.0004 | 0.000478 | *** | 397.987 | 0.0001 |
| P10 | IT130 | 153.894 | 0.0001 | 0.000167 | *** | 397.987 | 0.0001 |
| P10 | F60015 | 61.144 | 0.0001 | 0.000167 | *** | 397.987 | 0.0001 |
| P10 | IT143 | 464.202 | 0.0003 | 0.000367 | *** | 397.987 | 0.0001 |
| P394 | P395 | 144.861 | 0.0001 | 0.000167 | *** | 397.987 | 0.0001 |
| P394 | IT72 | 167.091 | 0.0001 | 0.000167 | *** | 397.987 | 0.0001 |
| P394 | IT130 | 118.075 | 0.0002 | 0.000262 | *** | 397.987 | 0.0001 |
| P394 | F60015 | 33.997 | 0.0001 | 0.000167 | *** | 397.987 | 0.0001 |
| P394 | IT143 | 288.596 | 0.0002 | 0.000262 | *** | 397.987 | 0.0001 |
| P395 | IT72 | 223.655 | 0.0001 | 0.000167 | *** | 397.987 | 0.0001 |
| P395 | IT130 | 175.033 | 0.0001 | 0.000167 | *** | 397.987 | 0.0001 |
| P395 | F60015 | 80.499 | 0.0001 | 0.000167 | *** | 397.987 | 0.0001 |
| P395 | IT143 | 402.776 | 0.0002 | 0.000262 | *** | 397.987 | 0.0001 |
| IT72 | IT130 | 53.183 | 0.0006 | 0.00066 | *** | 397.987 | 0.0001 |
| IT72 | F60015 | 28.699 | 0.0006 | 0.00066 | *** | 397.987 | 0.0001 |
| IT72 | IT143 | 38.405 | 0.0007 | 0.000755 | *** | 397.987 | 0.0001 |
| IT130 | F60015 | 25.844 | 0.0008 | 0.000846 | *** | 397.987 | 0.0001 |
| IT130 | IT143 | 78.997 | 0.0006 | 0.00066 | *** | 397.987 | 0.0001 |
| F60015 | IT143 | 28.165 | 0.0003 | 0.000367 | *** | 397.987 | 0.0001 |

**PCA analysis (Fig. 1c)**

**Filamentous fungi and *A. hylecoeti***

Permutation test for adonis under reduced model

Permutation: free

Number of permutations: 9999

adonis2(formula = dist_mat ~ group_clean, permutations = 9999)

Df SumOfSqs R2 F Pr(>F)

Model 13 6.9513 0.95518 111.47 1e-04 ***

Residual 68 0.3262 0.04482

Total 81 7.2775 1.00000

---

Signif. codes: 0 ‘***’ 0.001 ‘**’ 0.01 ‘*’ 0.05 ‘.’ 0.1 ‘ ’ 1

**PCA analysis (Fig. 1c)**

Pairwise PERMANOVA with BH correction

| Group1 | Group2 | PERMANOVA_F | PERMANOVA_p | PERMANOVA_p_adj | PERMANOVA_Sig | Global_PERMANOVA_F | Global_PERMANOVA_p |
| --- | --- | --- | --- | --- | --- | --- | --- |
| 159 | 167 | 34.573 | 0.0043 | 0.00434778 | ** | 111.47 | 0.0001 |
| 159 | 188 | 14.189 | 0.0021 | 0.00310732 | ** | 111.47 | 0.0001 |
| 159 | 2 | 22.258 | 0.0022 | 0.00310732 | ** | 111.47 | 0.0001 |
| 159 | 207 | 18.941 | 0.002 | 0.00310732 | ** | 111.47 | 0.0001 |
| 159 | 209 | 30.281 | 0.0017 | 0.00310732 | ** | 111.47 | 0.0001 |
| 159 | 21 | 21.976 | 0.0026 | 0.00310732 | ** | 111.47 | 0.0001 |
| 159 | 211 | 31.528 | 0.0014 | 0.00310732 | ** | 111.47 | 0.0001 |
| 159 | 213 | 33.259 | 0.0015 | 0.00310732 | ** | 111.47 | 0.0001 |
| 159 | 338 | 53.215 | 0.0017 | 0.00310732 | ** | 111.47 | 0.0001 |
| 159 | 42_P10 | 62.459 | 0.0023 | 0.00310732 | ** | 111.47 | 0.0001 |
| 159 | 7 | 33.711 | 0.002 | 0.00310732 | ** | 111.47 | 0.0001 |
| 159 | Control Sawdust | 197.863 | 0.0024 | 0.00310732 | ** | 111.47 | 0.0001 |
| 159 | P311 | 7.589 | 0.0026 | 0.00310732 | ** | 111.47 | 0.0001 |
| 167 | 188 | 98.827 | 0.0023 | 0.00310732 | ** | 111.47 | 0.0001 |
| 167 | 2 | 111.339 | 0.0017 | 0.00310732 | ** | 111.47 | 0.0001 |
| 167 | 207 | 35.403 | 0.0025 | 0.00310732 | ** | 111.47 | 0.0001 |
| 167 | 209 | 62.217 | 0.0026 | 0.00310732 | ** | 111.47 | 0.0001 |
| 167 | 21 | 154.197 | 0.0024 | 0.00310732 | ** | 111.47 | 0.0001 |
| 167 | 211 | 82.078 | 0.0023 | 0.00310732 | ** | 111.47 | 0.0001 |
| 167 | 213 | 33.954 | 0.0019 | 0.00310732 | ** | 111.47 | 0.0001 |
| 167 | 338 | 276.872 | 0.0024 | 0.00310732 | ** | 111.47 | 0.0001 |
| 167 | 42_P10 | 116.941 | 0.0019 | 0.00310732 | ** | 111.47 | 0.0001 |
| 167 | 7 | 382.508 | 0.0093 | 0.0093 | ** | 111.47 | 0.0001 |
| 167 | Control Sawdust | 752.8 | 0.0022 | 0.00310732 | ** | 111.47 | 0.0001 |
| 167 | P311 | 70.293 | 0.0017 | 0.00310732 | ** | 111.47 | 0.0001 |
| 188 | 2 | 58.18 | 0.0021 | 0.00310732 | ** | 111.47 | 0.0001 |
| 188 | 207 | 17.073 | 0.0013 | 0.00310732 | ** | 111.47 | 0.0001 |
| 188 | 209 | 48.905 | 0.0018 | 0.00310732 | ** | 111.47 | 0.0001 |
| 188 | 21 | 87.932 | 0.0023 | 0.00310732 | ** | 111.47 | 0.0001 |
| 188 | 211 | 78.427 | 0.0025 | 0.00310732 | ** | 111.47 | 0.0001 |
| 188 | 213 | 65.383 | 0.0027 | 0.00310732 | ** | 111.47 | 0.0001 |
| 188 | 338 | 227.454 | 0.0029 | 0.00314167 | ** | 111.47 | 0.0001 |
| 188 | 42_P10 | 247.745 | 0.0024 | 0.00310732 | ** | 111.47 | 0.0001 |
| 188 | 7 | 223.87 | 0.0028 | 0.00310732 | ** | 111.47 | 0.0001 |
| 188 | Control Sawdust | 669.973 | 0.0025 | 0.00310732 | ** | 111.47 | 0.0001 |
| 188 | P311 | 22.497 | 0.0021 | 0.00310732 | ** | 111.47 | 0.0001 |
| 2 | 207 | 25.106 | 0.0027 | 0.00310732 | ** | 111.47 | 0.0001 |
| 2 | 209 | 59.899 | 0.0022 | 0.00310732 | ** | 111.47 | 0.0001 |
| 2 | 21 | 47.975 | 0.0023 | 0.00310732 | ** | 111.47 | 0.0001 |
| 2 | 211 | 71.758 | 0.002 | 0.00310732 | ** | 111.47 | 0.0001 |
| 2 | 213 | 74.142 | 0.0024 | 0.00310732 | ** | 111.47 | 0.0001 |
| 2 | 338 | 139.516 | 0.0017 | 0.00310732 | ** | 111.47 | 0.0001 |
| 2 | 42_P10 | 218.02 | 0.0033 | 0.00337416 | ** | 111.47 | 0.0001 |
| 2 | 7 | 95.67 | 0.0021 | 0.00310732 | ** | 111.47 | 0.0001 |
| 2 | Control Sawdust | 464.686 | 0.0018 | 0.00310732 | ** | 111.47 | 0.0001 |
| 2 | P311 | 32.922 | 0.0018 | 0.00310732 | ** | 111.47 | 0.0001 |
| 207 | 209 | 16.376 | 0.003 | 0.00321176 | ** | 111.47 | 0.0001 |
| 207 | 21 | 25.586 | 0.0018 | 0.00310732 | ** | 111.47 | 0.0001 |
| 207 | 211 | 24.527 | 0.0023 | 0.00310732 | ** | 111.47 | 0.0001 |
| 207 | 213 | 26.819 | 0.0022 | 0.00310732 | ** | 111.47 | 0.0001 |
| 207 | 338 | 72.959 | 0.002 | 0.00310732 | ** | 111.47 | 0.0001 |
| 207 | 42_P10 | 67.123 | 0.0021 | 0.00310732 | ** | 111.47 | 0.0001 |
| 207 | 7 | 52.694 | 0.002 | 0.00310732 | ** | 111.47 | 0.0001 |
| 207 | Control Sawdust | 244.679 | 0.002 | 0.00310732 | ** | 111.47 | 0.0001 |
| 207 | P311 | 17.626 | 0.0024 | 0.00310732 | ** | 111.47 | 0.0001 |
| 209 | 21 | 73.741 | 0.0016 | 0.00310732 | ** | 111.47 | 0.0001 |
| 209 | 211 | 18.864 | 0.0017 | 0.00310732 | ** | 111.47 | 0.0001 |
| 209 | 213 | 41.258 | 0.0026 | 0.00310732 | ** | 111.47 | 0.0001 |
| 209 | 338 | 160.24 | 0.0027 | 0.00310732 | ** | 111.47 | 0.0001 |
| 209 | 42_P10 | 98.734 | 0.0022 | 0.00310732 | ** | 111.47 | 0.0001 |
| 209 | 7 | 104.028 | 0.0022 | 0.00310732 | ** | 111.47 | 0.0001 |
| 209 | Control Sawdust | 337.161 | 0.002 | 0.00310732 | ** | 111.47 | 0.0001 |
| 209 | P311 | 43.192 | 0.0027 | 0.00310732 | ** | 111.47 | 0.0001 |
| 21 | 211 | 107.909 | 0.0022 | 0.00310732 | ** | 111.47 | 0.0001 |
| 21 | 213 | 74.49 | 0.0028 | 0.00310732 | ** | 111.47 | 0.0001 |
| 21 | 338 | 86.368 | 0.0026 | 0.00310732 | ** | 111.47 | 0.0001 |
| 21 | 42_P10 | 311.896 | 0.0031 | 0.00324253 | ** | 111.47 | 0.0001 |
| 21 | 7 | 117.669 | 0.002 | 0.00310732 | ** | 111.47 | 0.0001 |
| 21 | Control Sawdust | 656.206 | 0.0017 | 0.00310732 | ** | 111.47 | 0.0001 |
| 21 | P311 | 40.961 | 0.0016 | 0.00310732 | ** | 111.47 | 0.0001 |
| 211 | 213 | 50.012 | 0.0021 | 0.00310732 | ** | 111.47 | 0.0001 |
| 211 | 338 | 233.815 | 0.0027 | 0.00310732 | ** | 111.47 | 0.0001 |
| 211 | 42_P10 | 110.487 | 0.0031 | 0.00324253 | ** | 111.47 | 0.0001 |
| 211 | 7 | 134.373 | 0.0016 | 0.00310732 | ** | 111.47 | 0.0001 |
| 211 | Control Sawdust | 371.094 | 0.0029 | 0.00314167 | ** | 111.47 | 0.0001 |
| 211 | P311 | 46.732 | 0.0033 | 0.00337416 | ** | 111.47 | 0.0001 |
| 213 | 338 | 155.969 | 0.0022 | 0.00310732 | ** | 111.47 | 0.0001 |
| 213 | 42_P10 | 56.534 | 0.0021 | 0.00310732 | ** | 111.47 | 0.0001 |
| 213 | 7 | 158.518 | 0.0028 | 0.00310732 | ** | 111.47 | 0.0001 |
| 213 | Control Sawdust | 513.796 | 0.0023 | 0.00310732 | ** | 111.47 | 0.0001 |
| 213 | P311 | 56.819 | 0.0023 | 0.00310732 | ** | 111.47 | 0.0001 |
| 338 | 42_P10 | 408.083 | 0.0025 | 0.00310732 | ** | 111.47 | 0.0001 |
| 338 | 7 | 85.111 | 0.0018 | 0.00310732 | ** | 111.47 | 0.0001 |
| 338 | Control Sawdust | 571.086 | 0.0022 | 0.00310732 | ** | 111.47 | 0.0001 |
| 338 | P311 | 112.853 | 0.0015 | 0.00310732 | ** | 111.47 | 0.0001 |
| 42_P10 | 7 | 529.694 | 0.0018 | 0.00310732 | ** | 111.47 | 0.0001 |
| 42_P10 | Control Sawdust | 900.259 | 0.0027 | 0.00310732 | ** | 111.47 | 0.0001 |
| 42_P10 | P311 | 133.556 | 0.0024 | 0.00310732 | ** | 111.47 | 0.0001 |
| 7 | Control Sawdust | 620.772 | 0.0021 | 0.00310732 | ** | 111.47 | 0.0001 |
| 7 | P311 | 78.855 | 0.0024 | 0.00310732 | ** | 111.47 | 0.0001 |
| Control Sawdust | P311 | 363.806 | 0.0022 | 0.00310732 | ** | 111.47 | 0.0001 |

**Suppl. Fig. S2**

**Free amino acids (S2a)**

| Info | Value |
| --- | --- |
| Best model family | gaussian |
| Model name | Normal_log |

| Effect | Df | Deviance | Resid. Df | Resid. Dev | Pr(>Chi) |
| --- | --- | --- | --- | --- | --- |
| NULL |  |  | 82 | 160.3912 |  |
| Sample | 13 | 155.6644 | 69 | 4.726872 | 0 |

| Comparison | Estimate | SE | df | t.ratio | p_value | Significance |
| --- | --- | --- | --- | --- | --- | --- |
| 1_167 - 1a_159 | 1.725856 | 0.158489 | 69 | 10.88947 | 0 | *** |
| 1_167 - 2_2 | 1.675277 | 0.158489 | 69 | 10.57033 | 0 | *** |
| 1_167 - 3_10 | 0.344542 | 0.158489 | 69 | 2.173925 | 0.64865 |  |
| 1_167 - 4_331 | 2.062533 | 0.158489 | 69 | 13.01376 | 0 | *** |
| 1_167 - 5_188 | 1.051639 | 0.158489 | 69 | 6.63542 | 5.33E-07 | *** |
| 1_167 - 6_211 | 1.585167 | 0.158489 | 69 | 10.00177 | 0 | *** |
| 1_167 - 7_209 | 1.591942 | 0.158489 | 69 | 10.04452 | 0 | *** |
| 1_167 - 8_213 | 0.696312 | 0.158489 | 69 | 4.393452 | 0.002917 | ** |
| 1_167 - 9_207 | 1.816905 | 0.158489 | 69 | 11.46395 | 0 | *** |
| 1_167 - 91_7 | 3.007632 | 0.158489 | 69 | 18.97696 | 0 | *** |
| 1_167 - 92_338 | 2.592174 | 0.158489 | 69 | 16.35558 | 0 | *** |
| 1_167 - 93_21 | 1.829298 | 0.158489 | 69 | 11.54214 | 0 | *** |
| 1_167 - Control Sawdust | 5.942035 | 0.158489 | 69 | 37.49188 | 0 | *** |
| 1a_159 - 2_2 | -0.05058 | 0.151113 | 69 | -0.33471 | 1 |  |
| 1a_159 - 3_10 | -1.38131 | 0.151113 | 69 | -9.14094 | 0 | *** |
| 1a_159 - 4_331 | 0.336676 | 0.151113 | 69 | 2.227977 | 0.611164 |  |
| 1a_159 - 5_188 | -0.67422 | 0.151113 | 69 | -4.46168 | 0.002309 | ** |
| 1a_159 - 6_211 | -0.14069 | 0.151113 | 69 | -0.93102 | 0.99958 |  |
| 1a_159 - 7_209 | -0.13391 | 0.151113 | 69 | -0.88618 | 0.999753 |  |
| 1a_159 - 8_213 | -1.02954 | 0.151113 | 69 | -6.81308 | 2.56E-07 | *** |
| 1a_159 - 9_207 | 0.091049 | 0.151113 | 69 | 0.60252 | 0.999997 |  |
| 1a_159 - 91_7 | 1.281776 | 0.151113 | 69 | 8.482238 | 1.89E-10 | *** |
| 1a_159 - 92_338 | 0.866317 | 0.151113 | 69 | 5.732911 | 2.03E-05 | *** |
| 1a_159 - 93_21 | 0.103442 | 0.151113 | 69 | 0.684532 | 0.999987 |  |
| 1a_159 - Control Sawdust | 4.216179 | 0.151113 | 69 | 27.90084 | 0 | *** |
| 2_2 - 3_10 | -1.33073 | 0.151113 | 69 | -8.80622 | 1.33E-11 | *** |
| 2_2 - 4_331 | 0.387256 | 0.151113 | 69 | 2.562689 | 0.382845 |  |
| 2_2 - 5_188 | -0.62364 | 0.151113 | 69 | -4.12697 | 0.007079 | ** |
| 2_2 - 6_211 | -0.09011 | 0.151113 | 69 | -0.59631 | 0.999997 |  |
| 2_2 - 7_209 | -0.08333 | 0.151113 | 69 | -0.55147 | 0.999999 |  |
| 2_2 - 8_213 | -0.97897 | 0.151113 | 69 | -6.47837 | 1.02E-06 | *** |
| 2_2 - 9_207 | 0.141628 | 0.151113 | 69 | 0.937232 | 0.999549 |  |
| 2_2 - 91_7 | 1.332355 | 0.151113 | 69 | 8.816949 | 1.07E-11 | *** |
| 2_2 - 92_338 | 0.916896 | 0.151113 | 69 | 6.067623 | 5.37E-06 | *** |
| 2_2 - 93_21 | 0.154021 | 0.151113 | 69 | 1.019243 | 0.998916 |  |
| 2_2 - Control Sawdust | 4.266758 | 0.151113 | 69 | 28.23555 | 0 | *** |
| 3_10 - 4_331 | 1.71799 | 0.151113 | 69 | 11.36891 | 0 | *** |
| 3_10 - 5_188 | 0.707096 | 0.151113 | 69 | 4.679255 | 0.001078 | ** |
| 3_10 - 6_211 | 1.240625 | 0.151113 | 69 | 8.209914 | 6.96E-10 | *** |
| 3_10 - 7_209 | 1.2474 | 0.151113 | 69 | 8.254752 | 5.68E-10 | *** |
| 3_10 - 8_213 | 0.35177 | 0.151113 | 69 | 2.327859 | 0.541065 |  |
| 3_10 - 9_207 | 1.472363 | 0.151113 | 69 | 9.743457 | 0 | *** |
| 3_10 - 91_7 | 2.66309 | 0.151113 | 69 | 17.62317 | 0 | *** |
| 3_10 - 92_338 | 2.247631 | 0.151113 | 69 | 14.87385 | 0 | *** |
| 3_10 - 93_21 | 1.484756 | 0.151113 | 69 | 9.825468 | 0 | *** |
| 3_10 - Control Sawdust | 5.597493 | 0.151113 | 69 | 37.04178 | 0 | *** |
| 4_331 - 5_188 | -1.01089 | 0.151113 | 69 | -6.68966 | 4.27E-07 | *** |
| 4_331 - 6_211 | -0.47737 | 0.151113 | 69 | -3.159 | 0.113109 |  |
| 4_331 - 7_209 | -0.47059 | 0.151113 | 69 | -3.11416 | 0.125869 |  |
| 4_331 - 8_213 | -1.36622 | 0.151113 | 69 | -9.04105 | 0 | *** |
| 4_331 - 9_207 | -0.24563 | 0.151113 | 69 | -1.62546 | 0.933065 |  |
| 4_331 - 91_7 | 0.9451 | 0.151113 | 69 | 6.25426 | 2.53E-06 | *** |
| 4_331 - 92_338 | 0.529641 | 0.151113 | 69 | 3.504934 | 0.046188 | * |
| 4_331 - 93_21 | -0.23323 | 0.151113 | 69 | -1.54345 | 0.954152 |  |
| 4_331 - Control Sawdust | 3.879503 | 0.151113 | 69 | 25.67287 | 0 | *** |
| 5_188 - 6_211 | 0.533528 | 0.151113 | 69 | 3.530659 | 0.043011 | * |
| 5_188 - 7_209 | 0.540304 | 0.151113 | 69 | 3.575497 | 0.037932 | * |
| 5_188 - 8_213 | -0.35533 | 0.151113 | 69 | -2.3514 | 0.524585 |  |
| 5_188 - 9_207 | 0.765266 | 0.151113 | 69 | 5.064201 | 0.000265 | *** |
| 5_188 - 91_7 | 1.955994 | 0.151113 | 69 | 12.94392 | 0 | *** |
| 5_188 - 92_338 | 1.540535 | 0.151113 | 69 | 10.19459 | 0 | *** |
| 5_188 - 93_21 | 0.777659 | 0.151113 | 69 | 5.146213 | 0.000195 | *** |
| 5_188 - Control Sawdust | 4.890397 | 0.151113 | 69 | 32.36252 | 0 | *** |
| 6_211 - 7_209 | 0.006776 | 0.151113 | 69 | 0.044838 | 1 |  |
| 6_211 - 8_213 | -0.88885 | 0.151113 | 69 | -5.88206 | 1.13E-05 | *** |
| 6_211 - 9_207 | 0.231738 | 0.151113 | 69 | 1.533542 | 0.956329 |  |
| 6_211 - 91_7 | 1.422466 | 0.151113 | 69 | 9.41326 | 0 | *** |
| 6_211 - 92_338 | 1.007007 | 0.151113 | 69 | 6.663933 | 4.74E-07 | *** |
| 6_211 - 93_21 | 0.244131 | 0.151113 | 69 | 1.615554 | 0.935911 |  |
| 6_211 - Control Sawdust | 4.356869 | 0.151113 | 69 | 28.83186 | 0 | *** |
| 7_209 - 8_213 | -0.89563 | 0.151113 | 69 | -5.92689 | 9.43E-06 | *** |
| 7_209 - 9_207 | 0.224963 | 0.151113 | 69 | 1.488704 | 0.965257 |  |
| 7_209 - 91_7 | 1.41569 | 0.151113 | 69 | 9.368422 | 0 | *** |
| 7_209 - 92_338 | 1.000231 | 0.151113 | 69 | 6.619095 | 5.7E-07 | *** |
| 7_209 - 93_21 | 0.237355 | 0.151113 | 69 | 1.570715 | 0.947756 |  |
| 7_209 - Control Sawdust | 4.350093 | 0.151113 | 69 | 28.78703 | 0 | *** |
| 8_213 - 9_207 | 1.120593 | 0.151113 | 69 | 7.415597 | 2.08E-08 | *** |
| 8_213 - 91_7 | 2.31132 | 0.151113 | 69 | 15.29531 | 0 | *** |
| 8_213 - 92_338 | 1.895861 | 0.151113 | 69 | 12.54599 | 0 | *** |
| 8_213 - 93_21 | 1.132986 | 0.151113 | 69 | 7.497609 | 1.48E-08 | *** |
| 8_213 - Control Sawdust | 5.245723 | 0.151113 | 69 | 34.71392 | 0 | *** |
| 9_207 - 91_7 | 1.190727 | 0.151113 | 69 | 7.879717 | 2.93E-09 | *** |
| 9_207 - 92_338 | 0.775269 | 0.151113 | 69 | 5.130391 | 0.000207 | *** |
| 9_207 - 93_21 | 0.012393 | 0.151113 | 69 | 0.082011 | 1 |  |
| 9_207 - Control Sawdust | 4.12513 | 0.151113 | 69 | 27.29832 | 0 | *** |
| 91_7 - 92_338 | -0.41546 | 0.151113 | 69 | -2.74933 | 0.274718 |  |
| 91_7 - 93_21 | -1.17833 | 0.151113 | 69 | -7.79771 | 4.16E-09 | *** |
| 91_7 - Control Sawdust | 2.934403 | 0.151113 | 69 | 19.4186 | 0 | *** |
| 92_338 - 93_21 | -0.76288 | 0.151113 | 69 | -5.04838 | 0.000281 | *** |
| 92_338 - Control Sawdust | 3.349862 | 0.151113 | 69 | 22.16793 | 0 | *** |
| 93_21 - Control Sawdust | 4.112738 | 0.151113 | 69 | 27.21631 | 0 | *** |

| Sample | mean_Value | SE | df | lower.CL | upper.CL | Group |
| --- | --- | --- | --- | --- | --- | --- |
| Control Sawdust | 0.735852 | 0.106853 | 69 | 0.533525 | 1.014905 | a |
| 91_7 | 13.84157 | 0.106853 | 69 | 10.03575 | 19.09065 | b |
| 92_338 | 20.97088 | 0.106853 | 69 | 15.20482 | 28.92359 | b |
| 4_331 | 35.61532 | 0.106853 | 69 | 25.82269 | 49.12157 | c |
| 93_21 | 44.97067 | 0.106853 | 69 | 32.60573 | 62.02472 | c |
| 9_207 | 45.53146 | 0.106853 | 69 | 33.01233 | 62.79817 | c |
| 1a_159 | 49.87162 | 0.106853 | 69 | 36.15914 | 68.78424 | c |
| 2_2 | 52.45897 | 0.106853 | 69 | 38.03508 | 72.35278 | c |
| 7_209 | 57.01795 | 0.106853 | 69 | 41.34054 | 78.64063 | c |
| 6_211 | 57.40559 | 0.106853 | 69 | 41.6216 | 79.17528 | c |
| 5_188 | 97.87293 | 0.106853 | 69 | 70.96222 | 134.9889 | d |
| 8_213 | 139.6301 | 0.106853 | 69 | 101.238 | 192.5814 | de |
| 3_10 | 198.4954 | 0.106853 | 69 | 143.918 | 273.7701 | ef |
| 1_167 | 280.1453 | 0.117052 | 69 | 196.9792 | 398.4248 | f |

**Soluble sugars (Suppl. Fig. S2b)**

| Info | Value |
| --- | --- |
| Best model family | gaussian |
| Model name | Sqrt_Normal |

| Effect | Df | Deviance | Resid. Df | Resid. Dev | Pr(>Chi) |
| --- | --- | --- | --- | --- | --- |
| NULL |  |  | 82 | 454.3437 |  |
| Sample | 13 | 433.1081 | 69 | 21.23558 | 4.1E-293 |

| Comparison | Estimate | SE | df | t.ratio | p_value | Significance |
| --- | --- | --- | --- | --- | --- | --- |
| 1_167 - 1a_159 | 4.891764 | 0.335926 | 69 | 14.56204 | 0 | *** |
| 1_167 - 2_2 | 1.403389 | 0.335926 | 69 | 4.177678 | 0.006001 | ** |
| 1_167 - 3_10 | -4.02784 | 0.335926 | 69 | -11.9903 | 0 | *** |
| 1_167 - 4_331 | 4.185434 | 0.335926 | 69 | 12.4594 | 0 | *** |
| 1_167 - 5_188 | 3.250766 | 0.335926 | 69 | 9.677037 | 0 | *** |
| 1_167 - 6_211 | 2.722335 | 0.335926 | 69 | 8.103978 | 1.11E-09 | *** |
| 1_167 - 7_209 | 1.758882 | 0.335926 | 69 | 5.235927 | 0.000139 | *** |
| 1_167 - 8_213 | -0.79782 | 0.335926 | 69 | -2.37498 | 0.508144 |  |
| 1_167 - 9_207 | 2.373105 | 0.335926 | 69 | 7.064373 | 9.03E-08 | *** |
| 1_167 - 91_7 | 2.424623 | 0.335926 | 69 | 7.217736 | 4.77E-08 | *** |
| 1_167 - 92_338 | 1.719653 | 0.335926 | 69 | 5.119147 | 0.000216 | *** |
| 1_167 - 93_21 | 1.509977 | 0.335926 | 69 | 4.494974 | 0.002058 | ** |
| 1_167 - Control Sawdust | 4.922284 | 0.335926 | 69 | 14.65289 | 0 | *** |
| 1a_159 - 2_2 | -3.48837 | 0.320293 | 69 | -10.8912 | 0 | *** |
| 1a_159 - 3_10 | -8.9196 | 0.320293 | 69 | -27.8483 | 0 | *** |
| 1a_159 - 4_331 | -0.70633 | 0.320293 | 69 | -2.20526 | 0.626987 |  |
| 1a_159 - 5_188 | -1.641 | 0.320293 | 69 | -5.12343 | 0.000212 | *** |
| 1a_159 - 6_211 | -2.16943 | 0.320293 | 69 | -6.77327 | 3.02E-07 | *** |
| 1a_159 - 7_209 | -3.13288 | 0.320293 | 69 | -9.78131 | 0 | *** |
| 1a_159 - 8_213 | -5.68958 | 0.320293 | 69 | -17.7637 | 0 | *** |
| 1a_159 - 9_207 | -2.51866 | 0.320293 | 69 | -7.86362 | 3.14E-09 | *** |
| 1a_159 - 91_7 | -2.46714 | 0.320293 | 69 | -7.70277 | 6.22E-09 | *** |
| 1a_159 - 92_338 | -3.17211 | 0.320293 | 69 | -9.90379 | 0 | *** |
| 1a_159 - 93_21 | -3.38179 | 0.320293 | 69 | -10.5584 | 0 | *** |
| 1a_159 - Control Sawdust | 0.03052 | 0.320293 | 69 | 0.095289 | 1 |  |
| 2_2 - 3_10 | -5.43122 | 0.320293 | 69 | -16.9571 | 0 | *** |
| 2_2 - 4_331 | 2.782044 | 0.320293 | 69 | 8.685946 | 5.32E-11 | *** |
| 2_2 - 5_188 | 1.847376 | 0.320293 | 69 | 5.767777 | 1.77E-05 | *** |
| 2_2 - 6_211 | 1.318945 | 0.320293 | 69 | 4.117939 | 0.00729 | ** |
| 2_2 - 7_209 | 0.355493 | 0.320293 | 69 | 1.109901 | 0.997447 |  |
| 2_2 - 8_213 | -2.20121 | 0.320293 | 69 | -6.87249 | 2E-07 | *** |
| 2_2 - 9_207 | 0.969715 | 0.320293 | 69 | 3.027591 | 0.15372 |  |
| 2_2 - 91_7 | 1.021234 | 0.320293 | 69 | 3.18844 | 0.105315 |  |
| 2_2 - 92_338 | 0.316264 | 0.320293 | 69 | 0.987421 | 0.999219 |  |
| 2_2 - 93_21 | 0.106588 | 0.320293 | 69 | 0.332783 | 1 |  |
| 2_2 - Control Sawdust | 3.518895 | 0.320293 | 69 | 10.9865 | 0 | *** |
| 3_10 - 4_331 | 8.213269 | 0.320293 | 69 | 25.64302 | 0 | *** |
| 3_10 - 5_188 | 7.278601 | 0.320293 | 69 | 22.72485 | 0 | *** |
| 3_10 - 6_211 | 6.75017 | 0.320293 | 69 | 21.07501 | 0 | *** |
| 3_10 - 7_209 | 5.786718 | 0.320293 | 69 | 18.06697 | 0 | *** |
| 3_10 - 8_213 | 3.230019 | 0.320293 | 69 | 10.08459 | 0 | *** |
| 3_10 - 9_207 | 6.40094 | 0.320293 | 69 | 19.98466 | 0 | *** |
| 3_10 - 91_7 | 6.452459 | 0.320293 | 69 | 20.14551 | 0 | *** |
| 3_10 - 92_338 | 5.747489 | 0.320293 | 69 | 17.94449 | 0 | *** |
| 3_10 - 93_21 | 5.537813 | 0.320293 | 69 | 17.28986 | 0 | *** |
| 3_10 - Control Sawdust | 8.95012 | 0.320293 | 69 | 27.94357 | 0 | *** |
| 4_331 - 5_188 | -0.93467 | 0.320293 | 69 | -2.91817 | 0.195404 |  |
| 4_331 - 6_211 | -1.4631 | 0.320293 | 69 | -4.56801 | 0.001596 | ** |
| 4_331 - 7_209 | -2.42655 | 0.320293 | 69 | -7.57605 | 1.06E-08 | *** |
| 4_331 - 8_213 | -4.98325 | 0.320293 | 69 | -15.5584 | 0 | *** |
| 4_331 - 9_207 | -1.81233 | 0.320293 | 69 | -5.65835 | 2.73E-05 | *** |
| 4_331 - 91_7 | -1.76081 | 0.320293 | 69 | -5.49751 | 5.1E-05 | *** |
| 4_331 - 92_338 | -2.46578 | 0.320293 | 69 | -7.69852 | 6.33E-09 | *** |
| 4_331 - 93_21 | -2.67546 | 0.320293 | 69 | -8.35316 | 3.6E-10 | *** |
| 4_331 - Control Sawdust | 0.73685 | 0.320293 | 69 | 2.300554 | 0.56024 |  |
| 5_188 - 6_211 | -0.52843 | 0.320293 | 69 | -1.64984 | 0.925693 |  |
| 5_188 - 7_209 | -1.49188 | 0.320293 | 69 | -4.65788 | 0.001163 | ** |
| 5_188 - 8_213 | -4.04858 | 0.320293 | 69 | -12.6403 | 0 | *** |
| 5_188 - 9_207 | -0.87766 | 0.320293 | 69 | -2.74019 | 0.279533 |  |
| 5_188 - 91_7 | -0.82614 | 0.320293 | 69 | -2.57934 | 0.372419 |  |
| 5_188 - 92_338 | -1.53111 | 0.320293 | 69 | -4.78036 | 0.00075 | *** |
| 5_188 - 93_21 | -1.74079 | 0.320293 | 69 | -5.43499 | 6.5E-05 | *** |
| 5_188 - Control Sawdust | 1.671518 | 0.320293 | 69 | 5.218723 | 0.000148 | *** |
| 6_211 - 7_209 | -0.96345 | 0.320293 | 69 | -3.00804 | 0.160625 |  |
| 6_211 - 8_213 | -3.52015 | 0.320293 | 69 | -10.9904 | 0 | *** |
| 6_211 - 9_207 | -0.34923 | 0.320293 | 69 | -1.09035 | 0.997858 |  |
| 6_211 - 91_7 | -0.29771 | 0.320293 | 69 | -0.9295 | 0.999588 |  |
| 6_211 - 92_338 | -1.00268 | 0.320293 | 69 | -3.13052 | 0.121087 |  |
| 6_211 - 93_21 | -1.21236 | 0.320293 | 69 | -3.78516 | 0.020601 | * |
| 6_211 - Control Sawdust | 2.19995 | 0.320293 | 69 | 6.868561 | 2.04E-07 | *** |
| 7_209 - 8_213 | -2.5567 | 0.320293 | 69 | -7.98239 | 1.89E-09 | *** |
| 7_209 - 9_207 | 0.614222 | 0.320293 | 69 | 1.91769 | 0.809746 |  |
| 7_209 - 91_7 | 0.665741 | 0.320293 | 69 | 2.078539 | 0.71266 |  |
| 7_209 - 92_338 | -0.03923 | 0.320293 | 69 | -0.12248 | 1 |  |
| 7_209 - 93_21 | -0.24891 | 0.320293 | 69 | -0.77712 | 0.999943 |  |
| 7_209 - Control Sawdust | 3.163402 | 0.320293 | 69 | 9.876599 | 0 | *** |
| 8_213 - 9_207 | 3.170922 | 0.320293 | 69 | 9.900077 | 0 | *** |
| 8_213 - 91_7 | 3.22244 | 0.320293 | 69 | 10.06093 | 0 | *** |
| 8_213 - 92_338 | 2.51747 | 0.320293 | 69 | 7.859907 | 3.19E-09 | *** |
| 8_213 - 93_21 | 2.307794 | 0.320293 | 69 | 7.205269 | 5.02E-08 | *** |
| 8_213 - Control Sawdust | 5.720101 | 0.320293 | 69 | 17.85899 | 0 | *** |
| 9_207 - 91_7 | 0.051519 | 0.320293 | 69 | 0.160849 | 1 |  |
| 9_207 - 92_338 | -0.65345 | 0.320293 | 69 | -2.04017 | 0.737273 |  |
| 9_207 - 93_21 | -0.86313 | 0.320293 | 69 | -2.69481 | 0.304203 |  |
| 9_207 - Control Sawdust | 2.54918 | 0.320293 | 69 | 7.958909 | 2.09E-09 | *** |
| 91_7 - 92_338 | -0.70497 | 0.320293 | 69 | -2.20102 | 0.629935 |  |
| 91_7 - 93_21 | -0.91465 | 0.320293 | 69 | -2.85566 | 0.222626 |  |
| 91_7 - Control Sawdust | 2.497661 | 0.320293 | 69 | 7.79806 | 4.15E-09 | *** |
| 92_338 - 93_21 | -0.20968 | 0.320293 | 69 | -0.65464 | 0.999992 |  |
| 92_338 - Control Sawdust | 3.202631 | 0.320293 | 69 | 9.999079 | 0 | *** |
| 93_21 - Control Sawdust | 3.412307 | 0.320293 | 69 | 10.65372 | 0 | *** |

| Sample | mean_Value | SE | df | lower.CL | upper.CL | Group |
| --- | --- | --- | --- | --- | --- | --- |
| Control Sawdust | 1.960261 | 0.226481 | 69 | 1.278775 | 2.641746 | a |
| 1a_159 | 1.990781 | 0.226481 | 69 | 1.309296 | 2.672266 | a |
| 4_331 | 2.697111 | 0.226481 | 69 | 2.015626 | 3.378596 | ab |
| 5_188 | 3.631779 | 0.226481 | 69 | 2.950294 | 4.313264 | bc |
| 6_211 | 4.16021 | 0.226481 | 69 | 3.478725 | 4.841695 | cd |
| 91_7 | 4.457922 | 0.226481 | 69 | 3.776436 | 5.139407 | cde |
| 9_207 | 4.50944 | 0.226481 | 69 | 3.827955 | 5.190926 | cde |
| 7_209 | 5.123662 | 0.226481 | 69 | 4.442177 | 5.805148 | de |
| 92_338 | 5.162892 | 0.226481 | 69 | 4.481406 | 5.844377 | de |
| 93_21 | 5.372567 | 0.226481 | 69 | 4.691082 | 6.054053 | e |
| 2_2 | 5.479156 | 0.226481 | 69 | 4.79767 | 6.160641 | e |
| 1_167 | 6.882545 | 0.248098 | 69 | 6.136015 | 7.629075 | f |
| 8_213 | 7.680362 | 0.226481 | 69 | 6.998877 | 8.361847 | f |
| 3_10 | 10.91038 | 0.226481 | 69 | 10.2289 | 11.59187 | g |

**B vitamins (Suppl. Fig. S2c)**

| Info | Value |
| --- | --- |
| Best model family | Gamma |
| Model name | Gamma_log |

| Effect | Df | Deviance | Resid. Df | Resid. Dev | Pr(>Chi) |
| --- | --- | --- | --- | --- | --- |
| NULL |  |  | 82 | 71.12073 |  |
| Sample | 13 | 66.29986 | 69 | 4.820866 | 2E-210 |

| Comparison | Estimate | SE | df | t.ratio | p_value | Significance |
| --- | --- | --- | --- | --- | --- | --- |
| 1_167 - 1a_159 | 1.648332 | 0.154156 | 69 | 10.6926 | 0 | *** |
| 1_167 - 2_2 | 1.061771 | 0.154156 | 69 | 6.887622 | 1.88E-07 | *** |
| 1_167 - 3_10 | 0.498759 | 0.154156 | 69 | 3.235408 | 0.093795 |  |
| 1_167 - 4_331 | 1.111316 | 0.154156 | 69 | 7.209016 | 4.94E-08 | *** |
| 1_167 - 5_188 | 1.103173 | 0.154156 | 69 | 7.156192 | 6.16E-08 | *** |
| 1_167 - 6_211 | 0.677194 | 0.154156 | 69 | 4.392901 | 0.002922 | ** |
| 1_167 - 7_209 | 1.802483 | 0.154156 | 69 | 11.69256 | 0 | *** |
| 1_167 - 8_213 | 0.814631 | 0.154156 | 69 | 5.284445 | 0.000116 | *** |
| 1_167 - 9_207 | 0.110859 | 0.154156 | 69 | 0.719135 | 0.999976 |  |
| 1_167 - 91_7 | 1.476237 | 0.154156 | 69 | 9.576226 | 0 | *** |
| 1_167 - 92_338 | 3.291833 | 0.154156 | 69 | 21.35385 | 0 | *** |
| 1_167 - 93_21 | 1.723125 | 0.154156 | 69 | 11.17777 | 0 | *** |
| 1_167 - Control Sawdust | 3.971908 | 0.154156 | 69 | 25.76544 | 0 | *** |
| 1a_159 - 2_2 | -0.58656 | 0.146982 | 69 | -3.99069 | 0.010947 | * |
| 1a_159 - 3_10 | -1.14957 | 0.146982 | 69 | -7.82116 | 3.76E-09 | *** |
| 1a_159 - 4_331 | -0.53702 | 0.146982 | 69 | -3.65361 | 0.030346 | * |
| 1a_159 - 5_188 | -0.54516 | 0.146982 | 69 | -3.70901 | 0.025823 | * |
| 1a_159 - 6_211 | -0.97114 | 0.146982 | 69 | -6.60718 | 5.99E-07 | *** |
| 1a_159 - 7_209 | 0.154151 | 0.146982 | 69 | 1.04877 | 0.99855 |  |
| 1a_159 - 8_213 | -0.8337 | 0.146982 | 69 | -5.67212 | 2.58E-05 | *** |
| 1a_159 - 9_207 | -1.53747 | 0.146982 | 69 | -10.4603 | 0 | *** |
| 1a_159 - 91_7 | -0.1721 | 0.146982 | 69 | -1.17086 | 0.995718 |  |
| 1a_159 - 92_338 | 1.6435 | 0.146982 | 69 | 11.18161 | 0 | *** |
| 1a_159 - 93_21 | 0.074793 | 0.146982 | 69 | 0.508857 | 1 |  |
| 1a_159 - Control Sawdust | 2.323575 | 0.146982 | 69 | 15.80853 | 0 | *** |
| 2_2 - 3_10 | -0.56301 | 0.146982 | 69 | -3.83047 | 0.01797 | * |
| 2_2 - 4_331 | 0.049545 | 0.146982 | 69 | 0.337081 | 1 |  |
| 2_2 - 5_188 | 0.041402 | 0.146982 | 69 | 0.281679 | 1 |  |
| 2_2 - 6_211 | -0.38458 | 0.146982 | 69 | -2.61649 | 0.349667 |  |
| 2_2 - 7_209 | 0.740712 | 0.146982 | 69 | 5.039461 | 0.000291 | *** |
| 2_2 - 8_213 | -0.24714 | 0.146982 | 69 | -1.68143 | 0.91536 |  |
| 2_2 - 9_207 | -0.95091 | 0.146982 | 69 | -6.46956 | 1.05E-06 | *** |
| 2_2 - 91_7 | 0.414466 | 0.146982 | 69 | 2.819832 | 0.239365 |  |
| 2_2 - 92_338 | 2.230062 | 0.146982 | 69 | 15.1723 | 0 | *** |
| 2_2 - 93_21 | 0.661354 | 0.146982 | 69 | 4.499548 | 0.002026 | ** |
| 2_2 - Control Sawdust | 2.910137 | 0.146982 | 69 | 19.79922 | 0 | *** |
| 3_10 - 4_331 | 0.612557 | 0.146982 | 69 | 4.167555 | 0.006203 | ** |
| 3_10 - 5_188 | 0.604414 | 0.146982 | 69 | 4.112152 | 0.007428 | ** |
| 3_10 - 6_211 | 0.178435 | 0.146982 | 69 | 1.213989 | 0.993981 |  |
| 3_10 - 7_209 | 1.303724 | 0.146982 | 69 | 8.869934 | 0 | *** |
| 3_10 - 8_213 | 0.315872 | 0.146982 | 69 | 2.149047 | 0.665661 |  |
| 3_10 - 9_207 | -0.3879 | 0.146982 | 69 | -2.63909 | 0.336185 |  |
| 3_10 - 91_7 | 0.977478 | 0.146982 | 69 | 6.650306 | 5.02E-07 | *** |
| 3_10 - 92_338 | 2.793074 | 0.146982 | 69 | 19.00278 | 0 | *** |
| 3_10 - 93_21 | 1.224366 | 0.146982 | 69 | 8.330021 | 4.01E-10 | *** |
| 3_10 - Control Sawdust | 3.473149 | 0.146982 | 69 | 23.62969 | 0 | *** |
| 4_331 - 5_188 | -0.00814 | 0.146982 | 69 | -0.0554 | 1 |  |
| 4_331 - 6_211 | -0.43412 | 0.146982 | 69 | -2.95357 | 0.1811 |  |
| 4_331 - 7_209 | 0.691167 | 0.146982 | 69 | 4.702379 | 0.000992 | *** |
| 4_331 - 8_213 | -0.29669 | 0.146982 | 69 | -2.01851 | 0.750808 |  |
| 4_331 - 9_207 | -1.00046 | 0.146982 | 69 | -6.80665 | 2.63E-07 | *** |
| 4_331 - 91_7 | 0.364921 | 0.146982 | 69 | 2.482751 | 0.434672 |  |
| 4_331 - 92_338 | 2.180517 | 0.146982 | 69 | 14.83522 | 0 | *** |
| 4_331 - 93_21 | 0.611809 | 0.146982 | 69 | 4.162467 | 0.006307 | ** |
| 4_331 - Control Sawdust | 2.860592 | 0.146982 | 69 | 19.46214 | 0 | *** |
| 5_188 - 6_211 | -0.42598 | 0.146982 | 69 | -2.89816 | 0.203842 |  |
| 5_188 - 7_209 | 0.69931 | 0.146982 | 69 | 4.757782 | 0.000814 | *** |
| 5_188 - 8_213 | -0.28854 | 0.146982 | 69 | -1.9631 | 0.78408 |  |
| 5_188 - 9_207 | -0.99231 | 0.146982 | 69 | -6.75124 | 3.31E-07 | *** |
| 5_188 - 91_7 | 0.373064 | 0.146982 | 69 | 2.538153 | 0.398454 |  |
| 5_188 - 92_338 | 2.18866 | 0.146982 | 69 | 14.89063 | 0 | *** |
| 5_188 - 93_21 | 0.619952 | 0.146982 | 69 | 4.217869 | 0.005257 | ** |
| 5_188 - Control Sawdust | 2.868735 | 0.146982 | 69 | 19.51754 | 0 | *** |
| 6_211 - 7_209 | 1.125289 | 0.146982 | 69 | 7.655946 | 7.58E-09 | *** |
| 6_211 - 8_213 | 0.137437 | 0.146982 | 69 | 0.935059 | 0.99956 |  |
| 6_211 - 9_207 | -0.56633 | 0.146982 | 69 | -3.85308 | 0.016775 | * |
| 6_211 - 91_7 | 0.799043 | 0.146982 | 69 | 5.436317 | 6.47E-05 | *** |
| 6_211 - 92_338 | 2.614639 | 0.146982 | 69 | 17.78879 | 0 | *** |
| 6_211 - 93_21 | 1.045932 | 0.146982 | 69 | 7.116033 | 7.29E-08 | *** |
| 6_211 - Control Sawdust | 3.294714 | 0.146982 | 69 | 22.4157 | 0 | *** |
| 7_209 - 8_213 | -0.98785 | 0.146982 | 69 | -6.72089 | 3.75E-07 | *** |
| 7_209 - 9_207 | -1.69162 | 0.146982 | 69 | -11.509 | 0 | *** |
| 7_209 - 91_7 | -0.32625 | 0.146982 | 69 | -2.21963 | 0.61699 |  |
| 7_209 - 92_338 | 1.48935 | 0.146982 | 69 | 10.13284 | 0 | *** |
| 7_209 - 93_21 | -0.07936 | 0.146982 | 69 | -0.53991 | 0.999999 |  |
| 7_209 - Control Sawdust | 2.169425 | 0.146982 | 69 | 14.75976 | 0 | *** |
| 8_213 - 9_207 | -0.70377 | 0.146982 | 69 | -4.78814 | 0.00073 | *** |
| 8_213 - 91_7 | 0.661606 | 0.146982 | 69 | 4.501258 | 0.002014 | ** |
| 8_213 - 92_338 | 2.477202 | 0.146982 | 69 | 16.85373 | 0 | *** |
| 8_213 - 93_21 | 0.908494 | 0.146982 | 69 | 6.180974 | 3.4E-06 | *** |
| 8_213 - Control Sawdust | 3.157277 | 0.146982 | 69 | 21.48065 | 0 | *** |
| 9_207 - 91_7 | 1.365378 | 0.146982 | 69 | 9.289396 | 0 | *** |
| 9_207 - 92_338 | 3.180974 | 0.146982 | 69 | 21.64187 | 0 | *** |
| 9_207 - 93_21 | 1.612266 | 0.146982 | 69 | 10.96911 | 0 | *** |
| 9_207 - Control Sawdust | 3.861049 | 0.146982 | 69 | 26.26878 | 0 | *** |
| 91_7 - 92_338 | 1.815596 | 0.146982 | 69 | 12.35247 | 0 | *** |
| 91_7 - 93_21 | 0.246889 | 0.146982 | 69 | 1.679716 | 0.915943 |  |
| 91_7 - Control Sawdust | 2.495671 | 0.146982 | 69 | 16.97939 | 0 | *** |
| 92_338 - 93_21 | -1.56871 | 0.146982 | 69 | -10.6728 | 0 | *** |
| 92_338 - Control Sawdust | 0.680075 | 0.146982 | 69 | 4.626915 | 0.001297 | ** |
| 93_21 - Control Sawdust | 2.248782 | 0.146982 | 69 | 15.29967 | 0 | *** |

| Sample | mean_Value | SE | df | lower.CL | upper.CL | Group |
| --- | --- | --- | --- | --- | --- | --- |
| Control Sawdust | 1.348073 | 0.103932 | 69 | 1.035339 | 1.660807 | a |
| 92_338 | 2.028148 | 0.103932 | 69 | 1.715414 | 2.340882 | b |
| 7_209 | 3.517498 | 0.103932 | 69 | 3.204764 | 3.830232 | c |
| 93_21 | 3.596856 | 0.103932 | 69 | 3.284122 | 3.909589 | c |
| 1a_159 | 3.671649 | 0.103932 | 69 | 3.358915 | 3.984382 | c |
| 91_7 | 3.843744 | 0.103932 | 69 | 3.53101 | 4.156478 | cd |
| 4_331 | 4.208665 | 0.103932 | 69 | 3.895931 | 4.521399 | de |
| 5_188 | 4.216808 | 0.103932 | 69 | 3.904074 | 4.529542 | de |
| 2_2 | 4.25821 | 0.103932 | 69 | 3.945476 | 4.570944 | de |
| 8_213 | 4.50535 | 0.103932 | 69 | 4.192616 | 4.818084 | ef |
| 6_211 | 4.642787 | 0.103932 | 69 | 4.330053 | 4.955521 | ef |
| 3_10 | 4.821222 | 0.103932 | 69 | 4.508488 | 5.133956 | fg |
| 9_207 | 5.209122 | 0.103932 | 69 | 4.896388 | 5.521856 | g |
| 1_167 | 5.319981 | 0.113852 | 69 | 4.977398 | 5.662564 | g |

**Potassium (Suppl. Fig. S2d)**

| **Info** | **Value** |
| --- | --- |
| Best model family | gaussian |
| Model name | Sqrt_Normal |

| **Effect** | **Df** | **Deviance** | **Resid. Df** | **Resid. Dev** | **Pr(>Chi)** |
| --- | --- | --- | --- | --- | --- |
| NULL |  |  | 98 | 109399.7 |  |
| Sample | 13 | 105433.6 | 85 | 3966.133 | 0 |

| **Comparison** | **Estimate** | **SE** | **df** | **t.ratio** | **p_value** | **Significance** |
| --- | --- | --- | --- | --- | --- | --- |
| 1_167 - 1a_159 | 75.5497 | 3.651238 | 85 | 20.69153 | 2.08E-10 | *** |
| 1_167 - 2_2 | 68.02958 | 3.651238 | 85 | 18.63192 | 2.08E-10 | *** |
| 1_167 - 3_10 | 11.02482 | 3.248713 | 85 | 3.393596 | 0.058915 |  |
| 1_167 - 4_331 | 62.47566 | 3.651238 | 85 | 17.11082 | 2.08E-10 | *** |
| 1_167 - 5_188 | 73.23396 | 3.800329 | 85 | 19.27043 | 2.08E-10 | *** |
| 1_167 - 6_211 | 77.23698 | 3.651238 | 85 | 21.15364 | 2.08E-10 | *** |
| 1_167 - 7_209 | 83.80737 | 3.651238 | 85 | 22.95314 | 2.08E-10 | *** |
| 1_167 - 8_213 | 25.07175 | 3.800329 | 85 | 6.597257 | 3.02E-07 | *** |
| 1_167 - 9_207 | 74.71005 | 3.800329 | 85 | 19.65884 | 2.08E-10 | *** |
| 1_167 - 91_7 | 69.35386 | 3.800329 | 85 | 18.24944 | 2.08E-10 | *** |
| 1_167 - 92_338 | 92.32598 | 3.651238 | 85 | 25.28621 | 2.08E-10 | *** |
| 1_167 - 93_21 | 34.10197 | 3.651238 | 85 | 9.339839 | 2.09E-10 | *** |
| 1_167 - Control Sawdust | 114.9065 | 3.651238 | 85 | 31.47056 | 2.08E-10 | *** |
| 1a_159 - 2_2 | -7.52013 | 3.651238 | 85 | -2.05961 | 0.725647 |  |
| 1a_159 - 3_10 | -64.5249 | 3.248713 | 85 | -19.8617 | 2.08E-10 | *** |
| 1a_159 - 4_331 | -13.074 | 3.651238 | 85 | -3.58072 | 0.034653 | * |
| 1a_159 - 5_188 | -2.31574 | 3.800329 | 85 | -0.60935 | 0.999997 |  |
| 1a_159 - 6_211 | 1.687277 | 3.651238 | 85 | 0.462111 | 1 |  |
| 1a_159 - 7_209 | 8.257665 | 3.651238 | 85 | 2.261607 | 0.586916 |  |
| 1a_159 - 8_213 | -50.478 | 3.800329 | 85 | -13.2825 | 2.08E-10 | *** |
| 1a_159 - 9_207 | -0.83965 | 3.800329 | 85 | -0.22094 | 1 |  |
| 1a_159 - 91_7 | -6.19585 | 3.800329 | 85 | -1.63034 | 0.932866 |  |
| 1a_159 - 92_338 | 16.77627 | 3.651238 | 85 | 4.594681 | 0.00117 | ** |
| 1a_159 - 93_21 | -41.4477 | 3.651238 | 85 | -11.3517 | 2.08E-10 | *** |
| 1a_159 - Control Sawdust | 39.35678 | 3.651238 | 85 | 10.77902 | 2.08E-10 | *** |
| 2_2 - 3_10 | -57.0048 | 3.248713 | 85 | -17.5469 | 2.08E-10 | *** |
| 2_2 - 4_331 | -5.55392 | 3.651238 | 85 | -1.5211 | 0.959908 |  |
| 2_2 - 5_188 | 5.204387 | 3.800329 | 85 | 1.369457 | 0.98296 |  |
| 2_2 - 6_211 | 9.207406 | 3.651238 | 85 | 2.521722 | 0.406184 |  |
| 2_2 - 7_209 | 15.77779 | 3.651238 | 85 | 4.321218 | 0.003139 | ** |
| 2_2 - 8_213 | -42.9578 | 3.800329 | 85 | -11.3037 | 2.08E-10 | *** |
| 2_2 - 9_207 | 6.680477 | 3.800329 | 85 | 1.757868 | 0.888065 |  |
| 2_2 - 91_7 | 1.324284 | 3.800329 | 85 | 0.348466 | 1 |  |
| 2_2 - 92_338 | 24.2964 | 3.651238 | 85 | 6.654292 | 2.34E-07 | *** |
| 2_2 - 93_21 | -33.9276 | 3.651238 | 85 | -9.29208 | 2.1E-10 | *** |
| 2_2 - Control Sawdust | 46.87691 | 3.651238 | 85 | 12.83864 | 2.08E-10 | *** |
| 3_10 - 4_331 | 51.45084 | 3.248713 | 85 | 15.8373 | 2.08E-10 | *** |
| 3_10 - 5_188 | 62.20914 | 3.41542 | 85 | 18.2142 | 2.08E-10 | *** |
| 3_10 - 6_211 | 66.21216 | 3.248713 | 85 | 20.38105 | 2.08E-10 | *** |
| 3_10 - 7_209 | 72.78255 | 3.248713 | 85 | 22.4035 | 2.08E-10 | *** |
| 3_10 - 8_213 | 14.04693 | 3.41542 | 85 | 4.112796 | 0.006443 | ** |
| 3_10 - 9_207 | 63.68523 | 3.41542 | 85 | 18.64638 | 2.08E-10 | *** |
| 3_10 - 91_7 | 58.32904 | 3.41542 | 85 | 17.07814 | 2.08E-10 | *** |
| 3_10 - 92_338 | 81.30116 | 3.248713 | 85 | 25.02565 | 2.08E-10 | *** |
| 3_10 - 93_21 | 23.07715 | 3.248713 | 85 | 7.103476 | 3.14E-08 | *** |
| 3_10 - Control Sawdust | 103.8817 | 3.248713 | 85 | 31.97626 | 2.08E-10 | *** |
| 4_331 - 5_188 | 10.7583 | 3.800329 | 85 | 2.830887 | 0.229635 |  |
| 4_331 - 6_211 | 14.76132 | 3.651238 | 85 | 4.042827 | 0.008145 | ** |
| 4_331 - 7_209 | 21.33171 | 3.651238 | 85 | 5.842323 | 8.02E-06 | *** |
| 4_331 - 8_213 | -37.4039 | 3.800329 | 85 | -9.84228 | 2.09E-10 | *** |
| 4_331 - 9_207 | 12.23439 | 3.800329 | 85 | 3.219298 | 0.093456 |  |
| 4_331 - 91_7 | 6.8782 | 3.800329 | 85 | 1.809896 | 0.865492 |  |
| 4_331 - 92_338 | 29.85032 | 3.651238 | 85 | 8.175396 | 4.36E-10 | *** |
| 4_331 - 93_21 | -28.3737 | 3.651238 | 85 | -7.77098 | 1.68E-09 | *** |
| 4_331 - Control Sawdust | 52.43083 | 3.651238 | 85 | 14.35974 | 2.08E-10 | *** |
| 5_188 - 6_211 | 4.003019 | 3.800329 | 85 | 1.053335 | 0.998557 |  |
| 5_188 - 7_209 | 10.57341 | 3.800329 | 85 | 2.782235 | 0.253483 |  |
| 5_188 - 8_213 | -48.1622 | 3.943788 | 85 | -12.2122 | 2.08E-10 | *** |
| 5_188 - 9_207 | 1.47609 | 3.943788 | 85 | 0.374282 | 1 |  |
| 5_188 - 91_7 | -3.8801 | 3.943788 | 85 | -0.98385 | 0.999287 |  |
| 5_188 - 92_338 | 19.09201 | 3.800329 | 85 | 5.02378 | 0.000228 | *** |
| 5_188 - 93_21 | -39.132 | 3.800329 | 85 | -10.297 | 2.08E-10 | *** |
| 5_188 - Control Sawdust | 41.67252 | 3.800329 | 85 | 10.9655 | 2.08E-10 | *** |
| 6_211 - 7_209 | 6.570388 | 3.651238 | 85 | 1.799496 | 0.870202 |  |
| 6_211 - 8_213 | -52.1652 | 3.800329 | 85 | -13.7265 | 2.08E-10 | *** |
| 6_211 - 9_207 | -2.52693 | 3.800329 | 85 | -0.66492 | 0.999991 |  |
| 6_211 - 91_7 | -7.88312 | 3.800329 | 85 | -2.07433 | 0.716065 |  |
| 6_211 - 92_338 | 15.089 | 3.651238 | 85 | 4.13257 | 0.006026 | ** |
| 6_211 - 93_21 | -43.135 | 3.651238 | 85 | -11.8138 | 2.08E-10 | *** |
| 6_211 - Control Sawdust | 37.66951 | 3.651238 | 85 | 10.31691 | 2.08E-10 | *** |
| 7_209 - 8_213 | -58.7356 | 3.800329 | 85 | -15.4554 | 2.08E-10 | *** |
| 7_209 - 9_207 | -9.09732 | 3.800329 | 85 | -2.39382 | 0.493253 |  |
| 7_209 - 91_7 | -14.4535 | 3.800329 | 85 | -3.80323 | 0.017654 | * |
| 7_209 - 92_338 | 8.518608 | 3.651238 | 85 | 2.333074 | 0.536108 |  |
| 7_209 - 93_21 | -49.7054 | 3.651238 | 85 | -13.6133 | 2.08E-10 | *** |
| 7_209 - Control Sawdust | 31.09912 | 3.651238 | 85 | 8.517418 | 2.55E-10 | *** |
| 8_213 - 9_207 | 49.63831 | 3.943788 | 85 | 12.58646 | 2.08E-10 | *** |
| 8_213 - 91_7 | 44.28211 | 3.943788 | 85 | 11.22832 | 2.08E-10 | *** |
| 8_213 - 92_338 | 67.25423 | 3.800329 | 85 | 17.69695 | 2.08E-10 | *** |
| 8_213 - 93_21 | 9.030228 | 3.800329 | 85 | 2.37617 | 0.505643 |  |
| 8_213 - Control Sawdust | 89.83474 | 3.800329 | 85 | 23.63868 | 2.08E-10 | *** |
| 9_207 - 91_7 | -5.35619 | 3.943788 | 85 | -1.35813 | 0.984131 |  |
| 9_207 - 92_338 | 17.61592 | 3.800329 | 85 | 4.635369 | 0.001006 | ** |
| 9_207 - 93_21 | -40.6081 | 3.800329 | 85 | -10.6854 | 2.08E-10 | *** |
| 9_207 - Control Sawdust | 40.19644 | 3.800329 | 85 | 10.57709 | 2.08E-10 | *** |
| 91_7 - 92_338 | 22.97212 | 3.800329 | 85 | 6.044771 | 3.38E-06 | *** |
| 91_7 - 93_21 | -35.2519 | 3.800329 | 85 | -9.27601 | 2.1E-10 | *** |
| 91_7 - Control Sawdust | 45.55263 | 3.800329 | 85 | 11.9865 | 2.08E-10 | *** |
| 92_338 - 93_21 | -58.224 | 3.651238 | 85 | -15.9464 | 2.08E-10 | *** |
| 92_338 - Control Sawdust | 22.58051 | 3.651238 | 85 | 6.184344 | 1.85E-06 | *** |
| 93_21 - Control Sawdust | 80.80451 | 3.651238 | 85 | 22.13072 | 2.08E-10 | *** |

| **Sample** | **mean_Value** | **SE** | **df** | **lower.CL** | **upper.CL** | **Group** |
| --- | --- | --- | --- | --- | --- | --- |
| Control Sawdust | 29.0942 | 2.581815 | 85 | 21.37668 | 36.81171 | a |
| 92_338 | 51.67471 | 2.581815 | 85 | 43.95719 | 59.39222 | b |
| 7_209 | 60.19332 | 2.581815 | 85 | 52.4758 | 67.91083 | bc |
| 6_211 | 66.7637 | 2.581815 | 85 | 59.04619 | 74.48122 | cd |
| 1a_159 | 68.45098 | 2.581815 | 85 | 60.73346 | 76.1685 | cd |
| 9_207 | 69.29063 | 2.788679 | 85 | 60.95476 | 77.6265 | cde |
| 5_188 | 70.76672 | 2.788679 | 85 | 62.43085 | 79.10259 | cde |
| 91_7 | 74.64683 | 2.788679 | 85 | 66.31096 | 82.9827 | de |
| 2_2 | 75.97111 | 2.581815 | 85 | 68.25359 | 83.68863 | de |
| 4_331 | 81.52503 | 2.581815 | 85 | 73.80751 | 89.24254 | e |
| 93_21 | 109.8987 | 2.581815 | 85 | 102.1812 | 117.6162 | f |
| 8_213 | 118.9289 | 2.788679 | 85 | 110.5931 | 127.2648 | f |
| 3_10 | 132.9759 | 1.971894 | 85 | 127.0815 | 138.8702 | g |
| 1_167 | 144.0007 | 2.581815 | 85 | 136.2832 | 151.7182 | g |

**Phosphorus (Suppl. Fig. S2e)**

| **Info** | **Value** |
| --- | --- |
| Best model family | gaussian |
| Model name | Sqrt_Normal |

| **Effect** | **Df** | **Deviance** | **Resid. Df** | **Resid. Dev** | **Pr(>Chi)** |
| --- | --- | --- | --- | --- | --- |
| NULL |  |  | 98 | 49624.76 |  |
| Sample | 13 | 48263.22 | 85 | 1361.539 | 0 |

| **Comparison** | **Estimate** | **SE** | **df** | **t.ratio** | **p_value** | **Significance** |
| --- | --- | --- | --- | --- | --- | --- |
| 1_167 - 1a_159 | 2.533018 | 2.139299 | 85 | 1.184041 | 0.995433 |  |
| 1_167 - 2_2 | 11.63257 | 2.139299 | 85 | 5.43756 | 4.34E-05 | *** |
| 1_167 - 3_10 | -22.6714 | 1.903455 | 85 | -11.9107 | 2.08E-10 | *** |
| 1_167 - 4_331 | 1.397998 | 2.139299 | 85 | 0.653484 | 0.999993 |  |
| 1_167 - 5_188 | 21.60626 | 2.226653 | 85 | 9.703467 | 2.09E-10 | *** |
| 1_167 - 6_211 | 32.80515 | 2.139299 | 85 | 15.33453 | 2.08E-10 | *** |
| 1_167 - 7_209 | 34.59971 | 2.139299 | 85 | 16.17339 | 2.08E-10 | *** |
| 1_167 - 8_213 | -27.9473 | 2.226653 | 85 | -12.5513 | 2.08E-10 | *** |
| 1_167 - 9_207 | 16.65546 | 2.226653 | 85 | 7.480043 | 5.81E-09 | *** |
| 1_167 - 91_7 | 20.60176 | 2.226653 | 85 | 9.252342 | 2.1E-10 | *** |
| 1_167 - 92_338 | 13.22109 | 2.139299 | 85 | 6.180103 | 1.88E-06 | *** |
| 1_167 - 93_21 | 8.60357 | 2.139299 | 85 | 4.021677 | 0.008737 | ** |
| 1_167 - Control Sawdust | 57.06873 | 2.139299 | 85 | 26.67637 | 2.08E-10 | *** |
| 1a_159 - 2_2 | 9.09955 | 2.139299 | 85 | 4.253519 | 0.003978 | ** |
| 1a_159 - 3_10 | -25.2045 | 1.903455 | 85 | -13.2414 | 2.08E-10 | *** |
| 1a_159 - 4_331 | -1.13502 | 2.139299 | 85 | -0.53056 | 0.999999 |  |
| 1a_159 - 5_188 | 19.07324 | 2.226653 | 85 | 8.565878 | 2.45E-10 | *** |
| 1a_159 - 6_211 | 30.27214 | 2.139299 | 85 | 14.15049 | 2.08E-10 | *** |
| 1a_159 - 7_209 | 32.06669 | 2.139299 | 85 | 14.98935 | 2.08E-10 | *** |
| 1a_159 - 8_213 | -30.4803 | 2.226653 | 85 | -13.6889 | 2.08E-10 | *** |
| 1a_159 - 9_207 | 14.12244 | 2.226653 | 85 | 6.342453 | 9.28E-07 | *** |
| 1a_159 - 91_7 | 18.06874 | 2.226653 | 85 | 8.114753 | 5.09E-10 | *** |
| 1a_159 - 92_338 | 10.68807 | 2.139299 | 85 | 4.996062 | 0.000254 | *** |
| 1a_159 - 93_21 | 6.070553 | 2.139299 | 85 | 2.837636 | 0.226451 |  |
| 1a_159 - Control Sawdust | 54.53571 | 2.139299 | 85 | 25.49232 | 2.08E-10 | *** |
| 2_2 - 3_10 | -34.304 | 1.903455 | 85 | -18.022 | 2.08E-10 | *** |
| 2_2 - 4_331 | -10.2346 | 2.139299 | 85 | -4.78408 | 0.000576 | *** |
| 2_2 - 5_188 | 9.973689 | 2.226653 | 85 | 4.479229 | 0.001785 | ** |
| 2_2 - 6_211 | 21.17259 | 2.139299 | 85 | 9.896973 | 2.09E-10 | *** |
| 2_2 - 7_209 | 22.96714 | 2.139299 | 85 | 10.73583 | 2.08E-10 | *** |
| 2_2 - 8_213 | -39.5799 | 2.226653 | 85 | -17.7755 | 2.08E-10 | *** |
| 2_2 - 9_207 | 5.022894 | 2.226653 | 85 | 2.255804 | 0.591037 |  |
| 2_2 - 91_7 | 8.96919 | 2.226653 | 85 | 4.028104 | 0.008553 | ** |
| 2_2 - 92_338 | 1.588521 | 2.139299 | 85 | 0.742543 | 0.999968 |  |
| 2_2 - 93_21 | -3.029 | 2.139299 | 85 | -1.41588 | 0.977434 |  |
| 2_2 - Control Sawdust | 45.43616 | 2.139299 | 85 | 21.23881 | 2.08E-10 | *** |
| 3_10 - 4_331 | 24.06943 | 1.903455 | 85 | 12.64513 | 2.08E-10 | *** |
| 3_10 - 5_188 | 44.27769 | 2.001131 | 85 | 22.12633 | 2.08E-10 | *** |
| 3_10 - 6_211 | 55.47659 | 1.903455 | 85 | 29.1452 | 2.08E-10 | *** |
| 3_10 - 7_209 | 57.27115 | 1.903455 | 85 | 30.08799 | 2.08E-10 | *** |
| 3_10 - 8_213 | -5.27588 | 2.001131 | 85 | -2.63645 | 0.334085 |  |
| 3_10 - 9_207 | 39.3269 | 2.001131 | 85 | 19.65233 | 2.08E-10 | *** |
| 3_10 - 91_7 | 43.27319 | 2.001131 | 85 | 21.62437 | 2.08E-10 | *** |
| 3_10 - 92_338 | 35.89252 | 1.903455 | 85 | 18.85651 | 2.08E-10 | *** |
| 3_10 - 93_21 | 31.275 | 1.903455 | 85 | 16.43065 | 2.08E-10 | *** |
| 3_10 - Control Sawdust | 79.74016 | 1.903455 | 85 | 41.89232 | 2.08E-10 | *** |
| 4_331 - 5_188 | 20.20826 | 2.226653 | 85 | 9.07562 | 2.12E-10 | *** |
| 4_331 - 6_211 | 31.40716 | 2.139299 | 85 | 14.68105 | 2.08E-10 | *** |
| 4_331 - 7_209 | 33.20171 | 2.139299 | 85 | 15.5199 | 2.08E-10 | *** |
| 4_331 - 8_213 | -29.3453 | 2.226653 | 85 | -13.1791 | 2.08E-10 | *** |
| 4_331 - 9_207 | 15.25746 | 2.226653 | 85 | 6.852196 | 9.69E-08 | *** |
| 4_331 - 91_7 | 19.20376 | 2.226653 | 85 | 8.624495 | 2.37E-10 | *** |
| 4_331 - 92_338 | 11.82309 | 2.139299 | 85 | 5.526619 | 3.01E-05 | *** |
| 4_331 - 93_21 | 7.205572 | 2.139299 | 85 | 3.368193 | 0.063142 |  |
| 4_331 - Control Sawdust | 55.67073 | 2.139299 | 85 | 26.02288 | 2.08E-10 | *** |
| 5_188 - 6_211 | 11.1989 | 2.226653 | 85 | 5.029475 | 0.000223 | *** |
| 5_188 - 7_209 | 12.99346 | 2.226653 | 85 | 5.83542 | 8.26E-06 | *** |
| 5_188 - 8_213 | -49.5536 | 2.310707 | 85 | -21.4452 | 2.08E-10 | *** |
| 5_188 - 9_207 | -4.95079 | 2.310707 | 85 | -2.14255 | 0.670306 |  |
| 5_188 - 91_7 | -1.0045 | 2.310707 | 85 | -0.43471 | 1 |  |
| 5_188 - 92_338 | -8.38517 | 2.226653 | 85 | -3.76582 | 0.019835 | * |
| 5_188 - 93_21 | -13.0027 | 2.226653 | 85 | -5.83956 | 8.12E-06 | *** |
| 5_188 - Control Sawdust | 35.46247 | 2.226653 | 85 | 15.92636 | 2.08E-10 | *** |
| 6_211 - 7_209 | 1.794558 | 2.139299 | 85 | 0.838853 | 0.999873 |  |
| 6_211 - 8_213 | -60.7525 | 2.226653 | 85 | -27.2842 | 2.08E-10 | *** |
| 6_211 - 9_207 | -16.1497 | 2.226653 | 85 | -7.2529 | 1.6E-08 | *** |
| 6_211 - 91_7 | -12.2034 | 2.226653 | 85 | -5.4806 | 3.64E-05 | *** |
| 6_211 - 92_338 | -19.5841 | 2.139299 | 85 | -9.15443 | 2.11E-10 | *** |
| 6_211 - 93_21 | -24.2016 | 2.139299 | 85 | -11.3129 | 2.08E-10 | *** |
| 6_211 - Control Sawdust | 24.26357 | 2.139299 | 85 | 11.34183 | 2.08E-10 | *** |
| 7_209 - 8_213 | -62.547 | 2.226653 | 85 | -28.0901 | 2.08E-10 | *** |
| 7_209 - 9_207 | -17.9443 | 2.226653 | 85 | -8.05884 | 5.98E-10 | *** |
| 7_209 - 91_7 | -13.998 | 2.226653 | 85 | -6.28654 | 1.19E-06 | *** |
| 7_209 - 92_338 | -21.3786 | 2.139299 | 85 | -9.99328 | 2.09E-10 | *** |
| 7_209 - 93_21 | -25.9961 | 2.139299 | 85 | -12.1517 | 2.08E-10 | *** |
| 7_209 - Control Sawdust | 22.46902 | 2.139299 | 85 | 10.50298 | 2.08E-10 | *** |
| 8_213 - 9_207 | 44.60277 | 2.310707 | 85 | 19.30265 | 2.08E-10 | *** |
| 8_213 - 91_7 | 48.54907 | 2.310707 | 85 | 21.01048 | 2.08E-10 | *** |
| 8_213 - 92_338 | 41.1684 | 2.226653 | 85 | 18.48891 | 2.08E-10 | *** |
| 8_213 - 93_21 | 36.55088 | 2.226653 | 85 | 16.41517 | 2.08E-10 | *** |
| 8_213 - Control Sawdust | 85.01604 | 2.226653 | 85 | 38.18109 | 2.08E-10 | *** |
| 9_207 - 91_7 | 3.946296 | 2.310707 | 85 | 1.70783 | 0.90742 |  |
| 9_207 - 92_338 | -3.43437 | 2.226653 | 85 | -1.54239 | 0.955398 |  |
| 9_207 - 93_21 | -8.05189 | 2.226653 | 85 | -3.61614 | 0.031219 | * |
| 9_207 - Control Sawdust | 40.41327 | 2.226653 | 85 | 18.14978 | 2.08E-10 | *** |
| 91_7 - 92_338 | -7.38067 | 2.226653 | 85 | -3.31469 | 0.0729 |  |
| 91_7 - 93_21 | -11.9982 | 2.226653 | 85 | -5.38844 | 5.31E-05 | *** |
| 91_7 - Control Sawdust | 36.46697 | 2.226653 | 85 | 16.37748 | 2.08E-10 | *** |
| 92_338 - 93_21 | -4.61752 | 2.139299 | 85 | -2.15843 | 0.659388 |  |
| 92_338 - Control Sawdust | 43.84764 | 2.139299 | 85 | 20.49626 | 2.08E-10 | *** |
| 93_21 - Control Sawdust | 48.46516 | 2.139299 | 85 | 22.65469 | 2.08E-10 | *** |

| **Sample** | **mean_Value** | **SE** | **df** | **lower.CL** | **upper.CL** | **Group** |
| --- | --- | --- | --- | --- | --- | --- |
| Control Sawdust | 7.391484 | 1.512713 | 85 | 2.869709 | 11.91326 | a |
| 7_209 | 29.8605 | 1.512713 | 85 | 25.33872 | 34.38227 | b |
| 6_211 | 31.65506 | 1.512713 | 85 | 27.13328 | 36.17683 | b |
| 5_188 | 42.85396 | 1.633917 | 85 | 37.96988 | 47.73803 | c |
| 91_7 | 43.85845 | 1.633917 | 85 | 38.97438 | 48.74253 | cd |
| 9_207 | 47.80475 | 1.633917 | 85 | 42.92067 | 52.68882 | cde |
| 92_338 | 51.23912 | 1.512713 | 85 | 46.71735 | 55.7609 | def |
| 2_2 | 52.82764 | 1.512713 | 85 | 48.30587 | 57.34942 | ef |
| 93_21 | 55.85664 | 1.512713 | 85 | 51.33487 | 60.37842 | fg |
| 1a_159 | 61.92719 | 1.512713 | 85 | 57.40542 | 66.44897 | gh |
| 4_331 | 63.06221 | 1.512713 | 85 | 58.54044 | 67.58399 | gh |
| 1_167 | 64.46021 | 1.512713 | 85 | 59.93844 | 68.98199 | h |
| 3_10 | 87.13165 | 1.155354 | 85 | 83.67808 | 90.58521 | i |
| 8_213 | 92.40752 | 1.633917 | 85 | 87.52345 | 97.2916 | i |

**Nitrogen (Suppl. Fig. S2f)**

| **Info** | **Value** |
| --- | --- |
| Best model family | gaussian |
| Model name | Normal |

| **Effect** | **Df** | **Deviance** | **Resid. Df** | **Resid. Dev** | **Pr(>Chi)** |
| --- | --- | --- | --- | --- | --- |
| NULL |  |  | 94 | 141.4848 |  |
| Sample | 13 | 133.175 | 81 | 8.309857 | 1.3E-269 |

| **Comparison** | **Estimate** | **SE** | **df** | **t.ratio** | **p_value** | **Significance** |
| --- | --- | --- | --- | --- | --- | --- |
| 10 - 159 | 0.511429 | 0.187547 | 81 | 2.726931 | 0.283369 |  |
| 10 - 167 | -0.61714 | 0.187547 | 81 | -3.2906 | 0.07853 |  |
| 10 - 188 | 1.811429 | 0.187547 | 81 | 9.658515 | 2.55E-11 | *** |
| 10 - 207 | 1.29 | 0.19395 | 81 | 6.651196 | 2.8E-07 | *** |
| 10 - 209 | 1.397143 | 0.187547 | 81 | 7.449548 | 8.15E-09 | *** |
| 10 - 21 | 0.682857 | 0.187547 | 81 | 3.640986 | 0.029518 | * |
| 10 - 211 | 1.868571 | 0.187547 | 81 | 9.9632 | 2.52E-11 | *** |
| 10 - 213 | -2.06 | 0.187547 | 81 | -10.9839 | 2.51E-11 | *** |
| 10 - 331 | -0.33143 | 0.187547 | 81 | -1.76717 | 0.8839 |  |
| 10 - 338 | 0.554286 | 0.187547 | 81 | 2.955445 | 0.176729 |  |
| 10 - 339 | 1.811429 | 0.187547 | 81 | 9.658515 | 2.55E-11 | *** |
| 10 - 7 | 0.811429 | 0.187547 | 81 | 4.326527 | 0.003199 | ** |
| 10 - (Sawdust-Control) | 2.597143 | 0.187547 | 81 | 13.84793 | 2.5E-11 | *** |
| 159 - 167 | -1.12857 | 0.171206 | 81 | -6.59187 | 3.63E-07 | *** |
| 159 - 188 | 1.3 | 0.171206 | 81 | 7.59317 | 4.28E-09 | *** |
| 159 - 207 | 0.778571 | 0.178197 | 81 | 4.369152 | 0.002755 | ** |
| 159 - 209 | 0.885714 | 0.171206 | 81 | 5.173368 | 0.000136 | *** |
| 159 - 21 | 0.171429 | 0.171206 | 81 | 1.001297 | 0.999134 |  |
| 159 - 211 | 1.357143 | 0.171206 | 81 | 7.926935 | 9.69E-10 | *** |
| 159 - 213 | -2.57143 | 0.171206 | 81 | -15.0195 | 2.5E-11 | *** |
| 159 - 331 | -0.84286 | 0.171206 | 81 | -4.92304 | 0.000359 | *** |
| 159 - 338 | 0.042857 | 0.171206 | 81 | 0.250324 | 1 |  |
| 159 - 339 | 1.3 | 0.171206 | 81 | 7.59317 | 4.28E-09 | *** |
| 159 - 7 | 0.3 | 0.171206 | 81 | 1.75227 | 0.890037 |  |
| 159 - (Sawdust-Control) | 2.085714 | 0.171206 | 81 | 12.18245 | 2.5E-11 | *** |
| 167 - 188 | 2.428571 | 0.171206 | 81 | 14.18504 | 2.5E-11 | *** |
| 167 - 207 | 1.907143 | 0.178197 | 81 | 10.70242 | 2.51E-11 | *** |
| 167 - 209 | 2.014286 | 0.171206 | 81 | 11.76524 | 2.5E-11 | *** |
| 167 - 21 | 1.3 | 0.171206 | 81 | 7.59317 | 4.28E-09 | *** |
| 167 - 211 | 2.485714 | 0.171206 | 81 | 14.51881 | 2.5E-11 | *** |
| 167 - 213 | -1.44286 | 0.171206 | 81 | -8.42758 | 1.23E-10 | *** |
| 167 - 331 | 0.285714 | 0.171206 | 81 | 1.668829 | 0.920624 |  |
| 167 - 338 | 1.171429 | 0.171206 | 81 | 6.842197 | 1.21E-07 | *** |
| 167 - 339 | 2.428571 | 0.171206 | 81 | 14.18504 | 2.5E-11 | *** |
| 167 - 7 | 1.428571 | 0.171206 | 81 | 8.344143 | 1.67E-10 | *** |
| 167 - (Sawdust-Control) | 3.214286 | 0.171206 | 81 | 18.77432 | 2.5E-11 | *** |
| 188 - 207 | -0.52143 | 0.178197 | 81 | -2.92613 | 0.188493 |  |
| 188 - 209 | -0.41429 | 0.171206 | 81 | -2.4198 | 0.47559 |  |
| 188 - 21 | -1.12857 | 0.171206 | 81 | -6.59187 | 3.63E-07 | *** |
| 188 - 211 | 0.057143 | 0.171206 | 81 | 0.333766 | 1 |  |
| 188 - 213 | -3.87143 | 0.171206 | 81 | -22.6126 | 2.5E-11 | *** |
| 188 - 331 | -2.14286 | 0.171206 | 81 | -12.5162 | 2.5E-11 | *** |
| 188 - 338 | -1.25714 | 0.171206 | 81 | -7.34285 | 1.31E-08 | *** |
| 188 - 339 | 6E-15 | 0.171206 | 81 | 3.5E-14 | 1 |  |
| 188 - 7 | -1 | 0.171206 | 81 | -5.8409 | 9.01E-06 | *** |
| 188 - (Sawdust-Control) | 0.785714 | 0.171206 | 81 | 4.589278 | 0.001251 | ** |
| 207 - 209 | 0.107143 | 0.178197 | 81 | 0.601259 | 0.999997 |  |
| 207 - 21 | -0.60714 | 0.178197 | 81 | -3.40714 | 0.057498 |  |
| 207 - 211 | 0.578571 | 0.178197 | 81 | 3.246801 | 0.087958 |  |
| 207 - 213 | -3.35 | 0.178197 | 81 | -18.7994 | 2.5E-11 | *** |
| 207 - 331 | -1.62143 | 0.178197 | 81 | -9.09906 | 2.97E-11 | *** |
| 207 - 338 | -0.73571 | 0.178197 | 81 | -4.12865 | 0.0063 | ** |
| 207 - 339 | 0.521429 | 0.178197 | 81 | 2.926129 | 0.188493 |  |
| 207 - 7 | -0.47857 | 0.178197 | 81 | -2.68563 | 0.306262 |  |
| 207 - (Sawdust-Control) | 1.307143 | 0.178197 | 81 | 7.335365 | 1.36E-08 | *** |
| 209 - 21 | -0.71429 | 0.171206 | 81 | -4.17207 | 0.005442 | ** |
| 209 - 211 | 0.471429 | 0.171206 | 81 | 2.753567 | 0.269178 |  |
| 209 - 213 | -3.45714 | 0.171206 | 81 | -20.1928 | 2.5E-11 | *** |
| 209 - 331 | -1.72857 | 0.171206 | 81 | -10.0964 | 2.51E-11 | *** |
| 209 - 338 | -0.84286 | 0.171206 | 81 | -4.92304 | 0.000359 | *** |
| 209 - 339 | 0.414286 | 0.171206 | 81 | 2.419801 | 0.47559 |  |
| 209 - 7 | -0.58571 | 0.171206 | 81 | -3.4211 | 0.055338 |  |
| 209 - (Sawdust-Control) | 1.2 | 0.171206 | 81 | 7.00908 | 5.8E-08 | *** |
| 21 - 211 | 1.185714 | 0.171206 | 81 | 6.925638 | 8.38E-08 | *** |
| 21 - 213 | -2.74286 | 0.171206 | 81 | -16.0208 | 2.5E-11 | *** |
| 21 - 331 | -1.01429 | 0.171206 | 81 | -5.92434 | 6.35E-06 | *** |
| 21 - 338 | -0.12857 | 0.171206 | 81 | -0.75097 | 0.999963 |  |
| 21 - 339 | 1.128571 | 0.171206 | 81 | 6.591873 | 3.63E-07 | *** |
| 21 - 7 | 0.128571 | 0.171206 | 81 | 0.750973 | 0.999963 |  |
| 21 - (Sawdust-Control) | 1.914286 | 0.171206 | 81 | 11.18115 | 2.5E-11 | *** |
| 211 - 213 | -3.92857 | 0.171206 | 81 | -22.9464 | 2.5E-11 | *** |
| 211 - 331 | -2.2 | 0.171206 | 81 | -12.85 | 2.5E-11 | *** |
| 211 - 338 | -1.31429 | 0.171206 | 81 | -7.67661 | 2.95E-09 | *** |
| 211 - 339 | -0.05714 | 0.171206 | 81 | -0.33377 | 1 |  |
| 211 - 7 | -1.05714 | 0.171206 | 81 | -6.17467 | 2.2E-06 | *** |
| 211 - (Sawdust-Control) | 0.728571 | 0.171206 | 81 | 4.255513 | 0.004092 | ** |
| 213 - 331 | 1.728571 | 0.171206 | 81 | 10.09641 | 2.51E-11 | *** |
| 213 - 338 | 2.614286 | 0.171206 | 81 | 15.26978 | 2.5E-11 | *** |
| 213 - 339 | 3.871429 | 0.171206 | 81 | 22.61263 | 2.5E-11 | *** |
| 213 - 7 | 2.871429 | 0.171206 | 81 | 16.77173 | 2.5E-11 | *** |
| 213 - (Sawdust-Control) | 4.657143 | 0.171206 | 81 | 27.2019 | 2.5E-11 | *** |
| 331 - 338 | 0.885714 | 0.171206 | 81 | 5.173368 | 0.000136 | *** |
| 331 - 339 | 2.142857 | 0.171206 | 81 | 12.51621 | 2.5E-11 | *** |
| 331 - 7 | 1.142857 | 0.171206 | 81 | 6.675314 | 2.52E-07 | *** |
| 331 - (Sawdust-Control) | 2.928571 | 0.171206 | 81 | 17.10549 | 2.5E-11 | *** |
| 338 - 339 | 1.257143 | 0.171206 | 81 | 7.342845 | 1.31E-08 | *** |
| 338 - 7 | 0.257143 | 0.171206 | 81 | 1.501946 | 0.963481 |  |
| 338 - (Sawdust-Control) | 2.042857 | 0.171206 | 81 | 11.93212 | 2.5E-11 | *** |
| 339 - 7 | -1 | 0.171206 | 81 | -5.8409 | 9.01E-06 | *** |
| 339 - (Sawdust-Control) | 0.785714 | 0.171206 | 81 | 4.589278 | 0.001251 | ** |
| 7 - (Sawdust-Control) | 1.785714 | 0.171206 | 81 | 10.43018 | 2.51E-11 | *** |

| **Sample** | **mean_Value** | **SE** | **df** | **lower.CL** | **upper.CL** | **Group** |
| --- | --- | --- | --- | --- | --- | --- |
| Sawdust-Control | 0.142857 | 0.121061 | 81 | -0.21953 | 0.50524 | a |
| 211 | 0.871429 | 0.121061 | 81 | 0.509045 | 1.233812 | b |
| 339 | 0.928571 | 0.121061 | 81 | 0.566188 | 1.290955 | b |
| 188 | 0.928571 | 0.121061 | 81 | 0.566188 | 1.290955 | b |
| 209 | 1.342857 | 0.121061 | 81 | 0.980474 | 1.70524 | bc |
| 207 | 1.45 | 0.130761 | 81 | 1.058581 | 1.841419 | bcd |
| 7 | 1.928571 | 0.121061 | 81 | 1.566188 | 2.290955 | cde |
| 21 | 2.057143 | 0.121061 | 81 | 1.69476 | 2.419526 | de |
| 338 | 2.185714 | 0.121061 | 81 | 1.823331 | 2.548097 | ef |
| 159 | 2.228571 | 0.121061 | 81 | 1.866188 | 2.590955 | ef |
| 10 | 2.74 | 0.143242 | 81 | 2.311222 | 3.168778 | fg |
| 331 | 3.071429 | 0.121061 | 81 | 2.709045 | 3.433812 | g |
| 167 | 3.357143 | 0.121061 | 81 | 2.99476 | 3.719526 | g |
| 213 | 4.8 | 0.121061 | 81 | 4.437617 | 5.162383 | h |

**Fatty acids (Suppl. Fig. S3a)**

| **Info** | **Value** |
| --- | --- |
| Best model family | gaussian |
| Model name | Normal_log |

| **Effect** | **Df** | **Deviance** | **Resid. Df** | **Resid. Dev** | **Pr(>Chi)** |
| --- | --- | --- | --- | --- | --- |
| NULL |  |  | 94 | 183.1554 |  |
| Sample | 13 | 176.4102 | 81 | 6.745249 | 0 |

| **Comparison** | **Estimate** | **SE** | **df** | **t.ratio** | **p_value** | **Significance** |
| --- | --- | --- | --- | --- | --- | --- |
| 10 - 159 | 0.812262 | 0.154249 | 81 | 5.265916 | 9.42E-05 | *** |
| 10 - 167 | 0.465698 | 0.154249 | 81 | 3.019133 | 0.153064 |  |
| 10 - 188 | 1.341735 | 0.154249 | 81 | 8.698499 | 5.35E-11 | *** |
| 10 - 207 | 1.699882 | 0.154249 | 81 | 11.02037 | 2.5E-11 | *** |
| 10 - 209 | 2.697355 | 0.154249 | 81 | 17.48702 | 2.5E-11 | *** |
| 10 - 211 | 2.925197 | 0.154249 | 81 | 18.96412 | 2.5E-11 | *** |
| 10 - 213 | 0.454059 | 0.160547 | 81 | 2.82819 | 0.231877 |  |
| 10 - 331 | 1.148651 | 0.154249 | 81 | 7.44673 | 8.25E-09 | *** |
| 10 - 338 | -1.03018 | 0.160547 | 81 | -6.41669 | 7.77E-07 | *** |
| 10 - CK | 4.16857 | 0.154249 | 81 | 27.02493 | 2.5E-11 | *** |
| 10 - P2 | 1.136791 | 0.154249 | 81 | 7.369839 | 1.16E-08 | *** |
| 10 - P21 | 0.064591 | 0.154249 | 81 | 0.418746 | 1 |  |
| 10 - P7 | -0.6031 | 0.160547 | 81 | -3.75653 | 0.020839 | * |
| 159 - 167 | -0.34656 | 0.154249 | 81 | -2.24678 | 0.597559 |  |
| 159 - 188 | 0.529473 | 0.154249 | 81 | 3.432584 | 0.053613 |  |
| 159 - 207 | 0.88762 | 0.154249 | 81 | 5.754459 | 1.29E-05 | *** |
| 159 - 209 | 1.885093 | 0.154249 | 81 | 12.2211 | 2.5E-11 | *** |
| 159 - 211 | 2.112934 | 0.154249 | 81 | 13.6982 | 2.5E-11 | *** |
| 159 - 213 | -0.3582 | 0.160547 | 81 | -2.23114 | 0.608609 |  |
| 159 - 331 | 0.336388 | 0.154249 | 81 | 2.180814 | 0.643871 |  |
| 159 - 338 | -1.84245 | 0.160547 | 81 | -11.476 | 2.5E-11 | *** |
| 159 - CK | 3.356308 | 0.154249 | 81 | 21.75902 | 2.5E-11 | *** |
| 159 - P2 | 0.324528 | 0.154249 | 81 | 2.103923 | 0.696358 |  |
| 159 - P21 | -0.74767 | 0.154249 | 81 | -4.84717 | 0.000479 | *** |
| 159 - P7 | -1.41536 | 0.160547 | 81 | -8.81585 | 4.18E-11 | *** |
| 167 - 188 | 0.876037 | 0.154249 | 81 | 5.679367 | 1.76E-05 | *** |
| 167 - 207 | 1.234184 | 0.154249 | 81 | 8.001242 | 7E-10 | *** |
| 167 - 209 | 2.231657 | 0.154249 | 81 | 14.46788 | 2.5E-11 | *** |
| 167 - 211 | 2.459499 | 0.154249 | 81 | 15.94498 | 2.5E-11 | *** |
| 167 - 213 | -0.01164 | 0.160547 | 81 | -0.0725 | 1 |  |
| 167 - 331 | 0.682953 | 0.154249 | 81 | 4.427597 | 0.002241 | ** |
| 167 - 338 | -1.49588 | 0.160547 | 81 | -9.31737 | 2.68E-11 | *** |
| 167 - CK | 3.702872 | 0.154249 | 81 | 24.0058 | 2.5E-11 | *** |
| 167 - P2 | 0.671092 | 0.154249 | 81 | 4.350707 | 0.00294 | ** |
| 167 - P21 | -0.40111 | 0.154249 | 81 | -2.60039 | 0.356743 |  |
| 167 - P7 | -1.0688 | 0.160547 | 81 | -6.65722 | 2.73E-07 | *** |
| 188 - 207 | 0.358147 | 0.154249 | 81 | 2.321875 | 0.544319 |  |
| 188 - 209 | 1.35562 | 0.154249 | 81 | 8.788516 | 4.4E-11 | *** |
| 188 - 211 | 1.583462 | 0.154249 | 81 | 10.26562 | 2.51E-11 | *** |
| 188 - 213 | -0.88768 | 0.160547 | 81 | -5.52906 | 3.26E-05 | *** |
| 188 - 331 | -0.19308 | 0.154249 | 81 | -1.25177 | 0.992255 |  |
| 188 - 338 | -2.37192 | 0.160547 | 81 | -14.7739 | 2.5E-11 | *** |
| 188 - CK | 2.826835 | 0.154249 | 81 | 18.32643 | 2.5E-11 | *** |
| 188 - P2 | -0.20494 | 0.154249 | 81 | -1.32866 | 0.98679 |  |
| 188 - P21 | -1.27714 | 0.154249 | 81 | -8.27975 | 2.16E-10 | *** |
| 188 - P7 | -1.94484 | 0.160547 | 81 | -12.1138 | 2.5E-11 | *** |
| 207 - 209 | 0.997473 | 0.154249 | 81 | 6.466641 | 6.26E-07 | *** |
| 207 - 211 | 1.225315 | 0.154249 | 81 | 7.943743 | 9E-10 | *** |
| 207 - 213 | -1.24582 | 0.160547 | 81 | -7.75984 | 2.03E-09 | *** |
| 207 - 331 | -0.55123 | 0.154249 | 81 | -3.57364 | 0.035959 | * |
| 207 - 338 | -2.73006 | 0.160547 | 81 | -17.0047 | 2.5E-11 | *** |
| 207 - CK | 2.468688 | 0.154249 | 81 | 16.00456 | 2.5E-11 | *** |
| 207 - P2 | -0.56309 | 0.154249 | 81 | -3.65054 | 0.028694 | * |
| 207 - P21 | -1.63529 | 0.154249 | 81 | -10.6016 | 2.51E-11 | *** |
| 207 - P7 | -2.30298 | 0.160547 | 81 | -14.3446 | 2.5E-11 | *** |
| 209 - 211 | 0.227842 | 0.154249 | 81 | 1.477102 | 0.967981 |  |
| 209 - 213 | -2.2433 | 0.160547 | 81 | -13.9728 | 2.5E-11 | *** |
| 209 - 331 | -1.5487 | 0.154249 | 81 | -10.0403 | 2.52E-11 | *** |
| 209 - 338 | -3.72754 | 0.160547 | 81 | -23.2177 | 2.5E-11 | *** |
| 209 - CK | 1.471215 | 0.154249 | 81 | 9.537917 | 2.57E-11 | *** |
| 209 - P2 | -1.56056 | 0.154249 | 81 | -10.1172 | 2.51E-11 | *** |
| 209 - P21 | -2.63276 | 0.154249 | 81 | -17.0683 | 2.5E-11 | *** |
| 209 - P7 | -3.30046 | 0.160547 | 81 | -20.5575 | 2.5E-11 | *** |
| 211 - 213 | -2.47114 | 0.160547 | 81 | -15.3919 | 2.5E-11 | *** |
| 211 - 331 | -1.77655 | 0.154249 | 81 | -11.5174 | 2.5E-11 | *** |
| 211 - 338 | -3.95538 | 0.160547 | 81 | -24.6368 | 2.5E-11 | *** |
| 211 - CK | 1.243373 | 0.154249 | 81 | 8.060816 | 5.4E-10 | *** |
| 211 - P2 | -1.78841 | 0.154249 | 81 | -11.5943 | 2.5E-11 | *** |
| 211 - P21 | -2.86061 | 0.154249 | 81 | -18.5454 | 2.5E-11 | *** |
| 211 - P7 | -3.5283 | 0.160547 | 81 | -21.9767 | 2.5E-11 | *** |
| 213 - 331 | 0.694592 | 0.160547 | 81 | 4.326397 | 0.0032 | ** |
| 213 - 338 | -1.48424 | 0.166608 | 81 | -8.90858 | 3.6E-11 | *** |
| 213 - CK | 3.714511 | 0.160547 | 81 | 23.13653 | 2.5E-11 | *** |
| 213 - P2 | 0.682732 | 0.160547 | 81 | 4.252523 | 0.004135 | ** |
| 213 - P21 | -0.38947 | 0.160547 | 81 | -2.42587 | 0.471396 |  |
| 213 - P7 | -1.05716 | 0.166608 | 81 | -6.34519 | 1.06E-06 | *** |
| 331 - 338 | -2.17883 | 0.160547 | 81 | -13.5713 | 2.5E-11 | *** |
| 331 - CK | 3.019919 | 0.154249 | 81 | 19.5782 | 2.5E-11 | *** |
| 331 - P2 | -0.01186 | 0.154249 | 81 | -0.07689 | 1 |  |
| 331 - P21 | -1.08406 | 0.154249 | 81 | -7.02798 | 5.33E-08 | *** |
| 331 - P7 | -1.75175 | 0.160547 | 81 | -10.9111 | 2.51E-11 | *** |
| 338 - CK | 5.198753 | 0.160547 | 81 | 32.3814 | 2.5E-11 | *** |
| 338 - P2 | 2.166973 | 0.160547 | 81 | 13.4974 | 2.5E-11 | *** |
| 338 - P21 | 1.094774 | 0.160547 | 81 | 6.819004 | 1.34E-07 | *** |
| 338 - P7 | 0.427082 | 0.166608 | 81 | 2.563393 | 0.379904 |  |
| CK - P2 | -3.03178 | 0.154249 | 81 | -19.6551 | 2.5E-11 | *** |
| CK - P21 | -4.10398 | 0.154249 | 81 | -26.6062 | 2.5E-11 | *** |
| CK - P7 | -4.77167 | 0.160547 | 81 | -29.7212 | 2.5E-11 | *** |
| P2 - P21 | -1.0722 | 0.154249 | 81 | -6.95109 | 7.49E-08 | *** |
| P2 - P7 | -1.73989 | 0.160547 | 81 | -10.8372 | 2.51E-11 | *** |
| P21 - P7 | -0.66769 | 0.160547 | 81 | -4.15885 | 0.005691 | ** |

| **Sample** | **mean_Value** | **SE** | **df** | **lower.CL** | **upper.CL** | **Group** |
| --- | --- | --- | --- | --- | --- | --- |
| CK | 0.552674 | 0.109071 | 81 | 0.398727 | 0.766058 | a |
| 211 | 1.916279 | 0.109071 | 81 | 1.382502 | 2.656144 | b |
| 209 | 2.406628 | 0.109071 | 81 | 1.736265 | 3.335815 | b |
| 207 | 6.525385 | 0.109071 | 81 | 4.707748 | 9.044802 | c |
| 188 | 9.335711 | 0.109071 | 81 | 6.735262 | 12.94018 | cd |
| 331 | 11.32408 | 0.109071 | 81 | 8.169771 | 15.69624 | d |
| P2 | 11.45918 | 0.109071 | 81 | 8.267244 | 15.88351 | d |
| 159 | 15.85238 | 0.109071 | 81 | 11.43672 | 21.9729 | de |
| 167 | 22.41844 | 0.109071 | 81 | 16.17381 | 31.07408 | ef |
| 213 | 22.6809 | 0.11781 | 81 | 15.94067 | 32.27113 | ef |
| P21 | 33.48143 | 0.109071 | 81 | 24.15522 | 46.40843 | f |
| 10 | 35.7154 | 0.109071 | 81 | 25.76693 | 49.50494 | f |
| P7 | 65.27983 | 0.11781 | 81 | 45.88018 | 92.88227 | g |
| 338 | 100.0595 | 0.11781 | 81 | 70.32414 | 142.3679 | g |

**Ergosterol (Suppl. Fig. S3b)**

| Info | Value |
| --- | --- |
| Best model family | gaussian |
| Model name | Sqrt_Normal |

| Effect | Df | Deviance | Resid. Df | Resid. Dev | Pr(>Chi) |
| --- | --- | --- | --- | --- | --- |
| NULL |  |  | 77 | 27.62522 |  |
| Sample | 12 | 26.17634 | 65 | 1.448884 | 5.8E-244 |

| Comparison | Estimate | SE | df | t.ratio | p_value | Significance |
| --- | --- | --- | --- | --- | --- | --- |
| 159 - 167 | -0.31723 | 0.086198 | 65 | -3.68023 | 0.025422 | * |
| 159 - 188 | 0.35744 | 0.086198 | 65 | 4.146704 | 0.006065 | ** |
| 159 - 207 | -0.2199 | 0.086198 | 65 | -2.55112 | 0.360387 |  |
| 159 - 209 | 0.230199 | 0.086198 | 65 | 2.670573 | 0.291995 |  |
| 159 - 211 | 0.590089 | 0.086198 | 65 | 6.845702 | 2.46E-07 | *** |
| 159 - 213 | -1.14291 | 0.086198 | 65 | -13.2591 | 1.46E-12 | *** |
| 159 - 331 | -0.39004 | 0.086198 | 65 | -4.52493 | 0.001717 | ** |
| 159 - 338 | -1.05329 | 0.086198 | 65 | -12.2194 | 1.48E-12 | *** |
| 159 - CK | 0.590089 | 0.086198 | 65 | 6.845702 | 2.46E-07 | *** |
| 159 - P10 | -0.82442 | 0.086198 | 65 | -9.56423 | 5.52E-12 | *** |
| 159 - P2 | 0.537384 | 0.086198 | 65 | 6.234269 | 2.84E-06 | *** |
| 159 - P7 | -0.30196 | 0.086198 | 65 | -3.5031 | 0.041962 | * |
| 167 - 188 | 0.67467 | 0.086198 | 65 | 7.826934 | 4.55E-09 | *** |
| 167 - 207 | 0.097328 | 0.086198 | 65 | 1.129112 | 0.995096 |  |
| 167 - 209 | 0.547429 | 0.086198 | 65 | 6.350803 | 1.79E-06 | *** |
| 167 - 211 | 0.907319 | 0.086198 | 65 | 10.52593 | 1.63E-12 | *** |
| 167 - 213 | -0.82568 | 0.086198 | 65 | -9.57883 | 5.29E-12 | *** |
| 167 - 331 | -0.07281 | 0.086198 | 65 | -0.8447 | 0.9997 |  |
| 167 - 338 | -0.73606 | 0.086198 | 65 | -8.53915 | 2.51E-10 | *** |
| 167 - CK | 0.907319 | 0.086198 | 65 | 10.52593 | 1.63E-12 | *** |
| 167 - P10 | -0.50719 | 0.086198 | 65 | -5.884 | 1.13E-05 | *** |
| 167 - P2 | 0.854615 | 0.086198 | 65 | 9.914498 | 2.54E-12 | *** |
| 167 - P7 | 0.015268 | 0.086198 | 65 | 0.177129 | 1 |  |
| 188 - 207 | -0.57734 | 0.086198 | 65 | -6.69782 | 4.47E-07 | *** |
| 188 - 209 | -0.12724 | 0.086198 | 65 | -1.47613 | 0.956331 |  |
| 188 - 211 | 0.232649 | 0.086198 | 65 | 2.698998 | 0.27695 |  |
| 188 - 213 | -1.50035 | 0.086198 | 65 | -17.4058 | 1.46E-12 | *** |
| 188 - 331 | -0.74748 | 0.086198 | 65 | -8.67164 | 1.47E-10 | *** |
| 188 - 338 | -1.41073 | 0.086198 | 65 | -16.3661 | 1.46E-12 | *** |
| 188 - CK | 0.232649 | 0.086198 | 65 | 2.698998 | 0.27695 |  |
| 188 - P10 | -1.18186 | 0.086198 | 65 | -13.7109 | 1.46E-12 | *** |
| 188 - P2 | 0.179945 | 0.086198 | 65 | 2.087564 | 0.671756 |  |
| 188 - P7 | -0.6594 | 0.086198 | 65 | -7.6498 | 9.38E-09 | *** |
| 207 - 209 | 0.450102 | 0.086198 | 65 | 5.221691 | 0.000141 | *** |
| 207 - 211 | 0.809991 | 0.086198 | 65 | 9.39682 | 9.31E-12 | *** |
| 207 - 213 | -0.92301 | 0.086198 | 65 | -10.7079 | 1.58E-12 | *** |
| 207 - 331 | -0.17014 | 0.086198 | 65 | -1.97382 | 0.745295 |  |
| 207 - 338 | -0.83339 | 0.086198 | 65 | -9.66827 | 4.18E-12 | *** |
| 207 - CK | 0.809991 | 0.086198 | 65 | 9.39682 | 9.31E-12 | *** |
| 207 - P10 | -0.60452 | 0.086198 | 65 | -7.01311 | 1.25E-07 | *** |
| 207 - P2 | 0.757287 | 0.086198 | 65 | 8.785386 | 9.31E-11 | *** |
| 207 - P7 | -0.08206 | 0.086198 | 65 | -0.95198 | 0.99901 |  |
| 209 - 211 | 0.35989 | 0.086198 | 65 | 4.175129 | 0.005532 | ** |
| 209 - 213 | -1.37311 | 0.086198 | 65 | -15.9296 | 1.46E-12 | *** |
| 209 - 331 | -0.62024 | 0.086198 | 65 | -7.19551 | 5.97E-08 | *** |
| 209 - 338 | -1.28349 | 0.086198 | 65 | -14.89 | 1.46E-12 | *** |
| 209 - CK | 0.35989 | 0.086198 | 65 | 4.175129 | 0.005532 | ** |
| 209 - P10 | -1.05462 | 0.086198 | 65 | -12.2348 | 1.48E-12 | *** |
| 209 - P2 | 0.307185 | 0.086198 | 65 | 3.563695 | 0.035455 | * |
| 209 - P7 | -0.53216 | 0.086198 | 65 | -6.17367 | 3.62E-06 | *** |
| 211 - 213 | -1.733 | 0.086198 | 65 | -20.1048 | 1.46E-12 | *** |
| 211 - 331 | -0.98013 | 0.086198 | 65 | -11.3706 | 1.51E-12 | *** |
| 211 - 338 | -1.64338 | 0.086198 | 65 | -19.0651 | 1.46E-12 | *** |
| 211 - CK | -1.4E-15 | 0.086198 | 65 | -1.7E-14 | 1 |  |
| 211 - P10 | -1.41451 | 0.086198 | 65 | -16.4099 | 1.46E-12 | *** |
| 211 - P2 | -0.0527 | 0.086198 | 65 | -0.61143 | 0.99999 |  |
| 211 - P7 | -0.89205 | 0.086198 | 65 | -10.3488 | 1.73E-12 | *** |
| 213 - 331 | 0.752868 | 0.086198 | 65 | 8.734122 | 1.14E-10 | *** |
| 213 - 338 | 0.089618 | 0.086198 | 65 | 1.039672 | 0.997699 |  |
| 213 - CK | 1.732999 | 0.086198 | 65 | 20.10476 | 1.46E-12 | *** |
| 213 - P10 | 0.318488 | 0.086198 | 65 | 3.694825 | 0.024365 | * |
| 213 - P2 | 1.680295 | 0.086198 | 65 | 19.49332 | 1.46E-12 | *** |
| 213 - P7 | 0.840948 | 0.086198 | 65 | 9.755956 | 3.41E-12 | *** |
| 331 - 338 | -0.66325 | 0.086198 | 65 | -7.69445 | 7.82E-09 | *** |
| 331 - CK | 0.980131 | 0.086198 | 65 | 11.37064 | 1.51E-12 | *** |
| 331 - P10 | -0.43438 | 0.086198 | 65 | -5.0393 | 0.000276 | *** |
| 331 - P2 | 0.927427 | 0.086198 | 65 | 10.7592 | 1.58E-12 | *** |
| 331 - P7 | 0.088081 | 0.086198 | 65 | 1.021834 | 0.998045 |  |
| 338 - CK | 1.643381 | 0.086198 | 65 | 19.06509 | 1.46E-12 | *** |
| 338 - P10 | 0.22887 | 0.086198 | 65 | 2.655153 | 0.300362 |  |
| 338 - P2 | 1.590677 | 0.086198 | 65 | 18.45365 | 1.46E-12 | *** |
| 338 - P7 | 0.75133 | 0.086198 | 65 | 8.716284 | 1.23E-10 | *** |
| CK - P10 | -1.41451 | 0.086198 | 65 | -16.4099 | 1.46E-12 | *** |
| CK - P2 | -0.0527 | 0.086198 | 65 | -0.61143 | 0.99999 |  |
| CK - P7 | -0.89205 | 0.086198 | 65 | -10.3488 | 1.73E-12 | *** |
| P10 - P2 | 1.361806 | 0.086198 | 65 | 15.7985 | 1.46E-12 | *** |
| P10 - P7 | 0.52246 | 0.086198 | 65 | 6.061131 | 5.63E-06 | *** |
| P2 - P7 | -0.83935 | 0.086198 | 65 | -9.73737 | 3.55E-12 | *** |

| Sample | mean_Value | SE | df | lower.CL | upper.CL | Group |
| --- | --- | --- | --- | --- | --- | --- |
| 211 | -1.6E-15 | 0.060952 | 65 | -0.18224 | 0.182237 | a |
| CK | -1.1E-16 | 0.060952 | 65 | -0.18224 | 0.182237 | a |
| P2 | 0.052705 | 0.060952 | 65 | -0.12953 | 0.234942 | a |
| 188 | 0.232649 | 0.060952 | 65 | 0.050412 | 0.414887 | ab |
| 209 | 0.35989 | 0.060952 | 65 | 0.177653 | 0.542127 | bc |
| 159 | 0.590089 | 0.060952 | 65 | 0.407852 | 0.772326 | cd |
| 207 | 0.809991 | 0.060952 | 65 | 0.627754 | 0.992229 | de |
| P7 | 0.892051 | 0.060952 | 65 | 0.709814 | 1.074288 | e |
| 167 | 0.907319 | 0.060952 | 65 | 0.725082 | 1.089556 | e |
| 331 | 0.980131 | 0.060952 | 65 | 0.797894 | 1.162368 | e |
| P10 | 1.414511 | 0.060952 | 65 | 1.232274 | 1.596748 | f |
| 338 | 1.643381 | 0.060952 | 65 | 1.461144 | 1.825618 | fg |
| 213 | 1.732999 | 0.060952 | 65 | 1.550762 | 1.915236 | g |

Phenolic acids and other host compounds

**Figure 2**

Comparison of fungal biomass (**Figure 2 a, left heatmap**)

**Gallic acid**

| Info | Value |
| --- | --- |
| Best model family | gaussian |
| Model name | Sqrt_Normal |

| Effect | Df | Deviance | Resid. Df | Resid. Dev | Pr(>Chi) |
| --- | --- | --- | --- | --- | --- |
| NULL |  |  | 28 | 277.8698 |  |
| Sample | 4 | 258.4392 | 24 | 19.43058 | 7.75E-68 |

| Comparison | Estimate | SE | df | t.ratio | p_value | Significance |
| --- | --- | --- | --- | --- | --- | --- |
| P10 - P159 | 7.148676 | 0.519489 | 24 | 13.76097 | 6.72E-12 | *** |
| P10 - P209 | 5.838157 | 0.544845 | 24 | 10.71526 | 1.21E-09 | *** |
| P10 - P331 | 8.46815 | 0.519489 | 24 | 16.30091 | 1.97E-13 | *** |
| P10 - P7 | 6.620552 | 0.519489 | 24 | 12.74435 | 3.44E-11 | *** |
| P159 - P209 | -1.31052 | 0.544845 | 24 | -2.40531 | 0.148214 |  |
| P159 - P331 | 1.319473 | 0.519489 | 24 | 2.539943 | 0.114821 |  |
| P159 - P7 | -0.52812 | 0.519489 | 24 | -1.01662 | 0.845218 |  |
| P209 - P331 | 2.629993 | 0.544845 | 24 | 4.827048 | 0.000566 | *** |
| P209 - P7 | 0.782396 | 0.544845 | 24 | 1.435997 | 0.611533 |  |
| P331 - P7 | -1.8476 | 0.519489 | 24 | -3.55656 | 0.012604 | * |

| Sample | mean_Value | SE | df | lower.CL | upper.CL | Group |
| --- | --- | --- | --- | --- | --- | --- |
| P331 | 0 | 0.367334 | 24 | -1.02418 | 1.024181 | a |
| P159 | 1.319473 | 0.367334 | 24 | 0.295292 | 2.343654 | ab |
| P7 | 1.847597 | 0.367334 | 24 | 0.823416 | 2.871778 | b |
| P209 | 2.629993 | 0.402395 | 24 | 1.508059 | 3.751927 | b |
| P10 | 8.46815 | 0.367334 | 24 | 7.443969 | 9.492331 | c |

**Protocatechuic acid**

| **Info** | **Value** |
| --- | --- |
| Best model family | gaussian |
| Model name | Sqrt_Normal |

| **Effect** | **Df** | **Deviance** | **Resid. Df** | **Resid. Dev** | **Pr(>Chi)** |
| --- | --- | --- | --- | --- | --- |
| NULL |  |  | 28 | 89.75243 |  |
| Sample | 4 | 71.5704 | 24 | 18.18203 | 1.48E-19 |

| **Comparison** | **Estimate** | **SE** | **df** | **t.ratio** | **p_value** | **Significance** |
| --- | --- | --- | --- | --- | --- | --- |
| P10 - P159 | 3.290041 | 0.502522 | 24 | 6.54706 | 8.37E-06 | *** |
| P10 - P209 | 2.18722 | 0.527049 | 24 | 4.149933 | 0.003023 | ** |
| P10 - P331 | 4.705692 | 0.502522 | 24 | 9.364154 | 1.67E-08 | *** |
| P10 - P7 | 2.953608 | 0.502522 | 24 | 5.877571 | 4.21E-05 | *** |
| P159 - P209 | -1.10282 | 0.527049 | 24 | -2.09244 | 0.255696 |  |
| P159 - P331 | 1.415651 | 0.502522 | 24 | 2.817094 | 0.065691 |  |
| P159 - P7 | -0.33643 | 0.502522 | 24 | -0.66949 | 0.961099 |  |
| P209 - P331 | 2.518473 | 0.527049 | 24 | 4.778438 | 0.000638 | *** |
| P209 - P7 | 0.766388 | 0.527049 | 24 | 1.45411 | 0.600438 |  |
| P331 - P7 | -1.75208 | 0.502522 | 24 | -3.48658 | 0.014846 | * |

| **Sample** | **mean_Value** | **SE** | **df** | **lower.CL** | **upper.CL** | **Group** |
| --- | --- | --- | --- | --- | --- | --- |
| P331 | 8.88E-16 | 0.355337 | 24 | -0.99073 | 0.990729 | a |
| P159 | 1.415651 | 0.355337 | 24 | 0.424922 | 2.406381 | ab |
| P7 | 1.752085 | 0.355337 | 24 | 0.761355 | 2.742814 | b |
| P209 | 2.518473 | 0.389252 | 24 | 1.433183 | 3.603762 | b |
| P10 | 4.705692 | 0.355337 | 24 | 3.714963 | 5.696422 | c |

**Vanillic acid**

| **Info** | **Value** |
| --- | --- |
| Best model family | gaussian |
| Model name | Sqrt_Normal |

| **Effect** | **Df** | **Deviance** | **Resid. Df** | **Resid. Dev** | **Pr(>Chi)** |
| --- | --- | --- | --- | --- | --- |
| NULL |  |  | 28 | 217.5327 |  |
| Sample | 4 | 136.3867 | 24 | 81.14594 | 3.68E-08 |

| **Comparison** | **Estimate** | **SE** | **df** | **t.ratio** | **p_value** | **Significance** |
| --- | --- | --- | --- | --- | --- | --- |
| P10 - P159 | 4.108764 | 1.061615 | 24 | 3.870295 | 0.005968 | ** |
| P10 - P209 | 3.918957 | 1.113431 | 24 | 3.519711 | 0.013741 | * |
| P10 - P331 | 0.722104 | 1.061615 | 24 | 0.680193 | 0.958857 |  |
| P10 - P7 | 5.596878 | 1.061615 | 24 | 5.27204 | 0.000187 | *** |
| P159 - P209 | -0.18981 | 1.113431 | 24 | -0.17047 | 0.999796 |  |
| P159 - P331 | -3.38666 | 1.061615 | 24 | -3.1901 | 0.02926 | * |
| P159 - P7 | 1.488114 | 1.061615 | 24 | 1.401745 | 0.632464 |  |
| P209 - P331 | -3.19685 | 1.113431 | 24 | -2.87117 | 0.05864 |  |
| P209 - P7 | 1.677921 | 1.113431 | 24 | 1.506982 | 0.568054 |  |
| P331 - P7 | 4.874774 | 1.061615 | 24 | 4.591846 | 0.001015 | ** |

| **Sample** | **mean_Value** | **SE** | **df** | **lower.CL** | **upper.CL** | **Group** |
| --- | --- | --- | --- | --- | --- | --- |
| P7 | 0.785272 | 0.750675 | 24 | -1.30772 | 2.878263 | a |
| P159 | 2.273386 | 0.750675 | 24 | 0.180396 | 4.366376 | a |
| P209 | 2.463194 | 0.822324 | 24 | 0.170438 | 4.75595 | ab |
| P331 | 5.660047 | 0.750675 | 24 | 3.567056 | 7.753037 | bc |
| P10 | 6.38215 | 0.750675 | 24 | 4.28916 | 8.475141 | c |

**Catechin**

| **Info** | **Value** |
| --- | --- |
| Best model family | gaussian |
| Model name | Sqrt_Normal |

| **Effect** | **Df** | **Deviance** | **Resid. Df** | **Resid. Dev** | **Pr(>Chi)** |
| --- | --- | --- | --- | --- | --- |
| NULL |  |  | 28 | 140.3594 |  |
| Sample | 4 | 105.2573 | 24 | 35.10218 | 8.72E-15 |

| **Comparison** | **Estimate** | **SE** | **df** | **t.ratio** | **p_value** | **Significance** |
| --- | --- | --- | --- | --- | --- | --- |
| P10 - P159 | 5.219512 | 0.698234 | 24 | 7.475309 | 9.66E-07 | *** |
| P10 - P209 | 3.417934 | 0.732314 | 24 | 4.667309 | 0.000842 | *** |
| P10 - P331 | 5.002787 | 0.698234 | 24 | 7.164918 | 1.97E-06 | *** |
| P10 - P7 | 3.770819 | 0.698234 | 24 | 5.400512 | 0.000136 | *** |
| P159 - P209 | -1.80158 | 0.732314 | 24 | -2.46012 | 0.133767 |  |
| P159 - P331 | -0.21673 | 0.698234 | 24 | -0.31039 | 0.997838 |  |
| P159 - P7 | -1.44869 | 0.698234 | 24 | -2.0748 | 0.263102 |  |
| P209 - P331 | 1.584853 | 0.732314 | 24 | 2.164173 | 0.227098 |  |
| P209 - P7 | 0.352885 | 0.732314 | 24 | 0.481877 | 0.988316 |  |
| P331 - P7 | -1.23197 | 0.698234 | 24 | -1.76441 | 0.41617 |  |

| **Sample** | **mean_Value** | **SE** | **df** | **lower.CL** | **upper.CL** | **Group** |
| --- | --- | --- | --- | --- | --- | --- |
| P159 | 0.397911 | 0.493726 | 24 | -0.97867 | 1.774489 | a |
| P331 | 0.614636 | 0.493726 | 24 | -0.76194 | 1.991214 | a |
| P7 | 1.846604 | 0.493726 | 24 | 0.470026 | 3.223182 | a |
| P209 | 2.19949 | 0.540849 | 24 | 0.691524 | 3.707455 | a |
| P10 | 5.617424 | 0.493726 | 24 | 4.240846 | 6.994002 | b |

**Caffeic acid**

| **Info** | **Value** |
| --- | --- |
| Best model family | gaussian |
| Model name | Sqrt_Normal |

| **Effect** | **Df** | **Deviance** | **Resid. Df** | **Resid. Dev** | **Pr(>Chi)** |
| --- | --- | --- | --- | --- | --- |
| NULL |  |  | 28 | 49.5427 |  |
| Sample | 4 | 32.00671 | 24 | 17.53599 | 7.04E-09 |

| **Comparison** | **Estimate** | **SE** | **df** | **t.ratio** | **p_value** | **Significance** |
| --- | --- | --- | --- | --- | --- | --- |
| P10 - P159 | 1.629096 | 0.493513 | 24 | 3.301017 | 0.022773 | * |
| P10 - P209 | -0.24906 | 0.517601 | 24 | -0.48119 | 0.988378 |  |
| P10 - P331 | 2.430206 | 0.493513 | 24 | 4.924296 | 0.000444 | *** |
| P10 - P7 | 1.869615 | 0.493513 | 24 | 3.788378 | 0.007268 | ** |
| P159 - P209 | -1.87816 | 0.517601 | 24 | -3.62858 | 0.010636 | * |
| P159 - P331 | 0.80111 | 0.493513 | 24 | 1.623279 | 0.497678 |  |
| P159 - P7 | 0.240519 | 0.493513 | 24 | 0.487361 | 0.987809 |  |
| P209 - P331 | 2.679268 | 0.517601 | 24 | 5.176318 | 0.000237 | *** |
| P209 - P7 | 2.118678 | 0.517601 | 24 | 4.093263 | 0.003472 | ** |
| P331 - P7 | -0.56059 | 0.493513 | 24 | -1.13592 | 0.786167 |  |

| **Sample** | **mean_Value** | **SE** | **df** | **lower.CL** | **upper.CL** | **Group** |
| --- | --- | --- | --- | --- | --- | --- |
| P331 | 8.88E-16 | 0.348967 | 24 | -0.97297 | 0.972969 | a |
| P7 | 0.56059 | 0.348967 | 24 | -0.41238 | 1.533559 | a |
| P159 | 0.80111 | 0.348967 | 24 | -0.17186 | 1.774079 | a |
| P10 | 2.430206 | 0.348967 | 24 | 1.457237 | 3.403174 | b |
| P209 | 2.679268 | 0.382274 | 24 | 1.613434 | 3.745102 | b |

**Syringic acid**

| **Info** | **Value** |
| --- | --- |
| Best model family | gaussian |
| Model name | Sqrt_Normal |

| **Effect** | **Df** | **Deviance** | **Resid. Df** | **Resid. Dev** | **Pr(>Chi)** |
| --- | --- | --- | --- | --- | --- |
| NULL |  |  | 28 | 322.9934 |  |
| Sample | 4 | 254.082 | 24 | 68.91136 | 2.76E-18 |

| **Comparison** | **Estimate** | **SE** | **df** | **t.ratio** | **p_value** | **Significance** |
| --- | --- | --- | --- | --- | --- | --- |
| P10 - P159 | 6.695707 | 0.978316 | 24 | 6.844114 | 4.15E-06 | *** |
| P10 - P209 | 5.840711 | 1.026066 | 24 | 5.692332 | 6.63E-05 | *** |
| P10 - P331 | 4.04319 | 0.978316 | 24 | 4.132806 | 0.003152 | ** |
| P10 - P7 | 8.601033 | 0.978316 | 24 | 8.791671 | 5.44E-08 | *** |
| P159 - P209 | -0.855 | 1.026066 | 24 | -0.83327 | 0.917574 |  |
| P159 - P331 | -2.65252 | 0.978316 | 24 | -2.71131 | 0.08169 |  |
| P159 - P7 | 1.905326 | 0.978316 | 24 | 1.947557 | 0.320788 |  |
| P209 - P331 | -1.79752 | 1.026066 | 24 | -1.75186 | 0.423176 |  |
| P209 - P7 | 2.760322 | 1.026066 | 24 | 2.690198 | 0.085264 |  |
| P331 - P7 | 4.557843 | 0.978316 | 24 | 4.658865 | 0.00086 | *** |

| **Sample** | **mean_Value** | **SE** | **df** | **lower.CL** | **upper.CL** | **Group** |
| --- | --- | --- | --- | --- | --- | --- |
| P7 | 1.78E-15 | 0.691774 | 24 | -1.92876 | 1.928764 | a |
| P159 | 1.905326 | 0.691774 | 24 | -0.02344 | 3.834091 | ab |
| P209 | 2.760322 | 0.7578 | 24 | 0.647466 | 4.873177 | ab |
| P331 | 4.557843 | 0.691774 | 24 | 2.629078 | 6.486607 | b |
| P10 | 8.601033 | 0.691774 | 24 | 6.672268 | 10.5298 | c |

**Quinic acid**

| **Info** | **Value** |
| --- | --- |
| Best model family | gaussian |
| Model name | Sqrt_Normal |

| **Effect** | **Df** | **Deviance** | **Resid. Df** | **Resid. Dev** | **Pr(>Chi)** |
| --- | --- | --- | --- | --- | --- |
| NULL |  |  | 28 | 326.535 |  |
| Sample | 4 | 317.5911 | 24 | 8.94386 | 3.7E-183 |

| **Comparison** | **Estimate** | **SE** | **df** | **t.ratio** | **p_value** | **Significance** |
| --- | --- | --- | --- | --- | --- | --- |
| P10 - P159 | -3.90277 | 0.352449 | 24 | -11.0733 | 6.24E-10 | *** |
| P10 - P209 | -6.59744 | 0.369652 | 24 | -17.8477 | 4.53E-14 | *** |
| P10 - P331 | -7.79435 | 0.352449 | 24 | -22.1148 | 2.12E-14 | *** |
| P10 - P7 | 0.252763 | 0.352449 | 24 | 0.717161 | 0.950482 |  |
| P159 - P209 | -2.69467 | 0.369652 | 24 | -7.28977 | 1.47E-06 | *** |
| P159 - P331 | -3.89158 | 0.352449 | 24 | -11.0416 | 6.61E-10 | *** |
| P159 - P7 | 4.155528 | 0.352449 | 24 | 11.79044 | 1.74E-10 | *** |
| P209 - P331 | -1.19691 | 0.369652 | 24 | -3.23794 | 0.026275 | * |
| P209 - P7 | 6.850201 | 0.369652 | 24 | 18.53151 | 3.12E-14 | *** |
| P331 - P7 | 8.047112 | 0.352449 | 24 | 22.83199 | 2.11E-14 | *** |

| **Sample** | **mean_Value** | **SE** | **df** | **lower.CL** | **upper.CL** | **Group** |
| --- | --- | --- | --- | --- | --- | --- |
| P7 | 3.33E-16 | 0.249219 | 24 | -0.69486 | 0.694858 | a |
| P10 | 0.252763 | 0.249219 | 24 | -0.4421 | 0.947621 | a |
| P159 | 4.155528 | 0.249219 | 24 | 3.46067 | 4.850386 | b |
| P209 | 6.850201 | 0.273006 | 24 | 6.089022 | 7.611381 | c |
| P331 | 8.047112 | 0.249219 | 24 | 7.352253 | 8.74197 | d |

**Ferulic acid**

| **Info** | **Value** |
| --- | --- |
| Best model family | gaussian |
| Model name | Sqrt_Normal |

| **Effect** | **Df** | **Deviance** | **Resid. Df** | **Resid. Dev** | **Pr(>Chi)** |
| --- | --- | --- | --- | --- | --- |
| NULL |  |  | 28 | 45.38015 |  |
| Sample | 4 | 36.43758 | 24 | 8.942575 | 2.9E-20 |

| **Comparison** | **Estimate** | **SE** | **df** | **t.ratio** | **p_value** | **Significance** |
| --- | --- | --- | --- | --- | --- | --- |
| P10 - P159 | 2.806924 | 0.352424 | 24 | 7.96463 | 3.23E-07 | *** |
| P10 - P209 | 1.72672 | 0.369625 | 24 | 4.671546 | 0.000833 | *** |
| P10 - P331 | 2.799083 | 0.352424 | 24 | 7.942382 | 3.39E-07 | *** |
| P10 - P7 | 2.855785 | 0.352424 | 24 | 8.103274 | 2.38E-07 | *** |
| P159 - P209 | -1.0802 | 0.369625 | 24 | -2.92243 | 0.052591 |  |
| P159 - P331 | -0.00784 | 0.352424 | 24 | -0.02225 | 1 |  |
| P159 - P7 | 0.048861 | 0.352424 | 24 | 0.138643 | 0.99991 |  |
| P209 - P331 | 1.072363 | 0.369625 | 24 | 2.901218 | 0.055022 |  |
| P209 - P7 | 1.129065 | 0.369625 | 24 | 3.054622 | 0.039509 | * |
| P331 - P7 | 0.056702 | 0.352424 | 24 | 0.160892 | 0.999838 |  |

| **Sample** | **mean_Value** | **SE** | **df** | **lower.CL** | **upper.CL** | **Group** |
| --- | --- | --- | --- | --- | --- | --- |
| P7 | 1.397806 | 0.249201 | 24 | 0.702998 | 2.092614 | a |
| P159 | 1.446667 | 0.249201 | 24 | 0.751859 | 2.141476 | ab |
| P331 | 1.454508 | 0.249201 | 24 | 0.7597 | 2.149316 | ab |
| P209 | 2.526871 | 0.272986 | 24 | 1.765746 | 3.287996 | b |
| P10 | 4.253591 | 0.249201 | 24 | 3.558783 | 4.9484 | c |

Comparison of phenolic acids and other host compounds in culture substrate

**Figure 2**

Comparison of medium (**Figure 2 a, right heatmap**)

**Vanillic acid**

| Info | Value |
| --- | --- |
| Best model family | gaussian |
| Model name | Sqrt_Normal |

| Effect | Df | Deviance | Resid. Df | Resid. Dev | Pr(>Chi) |
| --- | --- | --- | --- | --- | --- |
| NULL |  |  | 35 | 218.1081 |  |
| Sample | 5 | 203.6631 | 30 | 14.44499 | 3.31E-89 |

| Comparison | Estimate | SE | df | t.ratio | p_value | Significance |
| --- | --- | --- | --- | --- | --- | --- |
| Control - P10 | -2.6245 | 0.400624 | 30 | -6.55103 | 4.25E-06 | *** |
| Control - P159 | 0.357789 | 0.400624 | 30 | 0.893077 | 0.945218 |  |
| Control - P209 | 3.515136 | 0.400624 | 30 | 8.774143 | 1.27E-08 | *** |
| Control - P331 | -0.09341 | 0.400624 | 30 | -0.23316 | 0.999895 |  |
| Control - P7 | 4.449494 | 0.400624 | 30 | 11.1064 | 5.5E-11 | *** |
| P10 - P159 | 2.982292 | 0.400624 | 30 | 7.444108 | 3.85E-07 | *** |
| P10 - P209 | 6.139639 | 0.400624 | 30 | 15.32517 | 6.1E-14 | *** |
| P10 - P331 | 2.531092 | 0.400624 | 30 | 6.317867 | 8.06E-06 | *** |
| P10 - P7 | 7.073997 | 0.400624 | 30 | 17.65743 | 4.65E-14 | *** |
| P159 - P209 | 3.157348 | 0.400624 | 30 | 7.881066 | 1.23E-07 | *** |
| P159 - P331 | -0.4512 | 0.400624 | 30 | -1.12624 | 0.866593 |  |
| P159 - P7 | 4.091706 | 0.400624 | 30 | 10.21332 | 4.07E-10 | *** |
| P209 - P331 | -3.60855 | 0.400624 | 30 | -9.00731 | 7.14E-09 | *** |
| P209 - P7 | 0.934358 | 0.400624 | 30 | 2.332254 | 0.212952 |  |
| P331 - P7 | 4.542905 | 0.400624 | 30 | 11.33956 | 3.32E-11 | *** |

| Sample | mean_Value | SE | df | lower.CL | upper.CL | Group |
| --- | --- | --- | --- | --- | --- | --- |
| P7 | 2.66E-15 | 0.283284 | 30 | -0.79774 | 0.797736 | a |
| P209 | 0.934358 | 0.283284 | 30 | 0.136622 | 1.732094 | a |
| P159 | 4.091706 | 0.283284 | 30 | 3.29397 | 4.889441 | b |
| Control | 4.449494 | 0.283284 | 30 | 3.651758 | 5.24723 | b |
| P331 | 4.542905 | 0.283284 | 30 | 3.745169 | 5.340641 | b |
| P10 | 7.073997 | 0.283284 | 30 | 6.276261 | 7.871733 | c |

**Protocatechuic acid**

| Info | Value |
| --- | --- |
| Best model family | gaussian |
| Model name | Normal_log |

| Effect | Df | Deviance | Resid. Df | Resid. Dev | Pr(>Chi) |
| --- | --- | --- | --- | --- | --- |
| NULL |  |  | 35 | 79.19898 |  |
| Sample | 5 | 75.29854 | 30 | 3.900444 | 6.5E-123 |

| Comparison | Estimate | SE | df | t.ratio | p_value | Significance |
| --- | --- | --- | --- | --- | --- | --- |
| Control - P10 | 0.106103 | 0.208178 | 30 | 0.509675 | 0.995401 |  |
| Control - P159 | 1.207035 | 0.208178 | 30 | 5.798077 | 3.39E-05 | *** |
| Control - P209 | 2.742684 | 0.208178 | 30 | 13.17468 | 8.25E-13 | *** |
| Control - P331 | 2.169611 | 0.208178 | 30 | 10.42188 | 2.53E-10 | *** |
| Control - P7 | 4.060027 | 0.208178 | 30 | 19.50263 | 4.61E-14 | *** |
| P10 - P159 | 1.100931 | 0.208178 | 30 | 5.288402 | 0.000139 | *** |
| P10 - P209 | 2.636581 | 0.208178 | 30 | 12.665 | 2.16E-12 | *** |
| P10 - P331 | 2.063507 | 0.208178 | 30 | 9.912204 | 8.18E-10 | *** |
| P10 - P7 | 3.953924 | 0.208178 | 30 | 18.99295 | 4.61E-14 | *** |
| P159 - P209 | 1.53565 | 0.208178 | 30 | 7.376602 | 4.6E-07 | *** |
| P159 - P331 | 0.962576 | 0.208178 | 30 | 4.623802 | 0.000874 | *** |
| P159 - P7 | 2.852992 | 0.208178 | 30 | 13.70455 | 3.31E-13 | *** |
| P209 - P331 | -0.57307 | 0.208178 | 30 | -2.7528 | 0.093985 |  |
| P209 - P7 | 1.317343 | 0.208178 | 30 | 6.327949 | 7.84E-06 | *** |
| P331 - P7 | 1.890416 | 0.208178 | 30 | 9.080749 | 5.96E-09 | *** |

| Sample | mean_Value | SE | df | lower.CL | upper.CL | Group |
| --- | --- | --- | --- | --- | --- | --- |
| P7 | 0.628677 | 0.147204 | 30 | 0.415335 | 0.951605 | a |
| P209 | 2.347159 | 0.147204 | 30 | 1.55065 | 3.552803 | b |
| P331 | 4.163179 | 0.147204 | 30 | 2.750403 | 6.301644 | b |
| P159 | 10.90101 | 0.147204 | 30 | 7.201746 | 16.50043 | c |
| P10 | 32.77895 | 0.147204 | 30 | 21.6554 | 49.61623 | d |
| Control | 36.44812 | 0.147204 | 30 | 24.07944 | 55.17011 | d |

**Gallic acid**

| Info | Value |
| --- | --- |
| Best model family | gaussian |
| Model name | Normal_log |

| Effect | Df | Deviance | Resid. Df | Resid. Dev | Pr(>Chi) |
| --- | --- | --- | --- | --- | --- |
| NULL |  |  | 35 | 218.8917 |  |
| Sample | 5 | 211.6285 | 30 | 7.263245 | 1.1E-186 |

| Comparison | Estimate | SE | df | t.ratio | p_value | Significance |
| --- | --- | --- | --- | --- | --- | --- |
| Control - P10 | -0.36162 | 0.284082 | 30 | -1.27296 | 0.796969 |  |
| Control - P159 | 0.737178 | 0.284082 | 30 | 2.594945 | 0.129748 |  |
| Control - P209 | 4.744931 | 0.284082 | 30 | 16.70267 | 4.76E-14 | *** |
| Control - P331 | 4.609584 | 0.284082 | 30 | 16.22623 | 4.96E-14 | *** |
| Control - P7 | 5.369946 | 0.284082 | 30 | 18.90279 | 4.61E-14 | *** |
| P10 - P159 | 1.098802 | 0.284082 | 30 | 3.8679 | 0.006603 | ** |
| P10 - P209 | 5.106555 | 0.284082 | 30 | 17.97562 | 4.63E-14 | *** |
| P10 - P331 | 4.971208 | 0.284082 | 30 | 17.49918 | 4.65E-14 | *** |
| P10 - P7 | 5.73157 | 0.284082 | 30 | 20.17574 | 4.61E-14 | *** |
| P159 - P209 | 4.007753 | 0.284082 | 30 | 14.10772 | 1.8E-13 | *** |
| P159 - P331 | 3.872406 | 0.284082 | 30 | 13.63128 | 3.73E-13 | *** |
| P159 - P7 | 4.632769 | 0.284082 | 30 | 16.30784 | 4.92E-14 | *** |
| P209 - P331 | -0.13535 | 0.284082 | 30 | -0.47644 | 0.996649 |  |
| P209 - P7 | 0.625015 | 0.284082 | 30 | 2.200121 | 0.267359 |  |
| P331 - P7 | 0.760363 | 0.284082 | 30 | 2.676558 | 0.11006 |  |

| Sample | mean_Value | SE | df | lower.CL | upper.CL | Group |
| --- | --- | --- | --- | --- | --- | --- |
| P7 | 0.439429 | 0.200876 | 30 | 0.249586 | 0.773673 | a |
| P209 | 0.820974 | 0.200876 | 30 | 0.466295 | 1.445434 | a |
| P331 | 0.939961 | 0.200876 | 30 | 0.533877 | 1.654927 | a |
| P159 | 45.17254 | 0.200876 | 30 | 25.65699 | 79.53228 | b |
| Control | 94.4119 | 0.200876 | 30 | 53.62384 | 166.2247 | bc |
| P10 | 135.5433 | 0.200876 | 30 | 76.98554 | 238.642 | c |

**Caffeic acid**

| Info | Value |
| --- | --- |
| Best model family | gaussian |
| Model name | Sqrt_Normal |

| Effect | Df | Deviance | Resid. Df | Resid. Dev | Pr(>Chi) |
| --- | --- | --- | --- | --- | --- |
| NULL |  |  | 35 | 19.3338 |  |
| Sample | 5 | 17.78575 | 30 | 1.548045 | 2.45E-72 |

| Comparison | Estimate | SE | df | t.ratio | p_value | Significance |
| --- | --- | --- | --- | --- | --- | --- |
| Control - P10 | -1.46754 | 0.131151 | 30 | -11.1897 | 4.59E-11 | *** |
| Control - P159 | -0.40137 | 0.131151 | 30 | -3.06037 | 0.04791 | * |
| Control - P209 | 0.590305 | 0.131151 | 30 | 4.500965 | 0.001222 | ** |
| Control - P331 | 0.151946 | 0.131151 | 30 | 1.158563 | 0.852492 |  |
| Control - P7 | 0.573553 | 0.131151 | 30 | 4.373238 | 0.001728 | ** |
| P10 - P159 | 1.066169 | 0.131151 | 30 | 8.129347 | 6.46E-08 | *** |
| P10 - P209 | 2.057844 | 0.131151 | 30 | 15.69068 | 5.41E-14 | *** |
| P10 - P331 | 1.619486 | 0.131151 | 30 | 12.34828 | 4.05E-12 | *** |
| P10 - P7 | 2.041092 | 0.131151 | 30 | 15.56296 | 5.61E-14 | *** |
| P159 - P209 | 0.991674 | 0.131151 | 30 | 7.561337 | 2.83E-07 | *** |
| P159 - P331 | 0.553316 | 0.131151 | 30 | 4.218935 | 0.002616 | ** |
| P159 - P7 | 0.974923 | 0.131151 | 30 | 7.43361 | 3.96E-07 | *** |
| P209 - P331 | -0.43836 | 0.131151 | 30 | -3.3424 | 0.024706 | * |
| P209 - P7 | -0.01675 | 0.131151 | 30 | -0.12773 | 0.999995 |  |
| P331 - P7 | 0.421607 | 0.131151 | 30 | 3.214675 | 0.033504 | * |

| Sample | mean_Value | SE | df | lower.CL | upper.CL | Group |
| --- | --- | --- | --- | --- | --- | --- |
| P209 | 0.473008 | 0.092738 | 30 | 0.211856 | 0.734159 | a |
| P7 | 0.489759 | 0.092738 | 30 | 0.228608 | 0.750911 | a |
| P331 | 0.911366 | 0.092738 | 30 | 0.650215 | 1.172517 | b |
| Control | 1.063312 | 0.092738 | 30 | 0.802161 | 1.324464 | b |
| P159 | 1.464682 | 0.092738 | 30 | 1.203531 | 1.725834 | c |
| P10 | 2.530852 | 0.092738 | 30 | 2.2697 | 2.792003 | d |

**Quinic acid**

| Info | Value |
| --- | --- |
| Best model family | gaussian |
| Model name | Normal_log |

| Effect | Df | Deviance | Resid. Df | Resid. Dev | Pr(>Chi) |
| --- | --- | --- | --- | --- | --- |
| NULL |  |  | 35 | 12.06126 |  |
| Sample | 5 | 7.822186 | 30 | 4.239073 | 1.1E-10 |

| Comparison | Estimate | SE | df | t.ratio | p_value | Significance |
| --- | --- | --- | --- | --- | --- | --- |
| Control - P10 | -0.42683 | 0.217027 | 30 | -1.96669 | 0.384045 |  |
| Control - P159 | 0.669417 | 0.217027 | 30 | 3.084484 | 0.045342 | * |
| Control - P209 | 0.415911 | 0.217027 | 30 | 1.916402 | 0.412263 |  |
| Control - P331 | 1.034169 | 0.217027 | 30 | 4.765156 | 0.000593 | *** |
| Control - P7 | 0.407289 | 0.217027 | 30 | 1.876674 | 0.435199 |  |
| P10 - P159 | 1.096242 | 0.217027 | 30 | 5.051174 | 0.000269 | *** |
| P10 - P209 | 0.842737 | 0.217027 | 30 | 3.883092 | 0.006348 | ** |
| P10 - P331 | 1.460994 | 0.217027 | 30 | 6.731846 | 2.6E-06 | *** |
| P10 - P7 | 0.834115 | 0.217027 | 30 | 3.843364 | 0.007037 | ** |
| P159 - P209 | -0.25351 | 0.217027 | 30 | -1.16808 | 0.848199 |  |
| P159 - P331 | 0.364752 | 0.217027 | 30 | 1.680672 | 0.554396 |  |
| P159 - P7 | -0.26213 | 0.217027 | 30 | -1.20781 | 0.829619 |  |
| P209 - P331 | 0.618257 | 0.217027 | 30 | 2.848754 | 0.076632 |  |
| P209 - P7 | -0.00862 | 0.217027 | 30 | -0.03973 | 1 |  |
| P331 - P7 | -0.62688 | 0.217027 | 30 | -2.88848 | 0.070304 |  |

| Sample | mean_Value | SE | df | lower.CL | upper.CL | Group |
| --- | --- | --- | --- | --- | --- | --- |
| P331 | 3.354565 | 0.153461 | 30 | 2.177485 | 5.167937 | a |
| P159 | 4.831098 | 0.153461 | 30 | 3.135919 | 7.442636 | a |
| P209 | 6.225037 | 0.153461 | 30 | 4.04074 | 9.590094 | ab |
| P7 | 6.278941 | 0.153461 | 30 | 4.07573 | 9.673138 | ab |
| Control | 9.435609 | 0.153461 | 30 | 6.124759 | 14.5362 | bc |
| P10 | 14.45898 | 0.153461 | 30 | 9.385487 | 22.27505 | c |

**Ferulic acid**

| Info | Value |
| --- | --- |
| Best model family | gaussian |
| Model name | Sqrt_Normal |

| Effect | Df | Deviance | Resid. Df | Resid. Dev | Pr(>Chi) |
| --- | --- | --- | --- | --- | --- |
| NULL |  |  | 35 | 199.4895 |  |
| Sample | 5 | 194.0646 | 30 | 5.424871 | 8.5E-230 |

| Comparison | Estimate | SE | df | t.ratio | p_value | Significance |
| --- | --- | --- | --- | --- | --- | --- |
| Control - P10 | -5.15708 | 0.245512 | 30 | -21.0054 | 4.61E-14 | *** |
| Control - P159 | -2.28528 | 0.245512 | 30 | -9.3082 | 3.43E-09 | *** |
| Control - P209 | 1.218387 | 0.245512 | 30 | 4.962627 | 0.000344 | *** |
| Control - P331 | 0.665599 | 0.245512 | 30 | 2.71106 | 0.102521 |  |
| Control - P7 | 1.282763 | 0.245512 | 30 | 5.22484 | 0.000166 | *** |
| P10 - P159 | 2.871797 | 0.245512 | 30 | 11.69716 | 1.55E-11 | *** |
| P10 - P209 | 6.375463 | 0.245512 | 30 | 25.96799 | 4.61E-14 | *** |
| P10 - P331 | 5.822675 | 0.245512 | 30 | 23.71642 | 4.61E-14 | *** |
| P10 - P7 | 6.439839 | 0.245512 | 30 | 26.2302 | 4.61E-14 | *** |
| P159 - P209 | 3.503666 | 0.245512 | 30 | 14.27083 | 1.45E-13 | *** |
| P159 - P331 | 2.950878 | 0.245512 | 30 | 12.01926 | 7.91E-12 | *** |
| P159 - P7 | 3.568042 | 0.245512 | 30 | 14.53304 | 1.08E-13 | *** |
| P209 - P331 | -0.55279 | 0.245512 | 30 | -2.25157 | 0.245144 |  |
| P209 - P7 | 0.064376 | 0.245512 | 30 | 0.262212 | 0.999813 |  |
| P331 - P7 | 0.617164 | 0.245512 | 30 | 2.51378 | 0.152085 |  |

| Sample | mean_Value | SE | df | lower.CL | upper.CL | Group |
| --- | --- | --- | --- | --- | --- | --- |
| P7 | 0.475637 | 0.173603 | 30 | -0.01323 | 0.964509 | a |
| P209 | 0.540013 | 0.173603 | 30 | 0.051141 | 1.028886 | a |
| P331 | 1.092801 | 0.173603 | 30 | 0.603929 | 1.581673 | ab |
| Control | 1.7584 | 0.173603 | 30 | 1.269528 | 2.247272 | b |
| P159 | 4.043679 | 0.173603 | 30 | 3.554807 | 4.532551 | c |
| P10 | 6.915477 | 0.173603 | 30 | 6.426604 | 7.404349 | d |

**Syringic acid**

| Info | Value |
| --- | --- |
| Best model family | gaussian |
| Model name | Sqrt_Normal |

| Effect | Df | Deviance | Resid. Df | Resid. Dev | Pr(>Chi) |
| --- | --- | --- | --- | --- | --- |
| NULL |  |  | 35 | 467.106 |  |
| Sample | 5 | 450.7471 | 30 | 16.35893 | 2E-176 |

| Comparison | Estimate | SE | df | t.ratio | p_value | Significance |
| --- | --- | --- | --- | --- | --- | --- |
| Control - P10 | -4.04115 | 0.42634 | 30 | -9.47871 | 2.28E-09 | *** |
| Control - P159 | 0.257476 | 0.42634 | 30 | 0.603922 | 0.989938 |  |
| Control - P209 | 5.583327 | 0.42634 | 30 | 13.09595 | 9.53E-13 | *** |
| Control - P331 | 3.355435 | 0.42634 | 30 | 7.870325 | 1.26E-07 | *** |
| Control - P7 | 6.123341 | 0.42634 | 30 | 14.36257 | 1.3E-13 | *** |
| P10 - P159 | 4.298629 | 0.42634 | 30 | 10.08263 | 5.5E-10 | *** |
| P10 - P209 | 9.62448 | 0.42634 | 30 | 22.57465 | 4.61E-14 | *** |
| P10 - P331 | 7.396588 | 0.42634 | 30 | 17.34903 | 4.65E-14 | *** |
| P10 - P7 | 10.16449 | 0.42634 | 30 | 23.84128 | 4.61E-14 | *** |
| P159 - P209 | 5.325851 | 0.42634 | 30 | 12.49202 | 3.04E-12 | *** |
| P159 - P331 | 3.097959 | 0.42634 | 30 | 7.266403 | 6.17E-07 | *** |
| P159 - P7 | 5.865864 | 0.42634 | 30 | 13.75865 | 3.03E-13 | *** |
| P209 - P331 | -2.22789 | 0.42634 | 30 | -5.22562 | 0.000166 | *** |
| P209 - P7 | 0.540013 | 0.42634 | 30 | 1.266626 | 0.800253 |  |
| P331 - P7 | 2.767906 | 0.42634 | 30 | 6.492248 | 4.99E-06 | *** |

| Sample | mean_Value | SE | df | lower.CL | upper.CL | Group |
| --- | --- | --- | --- | --- | --- | --- |
| P7 | 1.78E-15 | 0.301468 | 30 | -0.84894 | 0.848942 | a |
| P209 | 0.540013 | 0.301468 | 30 | -0.30893 | 1.388955 | a |
| P331 | 2.767906 | 0.301468 | 30 | 1.918964 | 3.616847 | b |
| P159 | 5.865864 | 0.301468 | 30 | 5.016923 | 6.714806 | c |
| Control | 6.123341 | 0.301468 | 30 | 5.274399 | 6.972282 | c |
| P10 | 10.16449 | 0.301468 | 30 | 9.315552 | 11.01344 | d |

**Catechin**

| Info | Value |
| --- | --- |
| Best model family | gaussian |
| Model name | Sqrt_Normal |

| Effect | Df | Deviance | Resid. Df | Resid. Dev | Pr(>Chi) |
| --- | --- | --- | --- | --- | --- |
| NULL |  |  | 35 | 396.5485 |  |
| Sample | 5 | 376.5871 | 30 | 19.96138 | 4.5E-120 |

| Comparison | Estimate | SE | df | t.ratio | p_value | Significance |
| --- | --- | --- | --- | --- | --- | --- |
| Control - P10 | -2.23519 | 0.470949 | 30 | -4.74613 | 0.000625 | *** |
| Control - P159 | 2.477582 | 0.470949 | 30 | 5.260826 | 0.000151 | *** |
| Control - P209 | 5.600777 | 0.470949 | 30 | 11.89253 | 1.03E-11 | *** |
| Control - P331 | 5.363723 | 0.470949 | 30 | 11.38918 | 2.98E-11 | *** |
| Control - P7 | 6.706003 | 0.470949 | 30 | 14.23933 | 1.51E-13 | *** |
| P10 - P159 | 4.712768 | 0.470949 | 30 | 10.00696 | 6.56E-10 | *** |
| P10 - P209 | 7.835963 | 0.470949 | 30 | 16.63866 | 4.76E-14 | *** |
| P10 - P331 | 7.598909 | 0.470949 | 30 | 16.13531 | 5.01E-14 | *** |
| P10 - P7 | 8.941189 | 0.470949 | 30 | 18.98546 | 4.61E-14 | *** |
| P159 - P209 | 3.123195 | 0.470949 | 30 | 6.631703 | 3.41E-06 | *** |
| P159 - P331 | 2.886141 | 0.470949 | 30 | 6.128349 | 1.36E-05 | *** |
| P159 - P7 | 4.228421 | 0.470949 | 30 | 8.978508 | 7.66E-09 | *** |
| P209 - P331 | -0.23705 | 0.470949 | 30 | -0.50335 | 0.995661 |  |
| P209 - P7 | 1.105226 | 0.470949 | 30 | 2.346805 | 0.207489 |  |
| P331 - P7 | 1.34228 | 0.470949 | 30 | 2.850158 | 0.0764 |  |

| Sample | mean_Value | SE | df | lower.CL | upper.CL | Group |
| --- | --- | --- | --- | --- | --- | --- |
| P7 | 2.314451 | 0.333011 | 30 | 1.376682 | 3.25222 | a |
| P209 | 3.419677 | 0.333011 | 30 | 2.481908 | 4.357446 | a |
| P331 | 3.656731 | 0.333011 | 30 | 2.718962 | 4.5945 | a |
| P159 | 6.542872 | 0.333011 | 30 | 5.605104 | 7.480641 | b |
| Control | 9.020454 | 0.333011 | 30 | 8.082685 | 9.958223 | c |
| P10 | 11.25564 | 0.333011 | 30 | 10.31787 | 12.19341 | d |

Phenolic acids and other host compounds in medium over time

**Figure 2 a** (right heatmap, comparison of beech sawdust medium with *A. hylecoeti* culture medium) and **Figure 2 b** and **Suppl. Fig. 12**

**Vanillic acid**

| **Info** | **Value** |
| --- | --- |
| Best model family | gaussian |
| Model name | Normal_log |

| **Effect** | **Df** | **Deviance** | **Resid. Df** | **Resid. Dev** | **Pr(>Chi)** |
| --- | --- | --- | --- | --- | --- |
| NULL |  |  | 47 | 5.823448 |  |
| Sample | 7 | 4.793011 | 40 | 1.030438 | 1.02E-36 |

| **Comparison** | **Estimate** | **SE** | **df** | **t.ratio** | **p_value** | **Significance** |
| --- | --- | --- | --- | --- | --- | --- |
| Control - (P10-Day10) | -0.78829 | 0.092666 | 40 | -8.50684 | 4.5E-09 | *** |
| Control - (P10-Day12) | -1.05286 | 0.092666 | 40 | -11.3619 | 1.69E-12 | *** |
| Control - (P10-Day14) | -0.86598 | 0.092666 | 40 | -9.34519 | 3.61E-10 | *** |
| Control - (P10-Day17) | -0.95441 | 0.092666 | 40 | -10.2995 | 2.31E-11 | *** |
| Control - (P10-Day21) | -0.92934 | 0.092666 | 40 | -10.029 | 4.94E-11 | *** |
| Control - (P10-Day28) | -0.9875 | 0.092666 | 40 | -10.6566 | 8.76E-12 | *** |
| Control - (P10-Day7) | -0.92673 | 0.092666 | 40 | -10.0008 | 5.36E-11 | *** |
| (P10-Day10) - (P10-Day12) | -0.26456 | 0.092666 | 40 | -2.85502 | 0.1099 |  |
| (P10-Day10) - (P10-Day14) | -0.07769 | 0.092666 | 40 | -0.83835 | 0.989652 |  |
| (P10-Day10) - (P10-Day17) | -0.16611 | 0.092666 | 40 | -1.79262 | 0.62845 |  |
| (P10-Day10) - (P10-Day21) | -0.14105 | 0.092666 | 40 | -1.52213 | 0.791105 |  |
| (P10-Day10) - (P10-Day28) | -0.19921 | 0.092666 | 40 | -2.14972 | 0.402493 |  |
| (P10-Day10) - (P10-Day7) | -0.13844 | 0.092666 | 40 | -1.49393 | 0.806031 |  |
| (P10-Day12) - (P10-Day14) | 0.186877 | 0.092666 | 40 | 2.01667 | 0.484011 |  |
| (P10-Day12) - (P10-Day17) | 0.098449 | 0.092666 | 40 | 1.062402 | 0.960906 |  |
| (P10-Day12) - (P10-Day21) | 0.123514 | 0.092666 | 40 | 1.33289 | 0.880675 |  |
| (P10-Day12) - (P10-Day28) | 0.065357 | 0.092666 | 40 | 0.705297 | 0.996362 |  |
| (P10-Day12) - (P10-Day7) | 0.126127 | 0.092666 | 40 | 1.361088 | 0.869002 |  |
| (P10-Day14) - (P10-Day17) | -0.08843 | 0.092666 | 40 | -0.95427 | 0.97822 |  |
| (P10-Day14) - (P10-Day21) | -0.06336 | 0.092666 | 40 | -0.68378 | 0.997 |  |
| (P10-Day14) - (P10-Day28) | -0.12152 | 0.092666 | 40 | -1.31137 | 0.889163 |  |
| (P10-Day14) - (P10-Day7) | -0.06075 | 0.092666 | 40 | -0.65558 | 0.997696 |  |
| (P10-Day17) - (P10-Day21) | 0.025065 | 0.092666 | 40 | 0.270488 | 0.999994 |  |
| (P10-Day17) - (P10-Day28) | -0.03309 | 0.092666 | 40 | -0.3571 | 0.999958 |  |
| (P10-Day17) - (P10-Day7) | 0.027678 | 0.092666 | 40 | 0.298686 | 0.999988 |  |
| (P10-Day21) - (P10-Day28) | -0.05816 | 0.092666 | 40 | -0.62759 | 0.998252 |  |
| (P10-Day21) - (P10-Day7) | 0.002613 | 0.092666 | 40 | 0.028198 | 1 |  |
| (P10-Day28) - (P10-Day7) | 0.060769 | 0.092666 | 40 | 0.655791 | 0.997691 |  |

| **Sample** | **mean_Value** | **SE** | **df** | **lower.CL** | **upper.CL** | **Group** |
| --- | --- | --- | --- | --- | --- | --- |
| Control | 19.63516 | 0.065525 | 40 | 16.26039 | 23.71035 | a |
| P10-Day10 | 43.19032 | 0.065525 | 40 | 35.76703 | 52.15427 | b |
| P10-Day14 | 46.67938 | 0.065525 | 40 | 38.65642 | 56.36748 | b |
| P10-Day7 | 49.60307 | 0.065525 | 40 | 41.0776 | 59.89797 | b |
| P10-Day21 | 49.73285 | 0.065525 | 40 | 41.18507 | 60.05468 | b |
| P10-Day17 | 50.99516 | 0.065525 | 40 | 42.23043 | 61.57898 | b |
| P10-Day28 | 52.7109 | 0.065525 | 40 | 43.65127 | 63.65081 | b |
| P10-Day12 | 56.271 | 0.065525 | 40 | 46.59948 | 67.94979 | b |

**Protocatechuic acid**

| Info | Value |
| --- | --- |
| Best model family | gaussian |
| Model name | Normal_log |

| Effect | Df | Deviance | Resid. Df | Resid. Dev | Pr(>Chi) |
| --- | --- | --- | --- | --- | --- |
| NULL |  |  | 47 | 1.28392 |  |
| Sample | 7 | 0.463201 | 40 | 0.820719 | 0.00202 |

| Comparison | Estimate | SE | df | t.ratio | p_value | Significance |
| --- | --- | --- | --- | --- | --- | --- |
| Control - (P10-Day10) | 0.246057 | 0.0827 | 40 | 2.975286 | 0.084121 |  |
| Control - (P10-Day12) | 0.026002 | 0.0827 | 40 | 0.31441 | 0.999982 |  |
| Control - (P10-Day14) | 0.122996 | 0.0827 | 40 | 1.487254 | 0.80949 |  |
| Control - (P10-Day17) | 0.083476 | 0.0827 | 40 | 1.009377 | 0.970307 |  |
| Control - (P10-Day21) | -0.07473 | 0.0827 | 40 | -0.90362 | 0.984004 |  |
| Control - (P10-Day28) | -0.05448 | 0.0827 | 40 | -0.65875 | 0.997625 |  |
| Control - (P10-Day7) | 0.106103 | 0.0827 | 40 | 1.282987 | 0.899799 |  |
| (P10-Day10) - (P10-Day12) | -0.22006 | 0.0827 | 40 | -2.66088 | 0.164914 |  |
| (P10-Day10) - (P10-Day14) | -0.12306 | 0.0827 | 40 | -1.48803 | 0.809088 |  |
| (P10-Day10) - (P10-Day17) | -0.16258 | 0.0827 | 40 | -1.96591 | 0.516352 |  |
| (P10-Day10) - (P10-Day21) | -0.32079 | 0.0827 | 40 | -3.8789 | 0.008316 | ** |
| (P10-Day10) - (P10-Day28) | -0.30054 | 0.0827 | 40 | -3.63404 | 0.016284 | * |
| (P10-Day10) - (P10-Day7) | -0.13995 | 0.0827 | 40 | -1.6923 | 0.691939 |  |
| (P10-Day12) - (P10-Day14) | 0.096994 | 0.0827 | 40 | 1.172844 | 0.934988 |  |
| (P10-Day12) - (P10-Day17) | 0.057474 | 0.0827 | 40 | 0.694967 | 0.996681 |  |
| (P10-Day12) - (P10-Day21) | -0.10073 | 0.0827 | 40 | -1.21803 | 0.921716 |  |
| (P10-Day12) - (P10-Day28) | -0.08048 | 0.0827 | 40 | -0.97316 | 0.975707 |  |
| (P10-Day12) - (P10-Day7) | 0.080102 | 0.0827 | 40 | 0.968577 | 0.976336 |  |
| (P10-Day14) - (P10-Day17) | -0.03952 | 0.0827 | 40 | -0.47788 | 0.999703 |  |
| (P10-Day14) - (P10-Day21) | -0.19773 | 0.0827 | 40 | -2.39087 | 0.27345 |  |
| (P10-Day14) - (P10-Day28) | -0.17748 | 0.0827 | 40 | -2.14601 | 0.404687 |  |
| (P10-Day14) - (P10-Day7) | -0.01689 | 0.0827 | 40 | -0.20427 | 0.999999 |  |
| (P10-Day17) - (P10-Day21) | -0.15821 | 0.0827 | 40 | -1.913 | 0.550486 |  |
| (P10-Day17) - (P10-Day28) | -0.13795 | 0.0827 | 40 | -1.66813 | 0.706808 |  |
| (P10-Day17) - (P10-Day7) | 0.022628 | 0.0827 | 40 | 0.27361 | 0.999993 |  |
| (P10-Day21) - (P10-Day28) | 0.02025 | 0.0827 | 40 | 0.244866 | 0.999997 |  |
| (P10-Day21) - (P10-Day7) | 0.180833 | 0.0827 | 40 | 2.186605 | 0.381009 |  |
| (P10-Day28) - (P10-Day7) | 0.160582 | 0.0827 | 40 | 1.941739 | 0.531905 |  |

| Sample | mean_Value | SE | df | lower.CL | upper.CL | Group |
| --- | --- | --- | --- | --- | --- | --- |
| P10-Day10 | 28.49797 | 0.058478 | 40 | 24.08345 | 33.72168 | a |
| P10-Day14 | 32.22987 | 0.058478 | 40 | 27.23725 | 38.13763 | ab |
| P10-Day7 | 32.77895 | 0.058478 | 40 | 27.70128 | 38.78736 | ab |
| P10-Day17 | 33.52911 | 0.058478 | 40 | 28.33524 | 39.67503 | ab |
| P10-Day12 | 35.51262 | 0.058478 | 40 | 30.01148 | 42.02211 | ab |
| Control | 36.44812 | 0.058478 | 40 | 30.80207 | 43.12909 | ab |
| P10-Day28 | 38.48886 | 0.058478 | 40 | 32.52668 | 45.5439 | b |
| P10-Day21 | 39.27622 | 0.058478 | 40 | 33.19208 | 46.47559 | b |

**Gallic acid**

| Info | Value |
| --- | --- |
| Best model family | gaussian |
| Model name | Normal_log |

| Effect | Df | Deviance | Resid. Df | Resid. Dev | Pr(>Chi) |
| --- | --- | --- | --- | --- | --- |
| NULL |  |  | 47 | 6.588299 |  |
| Sample | 7 | 4.900311 | 40 | 1.687989 | 4.91E-22 |

| Comparison | Estimate | SE | df | t.ratio | p_value | Significance |
| --- | --- | --- | --- | --- | --- | --- |
| Control - (P10-Day10) | -0.12605 | 0.118603 | 40 | -1.06276 | 0.960837 |  |
| Control - (P10-Day12) | -0.25019 | 0.118603 | 40 | -2.1095 | 0.426525 |  |
| Control - (P10-Day14) | -0.06838 | 0.118603 | 40 | -0.57657 | 0.998984 |  |
| Control - (P10-Day17) | 0.505567 | 0.118603 | 40 | 4.262696 | 0.00276 | ** |
| Control - (P10-Day21) | 0.35437 | 0.118603 | 40 | 2.987877 | 0.081746 |  |
| Control - (P10-Day28) | 0.511397 | 0.118603 | 40 | 4.311853 | 0.002388 | ** |
| Control - (P10-Day7) | -0.36162 | 0.118603 | 40 | -3.04904 | 0.071006 |  |
| (P10-Day10) - (P10-Day12) | -0.12415 | 0.118603 | 40 | -1.04674 | 0.963876 |  |
| (P10-Day10) - (P10-Day14) | 0.057664 | 0.118603 | 40 | 0.486193 | 0.999667 |  |
| (P10-Day10) - (P10-Day17) | 0.631613 | 0.118603 | 40 | 5.325455 | 0.000106 | *** |
| (P10-Day10) - (P10-Day21) | 0.480416 | 0.118603 | 40 | 4.050636 | 0.005111 | ** |
| (P10-Day10) - (P10-Day28) | 0.637443 | 0.118603 | 40 | 5.374611 | 9.07E-05 | *** |
| (P10-Day10) - (P10-Day7) | -0.23558 | 0.118603 | 40 | -1.98628 | 0.503314 |  |
| (P10-Day12) - (P10-Day14) | 0.18181 | 0.118603 | 40 | 1.532933 | 0.785255 |  |
| (P10-Day12) - (P10-Day17) | 0.755759 | 0.118603 | 40 | 6.372195 | 3.75E-06 | *** |
| (P10-Day12) - (P10-Day21) | 0.604562 | 0.118603 | 40 | 5.097376 | 0.000217 | *** |
| (P10-Day12) - (P10-Day28) | 0.761589 | 0.118603 | 40 | 6.421352 | 3.2E-06 | *** |
| (P10-Day12) - (P10-Day7) | -0.11143 | 0.118603 | 40 | -0.93954 | 0.98004 |  |
| (P10-Day14) - (P10-Day17) | 0.573949 | 0.118603 | 40 | 4.839262 | 0.000484 | *** |
| (P10-Day14) - (P10-Day21) | 0.422752 | 0.118603 | 40 | 3.564443 | 0.019607 | * |
| (P10-Day14) - (P10-Day28) | 0.579779 | 0.118603 | 40 | 4.888419 | 0.000416 | *** |
| (P10-Day14) - (P10-Day7) | -0.29324 | 0.118603 | 40 | -2.47247 | 0.23651 |  |
| (P10-Day17) - (P10-Day21) | -0.1512 | 0.118603 | 40 | -1.27482 | 0.90274 |  |
| (P10-Day17) - (P10-Day28) | 0.00583 | 0.118603 | 40 | 0.049157 | 1 |  |
| (P10-Day17) - (P10-Day7) | -0.86719 | 0.118603 | 40 | -7.31174 | 1.87E-07 | *** |
| (P10-Day21) - (P10-Day28) | 0.157027 | 0.118603 | 40 | 1.323976 | 0.884236 |  |
| (P10-Day21) - (P10-Day7) | -0.71599 | 0.118603 | 40 | -6.03692 | 1.1E-05 | *** |
| (P10-Day28) - (P10-Day7) | -0.87302 | 0.118603 | 40 | -7.36089 | 1.6E-07 | *** |

| Sample | mean_Value | SE | df | lower.CL | upper.CL | Group |
| --- | --- | --- | --- | --- | --- | --- |
| P10-Day28 | 56.61479 | 0.083865 | 40 | 44.47358 | 72.07053 | a |
| P10-Day17 | 56.94582 | 0.083865 | 40 | 44.73362 | 72.49193 | a |
| P10-Day21 | 66.24084 | 0.083865 | 40 | 52.0353 | 84.32447 | ab |
| Control | 94.4119 | 0.083865 | 40 | 74.16499 | 120.1862 | bc |
| P10-Day14 | 101.0939 | 0.083865 | 40 | 79.41398 | 128.6923 | c |
| P10-Day10 | 107.0947 | 0.083865 | 40 | 84.12789 | 136.3313 | c |
| P10-Day12 | 121.2506 | 0.083865 | 40 | 95.24802 | 154.3517 | c |
| P10-Day7 | 135.5433 | 0.083865 | 40 | 106.4756 | 172.5464 | c |

**Caffeic acid**

| Info | Value |
| --- | --- |
| Best model family | gaussian |
| Model name | Normal_log |

| Effect | Df | Deviance | Resid. Df | Resid. Dev | Pr(>Chi) |
| --- | --- | --- | --- | --- | --- |
| NULL |  |  | 47 | 15.78404 |  |
| Sample | 7 | 15.089 | 40 | 0.695045 | 3.2E-183 |

| Comparison | Estimate | SE | df | t.ratio | p_value | Significance |
| --- | --- | --- | --- | --- | --- | --- |
| Control - (P10-Day10) | -1.73041 | 0.076105 | 40 | -22.737 | 4.64E-13 | *** |
| Control - (P10-Day12) | -1.86103 | 0.076105 | 40 | -24.4533 | 4.64E-13 | *** |
| Control - (P10-Day14) | -1.72522 | 0.076105 | 40 | -22.6688 | 4.64E-13 | *** |
| Control - (P10-Day17) | -1.43888 | 0.076105 | 40 | -18.9064 | 4.64E-13 | *** |
| Control - (P10-Day21) | -1.55968 | 0.076105 | 40 | -20.4937 | 4.64E-13 | *** |
| Control - (P10-Day28) | -1.45113 | 0.076105 | 40 | -19.0674 | 4.64E-13 | *** |
| Control - (P10-Day7) | -1.72965 | 0.076105 | 40 | -22.7271 | 4.64E-13 | *** |
| (P10-Day10) - (P10-Day12) | -0.13062 | 0.076105 | 40 | -1.71636 | 0.676946 |  |
| (P10-Day10) - (P10-Day14) | 0.005189 | 0.076105 | 40 | 0.068186 | 1 |  |
| (P10-Day10) - (P10-Day17) | 0.291527 | 0.076105 | 40 | 3.830571 | 0.009516 | ** |
| (P10-Day10) - (P10-Day21) | 0.170727 | 0.076105 | 40 | 2.243301 | 0.349128 |  |
| (P10-Day10) - (P10-Day28) | 0.279278 | 0.076105 | 40 | 3.669621 | 0.014795 | * |
| (P10-Day10) - (P10-Day7) | 0.000755 | 0.076105 | 40 | 0.009922 | 1 |  |
| (P10-Day12) - (P10-Day14) | 0.135814 | 0.076105 | 40 | 1.784543 | 0.633638 |  |
| (P10-Day12) - (P10-Day17) | 0.422152 | 0.076105 | 40 | 5.546928 | 5.25E-05 | *** |
| (P10-Day12) - (P10-Day21) | 0.301352 | 0.076105 | 40 | 3.959658 | 0.006625 | ** |
| (P10-Day12) - (P10-Day28) | 0.409902 | 0.076105 | 40 | 5.385979 | 8.75E-05 | *** |
| (P10-Day12) - (P10-Day7) | 0.131379 | 0.076105 | 40 | 1.726279 | 0.670714 |  |
| (P10-Day14) - (P10-Day17) | 0.286338 | 0.076105 | 40 | 3.762385 | 0.01149 | * |
| (P10-Day14) - (P10-Day21) | 0.165538 | 0.076105 | 40 | 2.175115 | 0.387642 |  |
| (P10-Day14) - (P10-Day28) | 0.274089 | 0.076105 | 40 | 3.601435 | 0.01777 | * |
| (P10-Day14) - (P10-Day7) | -0.00443 | 0.076105 | 40 | -0.05826 | 1 |  |
| (P10-Day17) - (P10-Day21) | -0.1208 | 0.076105 | 40 | -1.58727 | 0.754819 |  |
| (P10-Day17) - (P10-Day28) | -0.01225 | 0.076105 | 40 | -0.16095 | 1 |  |
| (P10-Day17) - (P10-Day7) | -0.29077 | 0.076105 | 40 | -3.82065 | 0.009782 | ** |
| (P10-Day21) - (P10-Day28) | 0.108551 | 0.076105 | 40 | 1.426321 | 0.839668 |  |
| (P10-Day21) - (P10-Day7) | -0.16997 | 0.076105 | 40 | -2.23338 | 0.354602 |  |
| (P10-Day28) - (P10-Day7) | -0.27852 | 0.076105 | 40 | -3.6597 | 0.015197 | * |

| Sample | mean_Value | SE | df | lower.CL | upper.CL | Group |
| --- | --- | --- | --- | --- | --- | --- |
| Control | 1.127932 | 0.053815 | 40 | 0.966088 | 1.31689 | a |
| P10-Day17 | 4.755339 | 0.053815 | 40 | 4.073006 | 5.55198 | b |
| P10-Day28 | 4.813946 | 0.053815 | 40 | 4.123203 | 5.620405 | b |
| P10-Day21 | 5.36592 | 0.053815 | 40 | 4.595976 | 6.264849 | bc |
| P10-Day14 | 6.331936 | 0.053815 | 40 | 5.423381 | 7.392697 | cd |
| P10-Day7 | 6.360075 | 0.053815 | 40 | 5.447483 | 7.425551 | cd |
| P10-Day10 | 6.36488 | 0.053815 | 40 | 5.451598 | 7.43116 | cd |
| P10-Day12 | 7.253032 | 0.053815 | 40 | 6.21231 | 8.4681 | d |

**Ferulic acid**

| Info | Value |
| --- | --- |
| Best model family | gaussian |
| Model name | Normal_log |

| Effect | Df | Deviance | Resid. Df | Resid. Dev | Pr(>Chi) |
| --- | --- | --- | --- | --- | --- |
| NULL |  |  | 47 | 46.16878 |  |
| Sample | 7 | 45.55881 | 40 | 0.609967 | 0 |

| Comparison | Estimate | SE | df | t.ratio | p_value | Significance |
| --- | --- | --- | --- | --- | --- | --- |
| Control - (P10-Day10) | -3.0301 | 0.071296 | 40 | -42.5006 | 4.64E-13 | *** |
| Control - (P10-Day12) | -3.15611 | 0.071296 | 40 | -44.2679 | 4.64E-13 | *** |
| Control - (P10-Day14) | -3.0279 | 0.071296 | 40 | -42.4696 | 4.64E-13 | *** |
| Control - (P10-Day17) | -2.85783 | 0.071296 | 40 | -40.0843 | 4.64E-13 | *** |
| Control - (P10-Day21) | -2.83043 | 0.071296 | 40 | -39.6999 | 4.64E-13 | *** |
| Control - (P10-Day28) | -2.78738 | 0.071296 | 40 | -39.0962 | 4.64E-13 | *** |
| Control - (P10-Day7) | -2.73632 | 0.071296 | 40 | -38.38 | 4.64E-13 | *** |
| (P10-Day10) - (P10-Day12) | -0.12601 | 0.071296 | 40 | -1.76737 | 0.644639 |  |
| (P10-Day10) - (P10-Day14) | 0.002207 | 0.071296 | 40 | 0.03095 | 1 |  |
| (P10-Day10) - (P10-Day17) | 0.172273 | 0.071296 | 40 | 2.416327 | 0.261541 |  |
| (P10-Day10) - (P10-Day21) | 0.199674 | 0.071296 | 40 | 2.800651 | 0.123535 |  |
| (P10-Day10) - (P10-Day28) | 0.242719 | 0.071296 | 40 | 3.404407 | 0.029754 | * |
| (P10-Day10) - (P10-Day7) | 0.293781 | 0.071296 | 40 | 4.120607 | 0.004178 | ** |
| (P10-Day12) - (P10-Day14) | 0.128212 | 0.071296 | 40 | 1.79832 | 0.624778 |  |
| (P10-Day12) - (P10-Day17) | 0.298279 | 0.071296 | 40 | 4.183696 | 0.003479 | ** |
| (P10-Day12) - (P10-Day21) | 0.32568 | 0.071296 | 40 | 4.56802 | 0.001109 | ** |
| (P10-Day12) - (P10-Day28) | 0.368725 | 0.071296 | 40 | 5.171777 | 0.000172 | *** |
| (P10-Day12) - (P10-Day7) | 0.419787 | 0.071296 | 40 | 5.887977 | 1.77E-05 | *** |
| (P10-Day14) - (P10-Day17) | 0.170067 | 0.071296 | 40 | 2.385377 | 0.276066 |  |
| (P10-Day14) - (P10-Day21) | 0.197467 | 0.071296 | 40 | 2.7697 | 0.131892 |  |
| (P10-Day14) - (P10-Day28) | 0.240513 | 0.071296 | 40 | 3.373457 | 0.032199 | * |
| (P10-Day14) - (P10-Day7) | 0.291574 | 0.071296 | 40 | 4.089657 | 0.004569 | ** |
| (P10-Day17) - (P10-Day21) | 0.027401 | 0.071296 | 40 | 0.384324 | 0.999931 |  |
| (P10-Day17) - (P10-Day28) | 0.070446 | 0.071296 | 40 | 0.98808 | 0.973578 |  |
| (P10-Day17) - (P10-Day7) | 0.121508 | 0.071296 | 40 | 1.70428 | 0.684495 |  |
| (P10-Day21) - (P10-Day28) | 0.043045 | 0.071296 | 40 | 0.603757 | 0.998634 |  |
| (P10-Day21) - (P10-Day7) | 0.094107 | 0.071296 | 40 | 1.319956 | 0.885821 |  |
| (P10-Day28) - (P10-Day7) | 0.051062 | 0.071296 | 40 | 0.7162 | 0.996 |  |

| Sample | mean_Value | SE | df | lower.CL | upper.CL | Group |
| --- | --- | --- | --- | --- | --- | --- |
| Control | 3.084305 | 0.050414 | 40 | 2.667732 | 3.565927 | a |
| P10-Day7 | 47.59124 | 0.050414 | 40 | 41.16346 | 55.02273 | b |
| P10-Day28 | 50.08445 | 0.050414 | 40 | 43.31993 | 57.90526 | b |
| P10-Day21 | 52.28742 | 0.050414 | 40 | 45.22536 | 60.45223 | bc |
| P10-Day17 | 53.73993 | 0.050414 | 40 | 46.4817 | 62.13156 | bc |
| P10-Day14 | 63.70246 | 0.050414 | 40 | 55.09866 | 73.64976 | cd |
| P10-Day10 | 63.84318 | 0.050414 | 40 | 55.22038 | 73.81245 | cd |
| P10-Day12 | 72.41659 | 0.050414 | 40 | 62.63584 | 83.72462 | d |

**Syringic acid**

| Info | Value |
| --- | --- |
| Best model family | gaussian |
| Model name | Normal_log |

| Effect | Df | Deviance | Resid. Df | Resid. Dev | Pr(>Chi) |
| --- | --- | --- | --- | --- | --- |
| NULL |  |  | 47 | 6.822892 |  |
| Sample | 7 | 5.611269 | 40 | 1.211623 | 1.52E-36 |

| Comparison | Estimate | SE | df | t.ratio | p_value | Significance |
| --- | --- | --- | --- | --- | --- | --- |
| Control - (P10-Day10) | -0.89257 | 0.100483 | 40 | -8.88276 | 1.44E-09 | *** |
| Control - (P10-Day12) | -1.15251 | 0.100483 | 40 | -11.4697 | 1.38E-12 | *** |
| Control - (P10-Day14) | -1.0224 | 0.100483 | 40 | -10.1748 | 3.27E-11 | *** |
| Control - (P10-Day17) | -0.77196 | 0.100483 | 40 | -7.68251 | 5.8E-08 | *** |
| Control - (P10-Day21) | -1.02778 | 0.100483 | 40 | -10.2284 | 2.82E-11 | *** |
| Control - (P10-Day28) | -1.01954 | 0.100483 | 40 | -10.1464 | 3.54E-11 | *** |
| Control - (P10-Day7) | -1.00588 | 0.100483 | 40 | -10.0105 | 5.21E-11 | *** |
| (P10-Day10) - (P10-Day12) | -0.25994 | 0.100483 | 40 | -2.58693 | 0.190766 |  |
| (P10-Day10) - (P10-Day14) | -0.12983 | 0.100483 | 40 | -1.29206 | 0.896467 |  |
| (P10-Day10) - (P10-Day17) | 0.120605 | 0.100483 | 40 | 1.200249 | 0.927129 |  |
| (P10-Day10) - (P10-Day21) | -0.13521 | 0.100483 | 40 | -1.34562 | 0.875483 |  |
| (P10-Day10) - (P10-Day28) | -0.12697 | 0.100483 | 40 | -1.26362 | 0.906685 |  |
| (P10-Day10) - (P10-Day7) | -0.11331 | 0.100483 | 40 | -1.12769 | 0.946673 |  |
| (P10-Day12) - (P10-Day14) | 0.130112 | 0.100483 | 40 | 1.294861 | 0.895428 |  |
| (P10-Day12) - (P10-Day17) | 0.380547 | 0.100483 | 40 | 3.787175 | 0.010731 | * |
| (P10-Day12) - (P10-Day21) | 0.12473 | 0.100483 | 40 | 1.241307 | 0.914249 |  |
| (P10-Day12) - (P10-Day28) | 0.132969 | 0.100483 | 40 | 1.323301 | 0.884503 |  |
| (P10-Day12) - (P10-Day7) | 0.146629 | 0.100483 | 40 | 1.459237 | 0.823685 |  |
| (P10-Day14) - (P10-Day17) | 0.250436 | 0.100483 | 40 | 2.492314 | 0.228073 |  |
| (P10-Day14) - (P10-Day21) | -0.00538 | 0.100483 | 40 | -0.05355 | 1 |  |
| (P10-Day14) - (P10-Day28) | 0.002858 | 0.100483 | 40 | 0.028441 | 1 |  |
| (P10-Day14) - (P10-Day7) | 0.016517 | 0.100483 | 40 | 0.164376 | 1 |  |
| (P10-Day17) - (P10-Day21) | -0.25582 | 0.100483 | 40 | -2.54587 | 0.206365 |  |
| (P10-Day17) - (P10-Day28) | -0.24758 | 0.100483 | 40 | -2.46387 | 0.240233 |  |
| (P10-Day17) - (P10-Day7) | -0.23392 | 0.100483 | 40 | -2.32794 | 0.304366 |  |
| (P10-Day21) - (P10-Day28) | 0.008239 | 0.100483 | 40 | 0.081994 | 1 |  |
| (P10-Day21) - (P10-Day7) | 0.021898 | 0.100483 | 40 | 0.217929 | 0.999999 |  |
| (P10-Day28) - (P10-Day7) | 0.013659 | 0.100483 | 40 | 0.135935 | 1 |  |

| Sample | mean_Value | SE | df | lower.CL | upper.CL | Group |
| --- | --- | --- | --- | --- | --- | --- |
| Control | 37.31405 | 0.071052 | 40 | 30.41302 | 45.78099 | a |
| P10-Day17 | 80.74798 | 0.071052 | 40 | 65.81409 | 99.07053 | b |
| P10-Day10 | 91.09818 | 0.071052 | 40 | 74.25008 | 111.7693 | bc |
| P10-Day7 | 102.0284 | 0.071052 | 40 | 83.15884 | 125.1797 | bc |
| P10-Day28 | 103.4316 | 0.071052 | 40 | 84.30252 | 126.9013 | bc |
| P10-Day14 | 103.7276 | 0.071052 | 40 | 84.54378 | 127.2645 | bc |
| P10-Day21 | 104.2873 | 0.071052 | 40 | 84.99996 | 127.9512 | bc |
| P10-Day12 | 118.1412 | 0.071052 | 40 | 96.29161 | 144.9486 | c |

**Catechin**

| Info | Value |
| --- | --- |
| Best model family | gaussian |
| Model name | Normal_log |

| Effect | Df | Deviance | Resid. Df | Resid. Dev | Pr(>Chi) |
| --- | --- | --- | --- | --- | --- |
| NULL |  |  | 47 | 2.779762 |  |
| Sample | 7 | 2.531701 | 40 | 0.248061 | 4.08E-84 |

| Comparison | Estimate | SE | df | t.ratio | p_value | Significance |
| --- | --- | --- | --- | --- | --- | --- |
| Control - (P10-Day10) | -0.26245 | 0.045466 | 40 | -5.77237 | 2.56E-05 | *** |
| Control - (P10-Day12) | -0.29376 | 0.045466 | 40 | -6.46097 | 2.82E-06 | *** |
| Control - (P10-Day14) | -0.17353 | 0.045466 | 40 | -3.81678 | 0.009888 | ** |
| Control - (P10-Day17) | 0.162619 | 0.045466 | 40 | 3.576699 | 0.01898 | * |
| Control - (P10-Day21) | 0.14604 | 0.045466 | 40 | 3.212055 | 0.048142 | * |
| Control - (P10-Day28) | 0.21777 | 0.045466 | 40 | 4.789715 | 0.000564 | *** |
| Control - (P10-Day7) | -0.43944 | 0.045466 | 40 | -9.66526 | 1.41E-10 | *** |
| (P10-Day10) - (P10-Day12) | -0.03131 | 0.045466 | 40 | -0.6886 | 0.996866 |  |
| (P10-Day10) - (P10-Day14) | 0.088913 | 0.045466 | 40 | 1.95559 | 0.522982 |  |
| (P10-Day10) - (P10-Day17) | 0.425067 | 0.045466 | 40 | 9.34907 | 3.57E-10 | *** |
| (P10-Day10) - (P10-Day21) | 0.408488 | 0.045466 | 40 | 8.984426 | 1.06E-09 | *** |
| (P10-Day10) - (P10-Day28) | 0.480218 | 0.045466 | 40 | 10.56209 | 1.13E-11 | *** |
| (P10-Day10) - (P10-Day7) | -0.17699 | 0.045466 | 40 | -3.89288 | 0.007997 | ** |
| (P10-Day12) - (P10-Day14) | 0.120221 | 0.045466 | 40 | 2.644186 | 0.170501 |  |
| (P10-Day12) - (P10-Day17) | 0.456374 | 0.045466 | 40 | 10.03767 | 4.82E-11 | *** |
| (P10-Day12) - (P10-Day21) | 0.439795 | 0.045466 | 40 | 9.673022 | 1.38E-10 | *** |
| (P10-Day12) - (P10-Day28) | 0.511526 | 0.045466 | 40 | 11.25068 | 2.1E-12 | *** |
| (P10-Day12) - (P10-Day7) | -0.14569 | 0.045466 | 40 | -3.20429 | 0.049062 | * |
| (P10-Day14) - (P10-Day17) | 0.336153 | 0.045466 | 40 | 7.39348 | 1.44E-07 | *** |
| (P10-Day14) - (P10-Day21) | 0.319574 | 0.045466 | 40 | 7.028836 | 4.59E-07 | *** |
| (P10-Day14) - (P10-Day28) | 0.391305 | 0.045466 | 40 | 8.606496 | 3.32E-09 | *** |
| (P10-Day14) - (P10-Day7) | -0.26591 | 0.045466 | 40 | -5.84848 | 2.01E-05 | *** |
| (P10-Day17) - (P10-Day21) | -0.01658 | 0.045466 | 40 | -0.36464 | 0.999951 |  |
| (P10-Day17) - (P10-Day28) | 0.055151 | 0.045466 | 40 | 1.213016 | 0.923267 |  |
| (P10-Day17) - (P10-Day7) | -0.60206 | 0.045466 | 40 | -13.242 | 4.79E-13 | *** |
| (P10-Day21) - (P10-Day28) | 0.07173 | 0.045466 | 40 | 1.57766 | 0.76032 |  |
| (P10-Day21) - (P10-Day7) | -0.58548 | 0.045466 | 40 | -12.8773 | 4.96E-13 | *** |
| (P10-Day28) - (P10-Day7) | -0.65721 | 0.045466 | 40 | -14.455 | 4.65E-13 | *** |

| Sample | mean_Value | SE | df | lower.CL | upper.CL | Group |
| --- | --- | --- | --- | --- | --- | --- |
| P10-Day28 | 65.37975 | 0.032149 | 40 | 59.60156 | 71.71812 | a |
| P10-Day17 | 69.08681 | 0.032149 | 40 | 62.98099 | 75.78456 | a |
| P10-Day21 | 70.24174 | 0.032149 | 40 | 64.03385 | 77.05147 | a |
| Control | 81.28672 | 0.032149 | 40 | 74.10269 | 89.16722 | b |
| P10-Day14 | 96.69069 | 0.032149 | 40 | 88.14527 | 106.0646 | c |
| P10-Day10 | 105.6816 | 0.032149 | 40 | 96.34153 | 115.9271 | c |
| P10-Day12 | 109.0426 | 0.032149 | 40 | 99.40549 | 119.6139 | c |
| P10-Day7 | 126.1441 | 0.032149 | 40 | 114.9956 | 138.3734 | d |

Degradation of phenolic acids and other host compounds in medium from fungi

**Fig. 2 c** and **Suppl. Fig. 14**

**Gallic acid_Biomass**

| **Modell** |
| --- |
| Normal_log |

| **Resid. Df** | **Resid. Dev** | **Df** | **Deviance** | **Pr(>Chi)** |
| --- | --- | --- | --- | --- |
| 35 | 260.1651 |  |  |  |
| 30 | 4.310245 | 5 | 255.8549 | 0 |

| **contrast** | **estimate** | **SE** | **df** | **t.ratio** | **p.value** | **Signif** |
| --- | --- | --- | --- | --- | --- | --- |
| Control_Medium - P10 | 0.534826 | 0.218842 | 30 | 2.443897 | 0.025781 | * |
| Control_Medium - P148 | 4.870367 | 0.218842 | 30 | 22.25522 | 7.14E-20 | *** |
| Control_Medium - P159 | 5.932386 | 0.218842 | 30 | 27.10814 | 4.92E-22 | *** |
| Control_Medium - P331 | 6.444904 | 0.218842 | 30 | 29.45009 | 1.6E-22 | *** |
| Control_Medium - P7 | 6.042294 | 0.218842 | 30 | 27.61036 | 4.92E-22 | *** |
| P10 - P148 | 4.33554 | 0.218842 | 30 | 19.81132 | 1.65E-18 | *** |
| P10 - P159 | 5.39756 | 0.218842 | 30 | 24.66424 | 4.45E-21 | *** |
| P10 - P331 | 5.910077 | 0.218842 | 30 | 27.0062 | 4.92E-22 | *** |
| P10 - P7 | 5.507468 | 0.218842 | 30 | 25.16646 | 3E-21 | *** |
| P148 - P159 | 1.062019 | 0.218842 | 30 | 4.852914 | 4.81E-05 | *** |
| P148 - P331 | 1.574537 | 0.218842 | 30 | 7.194873 | 8.74E-08 | *** |
| P148 - P7 | 1.171927 | 0.218842 | 30 | 5.355141 | 1.28E-05 | *** |
| P159 - P331 | 0.512518 | 0.218842 | 30 | 2.341958 | 0.030016 | * |
| P159 - P7 | 0.109908 | 0.218842 | 30 | 0.502226 | 0.619175 |  |
| P331 - P7 | -0.40261 | 0.218842 | 30 | -1.83973 | 0.081131 |  |

| Sample | mean | SE | lower.CL | upper.CL | group |
| --- | --- | --- | --- | --- | --- |
| P331 | 3.707721 | 0.154744 | 2.398044 | 5.732671 | a |
| P7 | 5.545724 | 0.154744 | 3.58681 | 8.574489 | ab |
| P159 | 6.190001 | 0.154744 | 4.003509 | 9.570633 | b |
| P148 | 17.90275 | 0.154744 | 11.57897 | 27.68023 | c |
| P10 | 1367.165 | 0.154744 | 884.242 | 2113.835 | d |
| Control_Medium | 2333.959 | 0.154744 | 1509.535 | 3608.636 | e |

**Gallic acid_Medium**

| Info | Value |
| --- | --- |
| Best model family | gaussian |
| Model name | Normal_log |

| Effect | Df | Deviance | Resid. Df | Resid. Dev | Pr(>Chi) |
| --- | --- | --- | --- | --- | --- |
| NULL |  |  | 35 | 247.2966 |  |
| Sample | 5 | 237.8731 | 30 | 9.423416 | 2E-161 |

| Comparison | Estimate | SE | df | t.ratio | p_value | Significance |
| --- | --- | --- | --- | --- | --- | --- |
| Control_Medium - P10 | -0.07392 | 0.323581 | 30 | -0.22846 | 0.999905 |  |
| Control_Medium - P148 | 1.026165 | 0.323581 | 30 | 3.171278 | 0.037094 | * |
| Control_Medium - P159 | 6.083341 | 0.323581 | 30 | 18.80006 | 4.61E-14 | *** |
| Control_Medium - P331 | 4.852275 | 0.323581 | 30 | 14.99555 | 7.32E-14 | *** |
| Control_Medium - P7 | 5.127043 | 0.323581 | 30 | 15.8447 | 5.23E-14 | *** |
| P10 - P148 | 1.100089 | 0.323581 | 30 | 3.399735 | 0.021499 | * |
| P10 - P159 | 6.157265 | 0.323581 | 30 | 19.02852 | 4.61E-14 | *** |
| P10 - P331 | 4.926199 | 0.323581 | 30 | 15.22401 | 6.38E-14 | *** |
| P10 - P7 | 5.200968 | 0.323581 | 30 | 16.07316 | 5.05E-14 | *** |
| P148 - P159 | 5.057176 | 0.323581 | 30 | 15.62878 | 5.5E-14 | *** |
| P148 - P331 | 3.826109 | 0.323581 | 30 | 11.82427 | 1.19E-11 | *** |
| P148 - P7 | 4.100878 | 0.323581 | 30 | 12.67342 | 2.13E-12 | *** |
| P159 - P331 | -1.23107 | 0.323581 | 30 | -3.80451 | 0.00778 | ** |
| P159 - P7 | -0.9563 | 0.323581 | 30 | -2.95536 | 0.06068 |  |
| P331 - P7 | 0.274769 | 0.323581 | 30 | 0.84915 | 0.955457 |  |

| Sample | mean_Value | SE | df | lower.CL | upper.CL | Group |
| --- | --- | --- | --- | --- | --- | --- |
| P159 | 5.322697 | 0.228806 | 30 | 2.794507 | 10.13814 | a |
| P7 | 13.8499 | 0.228806 | 30 | 7.271432 | 26.3799 | ab |
| P331 | 18.2296 | 0.228806 | 30 | 9.57085 | 34.72191 | b |
| P148 | 836.4409 | 0.228806 | 30 | 439.1458 | 1593.169 | c |
| Control_Medium | 2333.959 | 0.228806 | 30 | 1225.368 | 4445.491 | d |
| P10 | 2513.032 | 0.228806 | 30 | 1319.385 | 4786.573 | d |

**Syringic acid_Biomass**

| Info | Value |
| --- | --- |
| Best model family | gaussian |
| Model name | Normal_log |

| **Resid. Df** | **Resid. Dev** | **Df** | **Deviance** | **Pr(>Chi)** |
| --- | --- | --- | --- | --- |
| 35 | 260.5936 |  |  |  |
| 30 | 15.76418 | 5 | 244.8295 | 1.8E-98 |

| **contrast** | **estimate** | **SE** | **df** | **t.ratio** | **p.value** | **Signif** |
| --- | --- | --- | --- | --- | --- | --- |
| Control_Medium - P10 | 1.032709 | 0.418518 | 30 | 2.467536 | 0.024414 | * |
| Control_Medium - P148 | 6.005634 | 0.418518 | 30 | 14.34975 | 2.83E-14 | *** |
| Control_Medium - P159 | 6.434379 | 0.418518 | 30 | 15.37419 | 8.97E-15 | *** |
| Control_Medium - P331 | 4.699541 | 0.418518 | 30 | 11.229 | 6.17E-12 | *** |
| Control_Medium - P7 | 6.3687 | 0.418518 | 30 | 15.21726 | 8.97E-15 | *** |
| P10 - P148 | 4.972925 | 0.418518 | 30 | 11.88222 | 1.78E-12 | *** |
| P10 - P159 | 5.40167 | 0.418518 | 30 | 12.90665 | 3.32E-13 | *** |
| P10 - P331 | 3.666832 | 0.418518 | 30 | 8.761462 | 1.7E-09 | *** |
| P10 - P7 | 5.335991 | 0.418518 | 30 | 12.74972 | 3.63E-13 | *** |
| P148 - P159 | 0.428745 | 0.418518 | 30 | 1.024435 | 0.3621 |  |
| P148 - P331 | -1.30609 | 0.418518 | 30 | -3.12076 | 0.005412 | ** |
| P148 - P7 | 0.363066 | 0.418518 | 30 | 0.867504 | 0.420592 |  |
| P159 - P331 | -1.73484 | 0.418518 | 30 | -4.14519 | 0.000426 | *** |
| P159 - P7 | -0.06568 | 0.418518 | 30 | -0.15693 | 0.87635 |  |
| P331 - P7 | 1.66916 | 0.418518 | 30 | 3.988261 | 0.000592 | *** |

| Sample | mean | SE | lower.CL | upper.CL | group |
| --- | --- | --- | --- | --- | --- |
| P159 | 5.441088 | 0.295937 | 2.364608 | 12.52023 | a |
| P7 | 5.810448 | 0.295937 | 2.525126 | 13.37015 | a |
| P148 | 8.353861 | 0.295937 | 3.630452 | 19.22267 | a |
| P331 | 30.84015 | 0.295937 | 13.40263 | 70.96482 | b |
| P10 | 1206.705 | 0.295937 | 524.4145 | 2776.693 | c |
| Control_Medium | 3389.23 | 0.295937 | 1472.904 | 7798.798 | d |

**Syringic acid_medium**

| Info | Value |
| --- | --- |
| Best model family | gaussian |
| Model name | Normal_log |

| Effect | Df | Deviance | Resid. Df | Resid. Dev | Pr(>Chi) |
| --- | --- | --- | --- | --- | --- |
| NULL |  |  | 35 | 218.7406 |  |
| Sample | 5 | 204.4339 | 30 | 14.30666 | 1.94E-90 |

| Comparison | Estimate | SE | df | t.ratio | p_value | Significance |
| --- | --- | --- | --- | --- | --- | --- |
| Control_Medium - P10 | -0.06368 | 0.398702 | 30 | -0.15972 | 0.999984 |  |
| Control_Medium - P148 | 0.556078 | 0.398702 | 30 | 1.394723 | 0.729748 |  |
| Control_Medium - P159 | 4.065801 | 0.398702 | 30 | 10.19761 | 4.22E-10 | *** |
| Control_Medium - P331 | 2.86995 | 0.398702 | 30 | 7.198241 | 7.4E-07 | *** |
| Control_Medium - P7 | 6.387935 | 0.398702 | 30 | 16.02185 | 5.07E-14 | *** |
| P10 - P148 | 0.619761 | 0.398702 | 30 | 1.554448 | 0.633269 |  |
| P10 - P159 | 4.129484 | 0.398702 | 30 | 10.35733 | 2.93E-10 | *** |
| P10 - P331 | 2.933632 | 0.398702 | 30 | 7.357966 | 4.84E-07 | *** |
| P10 - P7 | 6.451618 | 0.398702 | 30 | 16.18157 | 4.98E-14 | *** |
| P148 - P159 | 3.509723 | 0.398702 | 30 | 8.802883 | 1.18E-08 | *** |
| P148 - P331 | 2.313871 | 0.398702 | 30 | 5.803517 | 3.33E-05 | *** |
| P148 - P7 | 5.831857 | 0.398702 | 30 | 14.62712 | 9.87E-14 | *** |
| P159 - P331 | -1.19585 | 0.398702 | 30 | -2.99937 | 0.055 |  |
| P159 - P7 | 2.322134 | 0.398702 | 30 | 5.824241 | 3.15E-05 | *** |
| P331 - P7 | 3.517985 | 0.398702 | 30 | 8.823607 | 1.12E-08 | *** |

| Sample | mean_Value | SE | df | lower.CL | upper.CL | Group |
| --- | --- | --- | --- | --- | --- | --- |
| P7 | 5.699755 | 0.281925 | 30 | 2.576717 | 12.60798 | a |
| P159 | 58.12273 | 0.281925 | 30 | 26.27584 | 128.5688 | b |
| P331 | 192.1754 | 0.281925 | 30 | 86.87772 | 425.0962 | b |
| P148 | 1943.566 | 0.281925 | 30 | 878.6379 | 4299.211 | c |
| Control_Medium | 3389.23 | 0.281925 | 30 | 1532.187 | 7497.052 | c |
| P10 | 3612.086 | 0.281925 | 30 | 1632.934 | 7990.013 | c |

**Caffeic acid_Biomass**

| Info | Value |
| --- | --- |
| Best model family | gaussian |
| Model name | Sqrt_Normal |

| Effect | Df | Deviance | Resid. Df | Resid. Dev | Pr(>Chi) |
| --- | --- | --- | --- | --- | --- |
| NULL |  |  | 35 | 1697.516 |  |
| Sample | 5 | 1685.54 | 30 | 11.97572 | 0 |

| Comparison | Estimate | SE | df | t.ratio | p_value | Significance |
| --- | --- | --- | --- | --- | --- | --- |
| Control_Medium - P10 | 16.70207 | 0.364779 | 30 | 45.78685 | 4.61E-14 | *** |
| Control_Medium - P148 | 18.56194 | 0.364779 | 30 | 50.88547 | 4.61E-14 | *** |
| Control_Medium - P159 | 19.09921 | 0.364779 | 30 | 52.35833 | 4.61E-14 | *** |
| Control_Medium - P331 | 18.62181 | 0.364779 | 30 | 51.0496 | 4.61E-14 | *** |
| Control_Medium - P7 | 18.26796 | 0.364779 | 30 | 50.07957 | 4.61E-14 | *** |
| P10 - P148 | 1.859868 | 0.364779 | 30 | 5.098619 | 0.000236 | *** |
| P10 - P159 | 2.397134 | 0.364779 | 30 | 6.571474 | 4.02E-06 | *** |
| P10 - P331 | 1.91974 | 0.364779 | 30 | 5.262751 | 0.00015 | *** |
| P10 - P7 | 1.565893 | 0.364779 | 30 | 4.292719 | 0.002146 | ** |
| P148 - P159 | 0.537266 | 0.364779 | 30 | 1.472856 | 0.683385 |  |
| P148 - P331 | 0.059872 | 0.364779 | 30 | 0.164132 | 0.999982 |  |
| P148 - P7 | -0.29398 | 0.364779 | 30 | -0.8059 | 0.964206 |  |
| P159 - P331 | -0.47739 | 0.364779 | 30 | -1.30872 | 0.777989 |  |
| P159 - P7 | -0.83124 | 0.364779 | 30 | -2.27875 | 0.233935 |  |
| P331 - P7 | -0.35385 | 0.364779 | 30 | -0.97003 | 0.923834 |  |

| Sample | mean_Value | SE | df | lower.CL | upper.CL | Group |
| --- | --- | --- | --- | --- | --- | --- |
| P159 | 0.12724 | 0.257938 | 30 | -0.59912 | 0.853599 | a |
| P331 | 0.604635 | 0.257938 | 30 | -0.12172 | 1.330994 | a |
| P148 | 0.664507 | 0.257938 | 30 | -0.06185 | 1.390866 | a |
| P7 | 0.958482 | 0.257938 | 30 | 0.232123 | 1.684841 | a |
| P10 | 2.524374 | 0.257938 | 30 | 1.798016 | 3.250733 | b |
| Control_Medium | 19.22645 | 0.257938 | 30 | 18.50009 | 19.9528 | c |

**Caffeic acid_Medium**

| Info | Value |
| --- | --- |
| Best model family | gaussian |
| Model name | Sqrt_Normal |

| Effect | Df | Deviance | Resid. Df | Resid. Dev | Pr(>Chi) |
| --- | --- | --- | --- | --- | --- |
| NULL |  |  | 35 | 2336.275 |  |
| Sample | 5 | 2285.629 | 30 | 50.64584 | 1.3E-290 |

| Comparison | Estimate | SE | df | t.ratio | p_value | Significance |
| --- | --- | --- | --- | --- | --- | --- |
| Control_Medium - P10 | 1.303402 | 0.750154 | 30 | 1.737512 | 0.519078 |  |
| Control_Medium - P148 | 14.41345 | 0.750154 | 30 | 19.21397 | 4.61E-14 | *** |
| Control_Medium - P159 | 18.17702 | 0.750154 | 30 | 24.23103 | 4.61E-14 | *** |
| Control_Medium - P331 | 18.25373 | 0.750154 | 30 | 24.3333 | 4.61E-14 | *** |
| Control_Medium - P7 | 18.31091 | 0.750154 | 30 | 24.40952 | 4.61E-14 | *** |
| P10 - P148 | 13.11004 | 0.750154 | 30 | 17.47646 | 4.65E-14 | *** |
| P10 - P159 | 16.87361 | 0.750154 | 30 | 22.49352 | 4.61E-14 | *** |
| P10 - P331 | 16.95033 | 0.750154 | 30 | 22.59579 | 4.61E-14 | *** |
| P10 - P7 | 17.0075 | 0.750154 | 30 | 22.67201 | 4.61E-14 | *** |
| P148 - P159 | 3.76357 | 0.750154 | 30 | 5.017061 | 0.000296 | *** |
| P148 - P331 | 3.840288 | 0.750154 | 30 | 5.11933 | 0.000223 | *** |
| P148 - P7 | 3.89746 | 0.750154 | 30 | 5.195544 | 0.00018 | *** |
| P159 - P331 | 0.076718 | 0.750154 | 30 | 0.102269 | 0.999998 |  |
| P159 - P7 | 0.13389 | 0.750154 | 30 | 0.178483 | 0.999972 |  |
| P331 - P7 | 0.057172 | 0.750154 | 30 | 0.076214 | 1 |  |

| Sample | mean_Value | SE | df | lower.CL | upper.CL | Group |
| --- | --- | --- | --- | --- | --- | --- |
| P7 | 0.91554 | 0.530439 | 30 | -0.57819 | 2.409271 | a |
| P331 | 0.972712 | 0.530439 | 30 | -0.52102 | 2.466443 | a |
| P159 | 1.04943 | 0.530439 | 30 | -0.4443 | 2.543161 | a |
| P148 | 4.813 | 0.530439 | 30 | 3.319269 | 6.306731 | b |
| P10 | 17.92304 | 0.530439 | 30 | 16.42931 | 19.41677 | c |
| Control_Medium | 19.22645 | 0.530439 | 30 | 17.73271 | 20.72018 | c |

**Ferulic acid_Biomass**

| Info | Value |
| --- | --- |
| Best model family | gaussian |
| Model name | Normal_log |

| Effect | Df | Deviance | Resid. Df | Resid. Dev | Pr(>Chi) |
| --- | --- | --- | --- | --- | --- |
| NULL |  |  | 35 | 366.7849 |  |
| Sample | 5 | 361.1056 | 30 | 5.679289 | 0 |

| Comparison | Estimate | SE | df | t.ratio | p_value | Significance |
| --- | --- | --- | --- | --- | --- | --- |
| Control_Medium - P10 | 8.292627 | 0.251204 | 30 | 33.01159 | 4.61E-14 | *** |
| Control_Medium - P148 | 4.743045 | 0.251204 | 30 | 18.88129 | 4.61E-14 | *** |
| Control_Medium - P159 | 7.069298 | 0.251204 | 30 | 28.14172 | 4.61E-14 | *** |
| Control_Medium - P331 | 9.921485 | 0.251204 | 30 | 39.49581 | 4.61E-14 | *** |
| Control_Medium - P7 | 7.001423 | 0.251204 | 30 | 27.87152 | 4.61E-14 | *** |
| P10 - P148 | -3.54958 | 0.251204 | 30 | -14.1303 | 1.75E-13 | *** |
| P10 - P159 | -1.22333 | 0.251204 | 30 | -4.86987 | 0.000444 | *** |
| P10 - P331 | 1.628859 | 0.251204 | 30 | 6.484219 | 5.1E-06 | *** |
| P10 - P7 | -1.2912 | 0.251204 | 30 | -5.14007 | 0.00021 | *** |
| P148 - P159 | 2.326252 | 0.251204 | 30 | 9.26043 | 3.85E-09 | *** |
| P148 - P331 | 5.17844 | 0.251204 | 30 | 20.61452 | 4.61E-14 | *** |
| P148 - P7 | 2.258378 | 0.251204 | 30 | 8.990231 | 7.44E-09 | *** |
| P159 - P331 | 2.852187 | 0.251204 | 30 | 11.35409 | 3.22E-11 | *** |
| P159 - P7 | -0.06787 | 0.251204 | 30 | -0.2702 | 0.999783 |  |
| P331 - P7 | -2.92006 | 0.251204 | 30 | -11.6243 | 1.81E-11 | *** |

| Sample | mean_Value | SE | df | lower.CL | upper.CL | Group |
| --- | --- | --- | --- | --- | --- | --- |
| P331 | 0.112246 | 0.177628 | 30 | 0.068067 | 0.185101 | a |
| P10 | 0.572237 | 0.177628 | 30 | 0.347008 | 0.943652 | b |
| P159 | 1.944737 | 0.177628 | 30 | 1.179302 | 3.206984 | c |
| P7 | 2.081318 | 0.177628 | 30 | 1.262126 | 3.432215 | c |
| P148 | 19.91313 | 0.177628 | 30 | 12.07545 | 32.8379 | d |
| Control_Medium | 2285.693 | 0.177628 | 30 | 1386.06 | 3769.241 | e |

**Ferulic acid_Medium**

| Info | Value |
| --- | --- |
| Best model family | gaussian |
| Model name | Normal_log |

| Effect | Df | Deviance | Resid. Df | Resid. Dev | Pr(>Chi) |
| --- | --- | --- | --- | --- | --- |
| NULL |  |  | 35 | 403.4856 |  |
| Sample | 5 | 382.9583 | 30 | 20.52727 | 1E-118 |

| Comparison | Estimate | SE | df | t.ratio | p_value | Significance |
| --- | --- | --- | --- | --- | --- | --- |
| Control_Medium - P10 | 3.257878 | 0.477578 | 30 | 6.821667 | 2.04E-06 | *** |
| Control_Medium - P148 | 0.841351 | 0.477578 | 30 | 1.761704 | 0.504183 |  |
| Control_Medium - P159 | 7.585802 | 0.477578 | 30 | 15.8839 | 5.21E-14 | *** |
| Control_Medium - P331 | 8.129212 | 0.477578 | 30 | 17.02175 | 4.7E-14 | *** |
| Control_Medium - P7 | 6.942199 | 0.477578 | 30 | 14.53626 | 1.08E-13 | *** |
| P10 - P148 | -2.41653 | 0.477578 | 30 | -5.05996 | 0.000263 | *** |
| P10 - P159 | 4.327924 | 0.477578 | 30 | 9.062234 | 6.24E-09 | *** |
| P10 - P331 | 4.871334 | 0.477578 | 30 | 10.20008 | 4.2E-10 | *** |
| P10 - P7 | 3.684321 | 0.477578 | 30 | 7.714594 | 1.89E-07 | *** |
| P148 - P159 | 6.744451 | 0.477578 | 30 | 14.1222 | 1.76E-13 | *** |
| P148 - P331 | 7.287861 | 0.477578 | 30 | 15.26004 | 6.29E-14 | *** |
| P148 - P7 | 6.100848 | 0.477578 | 30 | 12.77456 | 1.75E-12 | *** |
| P159 - P331 | 0.54341 | 0.477578 | 30 | 1.137846 | 0.861616 |  |
| P159 - P7 | -0.6436 | 0.477578 | 30 | -1.34764 | 0.756582 |  |
| P331 - P7 | -1.18701 | 0.477578 | 30 | -2.48548 | 0.160556 |  |

| Sample | mean_Value | SE | df | lower.CL | upper.CL | Group |
| --- | --- | --- | --- | --- | --- | --- |
| P331 | 0.673823 | 0.337699 | 30 | 0.260343 | 1.743999 | a |
| P159 | 1.160235 | 0.337699 | 30 | 0.448276 | 3.002937 | a |
| P7 | 2.208306 | 0.337699 | 30 | 0.853216 | 5.715569 | a |
| P10 | 87.93046 | 0.337699 | 30 | 33.9734 | 227.5829 | b |
| P148 | 985.4254 | 0.337699 | 30 | 380.7356 | 2550.492 | c |
| Control_Medium | 2285.693 | 0.337699 | 30 | 883.1157 | 5915.864 | c |

**Protocatechuic acid_Biomass**

| **Modell** |
| --- |
| Sqrt_Normal |

| **Resid. Df** | **Resid. Dev** | **Df** | **Deviance** | **Pr(>Chi)** |
| --- | --- | --- | --- | --- |
| 35 | 5760.253 |  |  |  |
| 30 | 216.6689 | 5 | 5543.584 | 1.2E-163 |

| **contrast** | **estimate** | **SE** | **df** | **t.ratio** | **p.value** | **Signif** |
| --- | --- | --- | --- | --- | --- | --- |
| Control_Medium - P10 | 15.09703 | 1.55159 | 30 | 9.730036 | 1.25E-09 | *** |
| Control_Medium - P148 | 29.42518 | 1.55159 | 30 | 18.96454 | 4.61E-14 | *** |
| Control_Medium - P159 | 32.84358 | 1.55159 | 30 | 21.16769 | 4.61E-14 | *** |
| Control_Medium - P331 | 30.86569 | 1.55159 | 30 | 19.89294 | 4.61E-14 | *** |
| Control_Medium - P7 | 34.68109 | 1.55159 | 30 | 22.35196 | 4.61E-14 | *** |
| P10 - P148 | 14.32816 | 1.55159 | 30 | 9.234499 | 4.1E-09 | *** |
| P10 - P159 | 17.74655 | 1.55159 | 30 | 11.43766 | 2.69E-11 | *** |
| P10 - P331 | 15.76866 | 1.55159 | 30 | 10.1629 | 4.57E-10 | *** |
| P10 - P7 | 19.58406 | 1.55159 | 30 | 12.62193 | 2.35E-12 | *** |
| P148 - P159 | 3.418395 | 1.55159 | 30 | 2.203156 | 0.266012 |  |
| P148 - P331 | 1.440503 | 1.55159 | 30 | 0.928405 | 0.935955 |  |
| P148 - P7 | 5.255902 | 1.55159 | 30 | 3.387429 | 0.022153 | * |
| P159 - P331 | -1.97789 | 1.55159 | 30 | -1.27475 | 0.796033 |  |
| P159 - P7 | 1.837507 | 1.55159 | 30 | 1.184273 | 0.840755 |  |
| P331 - P7 | 3.815398 | 1.55159 | 30 | 2.459025 | 0.168809 |  |

| Sample | mean | SE | lower.CL | upper.CL | group |
| --- | --- | --- | --- | --- | --- |
| P7 | 2.114307 | 1.09714 | -0.97527 | 5.203881 | a |
| P159 | 3.951813 | 1.09714 | 0.862239 | 7.041388 | ab |
| P331 | 5.929705 | 1.09714 | 2.84013 | 9.01928 | ab |
| P148 | 7.370208 | 1.09714 | 4.280634 | 10.45978 | b |
| P10 | 21.69837 | 1.09714 | 18.60879 | 24.78794 | c |
| Control_Medium | 36.79539 | 1.09714 | 33.70582 | 39.88497 | d |

**Protocatechuic acid_Medium**

| Info | Value |
| --- | --- |
| Best model family | gaussian |
| Model name | Normal_log |

| Resid. Df | Resid. Dev | Df | Deviance | Pr(>Chi) |
| --- | --- | --- | --- | --- |
| 35 | 162.6567 |  |  |  |
| 30 | 3.613665 | 5 | 159.043 | 2.5E-283 |

| contrast | estimate | SE | df | t.ratio | p.value | Signif |
| --- | --- | --- | --- | --- | --- | --- |
| Control_Medium - P10 | -0.01413 | 0.200379 | 30 | -0.07052 | 0.944245 |  |
| Control_Medium - P148 | 0.594877 | 0.200379 | 30 | 2.968758 | 0.006246 | ** |
| Control_Medium - P159 | 5.220715 | 0.200379 | 30 | 26.05417 | 2.77E-21 | *** |
| Control_Medium - P331 | 1.428736 | 0.200379 | 30 | 7.130159 | 9.36E-08 | *** |
| Control_Medium - P7 | 4.408016 | 0.200379 | 30 | 21.99837 | 1.39E-19 | *** |
| P10 - P148 | 0.609009 | 0.200379 | 30 | 3.039281 | 0.005633 | ** |
| P10 - P159 | 5.234846 | 0.200379 | 30 | 26.1247 | 2.77E-21 | *** |
| P10 - P331 | 1.442867 | 0.200379 | 30 | 7.200682 | 8.61E-08 | *** |
| P10 - P7 | 4.422147 | 0.200379 | 30 | 22.06889 | 1.39E-19 | *** |
| P148 - P159 | 4.625838 | 0.200379 | 30 | 23.08541 | 5.89E-20 | *** |
| P148 - P331 | 0.833858 | 0.200379 | 30 | 4.1614 | 0.000334 | *** |
| P148 - P7 | 3.813139 | 0.200379 | 30 | 19.02961 | 6.75E-18 | *** |
| P159 - P331 | -3.79198 | 0.200379 | 30 | -18.924 | 6.75E-18 | *** |
| P159 - P7 | -0.8127 | 0.200379 | 30 | -4.0558 | 0.000409 | *** |
| P331 - P7 | 2.97928 | 0.200379 | 30 | 14.86821 | 4.16E-15 | *** |

| Sample | mean | SE | lower.CL | upper.CL | group |
| --- | --- | --- | --- | --- | --- |
| P159 | 7.304015 | 0.14169 | 4.900919 | 10.88543 | a |
| P7 | 16.46313 | 0.14169 | 11.04659 | 24.53559 | b |
| P331 | 323.8899 | 0.14169 | 217.3268 | 482.7047 | c |
| P148 | 745.6541 | 0.14169 | 500.3263 | 1111.275 | d |
| Control_Medium | 1351.728 | 0.14169 | 906.9959 | 2014.529 | e |
| P10 | 1370.966 | 0.14169 | 919.9039 | 2043.199 | e |

**Quinic acid_Biomass**

| Info | Value |
| --- | --- |
| Best model family | gaussian |
| Model name | Normal_log |

| Effect | Df | Deviance | Resid. Df | Resid. Dev | Pr(>Chi) |
| --- | --- | --- | --- | --- | --- |
| NULL |  |  | 35 | 103.4255 |  |
| Sample | 5 | 99.05802 | 30 | 4.36749 | 8.4E-145 |

| Comparison | Estimate | SE | df | t.ratio | p_value | Significance |
| --- | --- | --- | --- | --- | --- | --- |
| Control_Medium - P10 | 1.603353 | 0.22029 | 30 | 7.278375 | 5.97E-07 | *** |
| Control_Medium - P148 | 0.023702 | 0.22029 | 30 | 0.107594 | 0.999998 |  |
| Control_Medium - P159 | 4.167994 | 0.22029 | 30 | 18.92049 | 4.61E-14 | *** |
| Control_Medium - P331 | 3.687117 | 0.22029 | 30 | 16.73756 | 4.74E-14 | *** |
| Control_Medium - P7 | 2.966194 | 0.22029 | 30 | 13.46495 | 4.93E-13 | *** |
| P10 - P148 | -1.57965 | 0.22029 | 30 | -7.17078 | 7.96E-07 | *** |
| P10 - P159 | 2.564641 | 0.22029 | 30 | 11.64212 | 1.74E-11 | *** |
| P10 - P331 | 2.083764 | 0.22029 | 30 | 9.459186 | 2.39E-09 | *** |
| P10 - P7 | 1.362841 | 0.22029 | 30 | 6.186576 | 1.16E-05 | *** |
| P148 - P159 | 4.144292 | 0.22029 | 30 | 18.8129 | 4.61E-14 | *** |
| P148 - P331 | 3.663415 | 0.22029 | 30 | 16.62997 | 4.79E-14 | *** |
| P148 - P7 | 2.942492 | 0.22029 | 30 | 13.35736 | 5.94E-13 | *** |
| P159 - P331 | -0.48088 | 0.22029 | 30 | -2.18293 | 0.275075 |  |
| P159 - P7 | -1.2018 | 0.22029 | 30 | -5.45554 | 8.77E-05 | *** |
| P331 - P7 | -0.72092 | 0.22029 | 30 | -3.27261 | 0.029207 | * |

| Sample | mean_Value | SE | df | lower.CL | upper.CL | Group |
| --- | --- | --- | --- | --- | --- | --- |
| P159 | 5.544312 | 0.155769 | 30 | 3.575569 | 8.597065 | a |
| P331 | 8.967886 | 0.155769 | 30 | 5.783457 | 13.90569 | a |
| P7 | 18.44094 | 0.155769 | 30 | 11.8927 | 28.5947 | b |
| P10 | 72.05385 | 0.155769 | 30 | 46.46807 | 111.7274 | c |
| P148 | 349.6962 | 0.155769 | 30 | 225.5217 | 542.2424 | d |
| Control_Medium | 358.0837 | 0.155769 | 30 | 230.9309 | 555.2481 | d |

**Quinic acid_Medium**

| Info | Value |
| --- | --- |
| Best model family | gaussian |
| Model name | Sqrt_Normal |

| Effect | Df | Deviance | Resid. Df | Resid. Dev | Pr(>Chi) |
| --- | --- | --- | --- | --- | --- |
| NULL |  |  | 35 | 1653.812 |  |
| Sample | 5 | 1611.335 | 30 | 42.47713 | 7.8E-244 |

| Comparison | Estimate | SE | df | t.ratio | p_value | Significance |
| --- | --- | --- | --- | --- | --- | --- |
| Control_Medium - P10 | -0.16161 | 0.686999 | 30 | -0.23524 | 0.99989 |  |
| Control_Medium - P148 | 1.166855 | 0.686999 | 30 | 1.69848 | 0.543293 |  |
| Control_Medium - P159 | 15.699 | 0.686999 | 30 | 22.85155 | 4.61E-14 | *** |
| Control_Medium - P331 | 14.80124 | 0.686999 | 30 | 21.54477 | 4.61E-14 | *** |
| Control_Medium - P7 | 5.873955 | 0.686999 | 30 | 8.55016 | 2.22E-08 | *** |
| P10 - P148 | 1.328465 | 0.686999 | 30 | 1.933721 | 0.402437 |  |
| P10 - P159 | 15.86061 | 0.686999 | 30 | 23.08679 | 4.61E-14 | *** |
| P10 - P331 | 14.96285 | 0.686999 | 30 | 21.78001 | 4.61E-14 | *** |
| P10 - P7 | 6.035565 | 0.686999 | 30 | 8.785401 | 1.23E-08 | *** |
| P148 - P159 | 14.53214 | 0.686999 | 30 | 21.15307 | 4.61E-14 | *** |
| P148 - P331 | 13.63439 | 0.686999 | 30 | 19.84629 | 4.61E-14 | *** |
| P148 - P7 | 4.7071 | 0.686999 | 30 | 6.85168 | 1.88E-06 | *** |
| P159 - P331 | -0.89776 | 0.686999 | 30 | -1.30678 | 0.779039 |  |
| P159 - P7 | -9.82504 | 0.686999 | 30 | -14.3014 | 1.4E-13 | *** |
| P331 - P7 | -8.92729 | 0.686999 | 30 | -12.9946 | 1.15E-12 | *** |

| Sample | mean_Value | SE | df | lower.CL | upper.CL | Group |
| --- | --- | --- | --- | --- | --- | --- |
| P159 | 3.261763 | 0.485782 | 30 | 1.893788 | 4.629738 | a |
| P331 | 4.159519 | 0.485782 | 30 | 2.791545 | 5.527494 | a |
| P7 | 13.08681 | 0.485782 | 30 | 11.71883 | 14.45478 | b |
| P148 | 17.79391 | 0.485782 | 30 | 16.42593 | 19.16188 | c |
| Control_Medium | 18.96076 | 0.485782 | 30 | 17.59279 | 20.32874 | c |
| P10 | 19.12237 | 0.485782 | 30 | 17.7544 | 20.49035 | c |

**Catechin_Biomass**

| Info | Value |
| --- | --- |
| Best model family | gaussian |
| Model name | Sqrt_Normal |

| Effect | Df | Deviance | Resid. Df | Resid. Dev | Pr(>Chi) |
| --- | --- | --- | --- | --- | --- |
| NULL |  |  | 35 | 13392.17 |  |
| Sample | 5 | 12995.51 | 30 | 396.6627 | 3.1E-210 |

| Comparison | Estimate | SE | df | t.ratio | p_value | Significance |
| --- | --- | --- | --- | --- | --- | --- |
| Control_Medium - P10 | 21.62808 | 2.099372 | 30 | 10.30217 | 3.32E-10 | *** |
| Control_Medium - P148 | 51.13465 | 2.099372 | 30 | 24.35712 | 4.61E-14 | *** |
| Control_Medium - P159 | 46.19623 | 2.099372 | 30 | 22.00479 | 4.61E-14 | *** |
| Control_Medium - P331 | 48.45144 | 2.099372 | 30 | 23.07902 | 4.61E-14 | *** |
| Control_Medium - P7 | 49.24305 | 2.099372 | 30 | 23.45609 | 4.61E-14 | *** |
| P10 - P148 | 29.50657 | 2.099372 | 30 | 14.05495 | 1.94E-13 | *** |
| P10 - P159 | 24.56815 | 2.099372 | 30 | 11.70262 | 1.53E-11 | *** |
| P10 - P331 | 26.82336 | 2.099372 | 30 | 12.77685 | 1.74E-12 | *** |
| P10 - P7 | 27.61497 | 2.099372 | 30 | 13.15392 | 8.57E-13 | *** |
| P148 - P159 | -4.93842 | 2.099372 | 30 | -2.35233 | 0.205441 |  |
| P148 - P331 | -2.68321 | 2.099372 | 30 | -1.2781 | 0.794282 |  |
| P148 - P7 | -1.8916 | 2.099372 | 30 | -0.90103 | 0.943213 |  |
| P159 - P331 | 2.255214 | 2.099372 | 30 | 1.074233 | 0.887693 |  |
| P159 - P7 | 3.046819 | 2.099372 | 30 | 1.4513 | 0.69637 |  |
| P331 - P7 | 0.791604 | 2.099372 | 30 | 0.377067 | 0.998902 |  |

| Sample | mean_Value | SE | df | lower.CL | upper.CL | Group |
| --- | --- | --- | --- | --- | --- | --- |
| P148 | 4.26E-14 | 1.48448 | 30 | -4.18034 | 4.180335 | a |
| P7 | 1.891604 | 1.48448 | 30 | -2.28873 | 6.071939 | a |
| P331 | 2.683208 | 1.48448 | 30 | -1.49713 | 6.863544 | a |
| P159 | 4.938422 | 1.48448 | 30 | 0.758087 | 9.118758 | a |
| P10 | 29.50657 | 1.48448 | 30 | 25.32623 | 33.68691 | b |
| Control_Medium | 51.13465 | 1.48448 | 30 | 46.95432 | 55.31499 | c |

**Catechin_Medium**

| Info | Value |
| --- | --- |
| Best model family | gaussian |
| Model name | Sqrt_Normal |

| Effect | Df | Deviance | Resid. Df | Resid. Dev | Pr(>Chi) |
| --- | --- | --- | --- | --- | --- |
| NULL |  |  | 35 | 15533.77 |  |
| Sample | 5 | 14676.02 | 30 | 857.7485 | 1.1E-108 |

| Comparison | Estimate | SE | df | t.ratio | p_value | Significance |
| --- | --- | --- | --- | --- | --- | --- |
| Control_Medium - P10 | 0.143654 | 3.087157 | 30 | 0.046533 | 1 |  |
| Control_Medium - P148 | 31.63362 | 3.087157 | 30 | 10.24684 | 3.77E-10 | *** |
| Control_Medium - P159 | 37.50224 | 3.087157 | 30 | 12.14782 | 6.08E-12 | *** |
| Control_Medium - P331 | 46.44626 | 3.087157 | 30 | 15.04499 | 7.07E-14 | *** |
| Control_Medium - P7 | 49.07076 | 3.087157 | 30 | 15.89513 | 5.18E-14 | *** |
| P10 - P148 | 31.48996 | 3.087157 | 30 | 10.20031 | 4.2E-10 | *** |
| P10 - P159 | 37.35858 | 3.087157 | 30 | 12.10129 | 6.69E-12 | *** |
| P10 - P331 | 46.30261 | 3.087157 | 30 | 14.99846 | 7.29E-14 | *** |
| P10 - P7 | 48.92711 | 3.087157 | 30 | 15.8486 | 5.23E-14 | *** |
| P148 - P159 | 5.86862 | 3.087157 | 30 | 1.900979 | 0.421104 |  |
| P148 - P331 | 14.81264 | 3.087157 | 30 | 4.79815 | 0.000542 | *** |
| P148 - P7 | 17.43714 | 3.087157 | 30 | 5.648285 | 5.13E-05 | *** |
| P159 - P331 | 8.944023 | 3.087157 | 30 | 2.897171 | 0.068982 |  |
| P159 - P7 | 11.56852 | 3.087157 | 30 | 3.747306 | 0.009011 | ** |
| P331 - P7 | 2.6245 | 3.087157 | 30 | 0.850135 | 0.955243 |  |

| Sample | mean_Value | SE | df | lower.CL | upper.CL | Group |
| --- | --- | --- | --- | --- | --- | --- |
| P7 | 2.063892 | 2.18295 | 30 | -4.08335 | 8.211136 | a |
| P331 | 4.688392 | 2.18295 | 30 | -1.45885 | 10.83564 | ab |
| P159 | 13.63242 | 2.18295 | 30 | 7.485171 | 19.77966 | bc |
| P148 | 19.50103 | 2.18295 | 30 | 13.35379 | 25.64828 | c |
| P10 | 50.991 | 2.18295 | 30 | 44.84376 | 57.13824 | d |
| Control_Medium | 51.13465 | 2.18295 | 30 | 44.98741 | 57.2819 | d |

**Vanillic acid_Biomass**

| Info | Value |
| --- | --- |
| Best model family | gaussian |
| Model name | Sqrt_Normal |

| Effect | Df | Deviance | Resid. Df | Resid. Dev | Pr(>Chi) |
| --- | --- | --- | --- | --- | --- |
| NULL |  |  | 35 | 12642.86 |  |
| Sample | 5 | 12558.44 | 30 | 84.42595 | 0 |

| Comparison | Estimate | SE | df | t.ratio | p_value | Significance |
| --- | --- | --- | --- | --- | --- | --- |
| Control_Medium - P10 | 16.52008 | 0.968538 | 30 | 17.05672 | 4.7E-14 | *** |
| Control_Medium - P148 | 46.62666 | 0.968538 | 30 | 48.14127 | 4.61E-14 | *** |
| Control_Medium - P159 | 47.7422 | 0.968538 | 30 | 49.29305 | 4.61E-14 | *** |
| Control_Medium - P331 | 43.73857 | 0.968538 | 30 | 45.15937 | 4.61E-14 | *** |
| Control_Medium - P7 | 47.7422 | 0.968538 | 30 | 49.29305 | 4.61E-14 | *** |
| P10 - P148 | 30.10658 | 0.968538 | 30 | 31.08455 | 4.61E-14 | *** |
| P10 - P159 | 31.22212 | 0.968538 | 30 | 32.23634 | 4.61E-14 | *** |
| P10 - P331 | 27.21849 | 0.968538 | 30 | 28.10266 | 4.61E-14 | *** |
| P10 - P7 | 31.22212 | 0.968538 | 30 | 32.23634 | 4.61E-14 | *** |
| P148 - P159 | 1.115547 | 0.968538 | 30 | 1.151784 | 0.855511 |  |
| P148 - P331 | -2.88808 | 0.968538 | 30 | -2.9819 | 0.057195 |  |
| P148 - P7 | 1.115547 | 0.968538 | 30 | 1.151784 | 0.855511 |  |
| P159 - P331 | -4.00363 | 0.968538 | 30 | -4.13368 | 0.003284 | ** |
| P159 - P7 | -7.1E-14 | 0.968538 | 30 | -7.3E-14 | 1 |  |
| P331 - P7 | 4.003629 | 0.968538 | 30 | 4.133682 | 0.003284 | ** |

| Sample | mean_Value | SE | df | lower.CL | upper.CL | Group |
| --- | --- | --- | --- | --- | --- | --- |
| P159 | -5.7E-14 | 0.68486 | 30 | -1.92858 | 1.928584 | a |
| P7 | 1.42E-14 | 0.68486 | 30 | -1.92858 | 1.928584 | a |
| P148 | 1.115547 | 0.68486 | 30 | -0.81304 | 3.04413 | ab |
| P331 | 4.003629 | 0.68486 | 30 | 2.075045 | 5.932212 | b |
| P10 | 31.22212 | 0.68486 | 30 | 29.29354 | 33.15071 | c |
| Control_Medium | 47.7422 | 0.68486 | 30 | 45.81362 | 49.67079 | d |

**Vanillic acid_Medium**

| Info | Value |
| --- | --- |
| Best model family | gaussian |
| Model name | Sqrt_Normal |

| Effect | Df | Deviance | Resid. Df | Resid. Dev | Pr(>Chi) |
| --- | --- | --- | --- | --- | --- |
| NULL |  |  | 35 | 15468.19 |  |
| Sample | 5 | 15265.29 | 30 | 202.9009 | 0 |

| Comparison | Estimate | SE | df | t.ratio | p_value | Significance |
| --- | --- | --- | --- | --- | --- | --- |
| Control_Medium - P10 | -0.87867 | 1.501484 | 30 | -0.5852 | 0.991284 |  |
| Control_Medium - P148 | 16.01665 | 1.501484 | 30 | 10.66721 | 1.45E-10 | *** |
| Control_Medium - P159 | 46.58038 | 1.501484 | 30 | 31.0229 | 4.61E-14 | *** |
| Control_Medium - P331 | 40.14451 | 1.501484 | 30 | 26.73655 | 4.61E-14 | *** |
| Control_Medium - P7 | 46.77268 | 1.501484 | 30 | 31.15097 | 4.61E-14 | *** |
| P10 - P148 | 16.89532 | 1.501484 | 30 | 11.25241 | 4.01E-11 | *** |
| P10 - P159 | 47.45906 | 1.501484 | 30 | 31.6081 | 4.61E-14 | *** |
| P10 - P331 | 41.02318 | 1.501484 | 30 | 27.32176 | 4.61E-14 | *** |
| P10 - P7 | 47.65135 | 1.501484 | 30 | 31.73617 | 4.61E-14 | *** |
| P148 - P159 | 30.56374 | 1.501484 | 30 | 20.35569 | 4.61E-14 | *** |
| P148 - P331 | 24.12786 | 1.501484 | 30 | 16.06934 | 5.05E-14 | *** |
| P148 - P7 | 30.75604 | 1.501484 | 30 | 20.48376 | 4.61E-14 | *** |
| P159 - P331 | -6.43588 | 1.501484 | 30 | -4.28634 | 0.002183 | ** |
| P159 - P7 | 0.192298 | 1.501484 | 30 | 0.128072 | 0.999995 |  |
| P331 - P7 | 6.628174 | 1.501484 | 30 | 4.414415 | 0.001545 | ** |

| Sample | mean_Value | SE | df | lower.CL | upper.CL | Group |
| --- | --- | --- | --- | --- | --- | --- |
| P7 | 0.969521 | 1.06171 | 30 | -2.02028 | 3.959324 | a |
| P159 | 1.16182 | 1.06171 | 30 | -1.82798 | 4.151622 | a |
| P331 | 7.597695 | 1.06171 | 30 | 4.607893 | 10.5875 | b |
| P148 | 31.72556 | 1.06171 | 30 | 28.73575 | 34.71536 | c |
| Control_Medium | 47.7422 | 1.06171 | 30 | 44.7524 | 50.732 | d |
| P10 | 48.62088 | 1.06171 | 30 | 45.63107 | 51.61068 | d |

**Bioassays**

**Added supernatant**

**Figure 3 a**

***T. harzianum***

Welch Two Sample t-test

data: Value by Sample

t = -3.9639, df = 10.399, p-value = 0.002474

alternative hypothesis: true difference in means between group 2%SP_338 and group Control_338 is not equal to 0

95 percent confidence interval:

-25.348087 -7.166199

sample estimates:

mean in group 2%SP_338 mean in group Control_338

44.70000 60.95714

***C. globosum***

Welch Two Sample t-test

data: Value by Sample

t = -5.2526, df = 10.888, p-value = 0.0002809

alternative hypothesis: true difference in means between group 2%SP_7 and group Control_7 is not equal to 0

95 percent confidence interval:

-56.21446 -22.98554

sample estimates:

mean in group 2%SP_7 mean in group Control_7

51.7 91.3

***P. ulmarius***

Welch Two Sample t-test

data: Value by Sample

t = -3.2774, df = 9.7279, p-value = 0.008631

alternative hypothesis: true difference in means between group 2%SP_209 and group Control_209 is not equal to 0

95 percent confidence interval:

-6.982115 -1.317885

sample estimates:

mean in group 2%SP_209 mean in group Control_209

9.683333 13.833333

***A.hylecoeti***

Welch Two Sample t-test

data: Value by Sample

t = -1.4855, df = 10.042, p-value = 0.1681

alternative hypothesis: true difference in means between group 2%SP_10 and group Control_10 is not equal to 0

95 percent confidence interval:

-13.459402 2.687974

sample estimates:

mean in group 2%SP_10 mean in group Control_10

37.75714 43.14286

***P. ostreatus***

Welch Two Sample t-test

data: Value by Sample

t = -6.6892, df = 11.636, p-value = 2.598e-05

alternative hypothesis: true difference in means between group 2%SP_148 and group Control_148 is not equal to 0

95 percent confidence interval:

-14.405863 -7.308422

sample estimates:

mean in group 2%SP_148 mean in group Control_148

3.957143 14.814286

1. ***aegerita***

Welch Two Sample t-test

data: Value by Sample

t = -9.2467, df = 6.7086, p-value = 4.649e-05

alternative hypothesis: true difference in means between group 2%SP_147 and group Control_147 is not equal to 0

95 percent confidence interval:

-16.291027 -9.608973

sample estimates:

mean in group 2%SP_147 mean in group Control_147

19.88333 32.83333

Bioassays testing individual compounds

**Fig. 3 b-e** and **Suppl. Fig. 15**

***P. ostreatus***

| Info | Value |
| --- | --- |
| Best model family | gaussian |
| Model name | Normal_log |

| contrast | estimate | SE | df | t.ratio | p.value | Significance |
| --- | --- | --- | --- | --- | --- | --- |
| Caffeic acid - Control | 0.289296 | 0.100667 | 66 | 2.873799 | 0.046564 | * |
| Catechin - Control | -0.07863 | 0.104777 | 66 | -0.75046 | 0.956708 |  |
| Citronellol - Control | -0.64353 | 0.100667 | 66 | -6.39265 | 2.09E-07 | *** |
| Ferulic acid - Control | -0.09978 | 0.104777 | 66 | -0.95229 | 0.899251 |  |
| Galic acid - Control | 0.185043 | 0.09747 | 66 | 1.898453 | 0.361542 |  |
| Linalool - Control | -0.41234 | 0.104777 | 66 | -3.93537 | 0.002027 | ** |
| Phenyllactic acid - Control | -0.5089 | 0.104777 | 66 | -4.857 | 8.09E-05 | *** |
| Protocatechuic acid - Control | 0.278691 | 0.100667 | 66 | 2.768446 | 0.060759 |  |
| Syringic acid - Control | -0.16772 | 0.104777 | 66 | -1.60071 | 0.544485 |  |
| Terpineol - Control | -0.62435 | 0.104777 | 66 | -5.95879 | 1.18E-06 | *** |
| Vanillic acid - Control | -0.13487 | 0.104777 | 66 | -1.28721 | 0.740017 |  |

***T. harzianum***

| Info | Value |
| --- | --- |
| Best model family | gaussian |
| Model name | Normal_log |

| contrast | estimate | SE | df | t.ratio | p.value | Significance |
| --- | --- | --- | --- | --- | --- | --- |
| Caffeic acid - Control | -0.0605 | 0.074479 | 65 | -0.81229 | 0.942242 |  |
| Catechin - Control | 0.096717 | 0.07752 | 65 | 1.247646 | 0.762423 |  |
| Citronellol - Control | -0.69327 | 0.07752 | 65 | -8.94312 | 8.22E-12 | *** |
| Ferulic acid - Control | -0.40523 | 0.081587 | 65 | -4.96677 | 5.53E-05 | *** |
| Galic acid - Control | -0.14623 | 0.074479 | 65 | -1.96333 | 0.325992 |  |
| Linalool - Control | -0.29336 | 0.074479 | 65 | -3.93888 | 0.002027 | ** |
| Phenyllactic acid - Control | -0.34059 | 0.07752 | 65 | -4.39361 | 0.000435 | *** |
| Protocatechuic acid - Control | -0.0985 | 0.074479 | 65 | -1.3225 | 0.719423 |  |
| Syringic acid - Control | -0.17686 | 0.07752 | 65 | -2.28154 | 0.182206 |  |
| Terpineol - Control | -0.08243 | 0.074479 | 65 | -1.1068 | 0.834975 |  |
| Vanillic acid - Control | -0.20323 | 0.07752 | 65 | -2.62167 | 0.086809 |  |

***C. globosum***

| Info | Value |
| --- | --- |
| Best model family | gaussian |
| Model name | Normal_log |

| contrast | estimate | SE | df | t.ratio | p.value | Significance |
| --- | --- | --- | --- | --- | --- | --- |
| Caffeic_acid - Control | -0.5497 | 0.16528 | 67 | -3.32586 | 0.013374 | * |
| Catechin - Control | -0.51288 | 0.172029 | 67 | -2.98137 | 0.035016 | * |
| Citronellol - Control | 0.118811 | 0.16528 | 67 | 0.718845 | 0.963092 |  |
| Ferulic_acid - Control | 0.124298 | 0.172029 | 67 | 0.72254 | 0.962382 |  |
| Galic_acid - Control | -0.24469 | 0.16528 | 67 | -1.48047 | 0.621718 |  |
| Linalool - Control | -0.4049 | 0.172029 | 67 | -2.35367 | 0.156782 |  |
| Phenyllactic_acid - Control | -0.20428 | 0.16528 | 67 | -1.23593 | 0.768895 |  |
| Protocatechuic_acid - Control | -0.24992 | 0.16528 | 67 | -1.51212 | 0.601462 |  |
| Syringic_acid - Control | -0.09015 | 0.16528 | 67 | -0.54546 | 0.987169 |  |
| Terpineol - Control | -0.52327 | 0.172029 | 67 | -3.04175 | 0.029772 | * |
| Vanillic_acid - Control | 0.29564 | 0.172029 | 67 | 1.718548 | 0.469267 |  |

1. ***hylecoeti***

| Info | Value |
| --- | --- |
| Best model family | gaussian |
| Model name | Normal_log |

| contrast | estimate | SE | df | t.ratio | p.value | Significance |
| --- | --- | --- | --- | --- | --- | --- |
| Caffeic acid - Control | -0.24917 | 0.133979 | 66 | -1.85976 | 0.383746 |  |
| Catechin - Control | 0.293319 | 0.144023 | 66 | 2.036608 | 0.28789 |  |
| Citronellol - Control | 0.35248 | 0.144023 | 66 | 2.447384 | 0.12855 |  |
| Ferulic acid - Control | 0.128422 | 0.144023 | 66 | 0.891679 | 0.919658 |  |
| Galic acid - Control | -0.23898 | 0.138373 | 66 | -1.72708 | 0.464045 |  |
| Linalool - Control | 0.238121 | 0.144023 | 66 | 1.653354 | 0.510696 |  |
| Phenyllactic acid - Control | 0.092815 | 0.144023 | 66 | 0.644446 | 0.975534 |  |
| Protocatechuic acid - Control | -0.24264 | 0.133979 | 66 | -1.811 | 0.412573 |  |
| Syringic acid - Control | -0.01146 | 0.144023 | 66 | -0.07955 | 0.999996 |  |
| Terpineol - Control | 0.043544 | 0.144023 | 66 | 0.302343 | 0.998817 |  |
| Vanillic acid - Control | -0.10332 | 0.144023 | 66 | -0.71738 | 0.963361 |  |

***A. aegerita***

| Info | Value |
| --- | --- |
| Best model family | gaussian |
| Model name | Normal_log |

| contrast | estimate | SE | df | t.ratio | p.value | Significance |
| --- | --- | --- | --- | --- | --- | --- |
| Caffeic acid - Control | -0.53962 | 0.091046 | 65 | -5.92693 | 1.4E-06 | *** |
| Catechin - Control | 0.047871 | 0.094763 | 65 | 0.505168 | 0.99051 |  |
| Citronellol - Control | -0.3466 | 0.094763 | 65 | -3.65751 | 0.004981 | ** |
| Ferulic acid - Control | 0.036698 | 0.099735 | 65 | 0.367949 | 0.997351 |  |
| Galic acid - Control | -0.35352 | 0.091046 | 65 | -3.88288 | 0.002432 | ** |
| Linalool - Control | -0.01592 | 0.091046 | 65 | -0.17484 | 0.999879 |  |
| Phenyllactic acid - Control | -0.47818 | 0.091046 | 65 | -5.25206 | 1.9E-05 | *** |
| Protocatechuic acid - Control | -0.48917 | 0.091046 | 65 | -5.37284 | 1.2E-05 | *** |
| Syringic acid - Control | -0.23757 | 0.094763 | 65 | -2.50704 | 0.112884 |  |
| Terpineol - Control | -0.31183 | 0.094763 | 65 | -3.29059 | 0.014987 | * |
| Vanillic acid - Control | -0.13368 | 0.094763 | 65 | -1.41071 | 0.665802 |  |

***P. ulmarius***

| Info | Value |
| --- | --- |
| Best model family | gaussian |
| Model name | Normal_log |

| contrast | estimate | SE | df | t.ratio | p.value | Significance |
| --- | --- | --- | --- | --- | --- | --- |
| Caffeic acid - Control | -0.13188 | 0.143233 | 66 | -0.92077 | 0.910207 |  |
| Catechin - Control | 0.256664 | 0.149082 | 66 | 1.721628 | 0.467456 |  |
| Citronellol - Control | -0.56985 | 0.149082 | 66 | -3.82238 | 0.002927 | ** |
| Ferulic acid - Control | 0.801861 | 0.149082 | 66 | 5.378663 | 1.14E-05 | *** |
| Galic acid - Control | -0.19139 | 0.143233 | 66 | -1.33618 | 0.711276 |  |
| Linalool - Control | -0.05217 | 0.149082 | 66 | -0.34992 | 0.997843 |  |
| Phenyllactic acid - Control | -0.87189 | 0.143233 | 66 | -6.08718 | 7.09E-07 | *** |
| Protocatechuic acid - Control | -0.12202 | 0.143233 | 66 | -0.85189 | 0.931564 |  |
| Syringic acid - Control | 0.45817 | 0.143233 | 66 | 3.19877 | 0.019368 | * |
| Terpineol - Control | -0.13757 | 0.149082 | 66 | -0.92277 | 0.909535 |  |
| Vanillic acid - Control | 0.086433 | 0.149082 | 66 | 0.579769 | 0.983714 |  |

Phenol blend bioassay

**Fig. 3 f**

***R. sulphurea***

Welch Two Sample t-test

data: Value by Sample

t = -0.47535, df = 6.0389, p-value = 0.6512

alternative hypothesis: true difference in means between group 1_159_Control and group 2_159_Mix is not equal to 0

95 percent confidence interval:

-32.06918 21.62251

sample estimates:

mean in group 1_159_Control mean in group 2_159_Mix

37.71667 42.94000

***T. harzianum***

Welch Two Sample t-test

data: Value by Sample

t = 4.2714, df = 7.8447, p-value = 0.002844

alternative hypothesis: true difference in means between group 338_Control and group 338_Phenol-blend is not equal to 0

95 percent confidence interval:

9.440249 31.759751

sample estimates:

mean in group 338_Control mean in group 338_Phenol-blend

74.5 53.9

***A. hylecoeti***

Welch Two Sample t-test

data: Value by Sample

t = -0.84997, df = 7.3505, p-value = 0.4222

alternative hypothesis: true difference in means between group 10_Control and group 10_Phenol-blend is not equal to 0

95 percent confidence interval:

-10.202010 4.768677

sample estimates:

mean in group 10_Control mean in group 10_Phenol-blend

21.25000 23.96667

***E. polonica***

Welch Two Sample t-test

data: Value by Sample

t = 0.19194, df = 9.0498, p-value = 0.852

alternative hypothesis: true difference in means between group 331_Control and group 331_Phenol-blend is not equal to 0

95 percent confidence interval:

-5.92663 7.02663

sample estimates:

mean in group 331_Control mean in group 331_Phenol-blend

19.63333 19.08333

***P. ostreatus***

Welch Two Sample t-test

data: Value by Sample

t = 4.2798, df = 7.5479, p-value = 0.003074

alternative hypothesis: true difference in means between group 148_Control and group 148_Phenol-blend is not equal to 0

95 percent confidence interval:

4.631056 15.702277

sample estimates:

mean in group 148_Control mean in group 148_Phenol-blend

19.16667 9.00000

***P. ulmarius***

Welch Two Sample t-test

data: Value by Sample

t = 5.4758, df = 8.8673, p-value = 0.0004132

alternative hypothesis: true difference in means between group 209_Control and group 209_Phenol-blend is not equal to 0

95 percent confidence interval:

18.59368 44.87298

sample estimates:

mean in group 209_Control mean in group 209_Phenol-blend

63.40000 31.66667

***C. globosum***

Welch Two Sample t-test

data: Value by Sample

t = 10.3, df = 7.9988, p-value = 6.811e-06

alternative hypothesis: true difference in means between group 7_Control and group 7_Phenol-blend is not equal to 0

95 percent confidence interval:

21.52944 33.95056

sample estimates:

mean in group 7_Control mean in group 7_Phenol-blend

61.76 34.02

Identification of pH of filamentous fungi

**Fig. 4 a**

| Info | Value |
| --- | --- |
| Best model family | gaussian |
| Model name | Normal_log |

| Effect | Df | Deviance | Resid. Df | Resid. Dev | Pr(>Chi) |
| --- | --- | --- | --- | --- | --- |
| NULL |  |  | 34 | 0.544338 |  |
| Sample | 6 | 0.523455 | 28 | 0.020883 | 2.4E-148 |

| Comparison | Estimate | SE | df | t.ratio | p_value | Significance |
| --- | --- | --- | --- | --- | --- | --- |
| Control - P10 | 0.250977 | 0.017272 | 28 | 14.53068 | 2.79E-13 | *** |
| Control - P148 | -0.02425 | 0.017272 | 28 | -1.40398 | 0.795135 |  |
| Control - P150 | -0.16917 | 0.017272 | 28 | -9.79441 | 3.05E-09 | *** |
| Control - P159 | -0.00379 | 0.017272 | 28 | -0.21945 | 0.999989 |  |
| Control - P339 | -0.11238 | 0.017272 | 28 | -6.50643 | 9.09E-06 | *** |
| Control - P7 | 0.012462 | 0.017272 | 28 | 0.7215 | 0.990049 |  |
| P10 - P148 | -0.27523 | 0.017272 | 28 | -15.9347 | 1.32E-14 | *** |
| P10 - P150 | -0.42015 | 0.017272 | 28 | -24.3251 | 0 | *** |
| P10 - P159 | -0.25477 | 0.017272 | 28 | -14.7501 | 1.87E-13 | *** |
| P10 - P339 | -0.36336 | 0.017272 | 28 | -21.0371 | 0 | *** |
| P10 - P7 | -0.23851 | 0.017272 | 28 | -13.8092 | 1.02E-12 | *** |
| P148 - P150 | -0.14492 | 0.017272 | 28 | -8.39043 | 7.9E-08 | *** |
| P148 - P159 | 0.020459 | 0.017272 | 28 | 1.184531 | 0.894124 |  |
| P148 - P339 | -0.08813 | 0.017272 | 28 | -5.10245 | 0.000379 | *** |
| P148 - P7 | 0.036712 | 0.017272 | 28 | 2.125481 | 0.366065 |  |
| P150 - P159 | 0.165381 | 0.017272 | 28 | 9.574958 | 4.99E-09 | *** |
| P150 - P339 | 0.056791 | 0.017272 | 28 | 3.287978 | 0.038431 | * |
| P150 - P7 | 0.181633 | 0.017272 | 28 | 10.51591 | 6.31E-10 | *** |
| P159 - P339 | -0.10859 | 0.017272 | 28 | -6.28698 | 1.62E-05 | *** |
| P159 - P7 | 0.016252 | 0.017272 | 28 | 0.94095 | 0.962258 |  |
| P339 - P7 | 0.124842 | 0.017272 | 28 | 7.22793 | 1.41E-06 | *** |

| Sample | mean_Value | SE | df | lower.CL | upper.CL | Group |
| --- | --- | --- | --- | --- | --- | --- |
| P10 | 3.796392 | 0.012213 | 28 | 3.664581 | 3.932944 | a |
| P7 | 4.818999 | 0.012213 | 28 | 4.651682 | 4.992333 | b |
| Control | 4.879428 | 0.012213 | 28 | 4.710014 | 5.054936 | b |
| P159 | 4.897958 | 0.012213 | 28 | 4.727901 | 5.074132 | b |
| P148 | 4.9992 | 0.012213 | 28 | 4.825627 | 5.179016 | b |
| P339 | 5.45978 | 0.012213 | 28 | 5.270216 | 5.656162 | c |
| P150 | 5.778818 | 0.012213 | 28 | 5.578177 | 5.986675 | d |

Bioassay pH

**Fig. 4 b** and **Suppl. Fig. 15 g**

***R. sulphurea***

Welch Two Sample t-test

data: Value by Sample

t = -1.4064, df = 9.4944, p-value = 0.1915

alternative hypothesis: true difference in means between group pH3.5 and group pH5.0 is not equal to 0

95 percent confidence interval:

-5.191544 1.191544

sample estimates:

mean in group pH3.5 mean in group pH5.0

7.333333 9.333333

***A. aegerita***

Welch Two Sample t-test

data: Value by Sample

t = -5.5141, df = 7.3004, p-value = 0.0007739

alternative hypothesis: true difference in means between group pH3.5 and group pH5.0 is not equal to 0

95 percent confidence interval:

-14.252807 -5.747193

sample estimates:

mean in group pH3.5 mean in group pH5.0

5.333333 15.333333

***P. ostreatus***

Welch Two Sample t-test

data: Value by Sample

t = -9.7823, df = 7.8976, p-value = 1.094e-05

alternative hypothesis: true difference in means between group pH3.5 and group pH5.0 is not equal to 0

95 percent confidence interval:

-20.81045 -12.85622

sample estimates:

mean in group pH3.5 mean in group pH5.0

3.50000 20.33333

***C. globosum***

Welch Two Sample t-test

data: Value by Sample

t = -7.2122, df = 6.2549, p-value = 0.0002977

alternative hypothesis: true difference in means between group 7-pH3.5 and group 7-pH5.0 is not equal to 0

95 percent confidence interval:

-36.73865 -18.26135

sample estimates:

mean in group 7-pH3.5 mean in group 7-pH5.0

5.666667 33.166667

***A. hylecoeti***

Welch Two Sample t-test

data: Value by Sample

t = 10.061, df = 7.7055, p-value = 1.06e-05

alternative hypothesis: true difference in means between group pH3.5 and group pH5.0 is not equal to 0

95 percent confidence interval:

33.97553 54.35781

sample estimates:

mean in group pH3.5 mean in group pH5.0

67.83333 23.66667

***P. nameko***

Welch Two Sample t-test

data: Value by Sample

t = -3.1911, df = 5.3437, p-value = 0.02209

alternative hypothesis: true difference in means between group pH3.5 and group pH5.0 is not equal to 0

95 percent confidence interval:

-24.048561 -2.818106

sample estimates:

mean in group pH3.5 mean in group pH5.0

13.16667 26.60000

**pH of liquid medium_Yeasts and *A.hylecoeti***

**Fig. 4c**

| Info | Value |
| --- | --- |
| Best model family | gaussian |
| Model name | Sqrt_Normal |

| Effect | Df | Deviance | Resid. Df | Resid. Dev | Pr(>Chi) |
| --- | --- | --- | --- | --- | --- |
| NULL |  |  | 20 | 0.614515 |  |
| Sample | 3 | 0.565737 | 17 | 0.048778 | 1.73E-42 |

| Comparison | Estimate | SE | df | t.ratio | p_value | Significance |
| --- | --- | --- | --- | --- | --- | --- |
| Control - IT130 | 0.22035 | 0.033878 | 17 | 6.504188 | 2.94E-05 | *** |
| Control - IT143 | 0.333397 | 0.033878 | 17 | 9.841059 | 1.1E-07 | *** |
| Control - P10 | 0.43939 | 0.032436 | 17 | 13.54642 | 8.81E-10 | *** |
| IT130 - IT143 | 0.113047 | 0.033878 | 17 | 3.336871 | 0.01852 | * |
| IT130 - P10 | 0.21904 | 0.032436 | 17 | 6.753021 | 1.85E-05 | *** |
| IT143 - P10 | 0.105993 | 0.032436 | 17 | 3.267773 | 0.021336 | * |

| Sample | mean_Value | SE | df | lower.CL | upper.CL | Group |
| --- | --- | --- | --- | --- | --- | --- |
| P10 | 1.769618 | 0.021868 | 17 | 1.708749 | 1.830487 | a |
| IT143 | 1.875611 | 0.023955 | 17 | 1.808932 | 1.942289 | b |
| IT130 | 1.988658 | 0.023955 | 17 | 1.921979 | 2.055336 | c |
| Control | 2.209007 | 0.023955 | 17 | 2.142329 | 2.275686 | d |

**Fig. 4d**

**Succinic acid**

| Info | Value |
| --- | --- |
| Best model family | gaussian |
| Model name | Normal_log |

| Effect | Df | Deviance | Resid. Df | Resid. Dev | Pr(>Chi) |
| --- | --- | --- | --- | --- | --- |
| NULL |  |  | 15 | 95.08281 |  |
| Sample | 2 | 88.59386 | 13 | 6.488945 | 2.87E-39 |

| Comparison | Estimate | SE | df | t.ratio | p_value | Significance |
| --- | --- | --- | --- | --- | --- | --- |
| IT130 - IT143 | -5.45636 | 0.446833 | 13 | -12.2112 | 4.8E-08 | *** |
| IT130 - P10 | -4.67166 | 0.42781 | 13 | -10.9199 | 1.82E-07 | *** |
| IT143 - P10 | 0.784708 | 0.42781 | 13 | 1.834243 | 0.19731 |  |

| Sample | mean_Value | SE | df | lower.CL | upper.CL | Group |
| --- | --- | --- | --- | --- | --- | --- |
| IT130 | 0.3 | 0.315959 | 13 | 0.126342 | 0.712353 | a |
| P10 | 32.06238 | 0.28843 | 13 | 14.55946 | 70.60678 | b |
| IT143 | 70.27324 | 0.315959 | 13 | 29.59484 | 166.8645 | b |

**Acetic acid**

| Info | Value |
| --- | --- |
| Best model family | gaussian |
| Model name | Sqrt_Normal |

| Effect | Df | Deviance | Resid. Df | Resid. Dev | Pr(>Chi) |
| --- | --- | --- | --- | --- | --- |
| NULL |  |  | 15 | 1218.76 |  |
| Sample | 2 | 1026.686 | 13 | 192.0741 | 8.14E-16 |

| Comparison | Estimate | SE | df | t.ratio | p_value | Significance |
| --- | --- | --- | --- | --- | --- | --- |
| IT130 - IT143 | 0.633942 | 2.431043 | 13 | 0.26077 | 0.963313 |  |
| IT130 - P10 | -16.2213 | 2.327547 | 13 | -6.96928 | 2.72E-05 | *** |
| IT143 - P10 | -16.8553 | 2.327547 | 13 | -7.24164 | 1.82E-05 | *** |

| Sample | mean_Value | SE | df | lower.CL | upper.CL | Group |
| --- | --- | --- | --- | --- | --- | --- |
| IT143 | 2.828427 | 1.719007 | 13 | -1.87656 | 7.533415 | a |
| IT130 | 3.462369 | 1.719007 | 13 | -1.24262 | 8.167357 | a |
| P10 | 19.68369 | 1.569232 | 13 | 15.38864 | 23.97874 | b |

Identification of major organic acids in yeasts

**Suppl. Fig. S16 d-f**

**PLA**

| Info | Value |
| --- | --- |
| Best model family | gaussian |
| Model name | Normal_log |

| Effect | Df | Deviance | Resid. Df | Resid. Dev | Pr(>Chi) |
| --- | --- | --- | --- | --- | --- |
| NULL |  |  | 14 | 12.11985 |  |
| Sample | 2 | 10.67593 | 12 | 1.443919 | 5.42E-20 |

| Comparison | Estimate | SE | df | t.ratio | p_value | Significance |
| --- | --- | --- | --- | --- | --- | --- |
| P10 - IT130 | -1.93683 | 0.219387 | 12 | -8.82838 | 3.77E-06 | *** |
| P10 - IT143 | -1.59235 | 0.219387 | 12 | -7.25818 | 2.77E-05 | *** |
| IT130 - IT143 | 0.344481 | 0.219387 | 12 | 1.570197 | 0.295252 |  |

| Sample | mean_Value | SE | df | lower.CL | upper.CL | Group |
| --- | --- | --- | --- | --- | --- | --- |
| P10 | 347.5429 | 0.15513 | 12 | 226.1348 | 534.1331 | a |
| IT143 | 1708.274 | 0.15513 | 12 | 1111.518 | 2625.418 | b |
| IT130 | 2410.813 | 0.15513 | 12 | 1568.637 | 3705.139 | b |

**Kynurenic acid**

| Info | Value |
| --- | --- |
| Best model family | gaussian |
| Model name | Normal_log |

| Effect | Df | Deviance | Resid. Df | Resid. Dev | Pr(>Chi) |
| --- | --- | --- | --- | --- | --- |
| NULL |  |  | 15 | 30.99443 |  |
| Sample | 2 | 29.47561 | 13 | 1.518819 | 1.64E-55 |

| Comparison | Estimate | SE | df | t.ratio | p_value | Significance |
| --- | --- | --- | --- | --- | --- | --- |
| IT130 - IT143 | 0.270901 | 0.216178 | 13 | 1.253138 | 0.444745 |  |
| IT130 - P10 | 2.93031 | 0.206975 | 13 | 14.15782 | 7.86E-09 | *** |
| IT143 - P10 | 2.659409 | 0.206975 | 13 | 12.84896 | 2.59E-08 | *** |

| Sample | mean_Value | SE | df | lower.CL | upper.CL | Group |
| --- | --- | --- | --- | --- | --- | --- |
| P10 | 23.9528 | 0.139542 | 13 | 16.34877 | 35.09356 | a |
| IT143 | 342.2338 | 0.152861 | 13 | 225.2268 | 520.0267 | b |
| IT130 | 448.7181 | 0.152861 | 13 | 295.305 | 681.8303 | b |

**IPM**

| Info | Value |
| --- | --- |
| Best model family | gaussian |
| Model name | Normal_log |

| Effect | Df | Deviance | Resid. Df | Resid. Dev | Pr(>Chi) |
| --- | --- | --- | --- | --- | --- |
| NULL |  |  | 15 | 7.692607 |  |
| Sample | 2 | 7.031426 | 13 | 0.66118 | 9.53E-31 |

| Comparison | Estimate | SE | df | t.ratio | p_value | Significance |
| --- | --- | --- | --- | --- | --- | --- |
| P10 - IT130 | 0.71256 | 0.13656 | 13 | 5.217922 | 0.000453 | *** |
| P10 - IT143 | -0.95778 | 0.13656 | 13 | -7.01358 | 2.55E-05 | *** |
| IT130 - IT143 | -1.67034 | 0.142632 | 13 | -11.7108 | 7.93E-08 | *** |

| Sample | mean_Value | SE | df | lower.CL | upper.CL | Group |
| --- | --- | --- | --- | --- | --- | --- |
| IT130 | 157.2401 | 0.100856 | 13 | 119.3101 | 207.2284 | a |
| P10 | 320.6448 | 0.092069 | 13 | 249.2205 | 412.5387 | b |
| IT143 | 835.5662 | 0.100856 | 13 | 634.0082 | 1101.202 | c |

Identification of major organic acids in filamentous fungi

**Suppl. Fig. S16 a-c**

**IPM**

| Info | Value |
| --- | --- |
| Best model family | gaussian |
| Model name | Sqrt_Normal |

| Effect | Df | Deviance | Resid. Df | Resid. Dev | Pr(>Chi) |
| --- | --- | --- | --- | --- | --- |
| NULL |  |  | 34 | 3535.722 |  |
| Sample | 6 | 3238.598 | 28 | 297.1242 | 6.3E-63 |

| Comparison | Estimate | SE | df | t.ratio | p_value | Significance |
| --- | --- | --- | --- | --- | --- | --- |
| Control - P10 | -15.1667 | 2.06025 | 28 | -7.36157 | 1E-06 | *** |
| Control - P148 | 5.06603 | 2.06025 | 28 | 2.458939 | 0.212666 |  |
| Control - P150 | -10.8527 | 2.06025 | 28 | -5.26764 | 0.000244 | *** |
| Control - P159 | -3.83943 | 2.06025 | 28 | -1.86357 | 0.519242 |  |
| Control - P339 | -25.6106 | 2.06025 | 28 | -12.4308 | 1.31E-11 | *** |
| Control - P7 | -2.78626 | 2.06025 | 28 | -1.35239 | 0.82133 |  |
| P10 - P148 | 20.23271 | 2.06025 | 28 | 9.820511 | 2.88E-09 | *** |
| P10 - P150 | 4.314018 | 2.06025 | 28 | 2.093929 | 0.383266 |  |
| P10 - P159 | 11.32725 | 2.06025 | 28 | 5.497999 | 0.000132 | *** |
| P10 - P339 | -10.444 | 2.06025 | 28 | -5.06926 | 0.000414 | *** |
| P10 - P7 | 12.38041 | 2.06025 | 28 | 6.00918 | 3.37E-05 | *** |
| P148 - P150 | -15.9187 | 2.06025 | 28 | -7.72658 | 4.01E-07 | *** |
| P148 - P159 | -8.90546 | 2.06025 | 28 | -4.32251 | 0.002972 | ** |
| P148 - P339 | -30.6767 | 2.06025 | 28 | -14.8898 | 1.44E-13 | *** |
| P148 - P7 | -7.85229 | 2.06025 | 28 | -3.81133 | 0.010939 | * |
| P150 - P159 | 7.013235 | 2.06025 | 28 | 3.40407 | 0.029343 | * |
| P150 - P339 | -14.758 | 2.06025 | 28 | -7.16319 | 1.66E-06 | *** |
| P150 - P7 | 8.066397 | 2.06025 | 28 | 3.915251 | 0.008434 | ** |
| P159 - P339 | -21.7712 | 2.06025 | 28 | -10.5673 | 5.66E-10 | *** |
| P159 - P7 | 1.053162 | 2.06025 | 28 | 0.511182 | 0.998474 |  |
| P339 - P7 | 22.82437 | 2.06025 | 28 | 11.07844 | 1.93E-10 | *** |

| Sample | mean_Value | SE | df | lower.CL | upper.CL | Group |
| --- | --- | --- | --- | --- | --- | --- |
| P148 | -1.2E-14 | 1.456817 | 28 | -4.21506 | 4.215058 | a |
| Control | 5.06603 | 1.456817 | 28 | 0.850971 | 9.281088 | ab |
| P7 | 7.852294 | 1.456817 | 28 | 3.637236 | 12.06735 | b |
| P159 | 8.905456 | 1.456817 | 28 | 4.690398 | 13.12051 | b |
| P150 | 15.91869 | 1.456817 | 28 | 11.70363 | 20.13375 | c |
| P10 | 20.23271 | 1.456817 | 28 | 16.01765 | 24.44777 | c |
| P339 | 30.67666 | 1.456817 | 28 | 26.4616 | 34.89172 | d |

**PLA**

| Info | Value |
| --- | --- |
| Best model family | gaussian |
| Model name | Sqrt_Normal |

| Effect | Df | Deviance | Resid. Df | Resid. Dev | Pr(>Chi) |
| --- | --- | --- | --- | --- | --- |
| NULL |  |  | 34 | 2471.11 |  |
| Sample | 6 | 1928.255 | 28 | 542.855 | 3.26E-19 |

| Comparison | Estimate | SE | df | t.ratio | p_value | Significance |
| --- | --- | --- | --- | --- | --- | --- |
| Control - P10 | -17.7543 | 2.784793 | 28 | -6.37545 | 1.28E-05 | *** |
| Control - P148 | 1.326193 | 2.784793 | 28 | 0.476227 | 0.998976 |  |
| Control - P150 | -10.3536 | 2.784793 | 28 | -3.7179 | 0.013784 | * |
| Control - P159 | 0.781313 | 2.784793 | 28 | 0.280564 | 0.999952 |  |
| Control - P339 | -4.76614 | 2.784793 | 28 | -1.71149 | 0.61442 |  |
| Control - P7 | 5.450637 | 2.784793 | 28 | 1.957286 | 0.462069 |  |
| P10 - P148 | 19.08051 | 2.784793 | 28 | 6.851679 | 3.7E-06 | *** |
| P10 - P150 | 7.400723 | 2.784793 | 28 | 2.657549 | 0.147117 |  |
| P10 - P159 | 18.53563 | 2.784793 | 28 | 6.656017 | 6.15E-06 | *** |
| P10 - P339 | 12.98818 | 2.784793 | 28 | 4.663965 | 0.001214 | ** |
| P10 - P7 | 23.20495 | 2.784793 | 28 | 8.332739 | 9.08E-08 | *** |
| P148 - P150 | -11.6798 | 2.784793 | 28 | -4.19413 | 0.004144 | ** |
| P148 - P159 | -0.54488 | 2.784793 | 28 | -0.19566 | 0.999994 |  |
| P148 - P339 | -6.09233 | 2.784793 | 28 | -2.18771 | 0.333397 |  |
| P148 - P7 | 4.124444 | 2.784793 | 28 | 1.48106 | 0.753203 |  |
| P150 - P159 | 11.13491 | 2.784793 | 28 | 3.998468 | 0.006835 | ** |
| P150 - P339 | 5.587455 | 2.784793 | 28 | 2.006417 | 0.433003 |  |
| P150 - P7 | 15.80423 | 2.784793 | 28 | 5.67519 | 8.2E-05 | *** |
| P159 - P339 | -5.54745 | 2.784793 | 28 | -1.99205 | 0.441425 |  |
| P159 - P7 | 4.669324 | 2.784793 | 28 | 1.676722 | 0.636132 |  |
| P339 - P7 | 10.21677 | 2.784793 | 28 | 3.668774 | 0.01555 | * |

| Sample | mean_Value | SE | df | lower.CL | upper.CL | Group |
| --- | --- | --- | --- | --- | --- | --- |
| P7 | -3.6E-15 | 1.969146 | 28 | -5.6974 | 5.697398 | a |
| P148 | 4.124444 | 1.969146 | 28 | -1.57295 | 9.821842 | ab |
| P159 | 4.669324 | 1.969146 | 28 | -1.02807 | 10.36672 | ab |
| Control | 5.450637 | 1.969146 | 28 | -0.24676 | 11.14803 | ab |
| P339 | 10.21677 | 1.969146 | 28 | 4.519377 | 15.91417 | bc |
| P150 | 15.80423 | 1.969146 | 28 | 10.10683 | 21.50163 | cd |
| P10 | 23.20495 | 1.969146 | 28 | 17.50755 | 28.90235 | d |

**Succinic acid**

| Info | Value |
| --- | --- |
| Best model family | gaussian |
| Model name | Sqrt_Normal |

| Effect | Df | Deviance | Resid. Df | Resid. Dev | Pr(>Chi) |
| --- | --- | --- | --- | --- | --- |
| NULL |  |  | 33 | 266.2104 |  |
| Sample | 6 | 265.469 | 27 | 0.7414 | 0 |

| Comparison | Estimate | SE | df | t.ratio | p_value | Significance |
| --- | --- | --- | --- | --- | --- | --- |
| Control - P10 | -5.85241 | 0.104803 | 27 | -55.8419 | 4.22E-15 | *** |
| Control - P148 | 2.172101 | 0.104803 | 27 | 20.72553 | 4.22E-15 | *** |
| Control - P150 | 1.982364 | 0.104803 | 27 | 18.91512 | 5.11E-15 | *** |
| Control - P159 | 2.093044 | 0.111161 | 27 | 18.82902 | 5.22E-15 | *** |
| Control - P339 | 2.172101 | 0.104803 | 27 | 20.72553 | 4.22E-15 | *** |
| Control - P7 | 2.172101 | 0.104803 | 27 | 20.72553 | 4.22E-15 | *** |
| P10 - P148 | 8.024506 | 0.104803 | 27 | 76.56742 | 4.22E-15 | *** |
| P10 - P150 | 7.834769 | 0.104803 | 27 | 74.75701 | 4.22E-15 | *** |
| P10 - P159 | 7.945449 | 0.111161 | 27 | 71.47726 | 4.22E-15 | *** |
| P10 - P339 | 8.024506 | 0.104803 | 27 | 76.56742 | 4.22E-15 | *** |
| P10 - P7 | 8.024506 | 0.104803 | 27 | 76.56742 | 4.22E-15 | *** |
| P148 - P150 | -0.18974 | 0.104803 | 27 | -1.81041 | 0.552754 |  |
| P148 - P159 | -0.07906 | 0.111161 | 27 | -0.7112 | 0.990729 |  |
| P148 - P339 | 4.44E-16 | 0.104803 | 27 | 4.24E-15 | 1 |  |
| P148 - P7 | 1.33E-15 | 0.104803 | 27 | 1.27E-14 | 1 |  |
| P150 - P159 | 0.11068 | 0.111161 | 27 | 0.995675 | 0.950615 |  |
| P150 - P339 | 0.189737 | 0.104803 | 27 | 1.81041 | 0.552754 |  |
| P150 - P7 | 0.189737 | 0.104803 | 27 | 1.81041 | 0.552754 |  |
| P159 - P339 | 0.079057 | 0.111161 | 27 | 0.711196 | 0.990729 |  |
| P159 - P7 | 0.079057 | 0.111161 | 27 | 0.711196 | 0.990729 |  |
| P339 - P7 | 8.88E-16 | 0.104803 | 27 | 8.47E-15 | 1 |  |

| Sample | mean_Value | SE | df | lower.CL | upper.CL | Group |
| --- | --- | --- | --- | --- | --- | --- |
| P7 | -2.7E-15 | 0.074107 | 27 | -0.21504 | 0.215037 | a |
| P339 | -1.8E-15 | 0.074107 | 27 | -0.21504 | 0.215037 | a |
| P148 | -1.3E-15 | 0.074107 | 27 | -0.21504 | 0.215037 | a |
| P159 | 0.079057 | 0.082854 | 27 | -0.16136 | 0.319475 | a |
| P150 | 0.189737 | 0.074107 | 27 | -0.0253 | 0.404773 | a |
| Control | 2.172101 | 0.074107 | 27 | 1.957064 | 2.387138 | b |
| P10 | 8.024506 | 0.074107 | 27 | 7.809469 | 8.239543 | c |

Bioassay succinic acid

**Suppl. Fig. 17**

***C. globosum***

Welch Two Sample t-test

data: Value by Sample

t = 2.5257, df = 5.203, p-value = 0.05095

alternative hypothesis: true difference in means between group P7-Control and group P7-Succinic acid is not equal to 0

95 percent confidence interval:

-0.1319384 44.4652717

sample estimates:

mean in group P7-Control mean in group P7-Succinic acid

50.16667 28.00000

***A. hylecoeti***

Welch Two Sample t-test

data: Value by Sample

t = 0.12022, df = 8.4115, p-value = 0.9071

alternative hypothesis: true difference in means between group P10-Control and group P10-Succinic acid is not equal to 0

95 percent confidence interval:

-18.01918 20.01918

sample estimates:

mean in group P10-Control mean in group P10-Succinic acid

42.16667 41.16667

***A. agerita***

Welch Two Sample t-test

data: Value by Sample

t = 1.6771, df = 9.3895, p-value = 0.1264

alternative hypothesis: true difference in means between group P147-Control and group P147-Succinic acid is not equal to 0

95 percent confidence interval:

-1.702047 11.702047

sample estimates:

mean in group P147-Control mean in group P147-Succinic acid

23.66667 18.66667

***P. ostreatus***

Welch Two Sample t-test

data: Value by Sample

t = 1.2702, df = 9.5283, p-value = 0.2342

alternative hypothesis: true difference in means between group P148-Control and group P148-Succinic acid is not equal to 0

95 percent confidence interval:

-3.19190 11.52523

sample estimates:

mean in group P148-Control mean in group P148-Succinic acid

28.66667 24.50000

***P. nameko***

Welch Two Sample t-test

data: Value by Sample

t = 0.93008, df = 6.8495, p-value = 0.3839

alternative hypothesis: true difference in means between group P208-Control and group P208-Succinic acid is not equal to 0

95 percent confidence interval:

-5.179167 11.845834

sample estimates:

mean in group P208-Control mean in group P208-Succinic acid

35.00000 31.66667

Bioassay acetic acid

**Fig. 4 e**

***T. harzianum***

| Info | Value |
| --- | --- |
| Best model family | gaussian |
| Model name | Normal_log |

| contrast | estimate | SE | df | t.ratio | p.value | Significance |
| --- | --- | --- | --- | --- | --- | --- |
| 338_0.0025% Acetic acid - Control | -0.18977 | 0.119919 | 21 | -1.58245 | 0.296012 |  |
| 338_0.05% Acetic acid - Control | -0.37827 | 0.119919 | 21 | -3.15441 | 0.013245 | * |
| 338_0.125% - Control | -0.97341 | 0.119919 | 21 | -8.11728 | 1.92E-07 | *** |

***R. sulphurea***

| Info | Value |
| --- | --- |
| Best model family | gaussian |
| Model name | Sqrt_Normal |

| contrast | estimate | SE | df | t.ratio | p.value | Significance |
| --- | --- | --- | --- | --- | --- | --- |
| 159_0.0025% Acetic acid - Control | -1.02144 | 0.376601 | 21 | -2.71227 | 0.035213 | * |
| 159_0.05% Acetic acid - Control | -1.22003 | 0.376601 | 21 | -3.23958 | 0.010913 | * |
| 159_0.125% Acetic acid - Control | -7.43721 | 0.362902 | 21 | -20.4937 | 3.52E-14 | *** |

***P. ulmarius***

| Info | Value |
| --- | --- |
| Best model family | gaussian |
| Model name | Sqrt_Normal |

| contrast | estimate | SE | df | t.ratio | p.value | Significance |
| --- | --- | --- | --- | --- | --- | --- |
| 209_0.0025% Acetic acid - Control | -0.78498 | 0.207041 | 22 | -3.79144 | 0.002845 | ** |
| 209_0.05% Acetic acid - Control | -0.85054 | 0.19951 | 22 | -4.26316 | 0.000911 | *** |
| 209_0.125% Acetic acid - Control | -4.7577 | 0.19951 | 22 | -23.847 | 1.57E-14 | *** |

***A. agerita***

| model |
| --- |
| Sqrt_Normal |

| contrast | estimate | SE | df | t.ratio | p.value | Signif |
| --- | --- | --- | --- | --- | --- | --- |
| 147_0.0025% Acetic acid - Control | -0.86776 | 0.229325 | 24 | -3.78397 | 0.002587 | ** |
| 147_0.05% Acetic acid - Control | -5.10864 | 0.229325 | 24 | -22.2768 | 1.41E-14 | *** |
| 147_0.125% Acetic acid - Control | -5.10864 | 0.229325 | 24 | -22.2768 | 1.41E-14 | *** |

***P. ostreatus***

| model |
| --- |
| Sqrt_Normal |

| contrast | estimate | SE | df | t.ratio | p.value | Signif |
| --- | --- | --- | --- | --- | --- | --- |
| 148_0.0025% Acetic acid - Control | 0.156168 | 0.327574 | 21 | 0.47674 | 0.906207 |  |
| 148_0.05% Acetic acid - Control | -1.81722 | 0.327574 | 21 | -5.5475 | 4.85E-05 | *** |
| 148_0.125% Acetic acid - Control | -3.72734 | 0.315659 | 21 | -11.8081 | 2.9E-10 | *** |

***C. globosum***

| model |
| --- |
| Sqrt_Normal |

| contrast | estimate | SE | df | t.ratio | p.value | Signif |
| --- | --- | --- | --- | --- | --- | --- |
| 7_0.0025% Acetic acid - Control | -0.32094 | 0.427425 | 23 | -0.75086 | 0.771708 |  |
| 7_0.05% Acetic acid - Control | -2.08809 | 0.444878 | 23 | -4.69362 | 0.000289 | *** |
| 7_0.125% Acetic acid - Control | -6.86014 | 0.427425 | 23 | -16.0499 | 1.76E-13 | *** |

**Fig. 4 f_*A.hylecoeti***

| model |
| --- |
| Normal_log |

| contrast | estimate | SE | df | t.ratio | p.value | Signif |
| --- | --- | --- | --- | --- | --- | --- |
| 10_0.0025% Acetic acid - Control | 0.274792 | 0.067822 | 23 | 4.051666 | 0.001417 | ** |
| 10_0.05% Acetic acid - Control | 0.03633 | 0.067822 | 23 | 0.535673 | 0.881359 |  |
| 10_0.125% Acetic acid - Control | 0.055721 | 0.067822 | 23 | 0.821571 | 0.730686 |  |
