# Supplementary figures and images for: Fungal symbiont of an ambrosia beetle possesses high nutrient content and suppresses competing fungi with antimicrobial compounds
Source: ISME J. 2025 Nov 20;19(1):wraf258. doi: 10.1093/ismejo/wraf258 (PMC12684719; doi:10.1093/ismejo/wraf258)

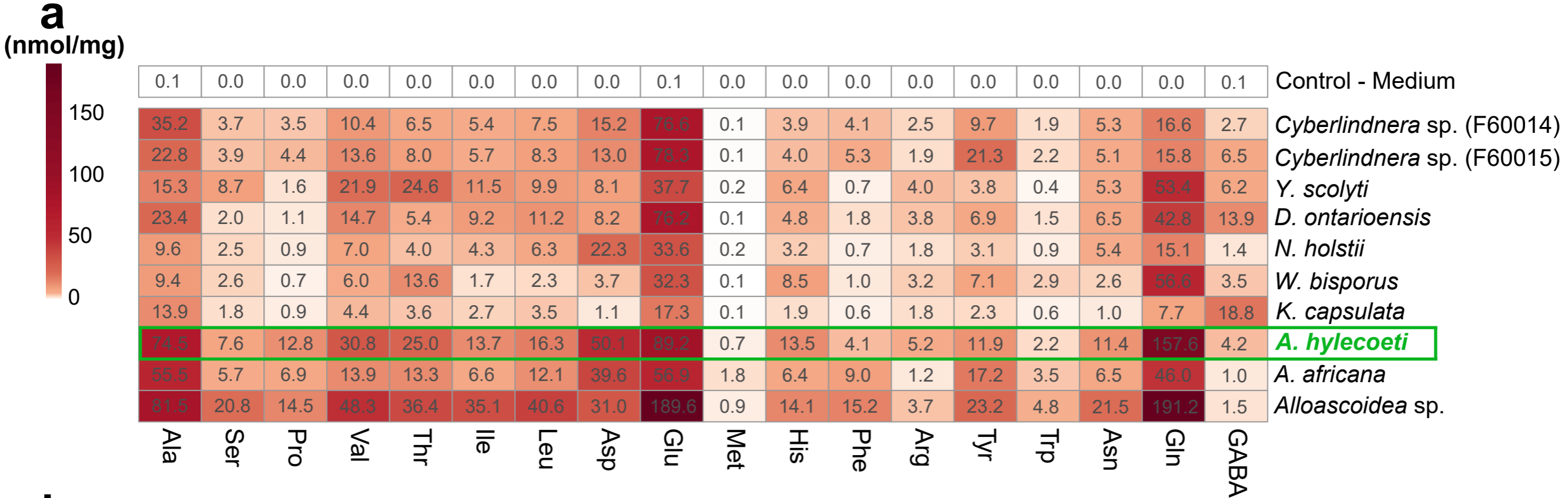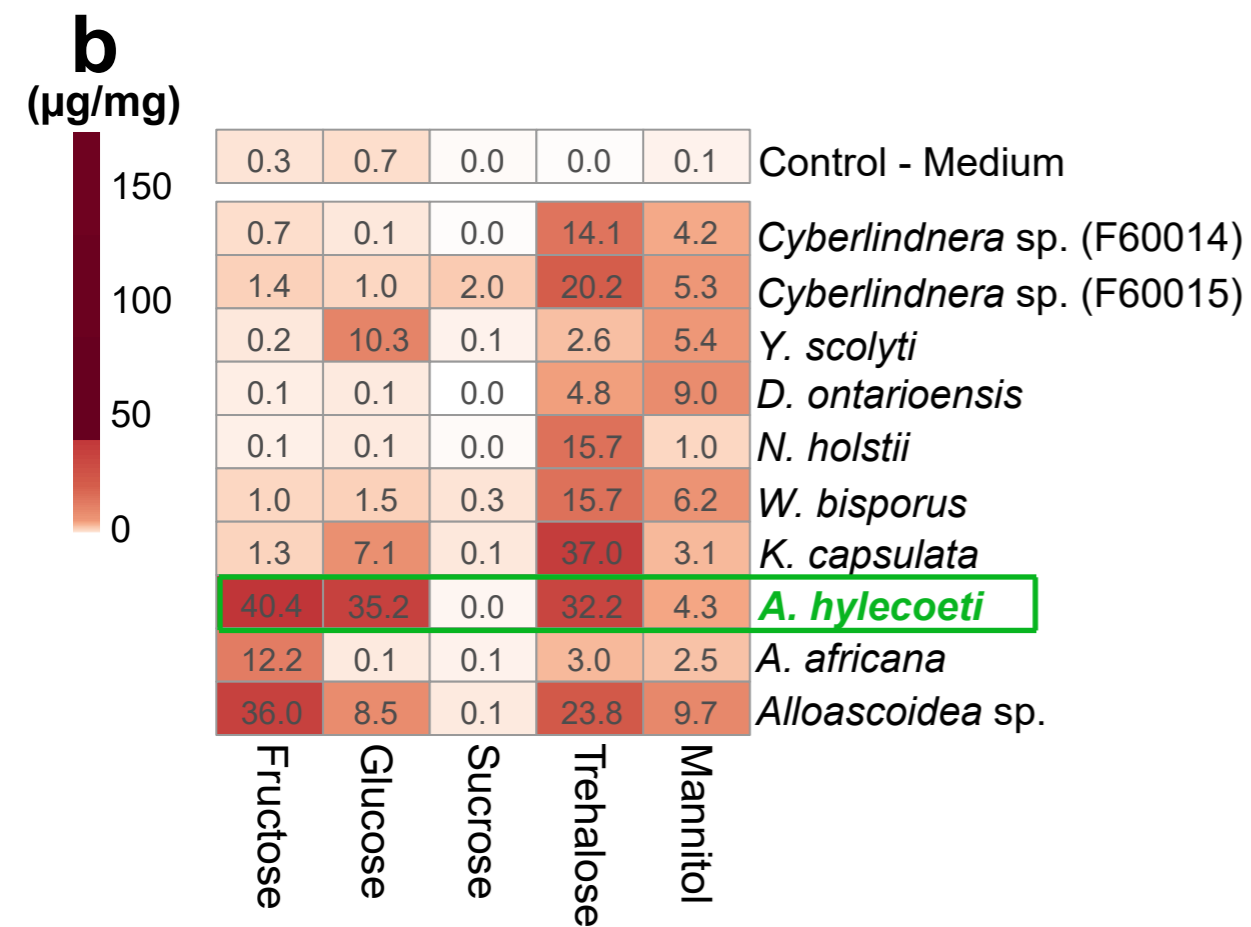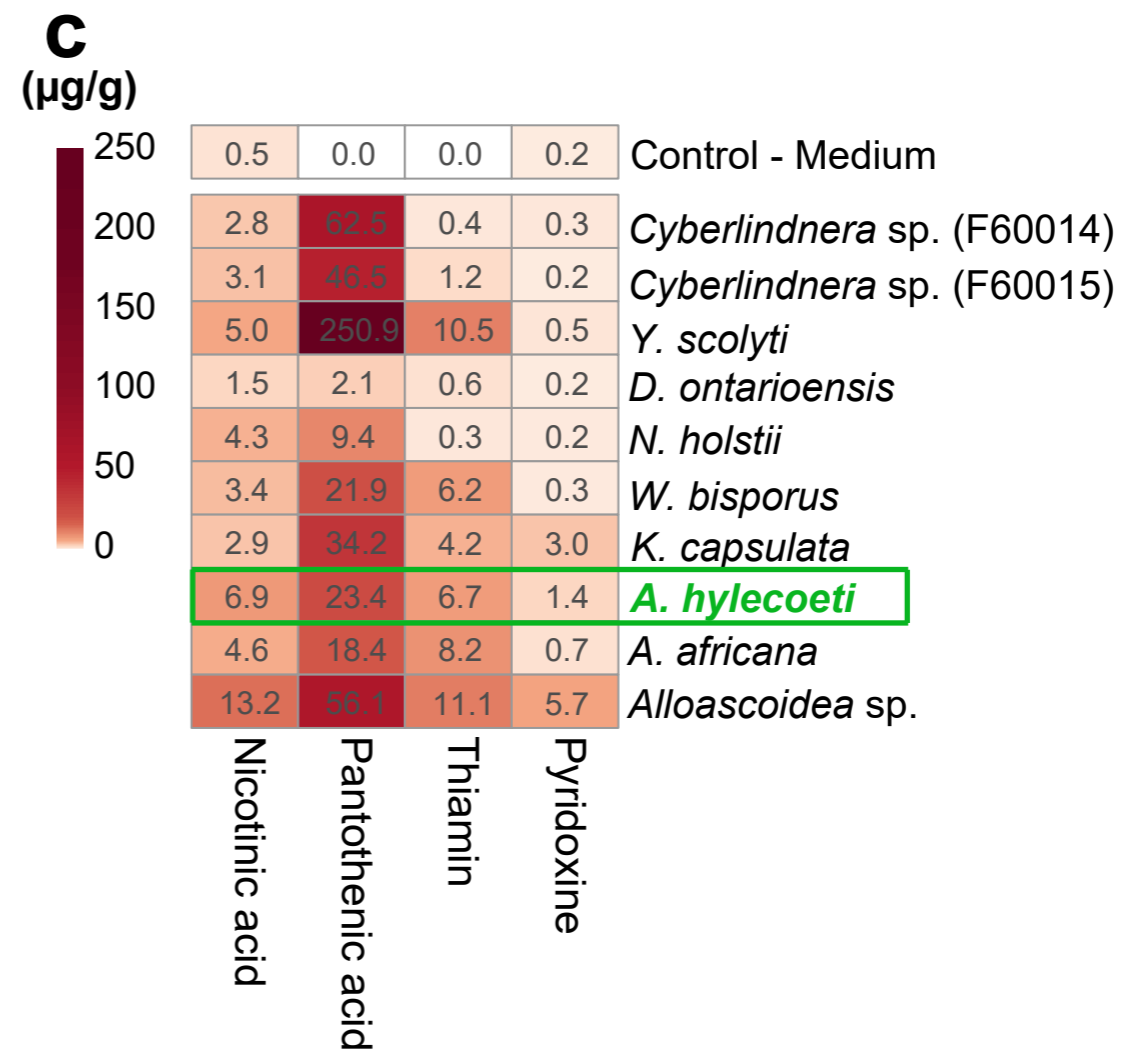

Supplement: suppl_wraf258 [file suppl_wraf258.zip › Suppl. Fig. 1.pdf]

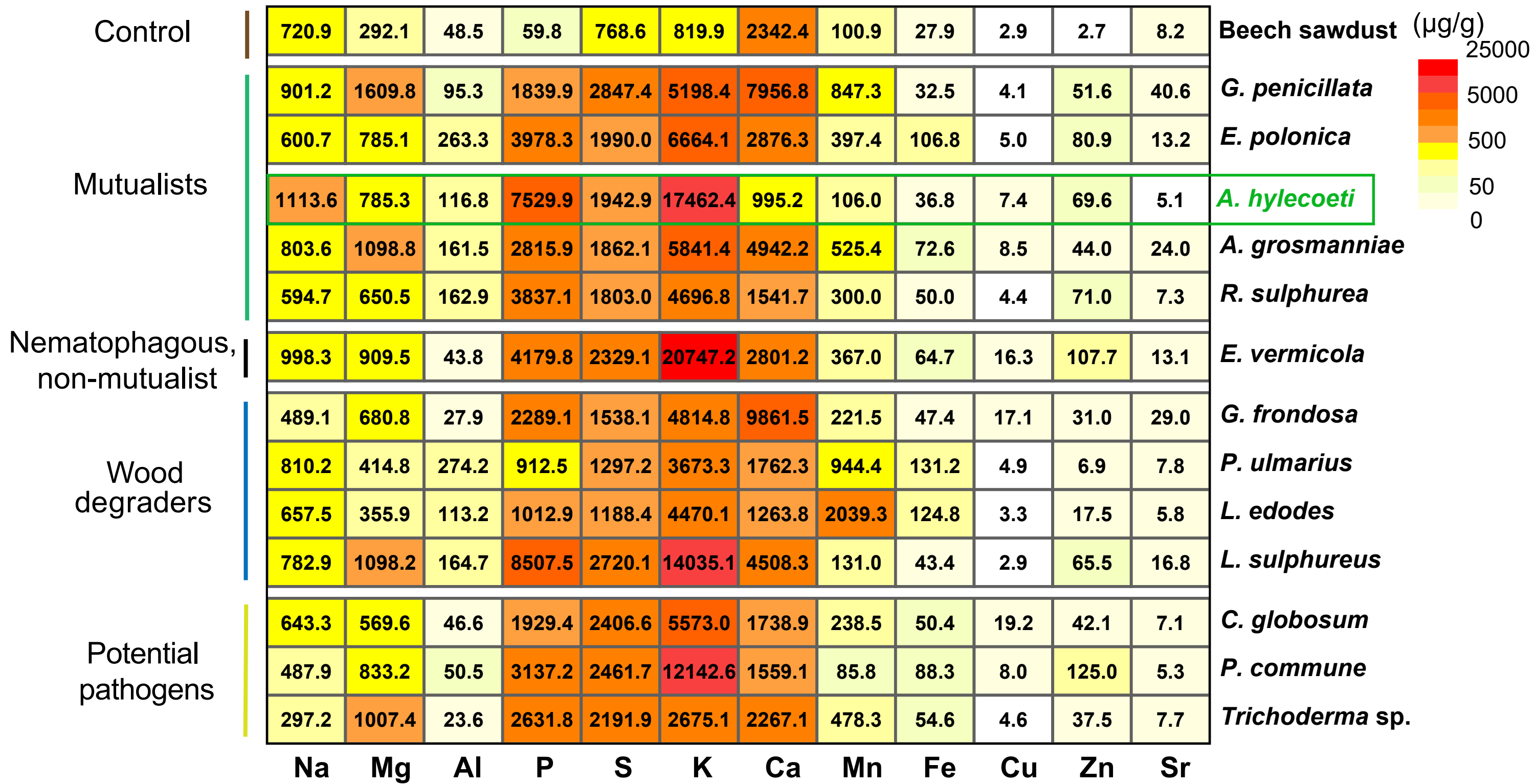

Supplement: suppl_wraf258 [file suppl_wraf258.zip › Suppl. Fig. 10.pdf]

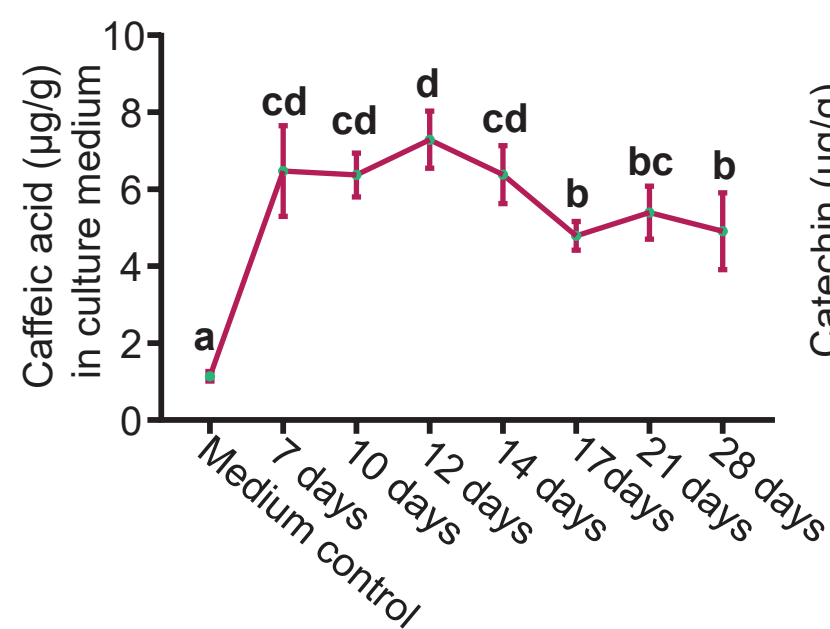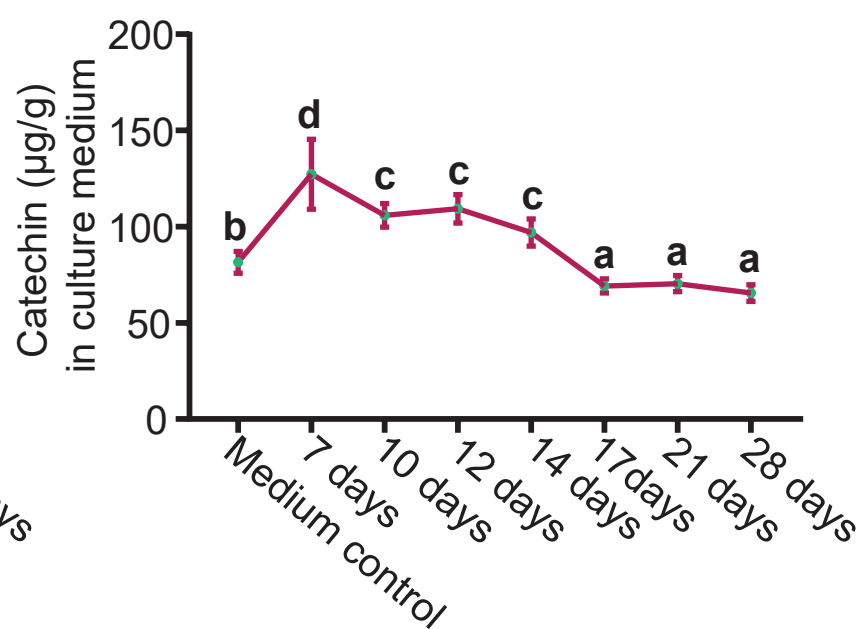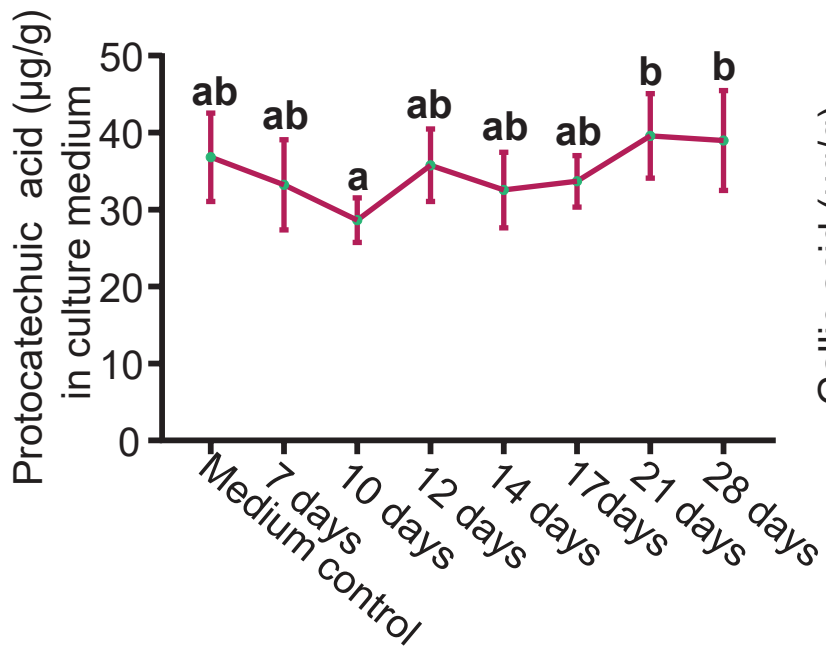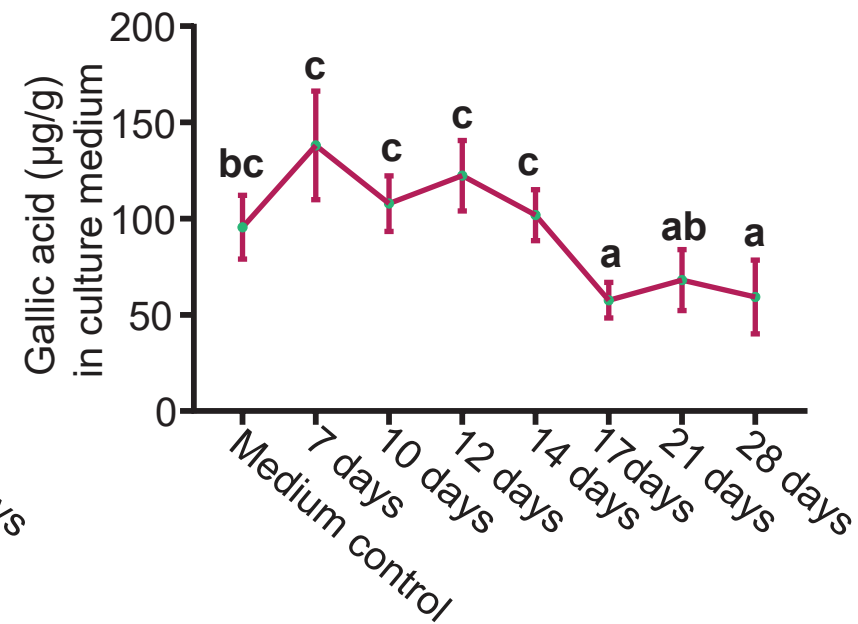

Supplement: suppl_wraf258 [file suppl_wraf258.zip › Suppl. Fig. 12.pdf]

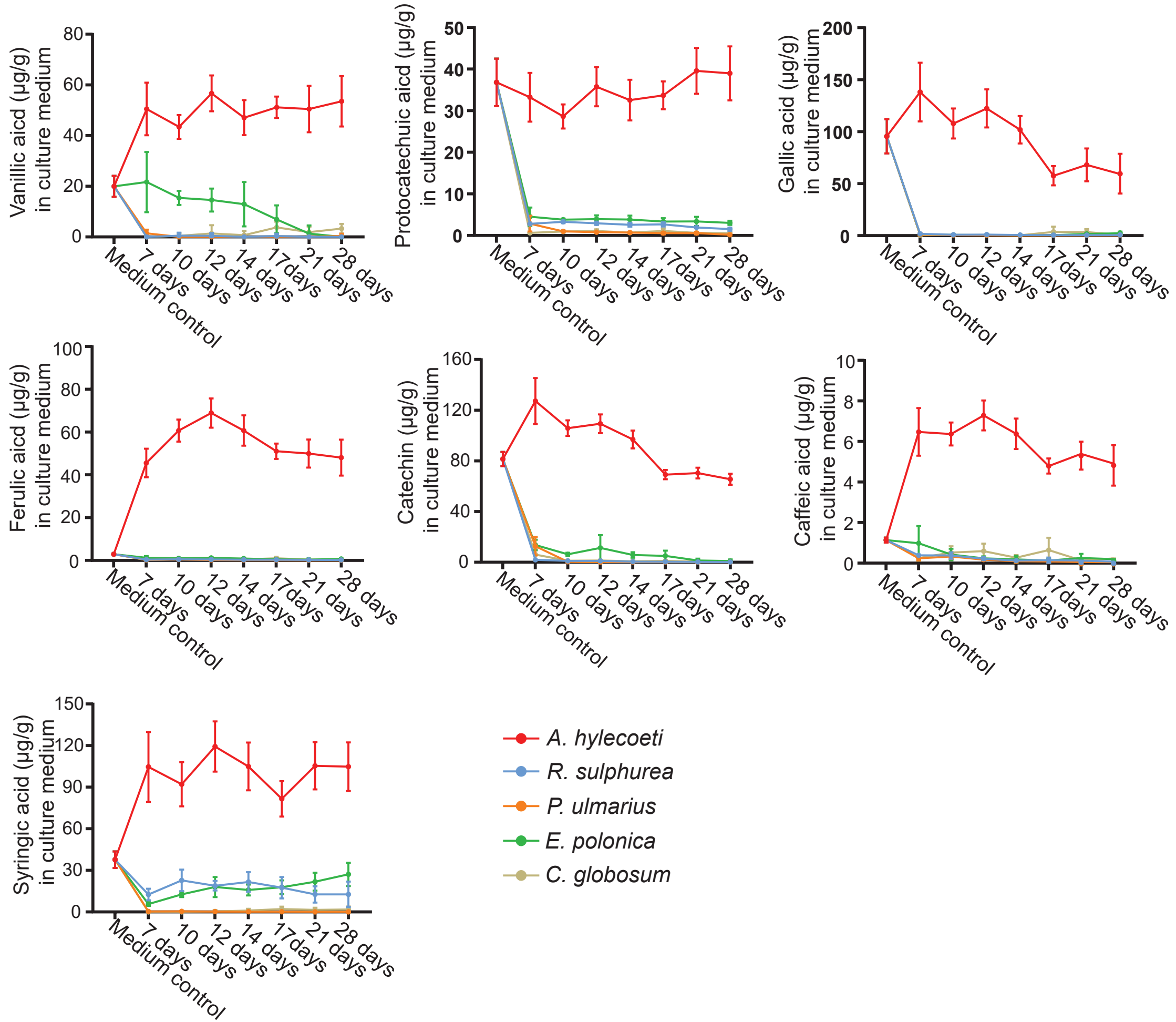

Supplement: suppl_wraf258 [file suppl_wraf258.zip › Suppl. Fig. 13.pdf]

**a**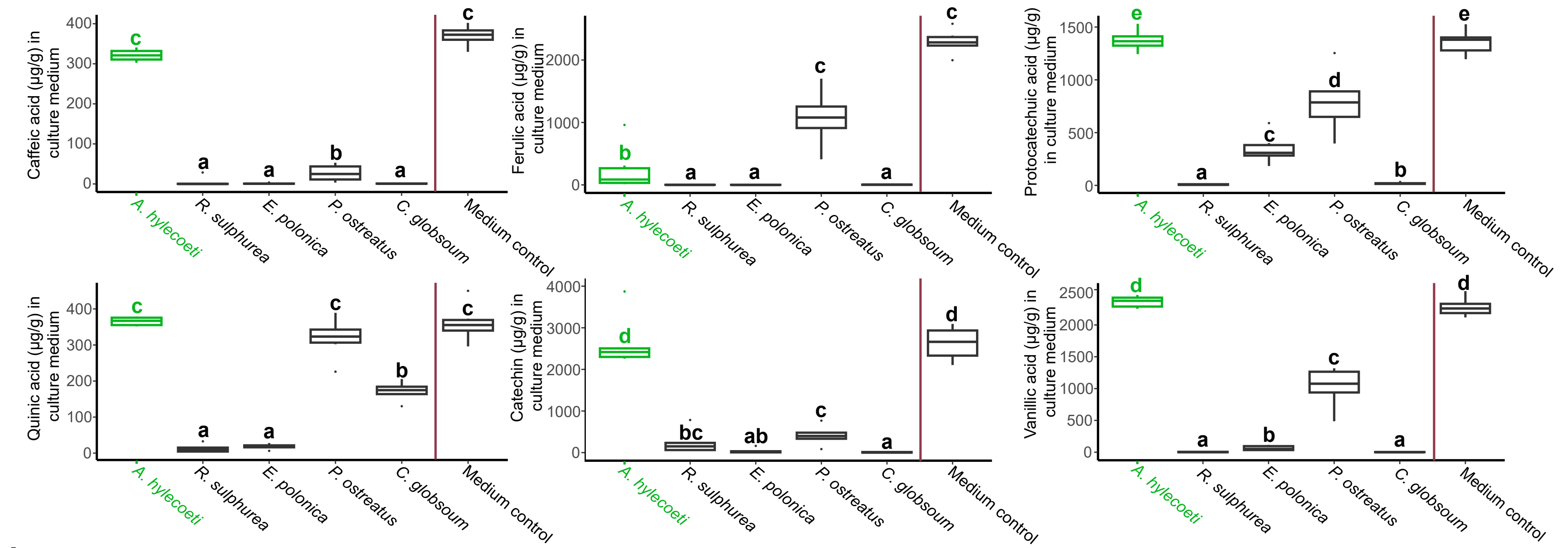**b**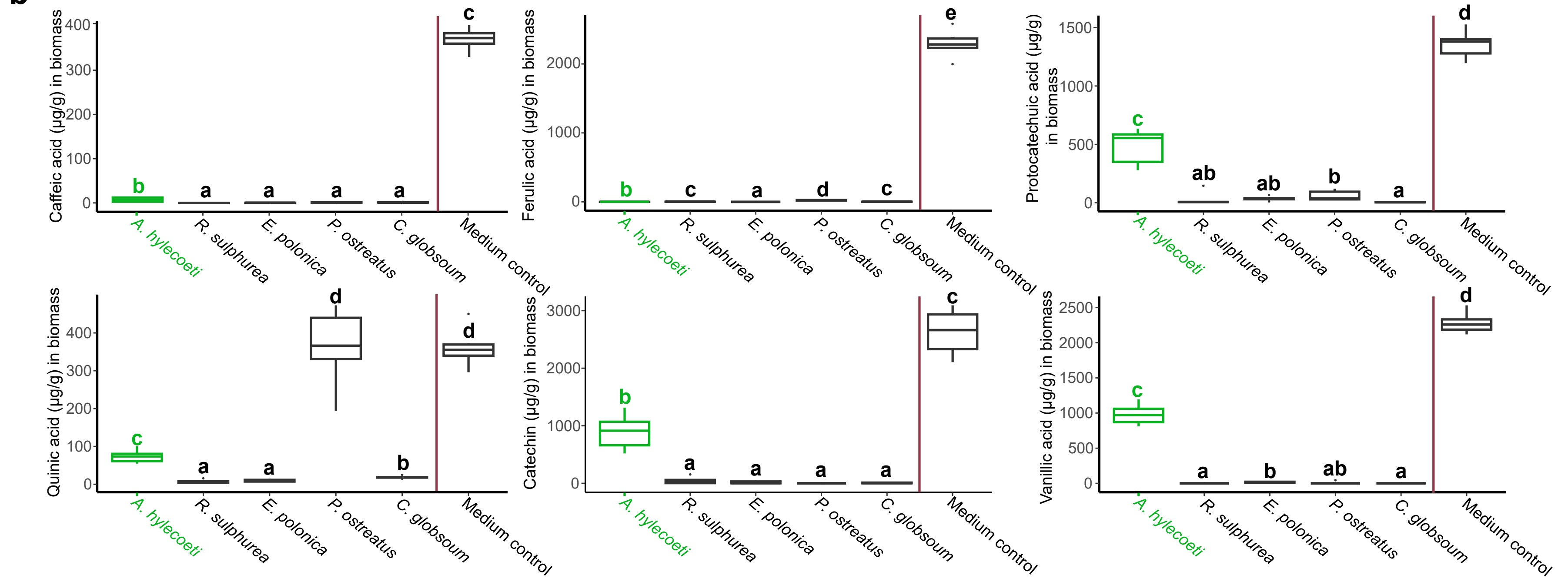

Supplement: suppl_wraf258 [file suppl_wraf258.zip › Suppl. Fig. 14.pdf]

**a**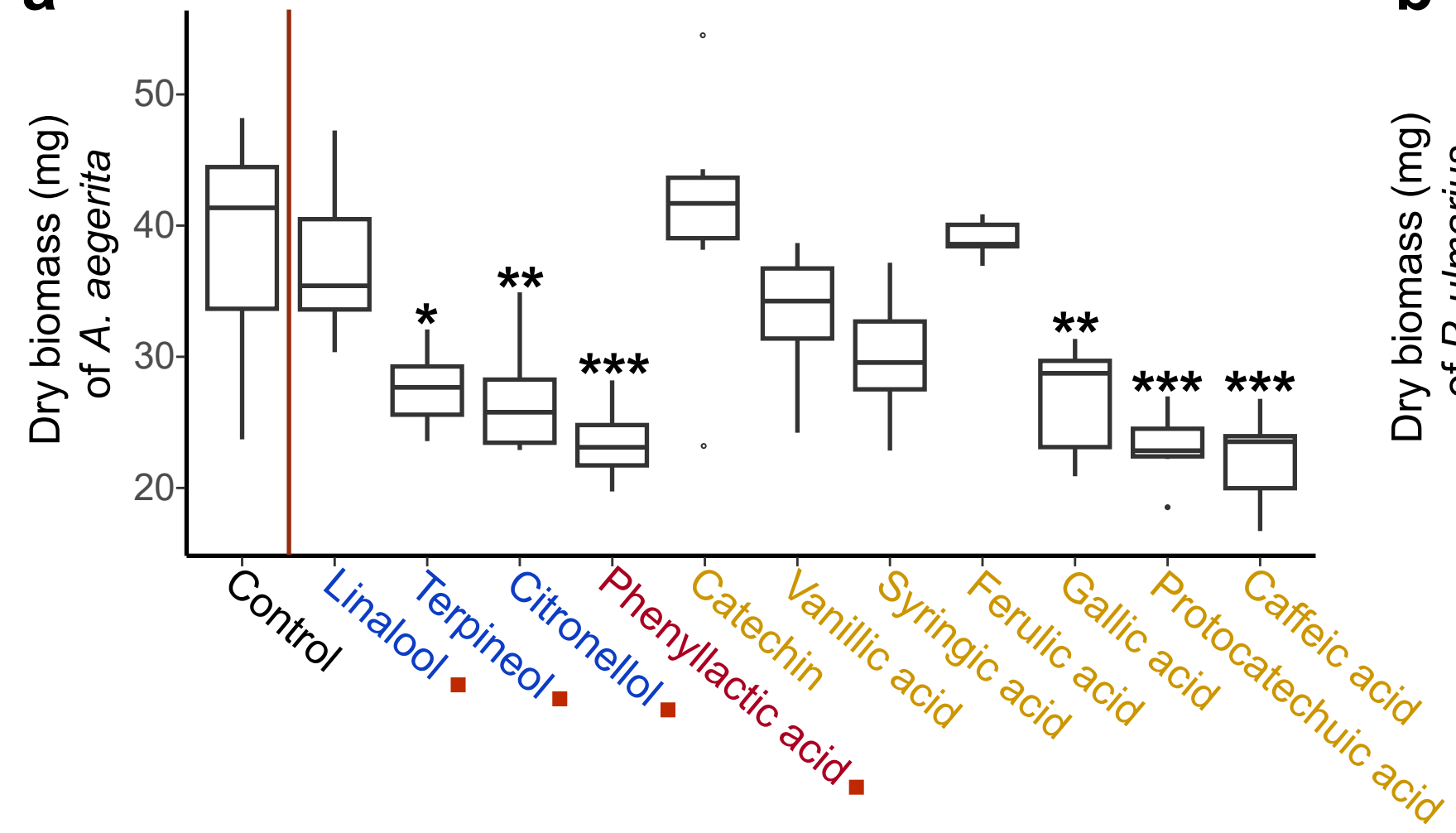**b**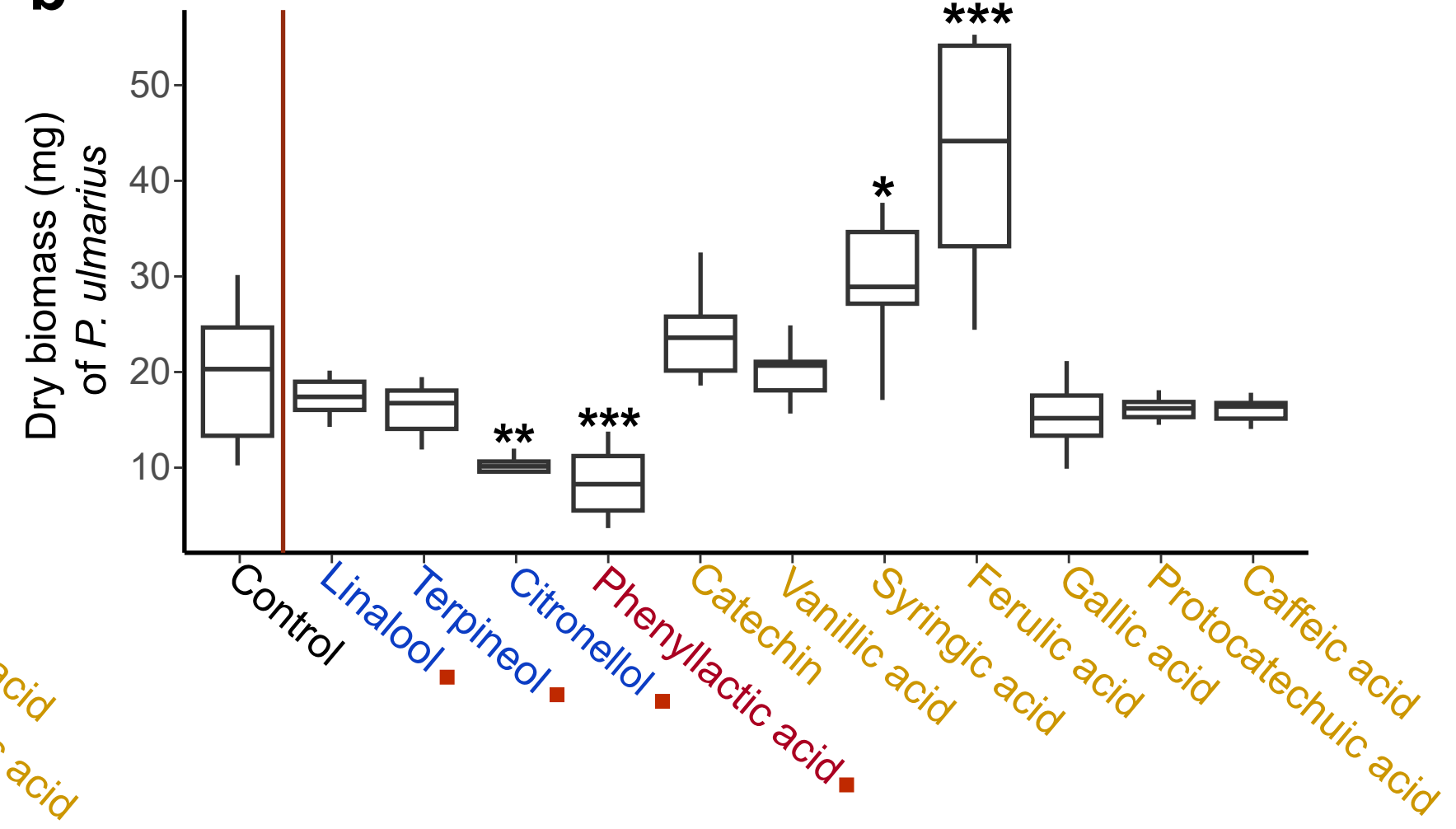

Supplement: suppl_wraf258 [file suppl_wraf258.zip › Suppl. Fig. 15.pdf]

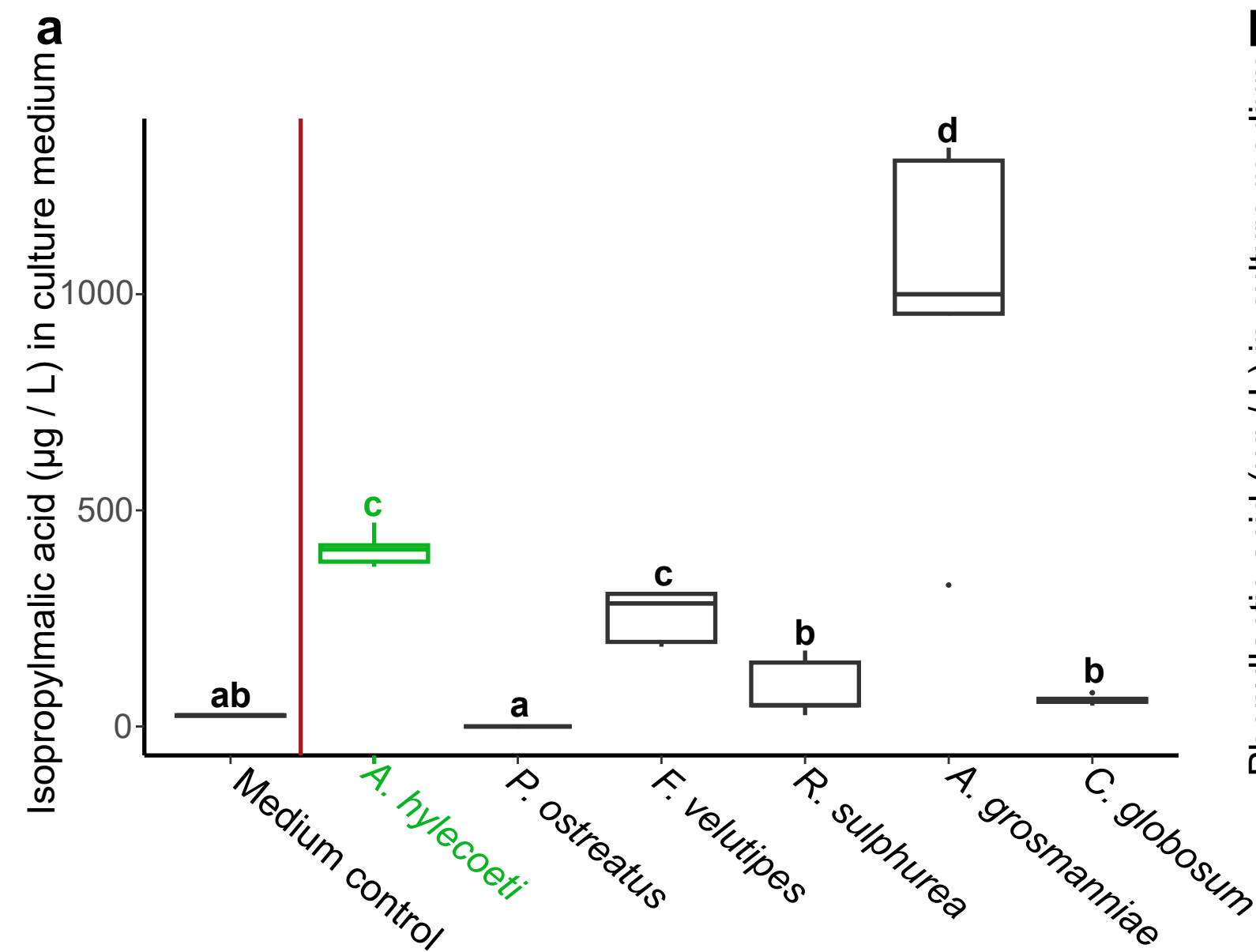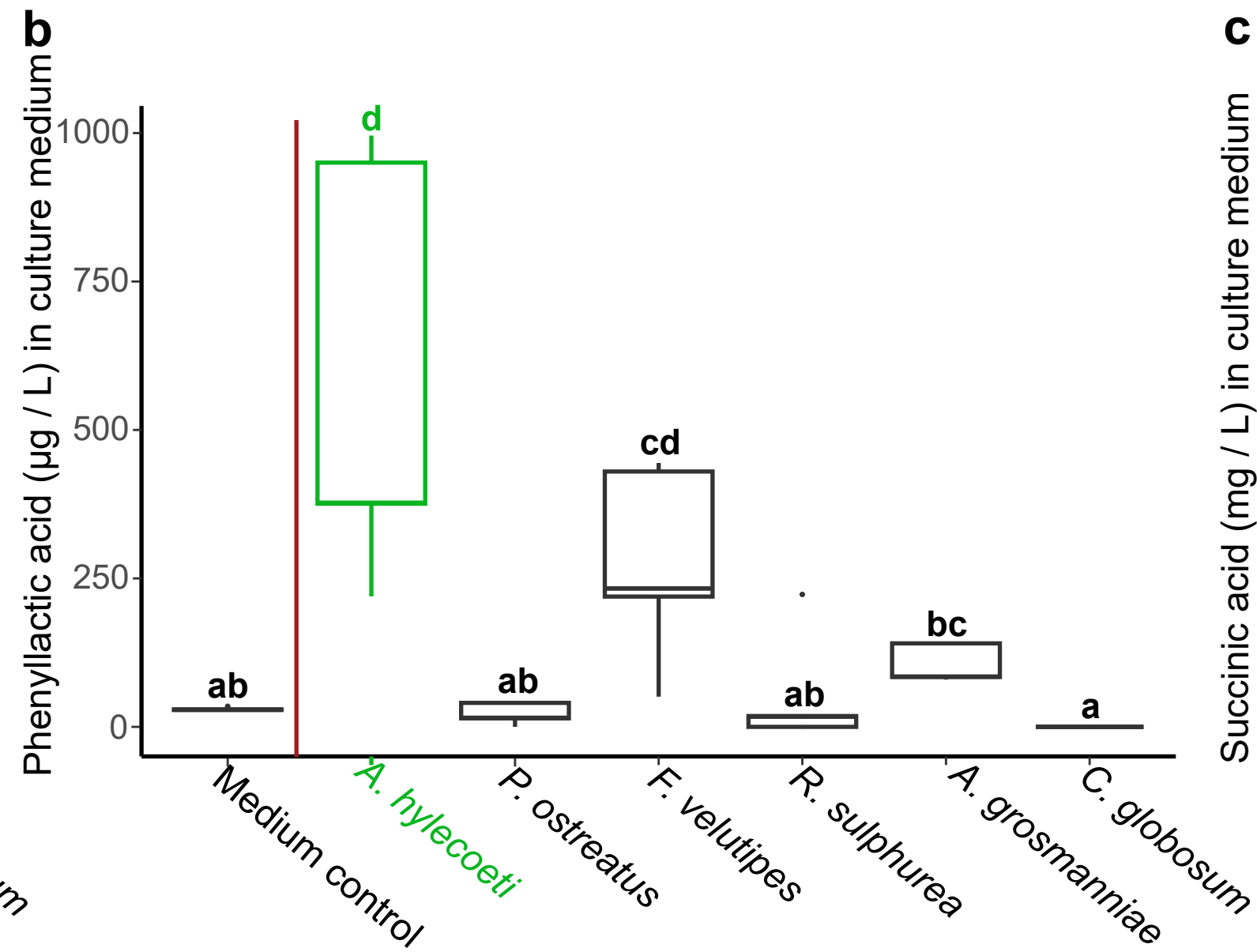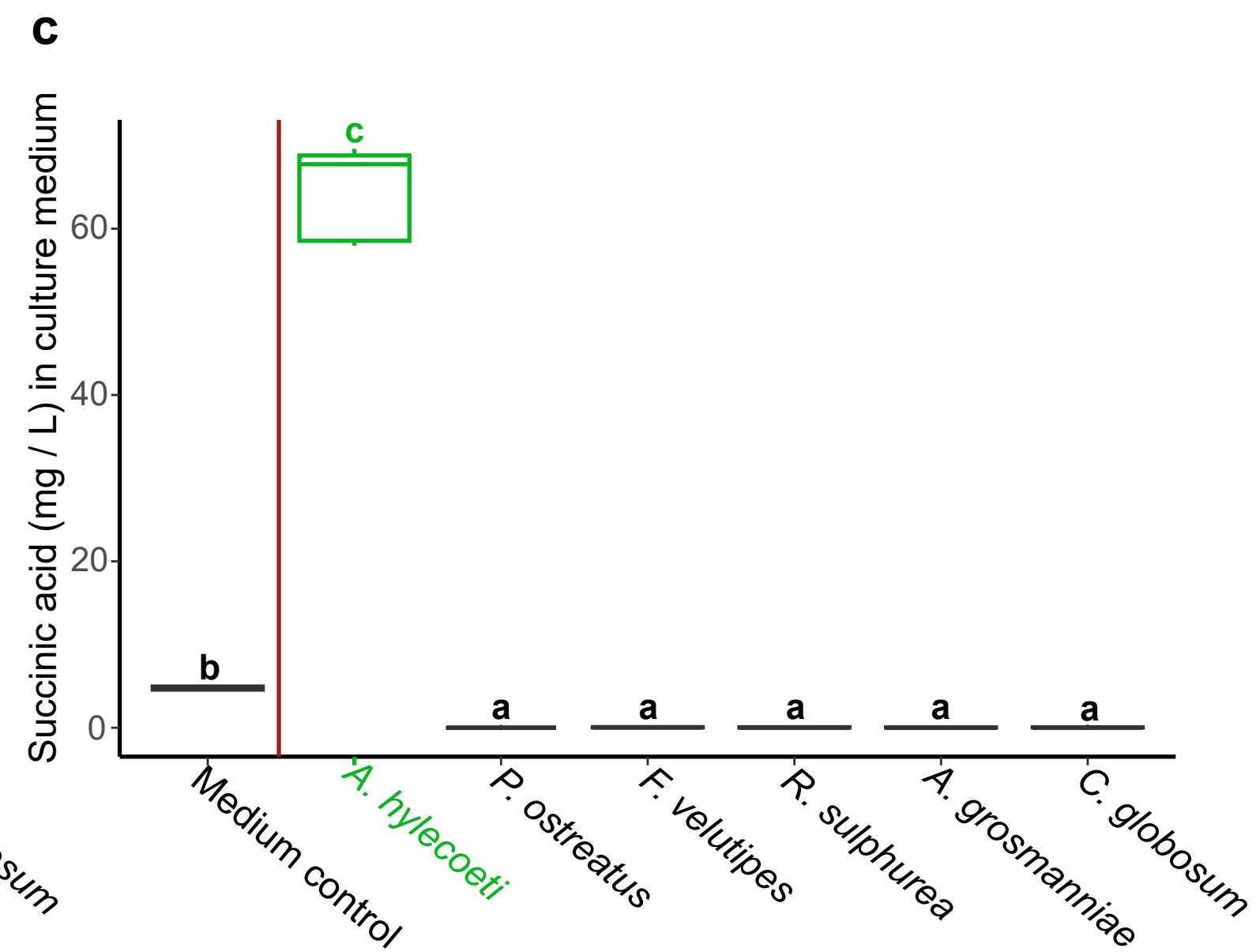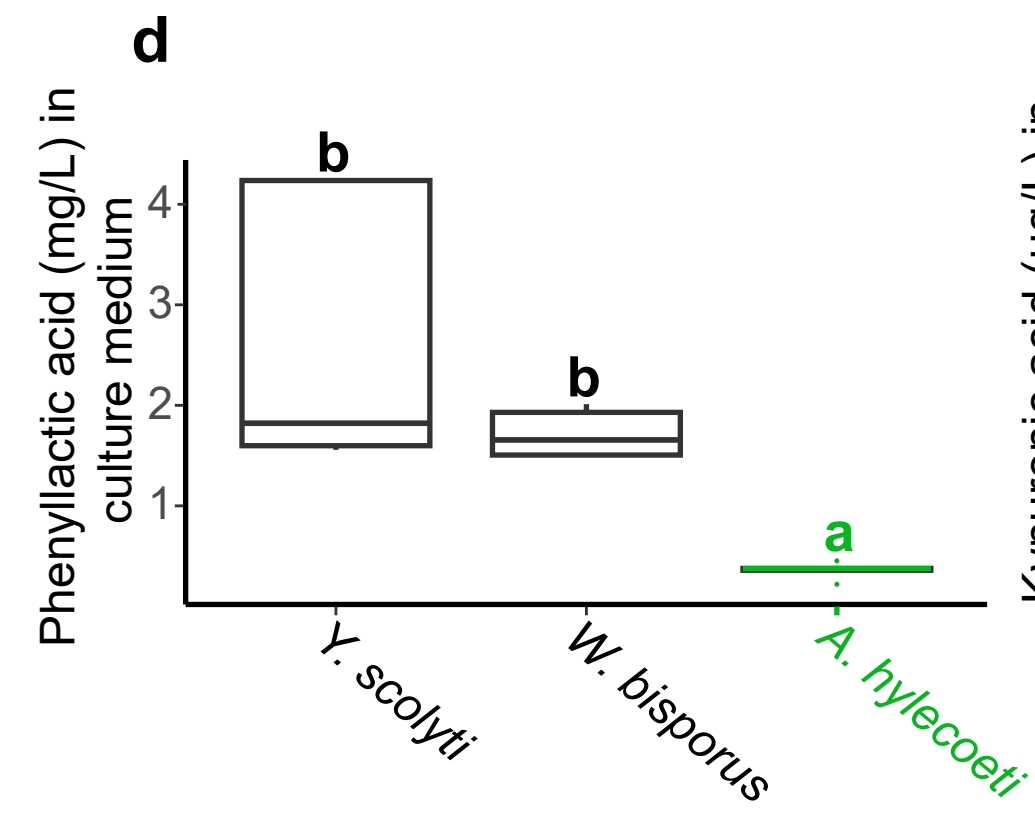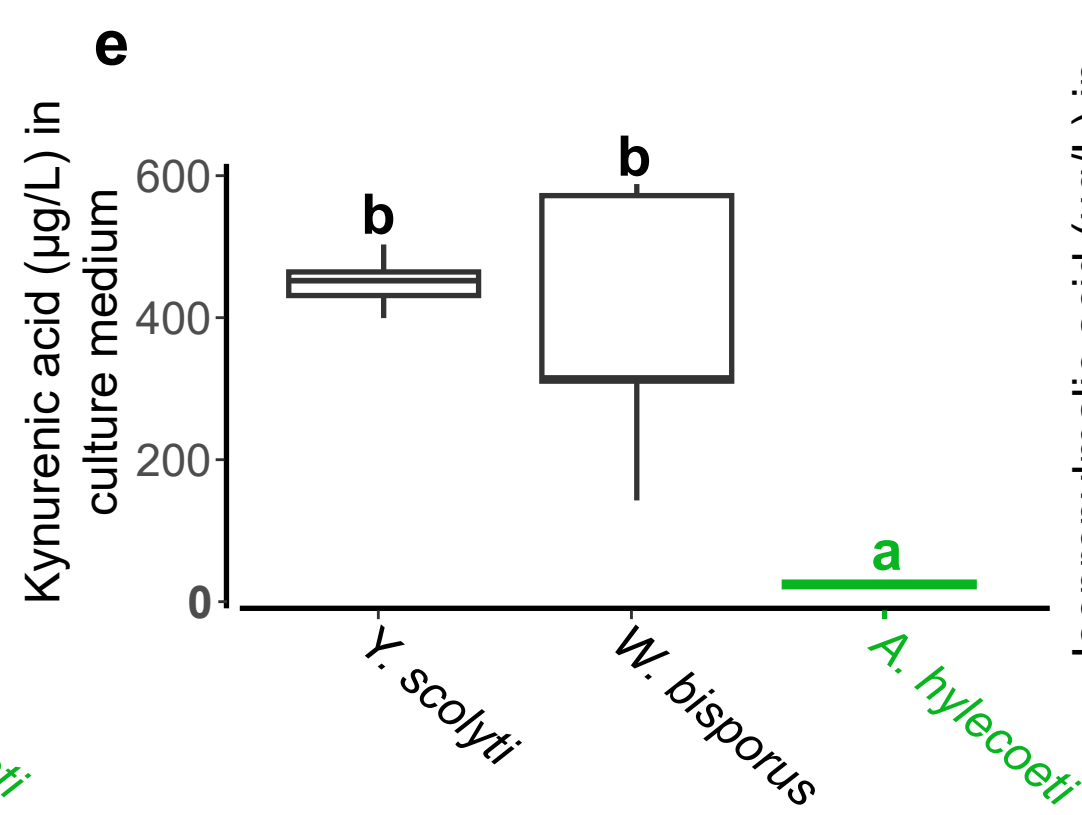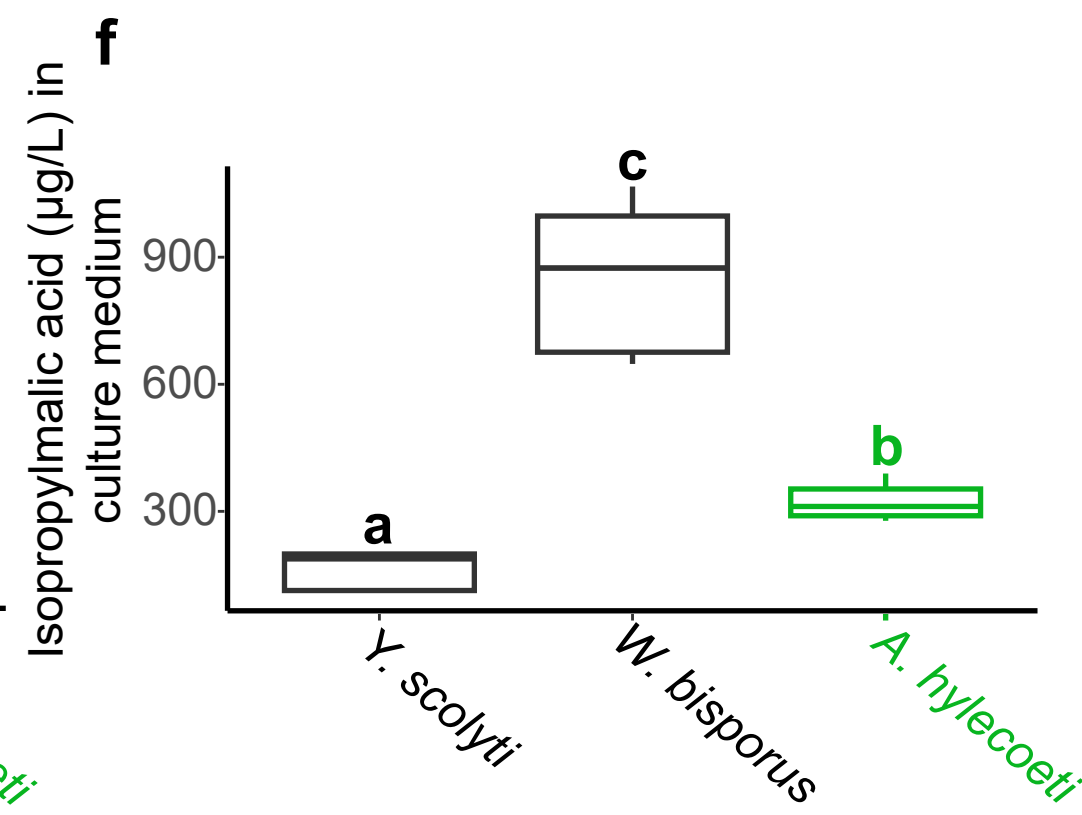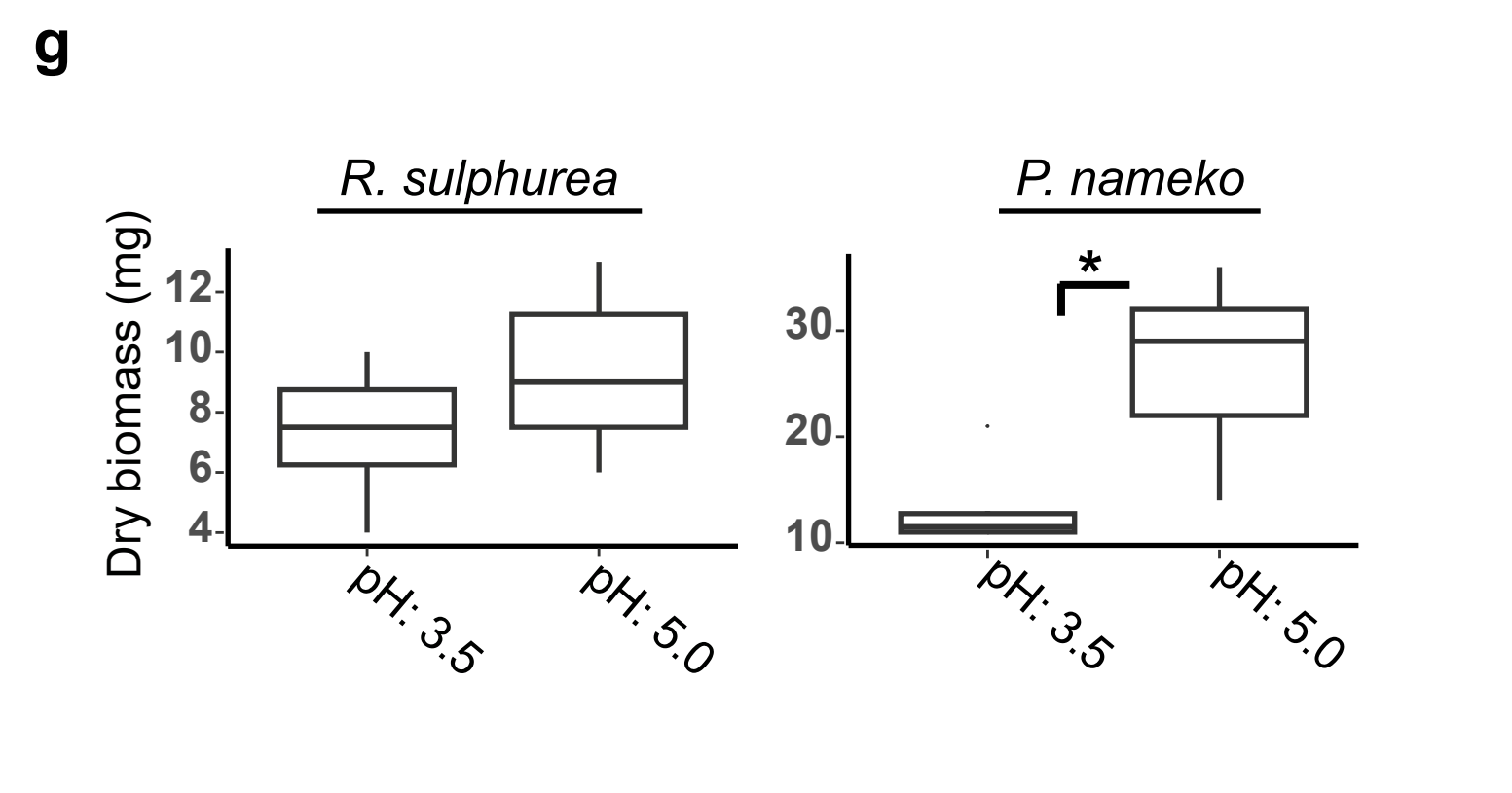

Supplement: suppl_wraf258 [file suppl_wraf258.zip › Suppl. Fig. 16.pdf]

*P. ostreatus*

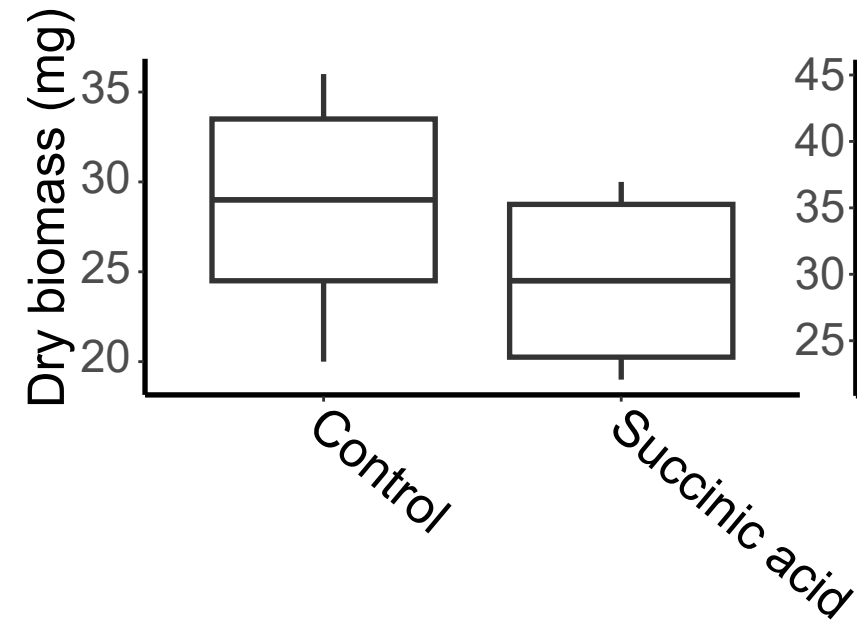

*P. nameko*

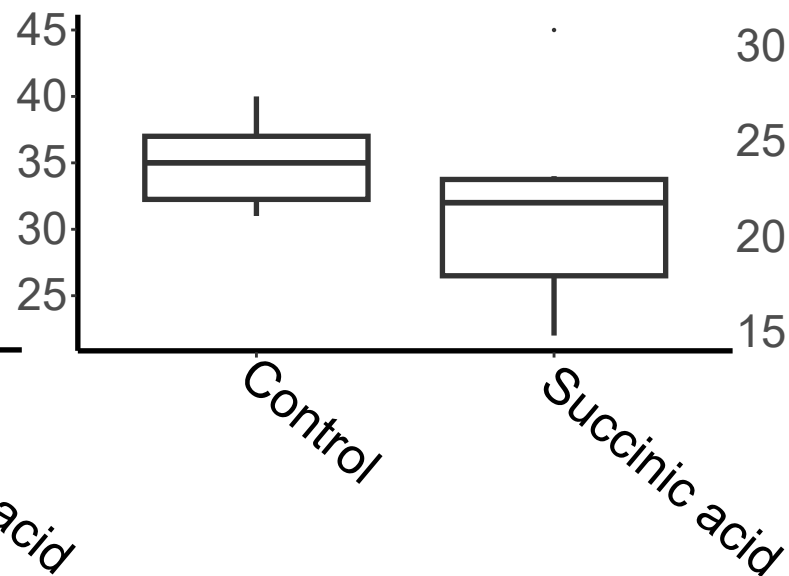

*A. aegerita*

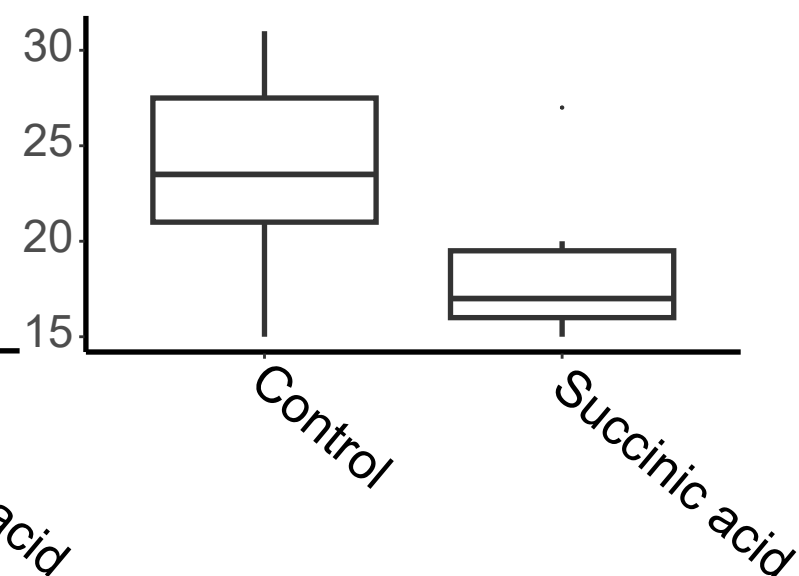

*C. globosum*

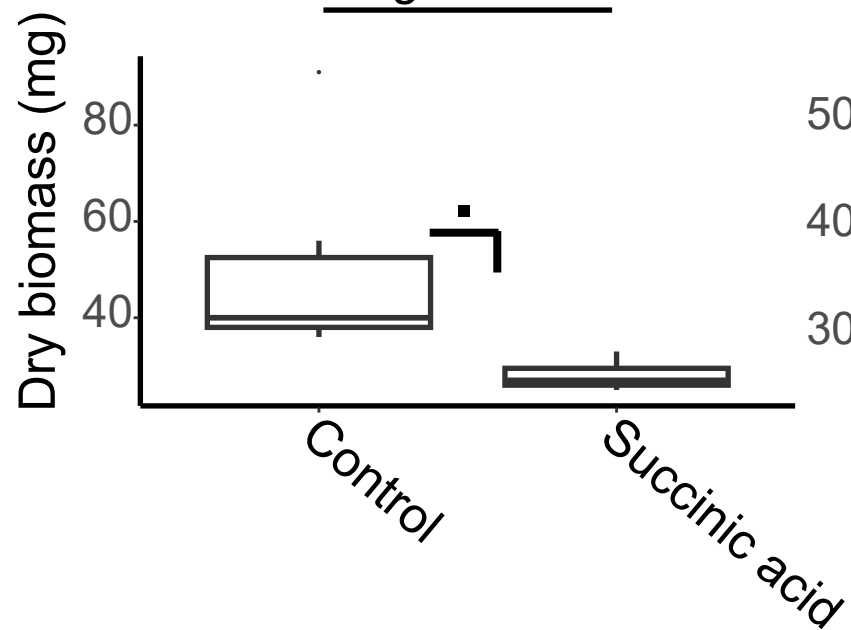

*A. hylecoeti*

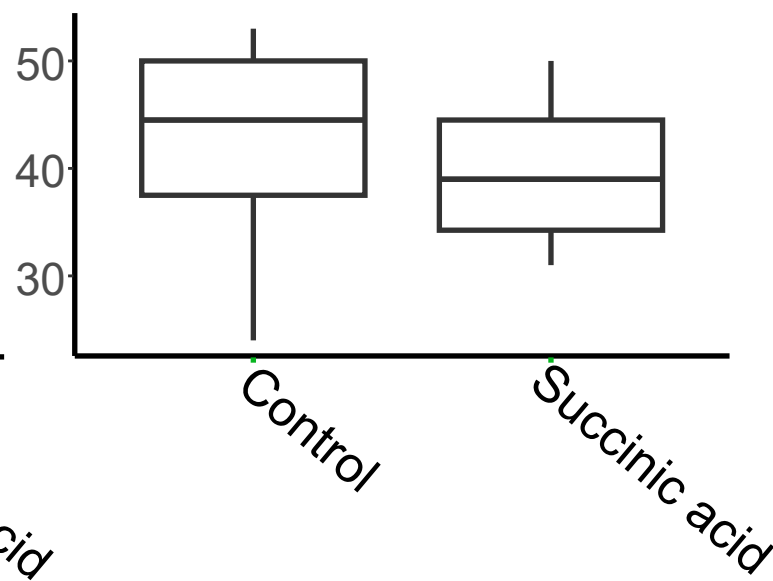

Supplement: suppl_wraf258 [file suppl_wraf258.zip › Suppl. Fig. 17.pdf]

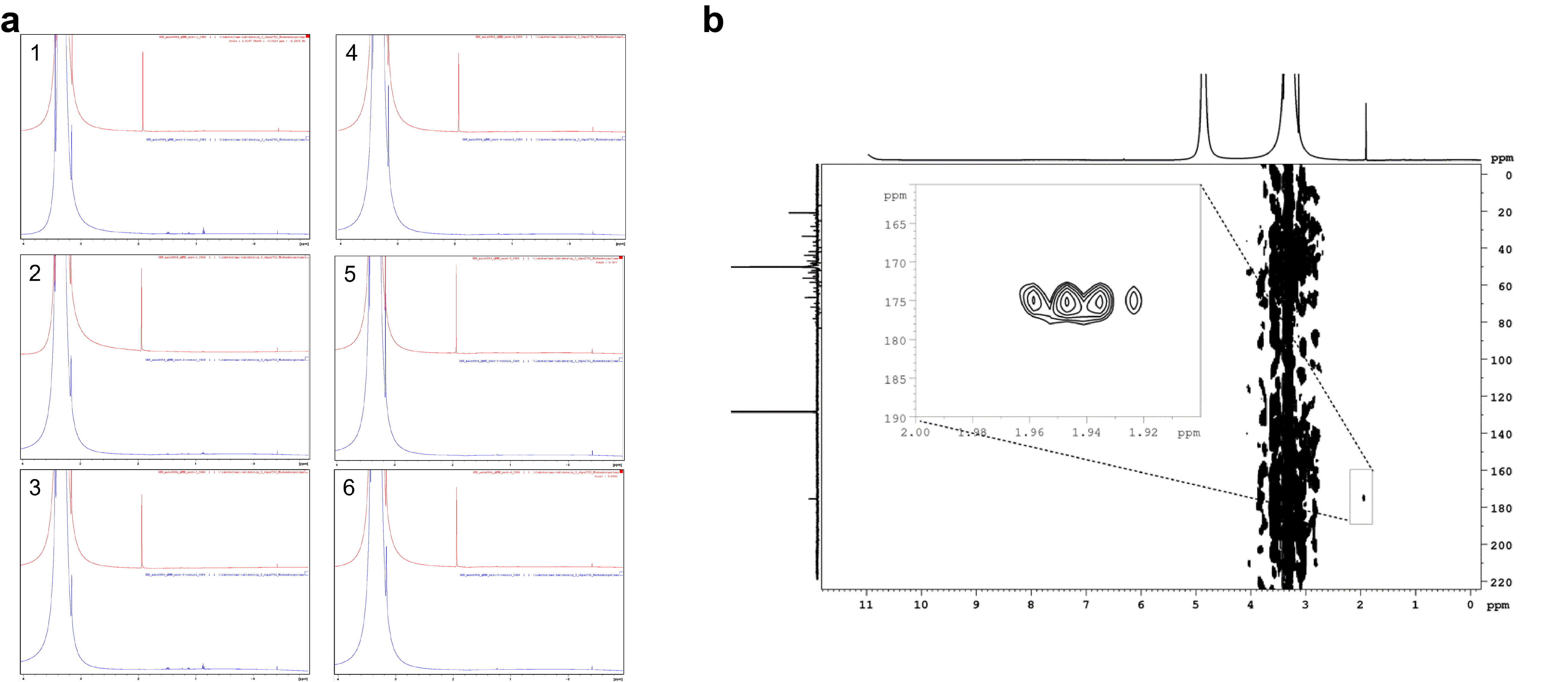

Supplement: suppl_wraf258 [file suppl_wraf258.zip › Suppl. Fig. 18.png]

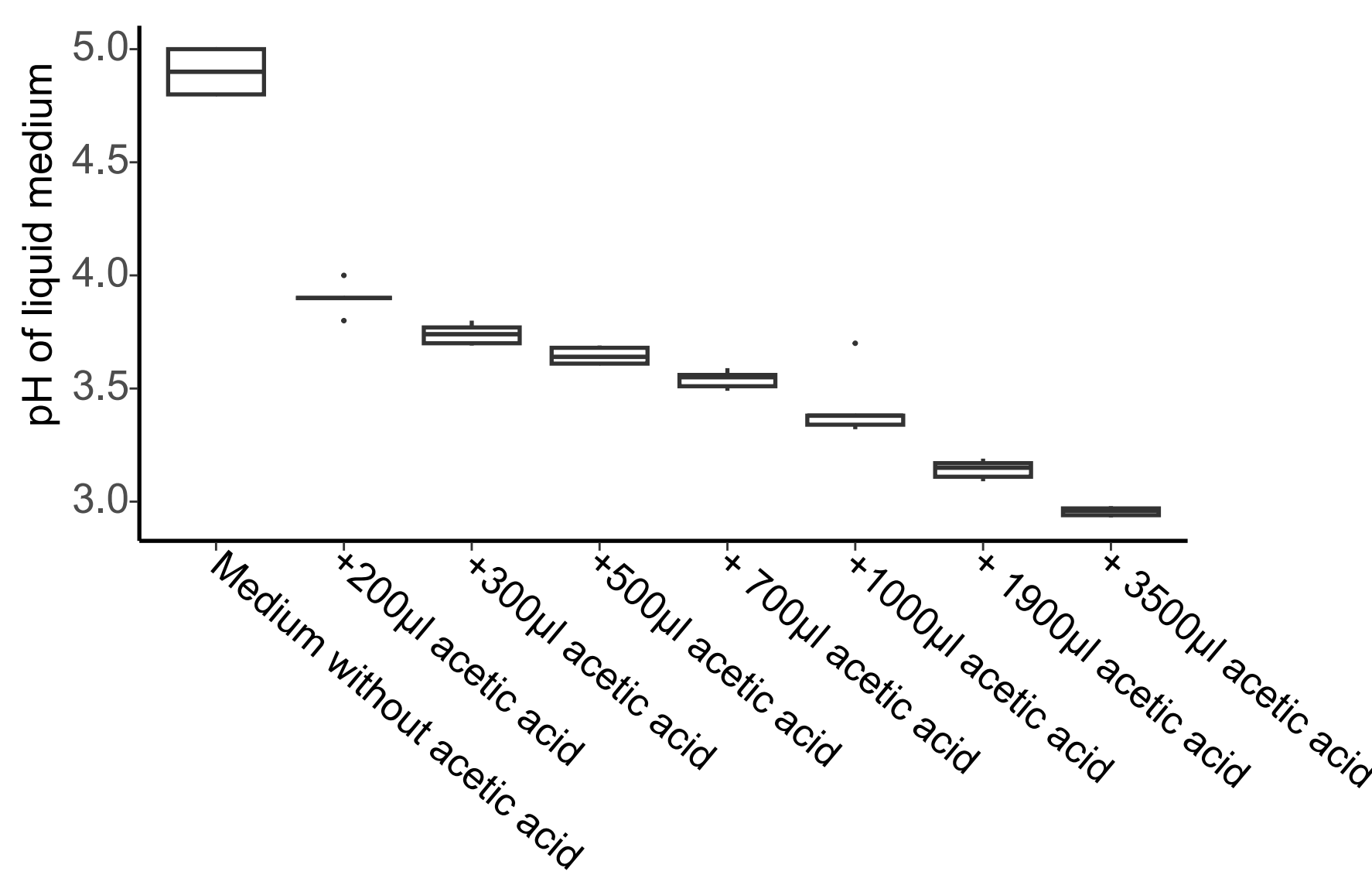

Supplement: suppl_wraf258 [file suppl_wraf258.zip › Suppl. Fig. 19.pdf]

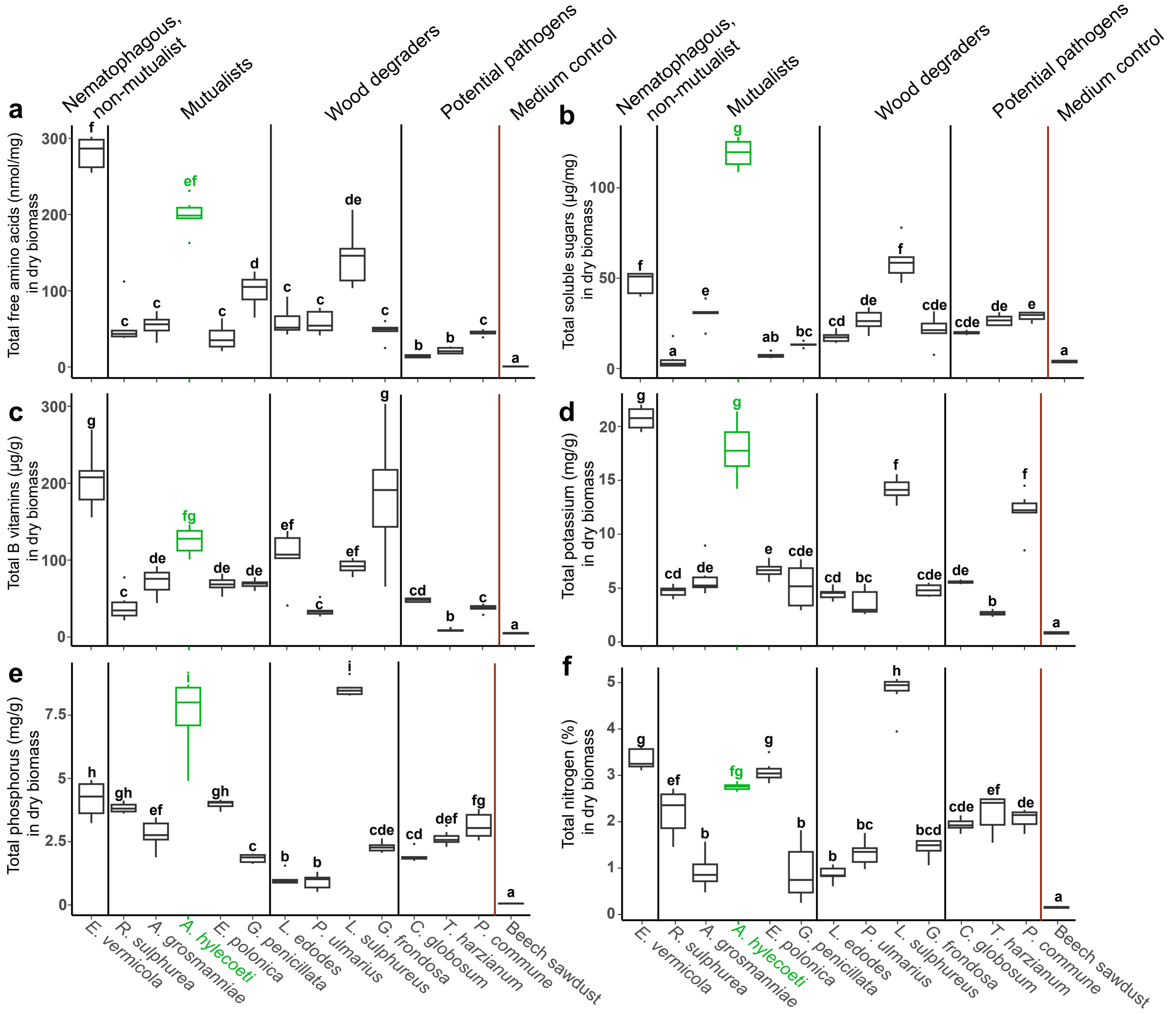

Supplement: suppl_wraf258 [file suppl_wraf258.zip › Suppl. Fig. 2.pdf]

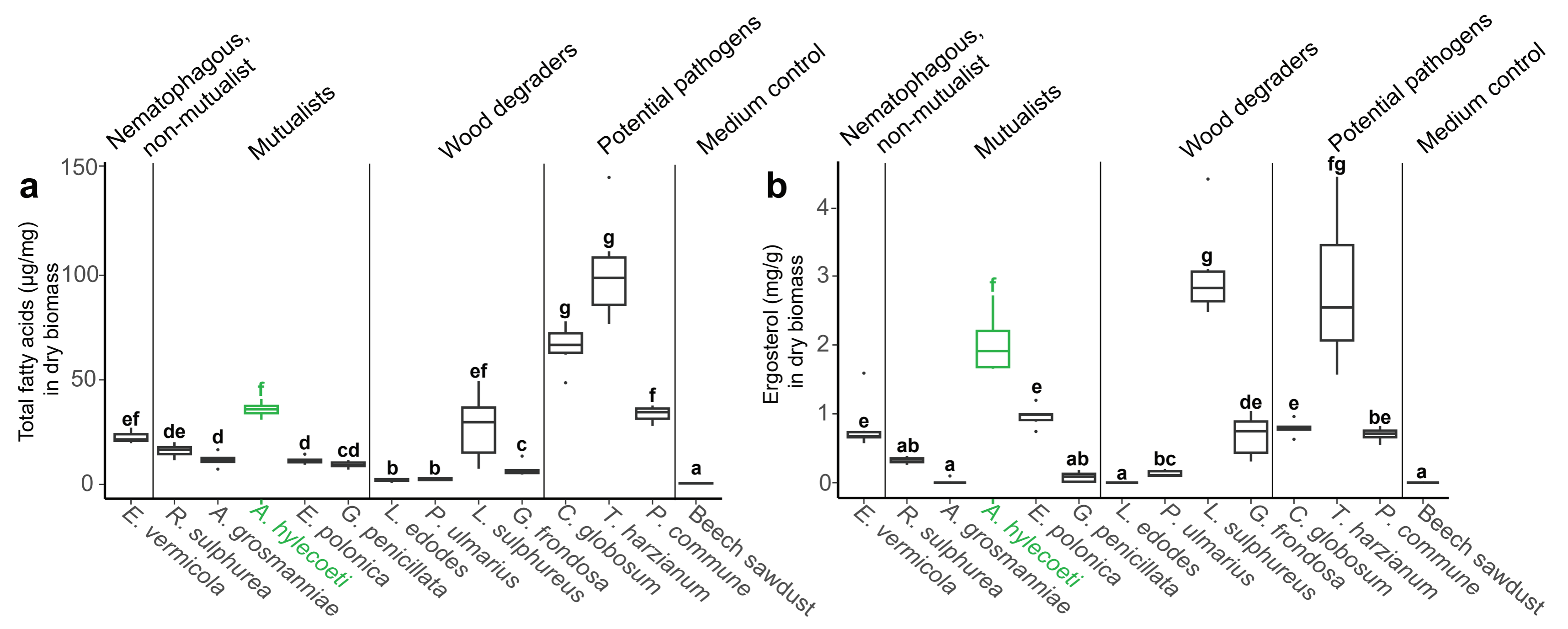

Supplement: suppl_wraf258 [file suppl_wraf258.zip › Suppl. Fig. 3.pdf]

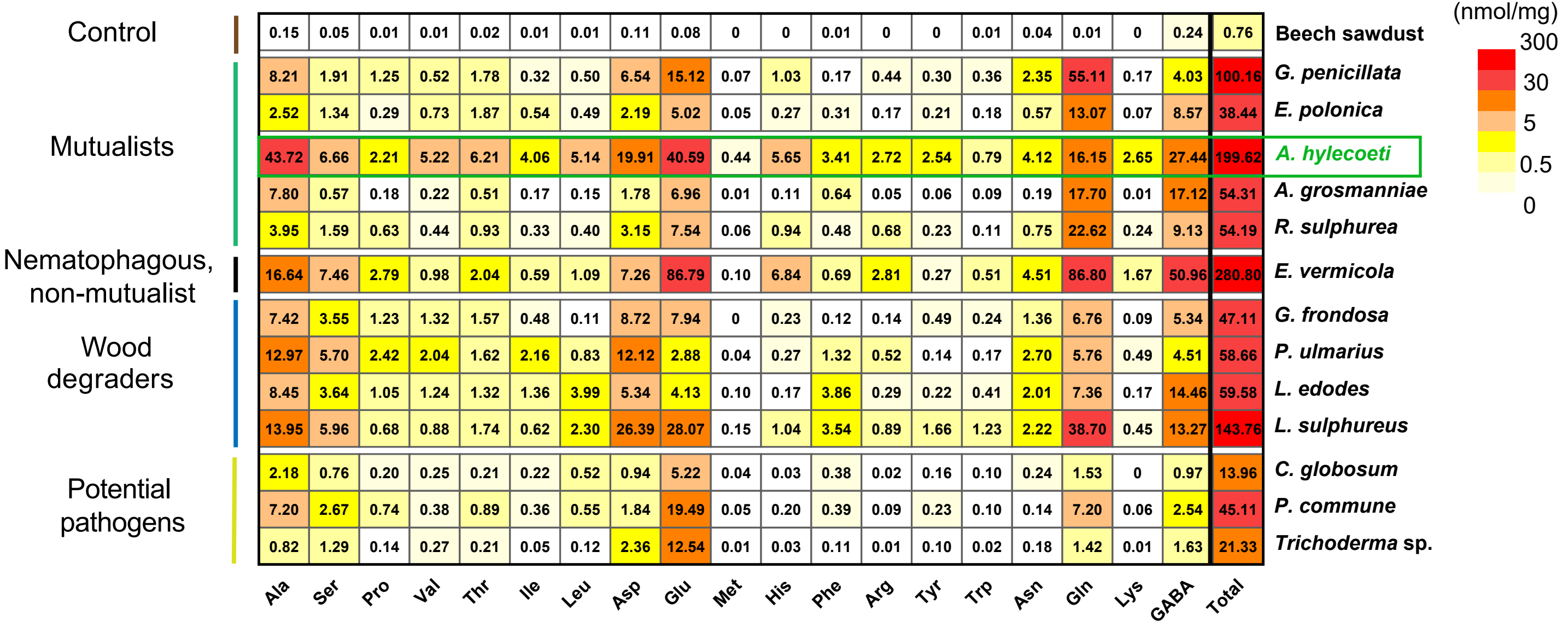

Supplement: suppl_wraf258 [file suppl_wraf258.zip › Suppl. Fig. 4.pdf]

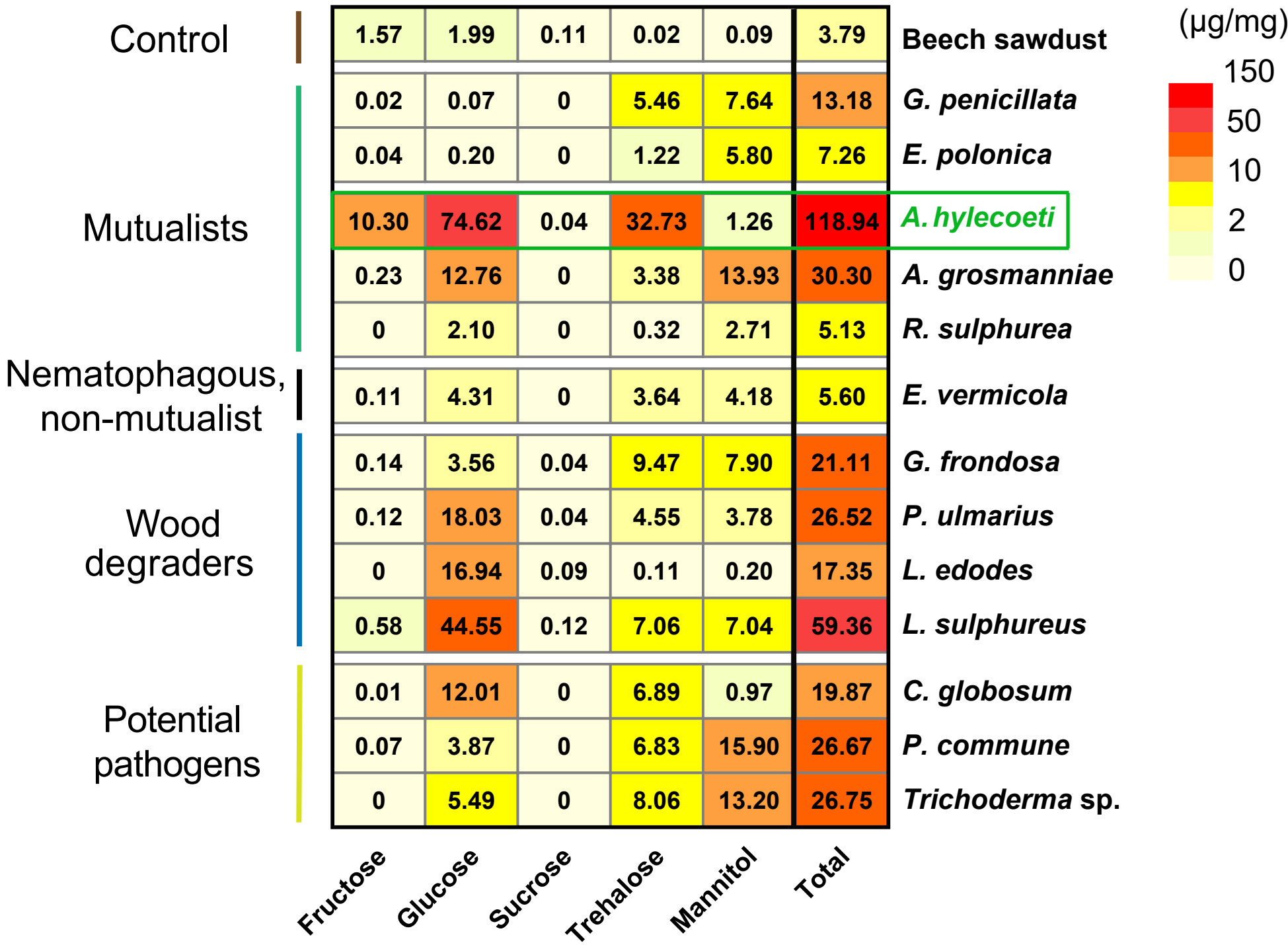

Supplement: suppl_wraf258 [file suppl_wraf258.zip › Suppl. Fig. 5.pdf]

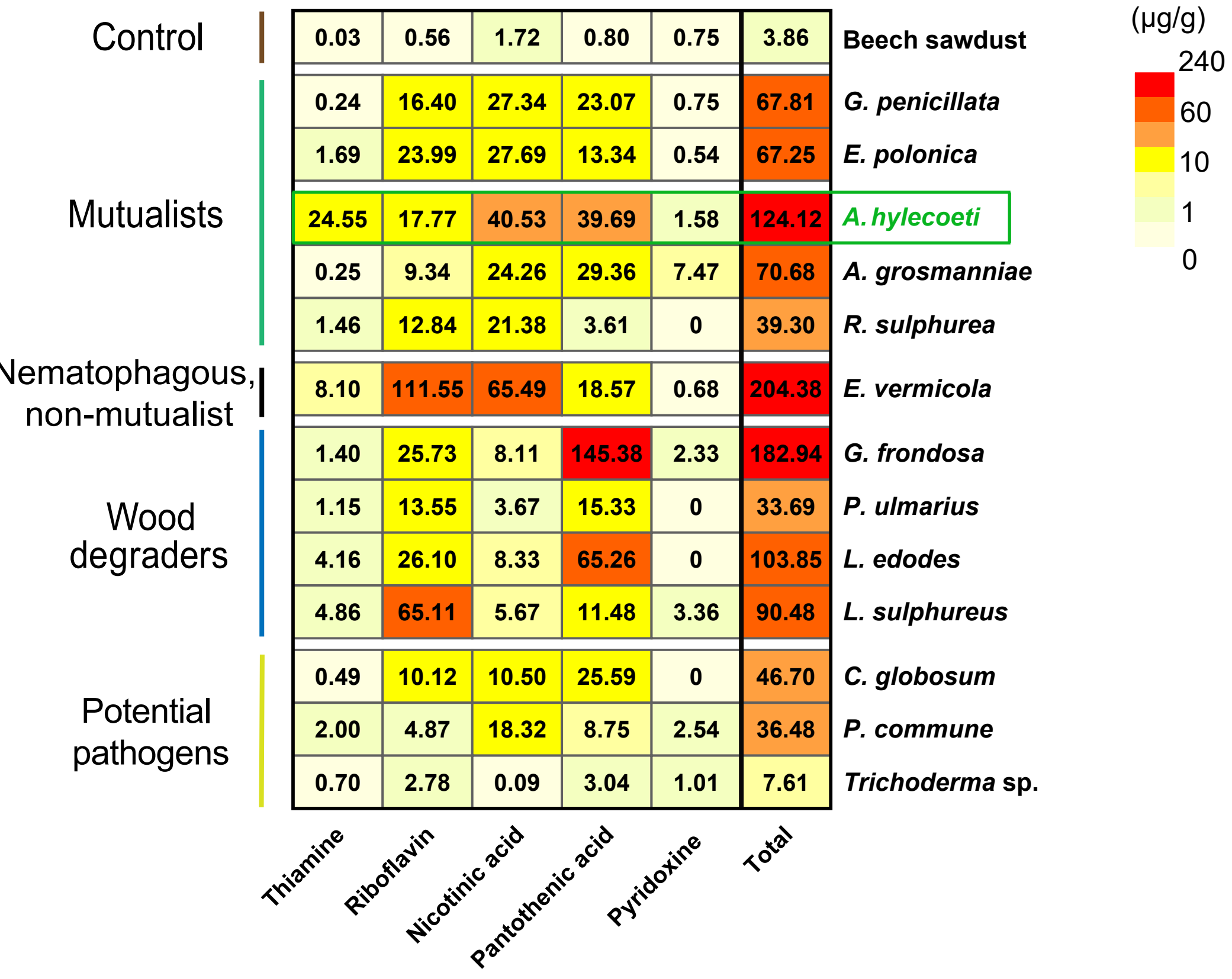

Supplement: suppl_wraf258 [file suppl_wraf258.zip › Suppl. Fig. 6.pdf]

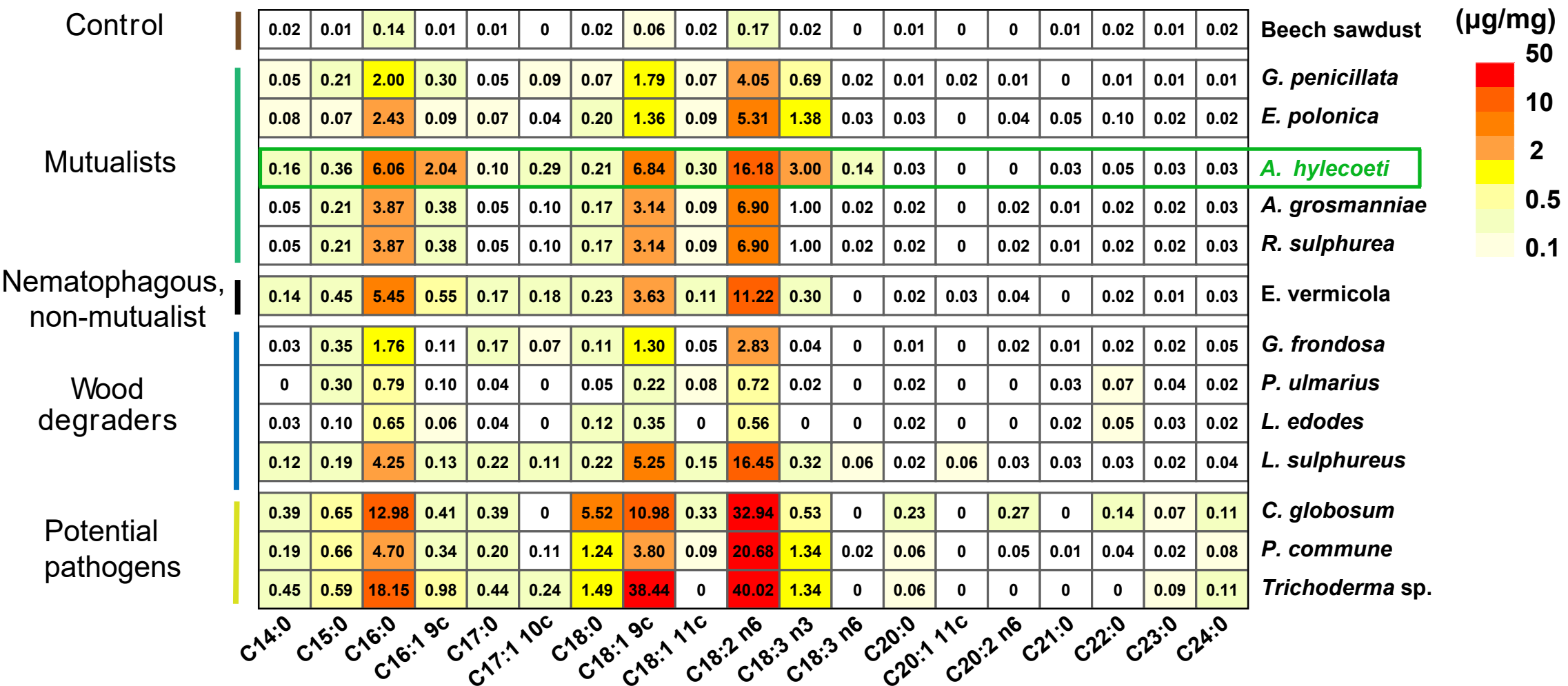

Supplement: suppl_wraf258 [file suppl_wraf258.zip › Suppl. Fig. 7.pdf]

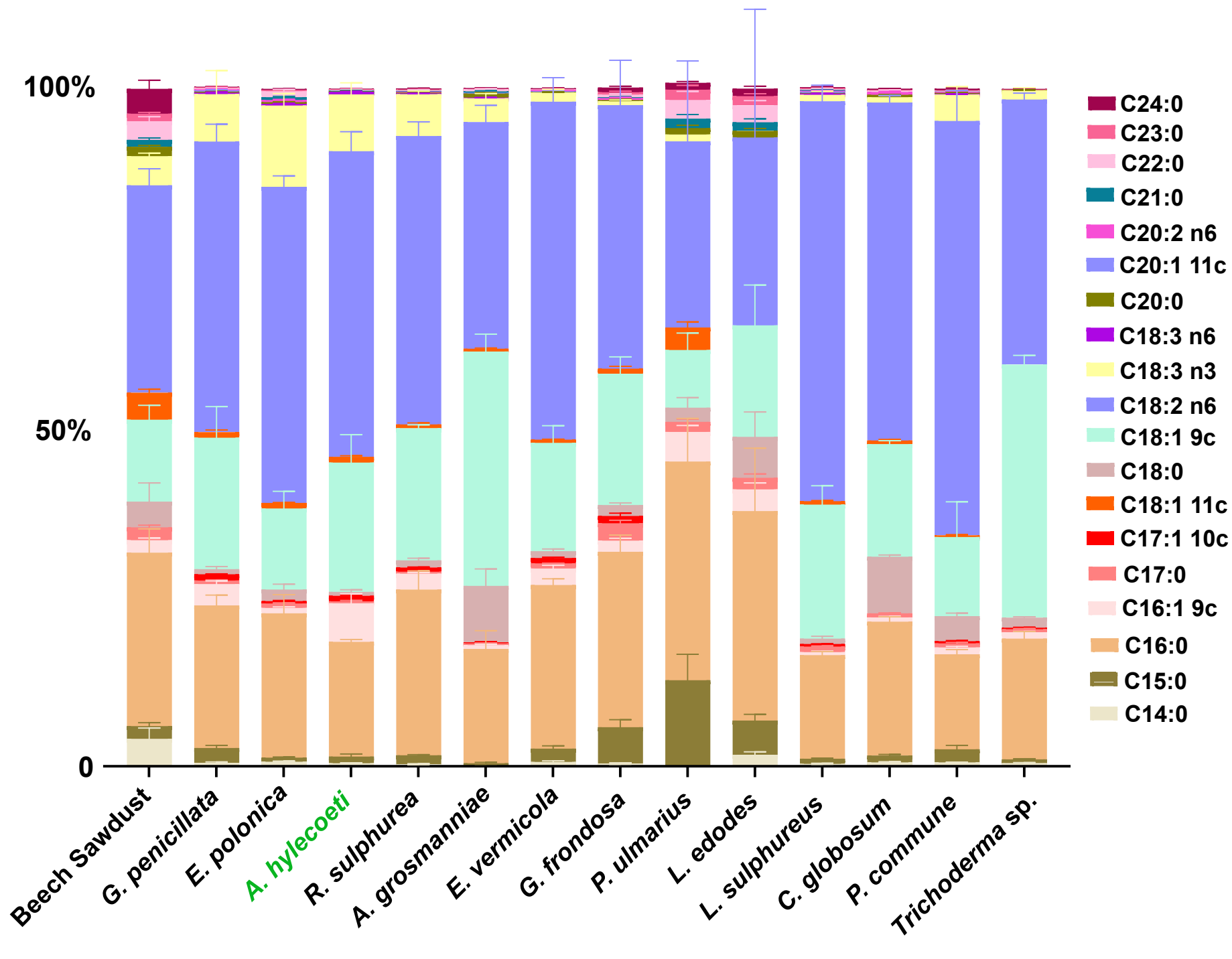

Supplement: suppl_wraf258 [file suppl_wraf258.zip › Suppl. Fig. 8.pdf]

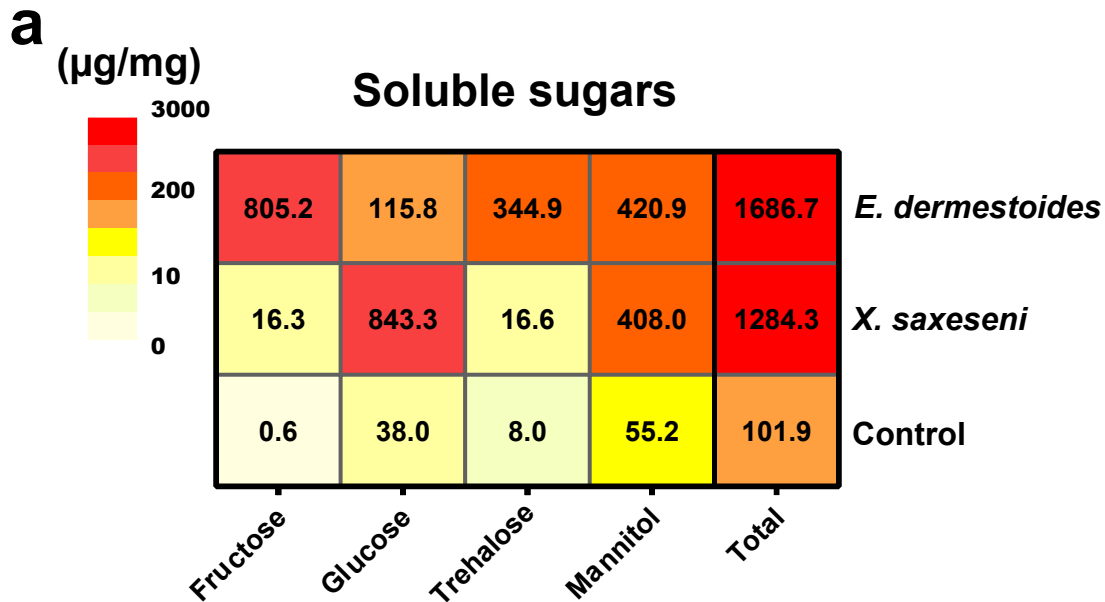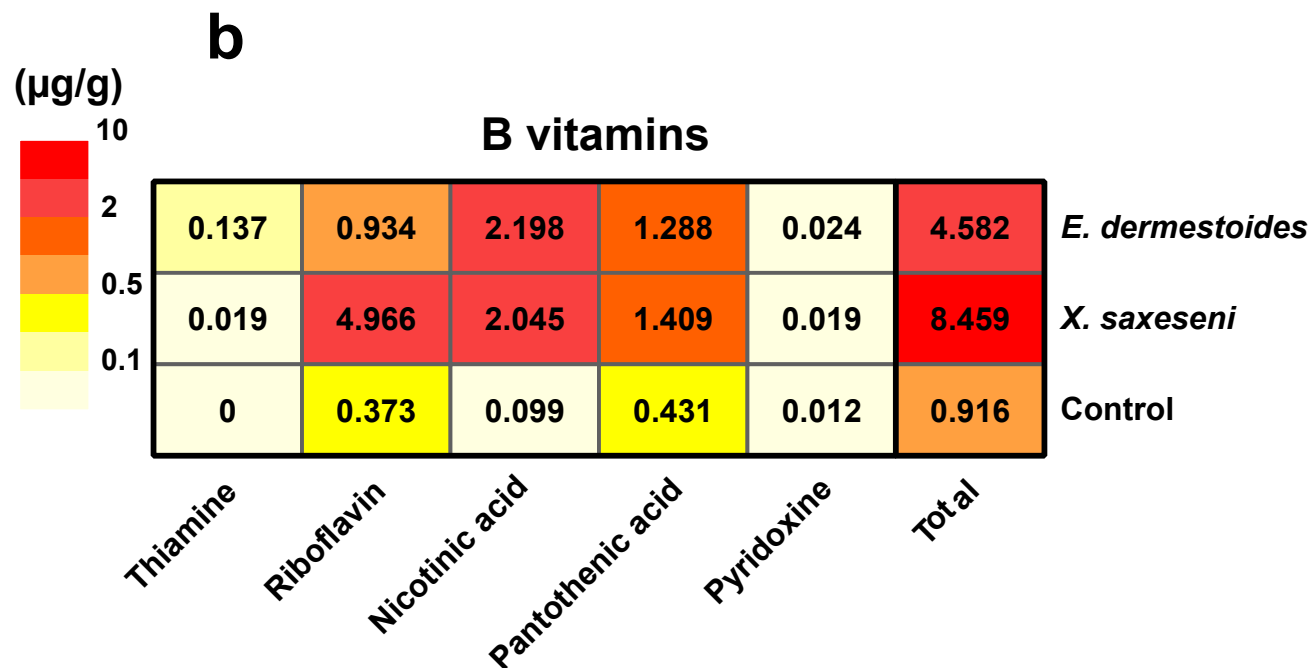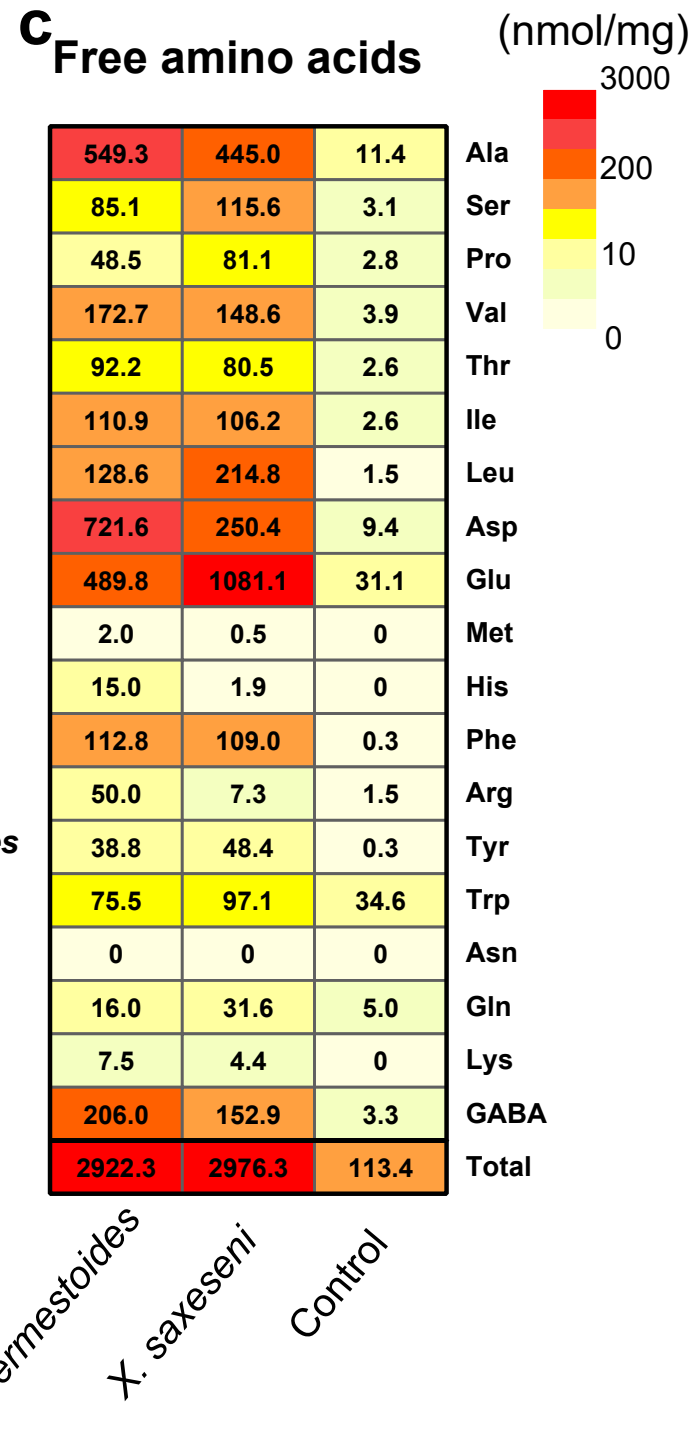

Supplement: suppl_wraf258 [file suppl_wraf258.zip › Suppl. Fig. 9.pdf]
